# Loss of IL1RA promotes prostate cancer growth and metastasis by activating Akt signaling pathway

**DOI:** 10.1371/journal.pone.0339611

**Published:** 2026-02-02

**Authors:** Cheng Zhang, Junjie Yu, Taoze Ji, Kai Zhao, Mingquan Chen

**Affiliations:** 1 Department of Urology, Northern Jiangsu People’s Hospital, Yangzhou, Jiangsu, China; 2 Department of Urology, Northern Jiangsu People’s Hospital Affiliated to Yangzhou University, Jiangsu, China; 3 Department of Urology, Jiangsu Province Hospital of Chinese Medicine, Affiliated Hospital of Nanjing University of Chinese Medicine, Nanjing, China; 4 Department of Urology, Guangdong Provincial People’s Hospital (Guangdong Academy of Medical Sciences), Southern Medical University, Guangzhou, China; 5 Guangdong Cardiovascular Institute, Guangdong Provincial People’s Hospital, Guangdong Academy of Medical Sciences, Guangzhou, China; University of Illinois at Chicago, UNITED STATES OF AMERICA

## Abstract

**Background.** Interleukin-1 receptor antagonist (IL1RA) blocks the interaction between IL-1 and its receptors. It modulates inflammatory responses, cell proliferation, and the invasion of cancer cells. In this study, we examined the biological functions of IL1RA and the mechanisms that influence its effects on prostate cancer (PCa).

**Materials and methods.** We performed RT-qPCR and Western blot analyses to evaluate IL1RA expression levels in various cell lines. For functional studies, we employed MTT, colony formation, soft agar, wound healing, and transwell migration and invasion assays. Then, we analyzed Western blots to elucidate the underlying mechanisms involved. Xenograft mouse models were ultimately established after the overexpression of IL1RA.

**Results.** IL1RA was expressed at higher levels in prostate epithelial cells compared with the PCa cell lines. BPH cells with lower IL1RA expression exhibited an increased cell proliferation. The PCa cell lines C4-2B and LNCap, which overexpressed IL1RA, demonstrated suppressed tumorigenic properties in vitro. The in vivo experiment demonstrated an inhibitory effect on tumor growth in xenograft mice. Furthermore, Western blot results indicated elevated phosphorylated AKT levels in BPH cells with IL1RA knockdown, and phosphorylated AKT and GSK-3β levels were reduced in C4-2B and LNCap cells that overexpressed IL1RA.

**Conclusion.** This study revealed that IL1RA low expression is associated with PCa progression. Our finding has great clinical and translational significance. The potential clinical application of IL1RA as a therapeutic target for PCa requires further investigation.

## Introduction

Prostate cancer (PCa) is one of the most common malignant tumors among men in the world, with its occurrence rate rising steadily, especially in China and other regions [1]. In Western countries, PCa is consistently ranked as the second most fatal cancer after lung cancer, and it stands as the most prevalent cancer type among adult males [2]. In 2016, approximately 180,890 men were diagnosed with PCa in America, which led to 26,120 fatalities attributed to the disease [3]. The early five-year survival rate is as high as 98%, but this percentage considerably declines to 30% for individuals diagnosed with metastatic diseases [4]. Surgical or pharmacological castration, also known as androgen-deprivation therapy, constitutes the standard treatment for PCa across all disease stages [5]. Although clinical management has advanced, key mechanisms underlying PCa progression are yet to be fully elucidated. One potentially involved molecule is the interleukin-1 receptor antagonist (IL1RA). In humans, the IL1RA gene encodes a protein called IL1RA, which naturally opposes IL-1α and IL-1β. IL1RA competes with IL-1α and IL-1β for interaction with IL-1R1, thereby exerting inhibitory control over inflammasome activation [6]. An Anakinra (Kineret) formulation containing human recombinant IL1RA is approved for the treatment of rheumatoid arthritis if administered subcutaneously once daily at a dose of 100 mg [7]. Iriondo et al. unveiled a correlation between elevated IL1RA expression levels and low aggressive breast cancer, with breast cancer patients with elevated IL1RA expression level exhibiting a better prognosis [8]. In bladder cancer, Schneider et al. demonstrated a negative correlation between IL1RA expression and the invasive and migratory capacities of tumor cells [9]. IL1RA can decrease metastatic capacity by blocking the IL-1α/vascular endothelial growth factor signaling pathway and may be used for the treatment of gastric cancer [10]. IL-1RA is downregulated in primary esophageal cancer tumors, and this downregulation of IL-1RA is closely related to TNM staging and survival prognosis. [11]. In colorectal cancer, IL1RA upregulation participates in cell invasion, migration, proliferation, colony formation, and epithelial–mesenchymal transition [12].

However, the role of IL1RA has not been investigated in PCa. This investigation centered on IL1RA-modulated signaling networks in prostate epithelial and carcinoma cells. We utilized in vitro methodologies, including cell transfection, real-time quantitative polymerase chain reaction (RT-qPCR), Western blot, and functional assays, for cell viability, migration, and invasion, respectively. Xenograft mouse models were also established. We systematically assessed IL1RA’s biological effect on PCa cells. The primary aim was to explore IL1RA’s regulation of proliferative and migratory capacities, with emphasis on its interaction with the AKT pathway.

We hypothesize that IL1RA acts as a tumor suppressor in the development of PCa, given its influence on critical signaling pathways. Investigating the mechanisms through which IL1RA mediates AKT may elucidate innovative therapeutic approaches aimed at this pathway. This research aimed to enhance our fundamental understanding of the role of IL1RA in the pathogenesis of PCa while also establishing a framework for the development IL1RA-centric clinical interventions.

## Materials and methods

### Cell culture

A normal human prostate epithelial cell line (BPH), normal human embryonic kidney cells (HEK 293T), and PCa cell lines (Arcap, PC3, C4-2B and LNCap) were purchased from Guangzhou Cellcook Biotech Co., Ltd. PC3, C4-2B, and LNCap cells were cultured in Roswell Park Memorial Institute (RPMI) 1640 medium (Gibco, USA), and Arcap and HEK-293T cells were maintained in Dulbecco’s Modified Eagle Medium (Gibco, USA). The culture media were supplemented with 10% fetal bovine serum (FBS, Gibco, USA) and 1% penicillin/streptomycin. Cellular incubation was carried out at 37 ^°^C in a 5% CO_2_ humidified environment. The cell lines were authenticated via short-tandem-repeat profiling.

### Cell transfection and infection

We carried out cell transfection and infection experiments as described by Trivedi et al. [13]. Sh scramble, shIL1RA, pBabe-puro empty vector, and IL1RA plasmids were obtained from Professor Cagatay Günes. The targeting sequences for IL1RA shRNA were 5’-GCCTTCAGAATCTGGGATGTT-3’ (shIL1RA-1#) and 5’-CGAGAACAGAAAGCAGGACAA -3’ (shIL1RA-2#). The target region was the coding sequence. Cells were seeded in 10 cm plates with 50% confluence, and they were infected by fresh virus the next day. The virus titer for infection was 1 x 10^8^ TU/ml. These infected cells were selected using puromycin at a concentration of 2 μg/ml for 5 days. Following their isolation, the infected cells were analyzed using RT-qPCR and Western blot techniques.

### RNA isolation and RT-qPCR

We performed RT-qPCR experiments as described by Bong et al. [14]. Total RNA was extracted following the manufacturer’s protocols using Trizol (Invitrogen, USA). Subsequently, the concentration and purity ratio of RNA were assessed using NanoDrop (Thermo Scientific, USA). RNA-to-cDNA kit (Applied Biosystems, USA) was used for RT-qPCR assay. Subsequent RT-qPCR experiments were performed using SYBR Green assays (Applied Biosystems, USA). The relative expression of IL1RA was assessed via the 2-*Δ*Ct method. The following primers were employed for qPCR analysis:

human IL1RA-forward: 5’- TGTTCCCATTCTTGCATGGC-3’,

human IL1RA-reverse: 5’- GCAGCATGGAGGCTGGTCAG-3’;

human glyceraldehyde-3-phosphate dehydrogenase (GAPDH)-forward: 5’-AAGGTCATCCCTGAGCTGAAC-3’,

human GAPDH-reverse: 5’-ACGCCTGCTTCACCACCTTCT-3’.

Applied Biosystems StepOne Plus Real-Time PCR System (Applied Biosystems, USA) was utilized to conduct reaction and result analysis. We repeated the RT-qPCR experiment three times independently.

### 3-[4,5-Dimethylthiazol-2-yl]-2,5 diphenyl tetrazolium bromide (MTT) assay

MTT assay was conducted as described by Choi et al. [15]. MTT assays were used to determine the viability of cells. 96-Well assay plates for C4-2B and LNCap cells were seeded with 5,000 cells/well (100 μl total volume per well). A density of 1,000 cells/well was used for the BPH cell line. We calculated cell viability for four days. For viability detection, each well of the 96-well plates received 20 μl MTT solution (5 mg/ml). The plates were then incubated in a CO_2_ incubator for 2 h. Afterward, 100 μl of dimethyl sulfoxide was added to induce cell lysis. Formazan absorbance values at 570 nm were assessed using a microplate reader. Each group had six replicates, and the MTT experiment was repeated thrice.

### Soft agar assay

We performed soft agar assay as described by Rotem et al. [16]. Soft agar assays were conducted in accordance with the following description. Briefly, an 8 ml top agar medium containing 0.3% agar solution was used to suspend cells (10 × 10^4^). Afterward, the cell suspensions were layered onto 10 cm dishes in triplicate with 8 ml bottom agar medium (the culture medium contained 0.5% agar). Fresh culture medium was added to the 10 cm plates every 3 days. The cells were photographed on days 1, 7, and 14 under a 5x objective microscope. Three independent repeated experiments were conducted.

### Colony formation assay

We conducted colony formation assay as described by Sun et al. [17]. This assay was used to assess the growth capacity of PCa cells. In brief, various numbers (300 cells/well for BPH; 1,500 cells/well for C4-2B and LNCap) of PCa cells were cultured in six-well plates for 7 days. Then, the cells were fixed and stained. Colonies containing more than 50 cells were counted after they were washed with water. Three replicates were prepared for each group. We performed three repeated experiments for colony formation assay.

### Wound healing assay

Wound healing assays were carried out as described by Hu et al. [18]. The assays were conducted to evaluate the effect of IL1RA on cell migration. A 12-well plate was seeded with 3x10^5^ cells per well. To create a wound, we scraped a 200 μl plastic pipette tip across the cultured cells at confluency. A series of images was obtained after the scratch and later at certain intervals. After the experiment, the gap in the confluent monolayer culture was evaluated to determine cell migration in a qualitative manner. Three independent experiments were conducted for wound-healing assay.

### Transwell migration and invasion assays

We performed transwell assay as described by Liu et al. [19]. Migration and invasion assays were performed using Boyden chambers (BD Biosciences, USA) equipped with 8 μm micropore membranes. The chambers were utilized without Matrigel for the migration assay and with Matrigel for the invasion assay. Following resuspension in RPMI 1640 serum-free medium, the cells were seeded into the upper chamber at a concentration of 2×10^4^ cells per 0.1 ml. Then, the cells were subjected to a 48 h incubation period within the chambers containing culture medium with 10% FBS. The cells were fixed, stained, and counted under a Zeiss microscope (Zeiss) with a 10x objective, with five random fields used for each chamber. This experiment was repeated thrice.

### Protein isolation and Western blot

Western blot experiment was conducted as described by Wang et al. [20]. The proteins were isolated and analyzed by Western blot. To extract cell lysates, we conducted a 48 h cell culture followed by cell lysis in radioimmunoprecipitation buffer (Sigma, USA). A total 40 μg protein was subjected to sodium dodecyl sulfate-polyacrylamide gel electrophoresis (Bio-Rad, USA) at 200 V for 45 min, followed by transfer onto a 0.45 mm pore-size polyvinylidene fluoride (polyvinylidene fluoride) membrane (Millipore) under a current of 300 mA for 2 h. The PVDF membranes were incubated with primary antibodies. The antibodies used, namely, rabbit anti-p-AKT Ser473 (#4060S) and anti-t-AKT (#75692S) (both at 1:1000; Cell Signaling Technology), were detected using anti-rabbit IgG. Similarly, mouse anti-α-tubulin (#T5168) (1:10000, Sigma), p-GSK-3β (#sc373800) and GSK-3β (#sc377213) (1:1000, Santa Cruz) were detected. To detect membranes, we used a chemiluminescence detection system (Quantity One; Bio-Rad, USA). We utilized ImageJ 1.53t (National Institutes of Health, Bethesda, MD, USA) to measure the intensity of signals observed on the Western blots. The Western blot experiment was repeated thrice.

### Subcutaneous xenograft tumor model

The protocol was performed in accordance with institutional ethics guidelines for animal experiments. A pathogen-free environment was maintained for BALB/c nude mice. An LNCap cell line (5 × 10^6^ cells/mouse) overexpressing IL1RA, or a negative control empty vector (EV), was subcutaneously injected into the mice. We measured the length and width of each tumor every five days with a caliper, and the tumor volume was calculated using the formula Volume = (Length × Width × Width)/2. The mice were euthanized after 20 days, and then the tumors were removed, weighed, photographed, and assessed.

### Statistical analysis

Statistical analysis was performed using GraphPad Prism 6.01 software. Student’s t-tests were conducted to compare two groups, and for comparisons involving three or more groups, one-way or two-way analysis of variance analyses were applied. All values are presented as mean ± standard error. Statistical significance was defined as follows: p < 0.05 (_*_), p < 0.01 (_**_), and p < 0.001 (_***_).

## Results

### IL1RA mRNA and protein expressions are lower in PCa cells

**Fig 1:**
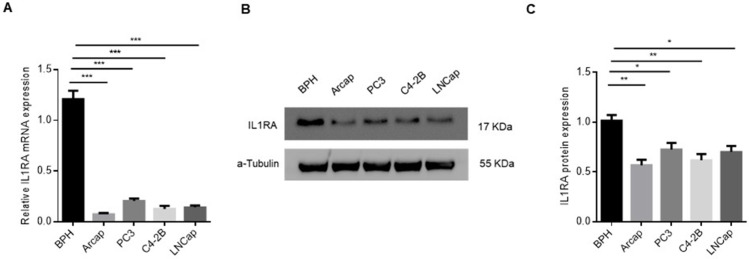
IL1RA is downregulated in prostate cancer. (A) The RT-qPCR assay demonstrated a significant downregulation of IL1RA mRNA levels in prostate cancer cell lines in comparison to the prostate epithelial cell line. (B) The Western blot experiment revealed a lower protein expression level of IL1RA in prostate cancer cell lines when contrasted with the prostate epithelial cell line. The full-length blots are presented in S1_File.pdf. The presented data are expressed as mean ± standard error of the mean (SEM), with each experiment being replicated three times. Statistical significance was denoted by *** p < 0.001.

Experiments were performed on prostate epithelial cells (BPH) and PCa cells (Arcap, PC3, C4-2B, and LNCap) to detect IL1RA expression. RT-qPCR analysis revealed the lower mRNA expression of IL1RA in PCa cells than in BPH cells (P < 0.001, Fig 1A). Western blots also showed IL1RA protein expression in these cell lines, which was consistent with mRNA levels (Fig 1B).

### Validation of knockdown or overexpression efficiency

**Fig 2:**
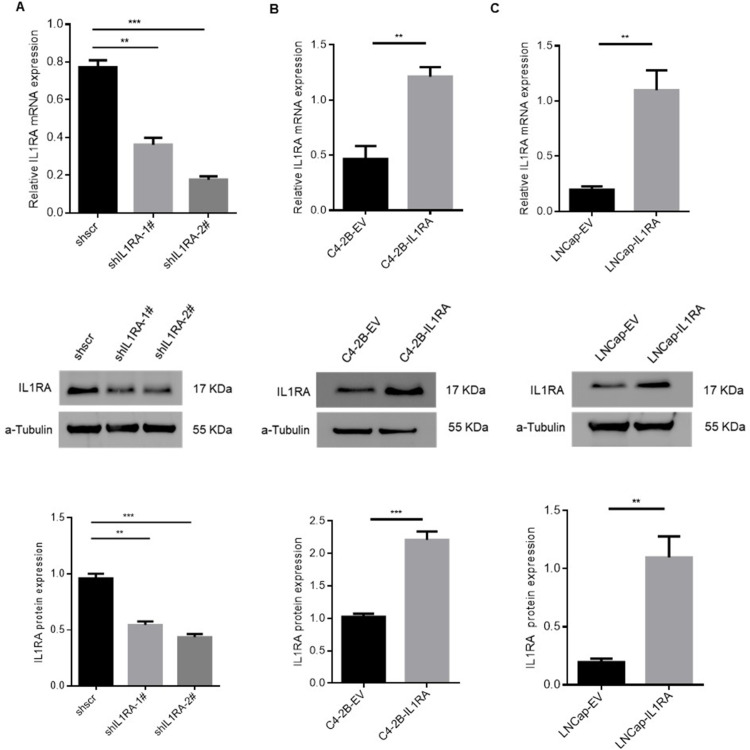
Assessment of IL1RA knockdown and overexpression efficacy in prostate cell lines. (A) The mRNA (P = 0.0001) and protein expression levels of IL1RA in the BPH knockdown cell line. (B, C) RT-qPCR and Western blot analyses were used to detect the mRNA (P = 0.0069 for C4-2B; P = 0.0080 for LNCap) and protein expression levels of IL1RA in PCa overexpression cells. The full-length blots are presented in S1 Fig. Each experiment was conducted three times. ** p < 0.01, *** p < 0.001.

RT-qPCR and Western blot analyses were conducted to confirm the establishment of stable cell lines that either silenced or overexpressed IL1RA at the mRNA and protein levels. In BPH knockdown cells, a comparison between shscramble and knockdown cell lines showed a notable reduction in IL1RA mRNA (P = 0.0001) and protein levels (Fig 2A). C4-2B (P = 0.0069) and LNCap (P = 0.0080) overexpression cell lines exhibited similar increases compared with the empty-vector cell lines (Fig 2B and 2C).

### IL1RA suppresses PCa proliferation

**Fig 3:**
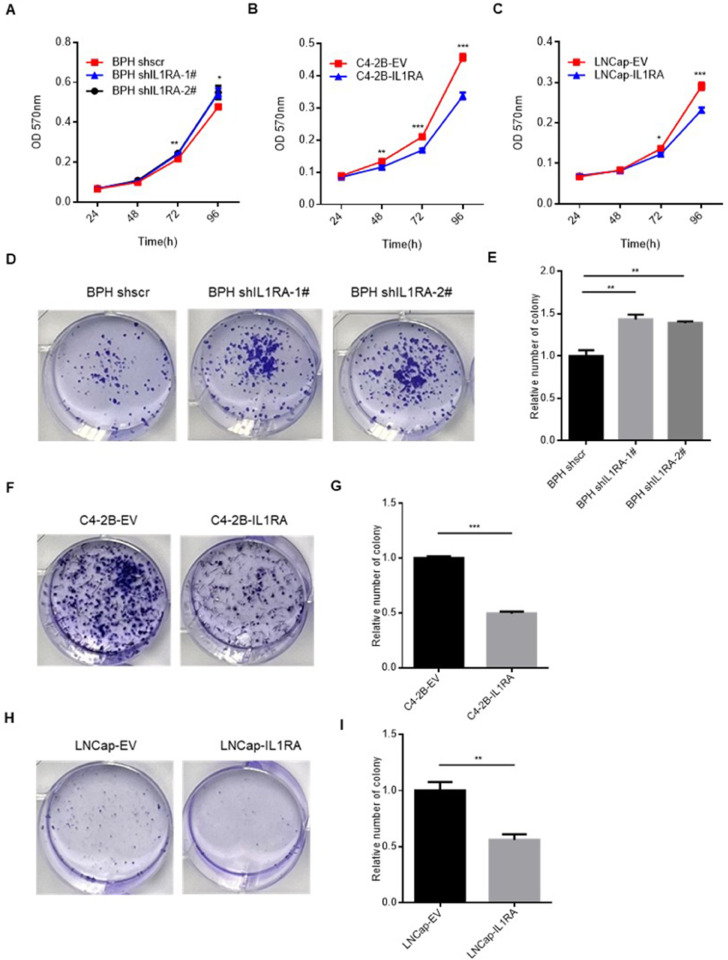
The suppressive impact of IL1RA on cell proliferation in prostate cancer. (A) MTT assay demonstrated that IL1RA knockdown enhanced BPH cell proliferation (P = 0.0254). (B, C) Overexpression of IL1RA in C4-2B and LNCap cells resulted in inhibited cell proliferation (P < 0.001 for C4-2B; P = 0.0004 for LNCap). (D, E) Colony formation assay indicated increased proliferation in shIL1RA cells compared to scrambled cells, as observed through colony counts (P = 0.0077). (F–I) Overexpressing IL1RA in C4-2B and LNCap cells led to a reduction in colony numbers (P < 0.001 for C4-2B; P = 0.0082 for LNCap). The data are presented as mean ± standard error of the mean (SEM). Each experiment was repeated three times. * p < 0.05, ** p < 0.01, *** p < 0.001.

MTT assays were conducted to determine whether IL1RA is involved in the regulation of cell proliferation in PCa and prostate epithelial cells. In comparison with the sh scramble cells, BPH knockdown cells showed significantly higher viability (P = 0.0254, Fig 3A). In addition, we observed a significant reduction in the proliferation of C4-2B (P < 0.001) and LNCap cells (P = 0.0004) compared with the empty-vector cells (Fig 3B and 3C).

Then, colony formation assay was employed to determine changes in cell viability. By knocking down or overexpressing IL1RA in prostate epithelial and PCa cells, we evaluated the colony forming potential. During IL1RA knockdown, the BPH cells exhibited a notable rise in colony numbers compared with the control scrambled cells (P = 0.0077, Fig 3D and 3E). In addition, IL1RA overexpression reduced the colony-forming ability of C4-2B (P < 0.001) and LNCap (P = 0.0082) cells (Fig 3F–3I).

**Fig 4:**
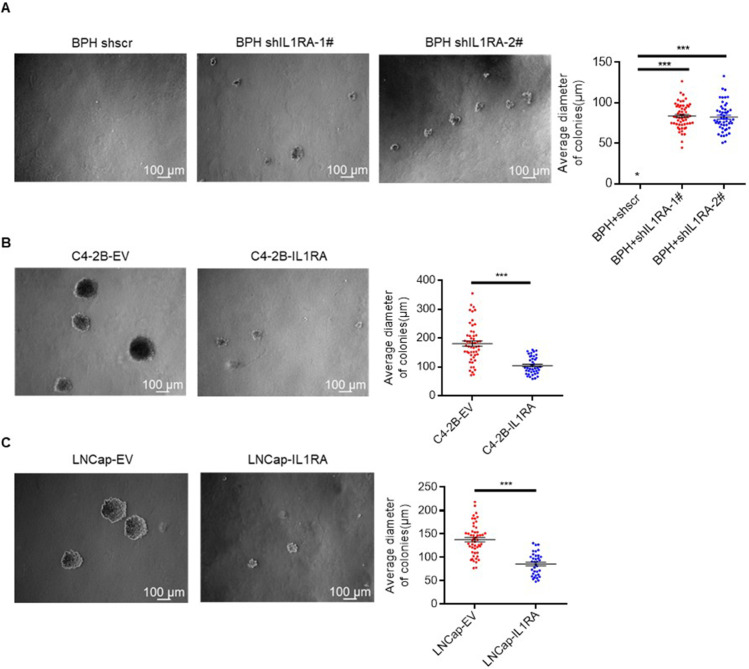
The inhibitory effect of IL1RA on cell growth in prostate cancer. (A) Soft agar assay demonstrated a substantial enhancement in anchorage-independent growth of BPH cells following IL1RA knockdown (P < 0.001). (B, C) IL1RA overexpression notably hindered the anchorage-independent growth of prostate cancer cells (P < 0.001 for C4-2B; P < 0.001 for LNCap). Images were captured at 5x objective under a microscope. Each experiment was replicated three times. *** p < 0.001.

Soft agar was used to determine whether human prostate cells can grow without anchorage. BPH, C4-2B, and LNCap cells grown on soft agar were tested for the effect of IL1RA on colony formation. The result demonstrated a substantial enhancement in the anchorage-independent growth of prostate epithelial cells upon knockdown of IL1RA (P < 0.001, Fig 4A). A further test using soft agar assays showed that IL1RA overexpression significantly impaired the anchorage-independent growth of PCa cells (P < 0.001, Fig 4B and 4C).

### IL1RA impairs the migratory and invasive capacities of PCa cells

**Fig 5:**
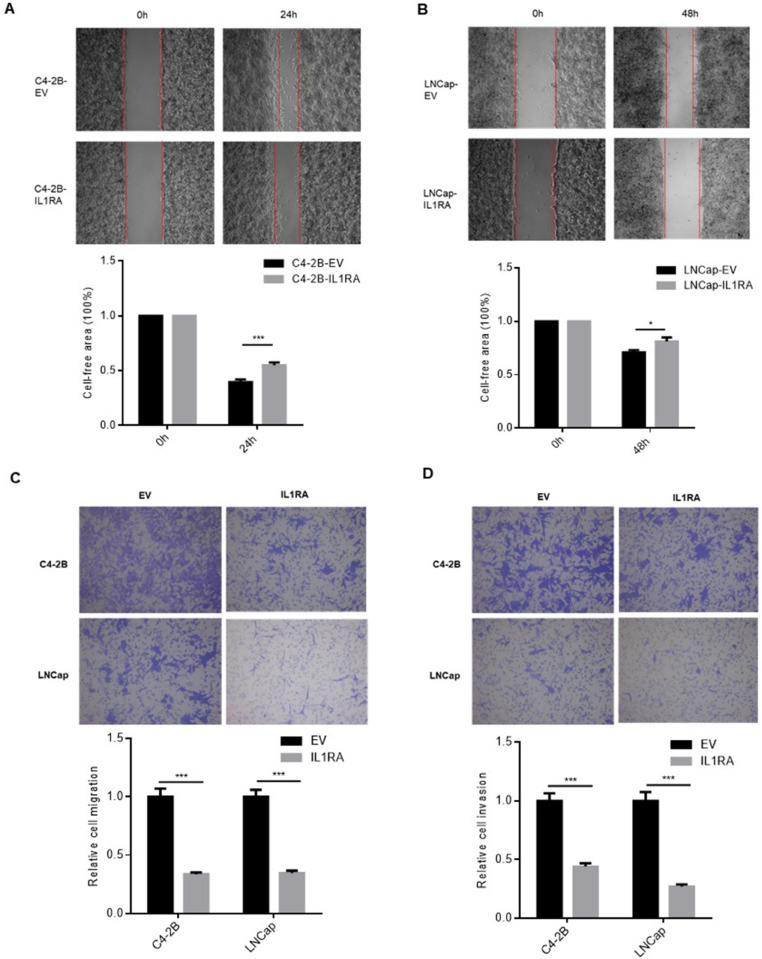
The inhibitory influence of IL1RA on the cell migration of prostate cancer cells. (A, B) Wound healing assay indicated that cells overexpressing IL1RA exhibited a decreased migration speed compared to cells with the empty vector (P = 0.0008 for C4-2B; P = 0.0395 for LNCap). Images were captured at a 5x objective. (C) Boyden chamber assay demonstrated a reduction in the migrative ability of C4-2B (P < 0.001) and LNCap (P < 0.001) cells following the overexpression of IL1RA in these cell lines. Pictures were taken at a 10x objective. (D) Boyden chamber assay revealed a decrease in the invasive capacity of C4-2B-IL1RA (P < 0.001) and LNCap-IL1RA (P < 0.001) cells. Images were obtained at a 10x objective. The data are presented as mean ± standard error of the mean (SEM), with each experiment repeated three times. * p < 0.05, *** p < 0.001.

Wound healing assays and transwell migration/invasion assays were conducted to detect cell migration and invasion abilities, respectively. For wound healing assay, different time points were selected based on our preliminary experiments, which demonstrated the optimal wound closure rate for each cell line. Based on wound healing assay, the IL1RA overexpression group had a slower wound closure rate (P = 0.0008 for C4-2B, P = 0.0395 for LNCap, Fig 5A and 5B), which suggests that IL1RA may decrease PCa cell migration. In addition, transwell migration/invasion assays revealed that IL1RA overexpression decreased the number of cells migrating and invading through the Boyden chamber in comparison with the empty-vector group (P < 0.001, Fig 5C and 5D). According to the above data, IL1RA may significantly diminish the migratory and invasive abilities of PCa cells.

### IL1RA is involved in the regulation of AKT signaling pathway

**Fig 6:**
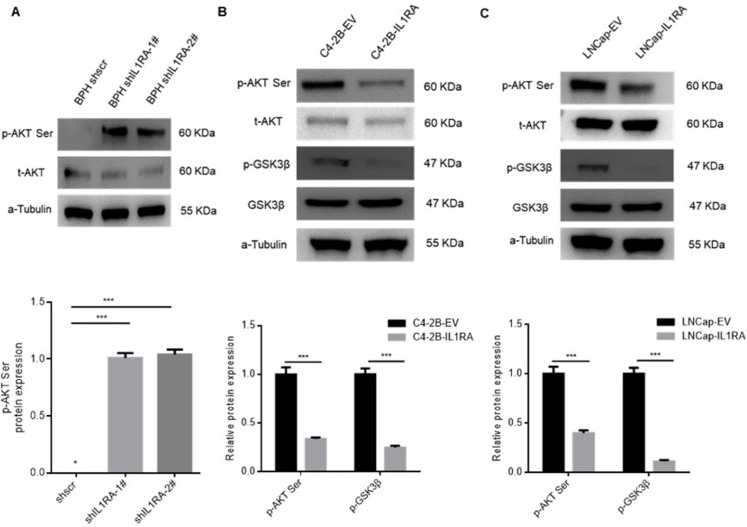
The involvement of IL1RA in modulating AKT pathway activation in BPH, C4-2B and LNCap cells. (A) Evaluation of phospho-AKT (Ser473) and total-AKT protein expression levels in BPH cells with shscramble and shIL1RA. (B) Analysis of phospho-AKT (Ser473) and total-AKT protein expression levels in C4-2B cells with empty vector and IL1RA overexpression. (C) Examination of phospho-AKT (Ser473) and total-AKT protein expression levels in LNCap cells with empty vector and ectopic IL1RA. The full-length blots are presented in S1 File.pdf. α-Tubulin was used as the loading control. Three independent repeated experiments were conducted.

We carried out Western blot analyses to examine whether IL1RA regulates the AKT pathway (Fig 6). The increased phosphorylation of AKT upon IL1RA knockdown is a crucial mechanistic insight. Activated Akt can phosphorylate many downstream substrates, including GSK3β, Foxo-family transcription factors and so on. These proteins collectively provide powerful signals for cell growth, and survival [21]. In BPH cells, IL1RA knockdown resulted in an increase in p-AKT Ser473 protein expression according to these analyses (Fig 6A). C4-2B and LNCap cells overexpressing IL1RA also showed a reduction in p-AKT Ser473 and GSK-3β protein expression (Fig 6B and 6C). Accordingly, IL1RA regulates the AKT signaling pathway based on the present data.

### IL1RA suppresses PCa cell oncogenesis in vivo

**Fig 7:**
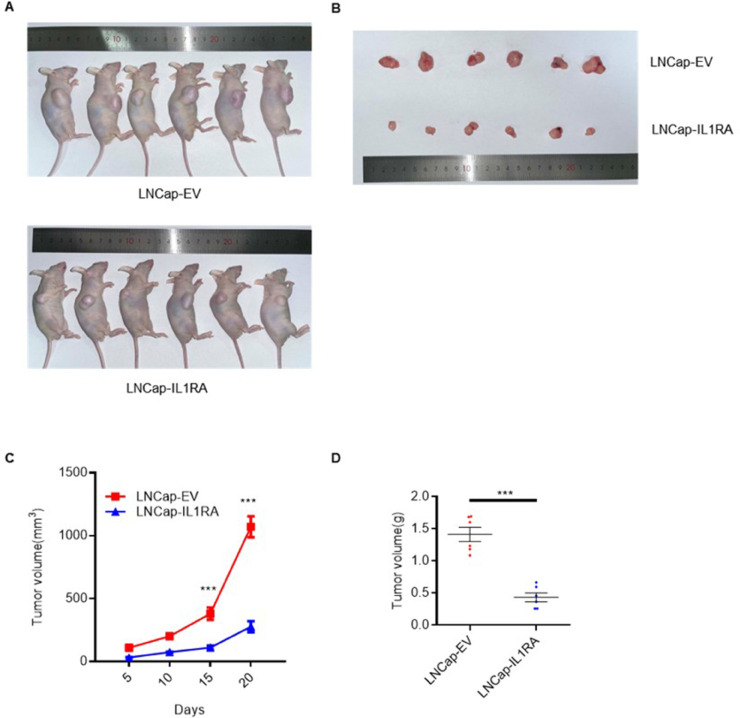
IL1RA suppresses LNCap cell oncogenesis in vivo. (A-B) Representative images of tumors formed in nude mice injected subcutaneously with LNCap-EV and LNCap-IL1RA cells (N = 6). (C) Tumor growth curves for the indicated groups. (D) Tumor weight assessment after dissection. *** p < 0.001.

To examine the function of IL1RA in PCa progression in vivo, we generated xenograft mice using LNCap cells overexpressing IL1RA. The diameters of the tumors were measured every five days, and tumor growth curves were drawn. Compared with the control group, the group with overexpressed IL1RA showed reduced tumor growth (Fig 7A and 7B). Tumors implanted with LNCap cells expressing stable IL1RA displayed considerable decreases in volume and weight 20 days after inoculation (Fig 7C and 7D). In vivo, IL1RA overexpression inhibited tumor growth and progression, demonstrating its potential role as a tumor suppressor.

## Discussion

According to previous studies, IL1RA inhibits cancer cell growth, migration, and invasion, which is a key factor in cancer progression and metastasis. In oral squamous-cell carcinoma, Ding et al. showed that IL1RA-mediated type I interferon induction curbed malignant progression [22]. An additional investigation suggested an association between IL1RA and reduced cervical cancer risk [23]. In lung cancer, Yigit et al. revealed a significant association between IL1RA levels and progression and survival outcomes [24]. Vanarsa et al. discovered that IL1RA can be used to distinguish between urine samples collected from the healthy group and urine from different stages of bladder cancer and can therefore be used as a tool for staging bladder cancer [25].

We conducted a comprehensive literature search and found no relevant reports on IL1RA and PCa. This study is the first to delve on the role of IL1RA in PCa. The results of RT-qPCR and Western blot indicate that the mRNA and protein expressions of IL1RA were higher in immortalized BPH cells, whereas its expression was notably downregulated in PCa cell lines. The outcomes of this investigation align with similar observations made on some cancer types, including bladder [9], esophageal [11], and gastric cancer [10].

IL1RA can impede the growth and proliferation of cancer cells in esophageal cancer [11], pancreatic cancer [26], and acute myeloid leukemia [27]. Our results suggest that IL1RA negatively affects PCa cell growth. As demonstrated by MTT assays, IL1RA suppresses PCa and prostate epithelial cell viability. Moreover, the results of colony formation assay show that IL1RA negatively influences colony-forming ability. In addition, soft agar assays were conducted to determine the anchorage-independent growth capacity of cells [28]. Our study demonstrated that IL1RA can suppress this growth of PCa and prostate epithelial cells. Xenograft mouse models also presented the inhibitory effect on tumor growth. These findings are consistent with those of prior published studies. Collectively, IL1RA likely plays a crucial role in the proliferation and growth of PCa cells.

Cancer cells can invade and migrate, which make them targets for cancer treatment [29]. IL1RA can suppress invasion and migration in various cancer types, including bladder [9] and colorectal cancer [12]. Our findings show that IL1RA can inhibit PCa cell migration through wound healing assay. According to migration/invasion assays, IL1RA also negatively influences PCa migration and invasion. All of these functional studies indicated that IL1RA upregulation inhibits the migration and invasion of PCa cells. These results align with those of previous studies, which indicates consistency. Thus, IL1RA may function as a tumor suppressor and regulate PCa migration and invasion.

The AKT pathway is a celebrity pathway. The core role of this pathway in cancer is to regulate the key pathway of cell proliferation; in addition, it shows a direct relation to tumor occurrence and development [30]. Shimizu et al. showed that elevated levels of phosphorylated AKT expression were correlated with an increased tumor grade in PCa [31]. Tumors, especially epithelial cancers, such as prostate adenocarcinoma, have the most deregulated AKT pathways [32]. Many studies have underlined the role of AKT in cell growth, proliferation, survival, and migration due to its involvement in vital oncogenic pathways [33–35]. Wang et al. highlighted that AKT signaling pathway is implicated in carcinogenesis and inhibits the growth of pancreatic cancer cells [36]. AKT stimulates proliferation and invasion in aggressive thyroid cancers by upregulating their activity [37]. Based on our research results, IL1RA knockdown activated the AKT pathway in BPH cells, and IL1RA overexpression decreased AKT pathway activation in C4-2B and LNCap cells. Based the above findings, we can conclude that IL1RA potentially influences the development and progression of PCa via the AKT signaling pathway.

However, this study still has its limitations. First, further research is warranted to elucidate the intricate mechanisms by which IL1RA contributes to the progression of PCa. Second, the lack of patient-derived samples, heterogeneity of PCa, or resistance mechanisms influence the treatment outcomes of IL1RA and thereby affect its translational value. Third, the translational applicability of IL1RA as a therapeutic target requires additional examination. We can conduct preclinical studies using animal models and clinical trials involving the therapeutic effects of IL1RA on PCa patients.

## Conclusion

Our findings reveal a significant increase in mRNA and protein expression levels of IL1RA in noncancerous cells when contrasted with PCa cells. This study marks the first identification of IL1RA as a key regulator influencing the proliferation and growth of prostate epithelial cells. Functional assays confirmed that IL1RA suppresses the proliferation, growth, migration, and invasion of PCa cells. The inhibitory effect of IL1RA on tumor growth was also observed in xenograft mice during in vivo experiments.

## Abbreviations

IL1RA: Interleukin-1 receptor antagonist

PCa: Prostate cancer

EMT: Epithelial-mesenchymal transition

RT-qPCR: Real-time quantitative polymerase chain reaction

RIPA: Radioimmunoprecipitation

## Supporting information

S1 File The full-length of Western blots. The full-length blots are presented in S1 File.pdf.
(PDF)

S2 File The Zip file of raw data. The raw data are presented in Raw data.zip.
(ZIP)

## References

[1] Liu J, Dong L, Zhu Y, Dong B, Sha J, Zhu HH, et al. Prostate cancer treatment - China’s perspective. Cancer Lett. 2022;550:215927. doi: 10.1016/j.canlet.2022.215927 36162714

[2] Cui D, Dai J, Keller JM, Mizokami A, Xia S, Keller ET. Notch Pathway Inhibition Using PF-03084014, a *γ*-Secretase Inhibitor (GSI), Enhances the Antitumor Effect of Docetaxel in Prostate Cancer. Clin Cancer Res. 2015;21(20):4619–29. doi: 10.1158/1078-0432.CCR-15-0242 26202948 PMC4609279

[3] Kinlock BL, Thorpe RJ Jr, Howard DL, Bowie JV, Ross LE, Fakunle DO, et al. Racial disparity in time between first diagnosis and initial treatment of prostate cancer. Cancer Control. 2016;23(1):47–51. doi: 10.1177/107327481602300108 27009456 PMC6448564

[4] Desai K, McManus JM, Sharifi N. Hormonal therapy for prostate cancer. Endocrine Reviews. 2021;42(3):354–73. doi: 10.1210/endrev/bnab002 33480983 PMC8152444

[5] Gamat M, McNeel DG. Androgen deprivation and immunotherapy for the treatment of prostate cancer. Endocr Relat Cancer. 2017;24(12):T297–310. doi: 10.1530/ERC-17-0145 28814451 PMC5669826

[6] Meyer NJ, Ferguson JF, Feng R, Wang F, Patel PN, Li M, et al. A functional synonymous coding variant in the IL1RN gene is associated with survival in septic shock. Am J Respir Crit Care Med. 2014;190(6):656–64. doi: 10.1164/rccm.201403-0586OC 25089931 PMC4214110

[7] Helmy A, Guilfoyle MR, Carpenter KLH, Pickard JD, Menon DK, Hutchinson PJ. Recombinant human interleukin-1 receptor antagonist in severe traumatic brain injury: a phase II randomized control trial. J Cereb Blood Flow Metab. 2014;34(5):845–51. doi: 10.1038/jcbfm.2014.23 24569690 PMC4013762

[8] Iriondo O, Liu Y, Lee G, Elhodaky M, Jimenez C, Li L, et al. TAK1 mediates microenvironment-triggered autocrine signals and promotes triple-negative breast cancer lung metastasis. Nat Commun. 2018;9(1):1994. doi: 10.1038/s41467-018-04460-w 29777109 PMC5959931

[9] Schneider L, Liu J, Zhang C, Azoitei A, Meessen S, Zheng X, et al. The role of Interleukin-1-receptor-antagonist in bladder cancer cell migration and invasion. Int J Mol Sci. 2021;22(11):5875. doi: 10.3390/ijms22115875 34070905 PMC8198563

[10] Gong Z, Ma J, Su H, Guo T, Cai H, Chen Q, et al. Interleukin-1 receptor antagonist inhibits angiogenesis in gastric cancer. Int J Clin Oncol. 2018;23(4):659–70. doi: 10.1007/s10147-018-1242-2 29344744 PMC6097079

[11] Chen S, Shen Z, Liu Z, Gao L, Han Z, Yu S, et al. IL-1RA suppresses esophageal cancer cell growth by blocking IL-1*α*. J Clin Lab Anal. 2019;33(6):e22903. doi: 10.1002/jcla.22903 31102307 PMC6642324

[12] Chen Y, Yang Z, Deng B, Wu D, Quan Y, Min Z. Interleukin 1*β*/1RA axis in colorectal cancer regulates tumor invasion, proliferation and apoptosis via autophagy. Oncol Rep. 2020;43(3):908–18. doi: 10.3892/or.2020.7475 32020215 PMC7041122

[13] Trivedi PD, Yu C, Chaudhuri P, Johnson EJ, Caton T, Adamson L, et al. Comparison of highly pure rAAV9 vector stocks produced in suspension by PEI transfection or HSV infection reveals striking quantitative and qualitative differences. Molecular Therapy - Methods & Clinical Development. 2022;24:154–70. doi: 10.1016/j.omtm.2021.12.006 35071688 PMC8760416

[14] Bong D, Sohn J, Lee S-JV. Brief guide to RT-qPCR. Mol Cells. 2024;47(12):100141. doi: 10.1016/j.mocell.2024.100141 39476972 PMC11612376

[15] Choi BH, Kim M-R, Jung YN, Kang S, Hong J. Interfering with color response by porphyrin-related compounds in the MTT tetrazolium-based colorimetric assay. Int J Mol Sci. 2022;24(1):562. doi: 10.3390/ijms24010562 36614004 PMC9820508

[16] Rotem A, Janzer A, Izar B, Ji Z, Doench JG, Garraway LA, et al. Alternative to the soft-agar assay that permits high-throughput drug and genetic screens for cellular transformation. Proc Natl Acad Sci U S A. 2015;112(18):5708–13. doi: 10.1073/pnas.1505979112 25902495 PMC4426412

[17] Sun Y, Zhang S, Zhang X, Li G, Sun F, Wang M, et al. AURKA enhances the glycolysis and development of ovarian endometriosis through ER*β*. Endocrinology. 2024;165(4):bqae018. doi: 10.1210/endocr/bqae018 38340326

[18] Hu Y, Rao S-S, Wang Z-X, Cao J, Tan Y-J, Luo J, et al. Exosomes from human umbilical cord blood accelerate cutaneous wound healing through miR-21-3p-mediated promotion of angiogenesis and fibroblast function. Theranostics. 2018;8(1):169–84. doi: 10.7150/thno.21234 29290800 PMC5743467

[19] Liu Z, Wang Y, Dou C, Xu M, Sun L, Wang L, et al. Hypoxia-induced up-regulation of VASP promotes invasiveness and metastasis of hepatocellular carcinoma. Theranostics. 2018;8(17):4649–63. doi: 10.7150/thno.26789 30279729 PMC6160773

[20] Wang W, Zheng X, Azoitei A, John A, Zengerling F, Wezel F, et al. The role of TKS5 in chromosome stability and bladder cancer progression. Int J Mol Sci. 2022;23(22):14283. doi: 10.3390/ijms232214283 36430759 PMC9698602

[21] Vincent EE, Elder DJE, Thomas EC, Phillips L, Morgan C, Pawade J, et al. Akt phosphorylation on Thr308 but not on Ser473 correlates with Akt protein kinase activity in human non-small cell lung cancer. Br J Cancer. 2011;104(11):1755–61. doi: 10.1038/bjc.2011.132 21505451 PMC3111153

[22] Ding Y, Shan Y, Gu J, Yi J, Sun Z. IL1RA inhibits the progression of oral squamous cell carcinoma by mediating type I interferon response. Transl Oncol. 2025;58:102428. doi: 10.1016/j.tranon.2025.102428 40441062 PMC12159535

[23] Tamandani DMK, Sobti RC, Shekari M, Kaur S, Huria A. Impact of polymorphism in IL-1RA gene on the risk of cervical cancer. Arch Gynecol Obstet. 2008;277(6):527–33. doi: 10.1007/s00404-007-0504-4 18008080

[24] Yigit M, Değirmencioğlu S, Ugurlu E, Yaren A. Effect of serum interleukin-1 receptor antagonist level on survival of patients with non-small cell lung cancer. Mol Clin Oncol. 2017;6(5):708–12. doi: 10.3892/mco.2017.1195 28515924 PMC5431311

[25] Vanarsa K, Enan S, Patel P, Strachan B, Sam Titus ASCL, Dennis A, et al. Urine protein biomarkers of bladder cancer arising from 16-plex antibody-based screens. Oncotarget. 2021;12(8):783–90. doi: 10.18632/oncotarget.27941 33889301 PMC8057279

[26] Morgan AG, Griffin MF, Longaker MT, Norton JA. Precision medicine: IL-1RA and pancreatic cancer organoids. Biology (Basel). 2025;14(6):604. doi: 10.3390/biology14060604 40563856 PMC12189712

[27] Grauers Wiktorin H, Aydin E, Christenson K, Issdisai N, Thorén FB, Hellstrand K, et al. Impact of IL-1*β* and the IL-1R antagonist on relapse risk and survival in AML patients undergoing immunotherapy for remission maintenance. Oncoimmunology. 2021;10(1):1944538. doi: 10.1080/2162402X.2021.1944538 34367728 PMC8317920

[28] Du F, Zhao X, Fan D. Soft agar colony formation assay as a hallmark of carcinogenesis. Bio Protoc. 2017;7(12):e2351. doi: 10.21769/BioProtoc.2351 34541100 PMC8410321

[29] Aust G, Zheng L, Quaas M. To detach, migrate, adhere, and metastasize: CD97/ADGRE5 in cancer. Cells. 2022;11(9):1538. doi: 10.3390/cells11091538 35563846 PMC9101421

[30] Chen H, Zhou L, Wu X, Li R, Wen J, Sha J, et al. The PI3K/AKT pathway in the pathogenesis of prostate cancer. Front Biosci (Landmark Ed). 2016;21(5):1084–91. doi: 10.2741/4443 27100493

[31] Shimizu Y, Segawa T, Inoue T, Shiraishi T, Yoshida T, Toda Y, et al. Increased Akt and phosphorylated Akt expression are associated with malignant biological features of prostate cancer in Japanese men. BJU Int. 2007;100(3):685–90. doi: 10.1111/j.1464-410X.2007.07014.x 17542985

[32] Rambur A, Lours-Calet C, Beaudoin C, Buñay J, Vialat M, Mirouse V, et al. Sequential Ras/MAPK and PI3K/AKT/mTOR pathways recruitment drives basal extrusion in the prostate-like gland of Drosophila. Nat Commun. 2020;11(1):2300. doi: 10.1038/s41467-020-16123-w 32385236 PMC7210301

[33] Ou Y, Ma L, Ma L, Huang Z, Zhou W, Zhao C, et al. Overexpression of cyclin B1 antagonizes chemotherapeutic-induced apoptosis through PTEN/Akt pathway in human esophageal squamous cell carcinoma cells. Cancer Biol Ther. 2013;14(1):45–55. doi: 10.4161/cbt.22627 23114644 PMC3566051

[34] Tang G, Tang Q, Jia L, Chen Y, Lin L, Kuai X, et al. TROP2 increases growth and metastasis of human oral squamous cell carcinoma through activation of the PI3K/Akt signaling pathway. Int J Mol Med. 2019;44(6):2161–70. doi: 10.3892/ijmm.2019.4378 31638186 PMC6844621

[35] Shin S-H, Lee GY, Lee M, Kang J, Shin H-W, Chun Y-S, et al. Aberrant expression of CITED2 promotes prostate cancer metastasis by activating the nucleolin-AKT pathway. Nat Commun. 2018;9(1):4113. doi: 10.1038/s41467-018-06606-2 30291252 PMC6173745

[36] Wang Y, Ren F, Song Z, Wang X, Ma X. Multiomics profile and prognostic gene signature of m6A regulators in uterine corpus endometrial carcinoma. J Cancer. 2020;11(21):6390–401. doi: 10.7150/jca.46386 33033522 PMC7532517

[37] Wu D, Li J, Zhang Q, Tian W, Zhong P, Liu Z, et al. Exogenous hydrogen sulfide regulates the growth of human thyroid carcinoma cells. Oxid Med Cell Longev. 2019;2019:6927298. doi: 10.1155/2019/6927298 31223424 PMC6541980

