# Supplementary figures and images for: Loss of IL1RA promotes prostate cancer growth and metastasis by activating Akt signaling pathway (part 1 of 2)
Source: PLoS One. 2026 Feb 2;21(2):e0339611. doi: 10.1371/journal.pone.0339611 (PMC12863537; doi:10.1371/journal.pone.0339611)

Figure 1B

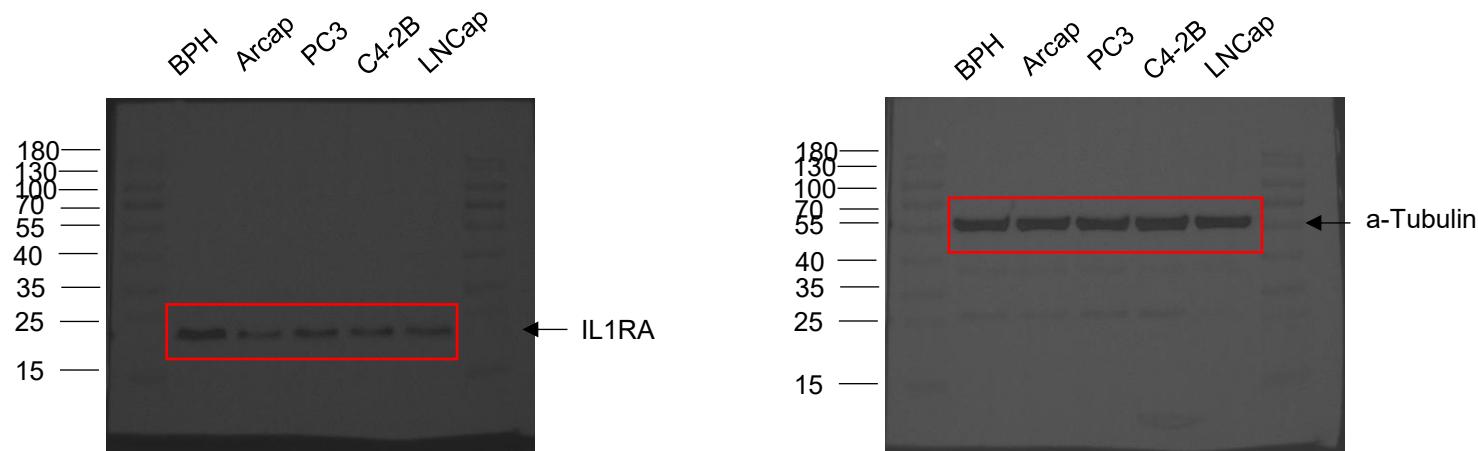

Figure 2A

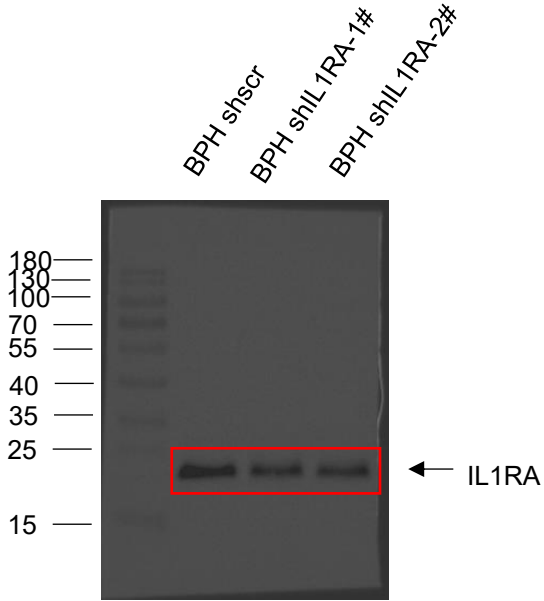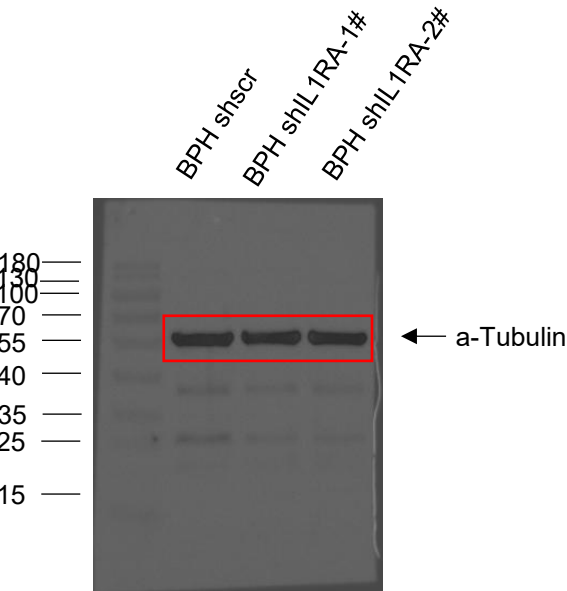

Figure 2B

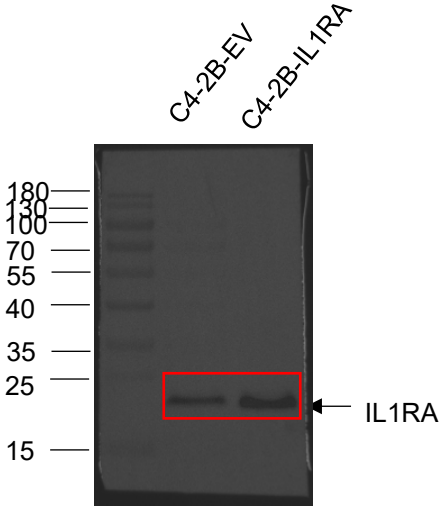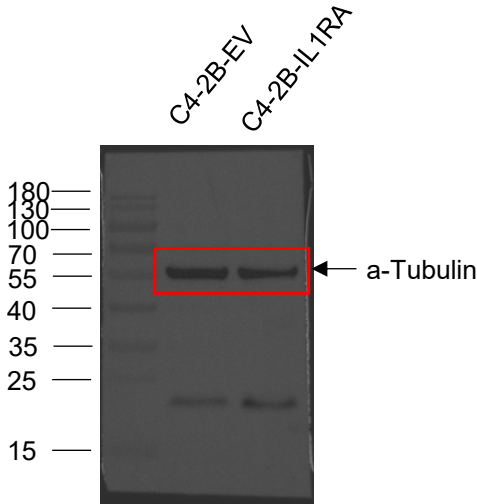

Figure 2C

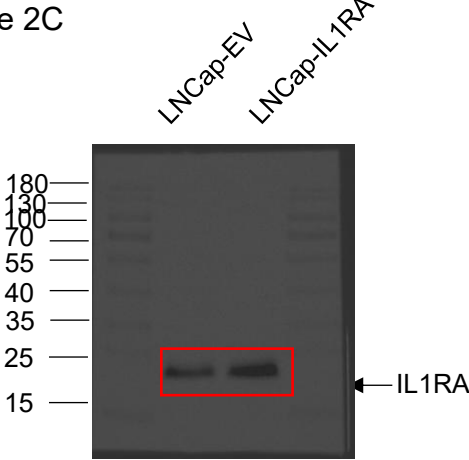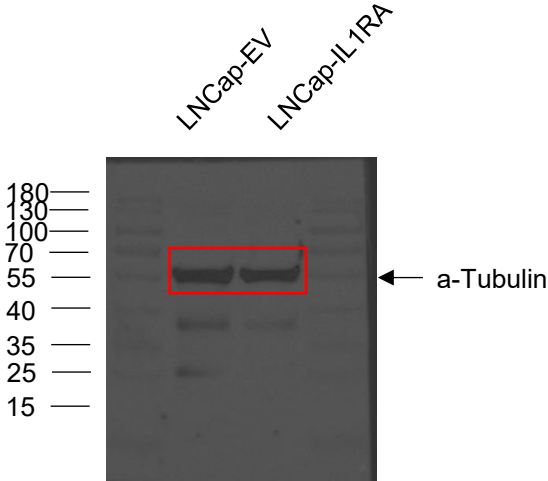

Figure 6A

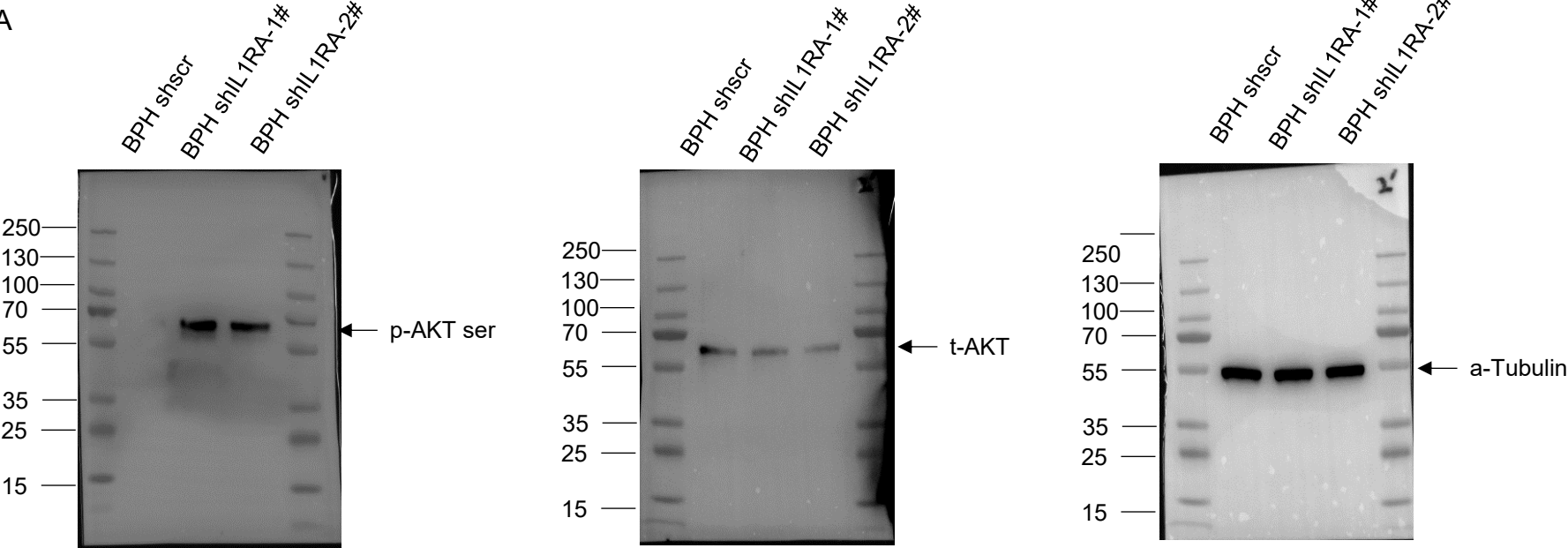

Figure 6B

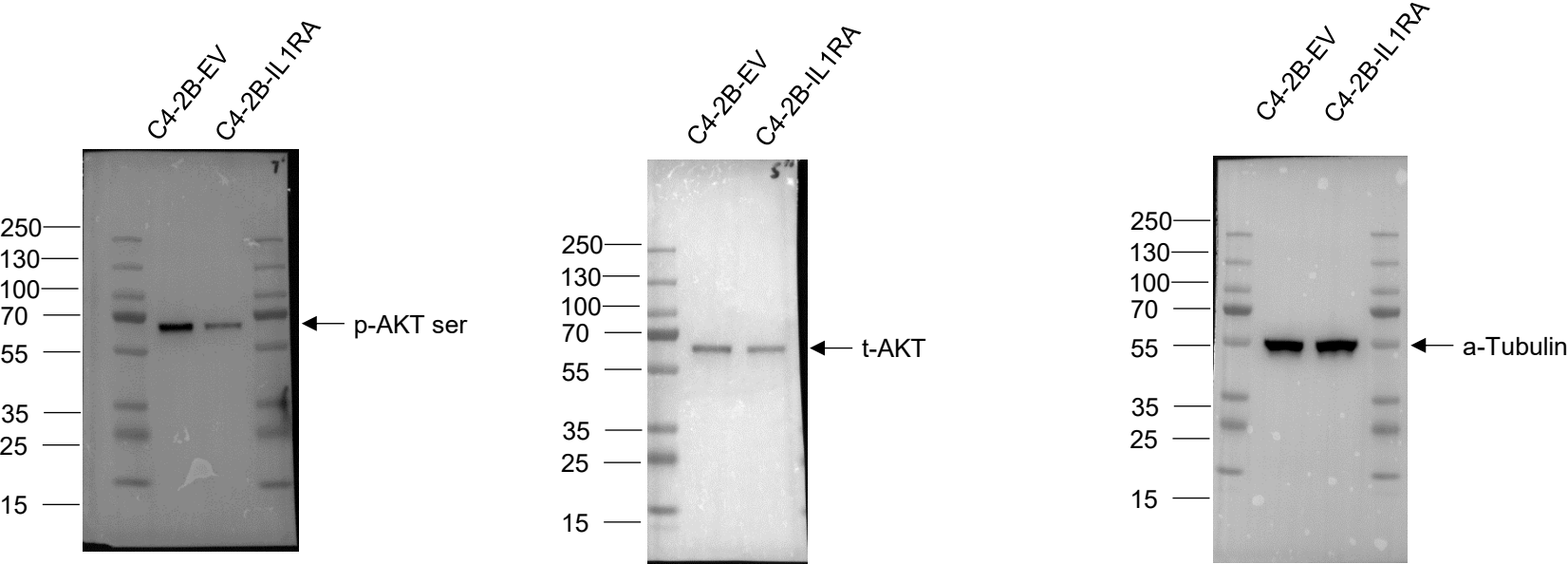

Figure 6C

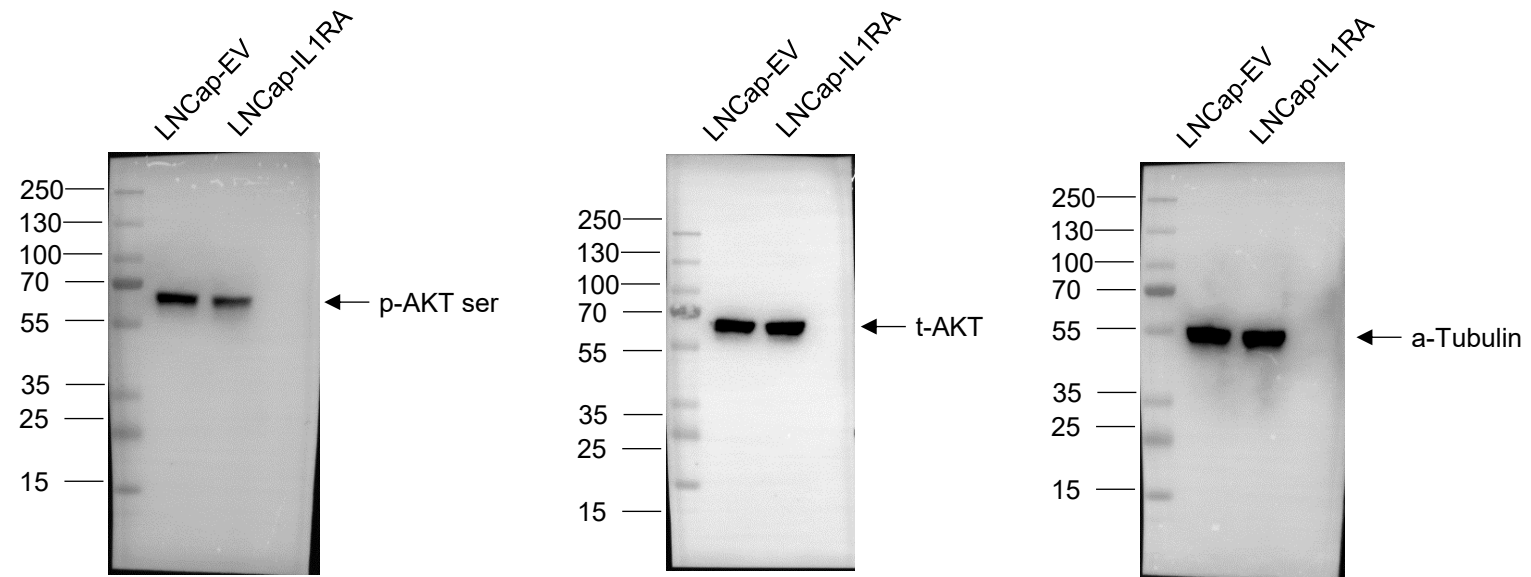

Figure 6

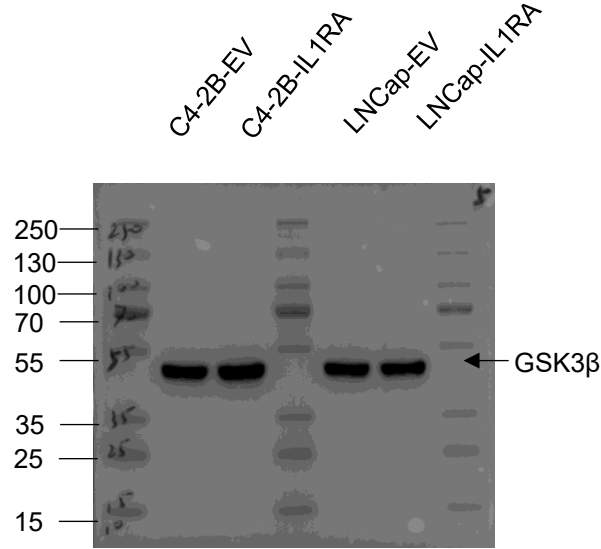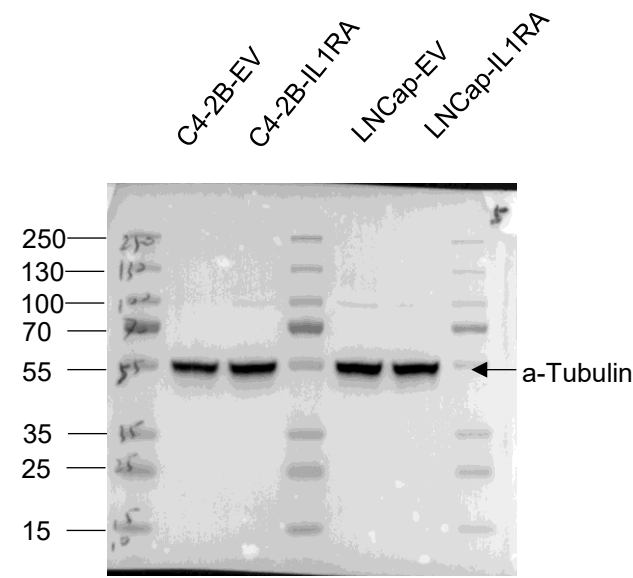

Figure 6

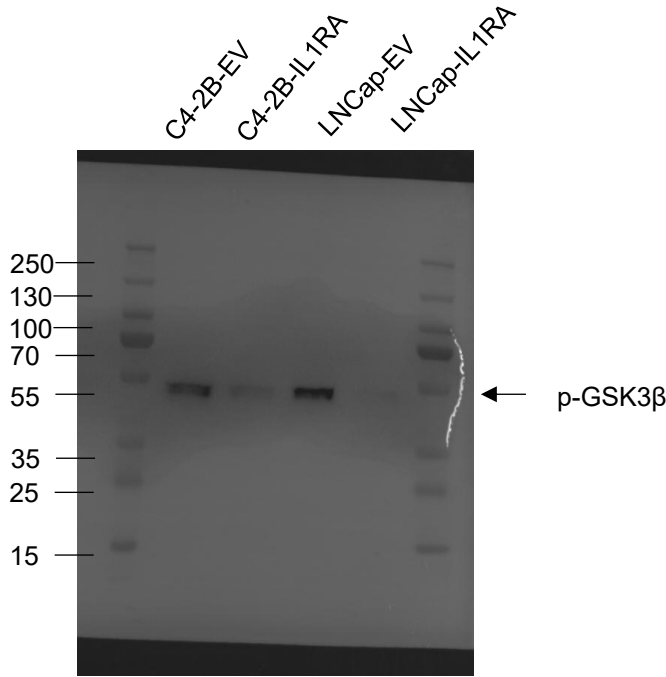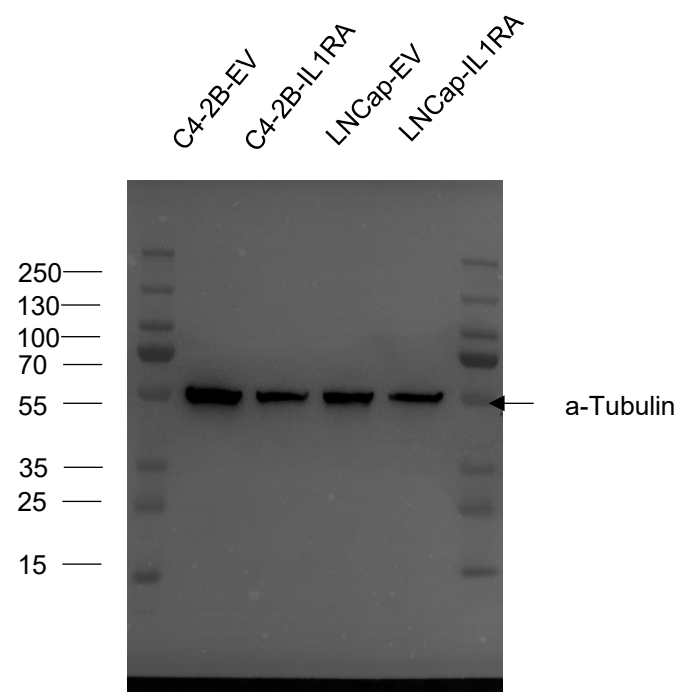

Supplement: S1 File — The full-length blots are presented in S1 File.pdf. (PDF) [file pone.0339611.s001.pdf]

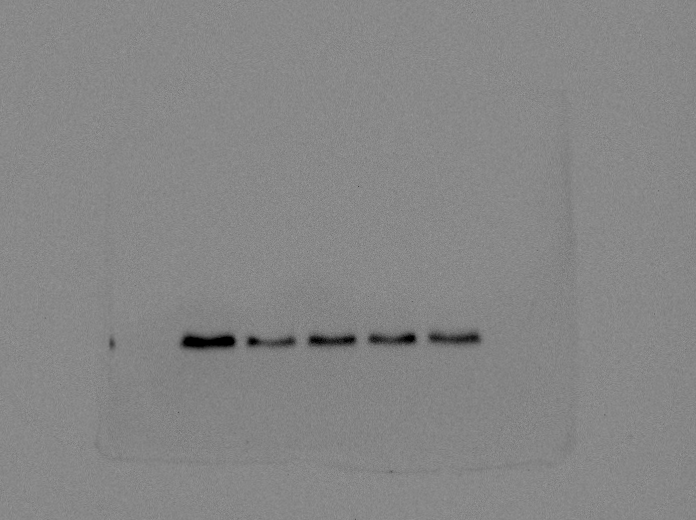

Supplement: S2 File — The raw data are presented in Raw data.zip. (ZIP) [file pone.0339611.s002.zip › Raw data/Figure 1/WB-IL1RA cell line/target/cell line yy 2.tif]

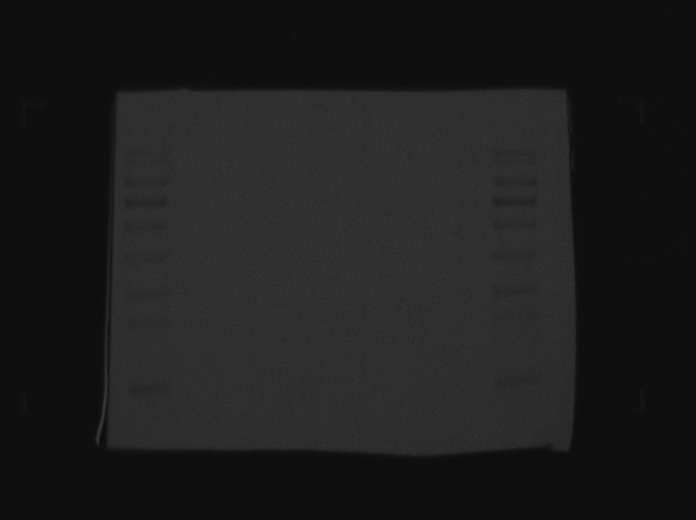

Supplement: S2 File — The raw data are presented in Raw data.zip. (ZIP) [file pone.0339611.s002.zip › Raw data/Figure 1/WB-IL1RA cell line/target/cell line'.tif]

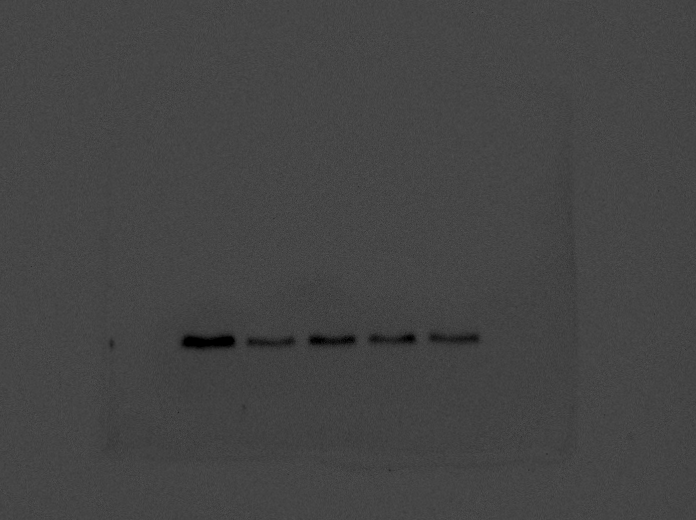

Supplement: S2 File — The raw data are presented in Raw data.zip. (ZIP) [file pone.0339611.s002.zip › Raw data/Figure 1/WB-IL1RA cell line/target/cell line.tif]

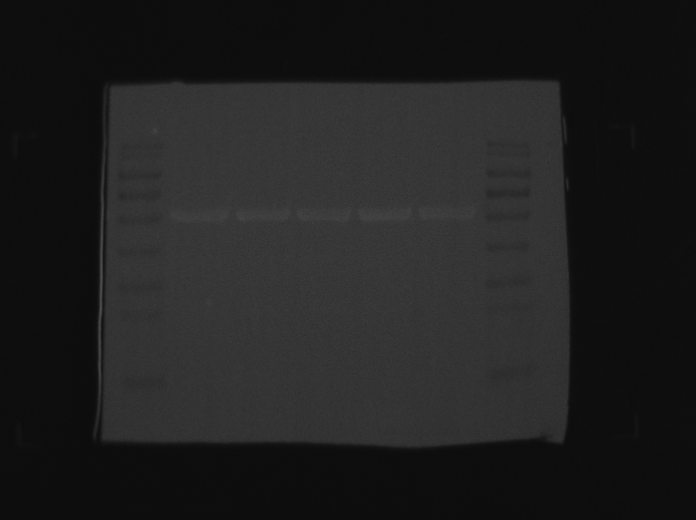

Supplement: S2 File — The raw data are presented in Raw data.zip. (ZIP) [file pone.0339611.s002.zip › Raw data/Figure 1/WB-IL1RA cell line/tubulin/cellline'.tif]

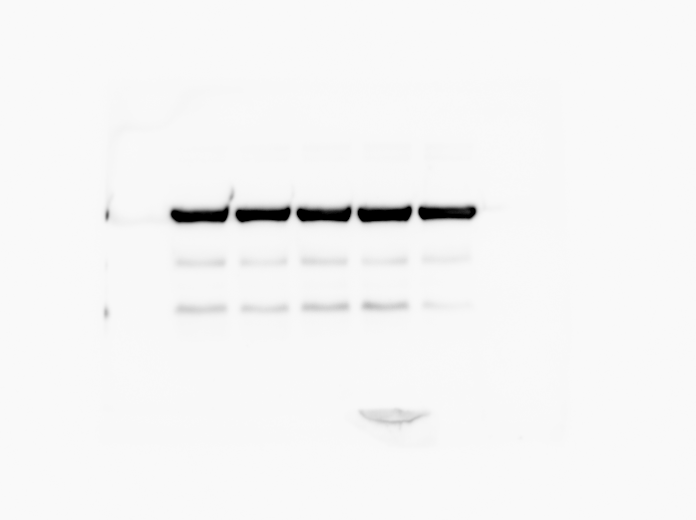

Supplement: S2 File — The raw data are presented in Raw data.zip. (ZIP) [file pone.0339611.s002.zip › Raw data/Figure 1/WB-IL1RA cell line/tubulin/cellline.tif]

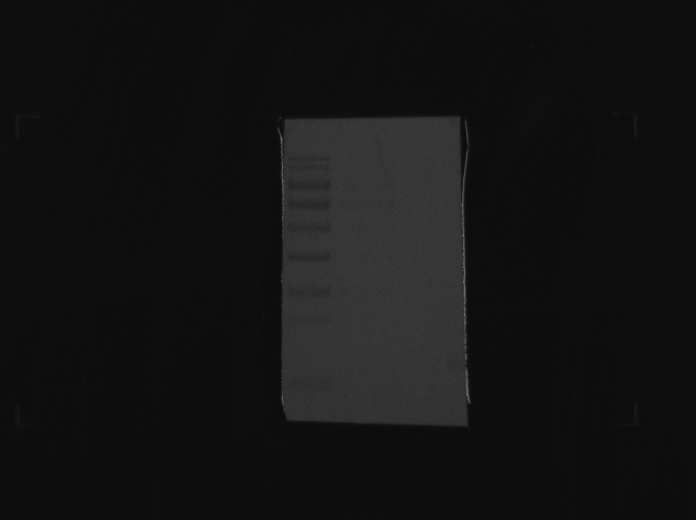

Supplement: S2 File — The raw data are presented in Raw data.zip. (ZIP) [file pone.0339611.s002.zip › Raw data/Figure 2/WB-IL1RA cell line/C4-2B-OE/target/oe3'.tif]

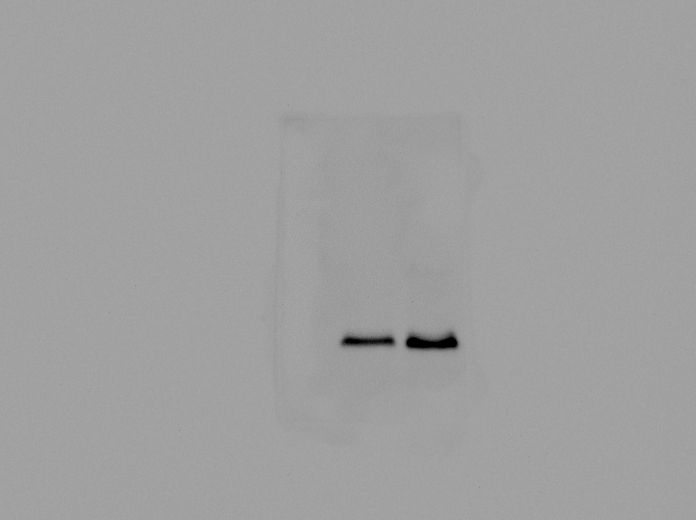

Supplement: S2 File — The raw data are presented in Raw data.zip. (ZIP) [file pone.0339611.s002.zip › Raw data/Figure 2/WB-IL1RA cell line/C4-2B-OE/target/oe3.tif]

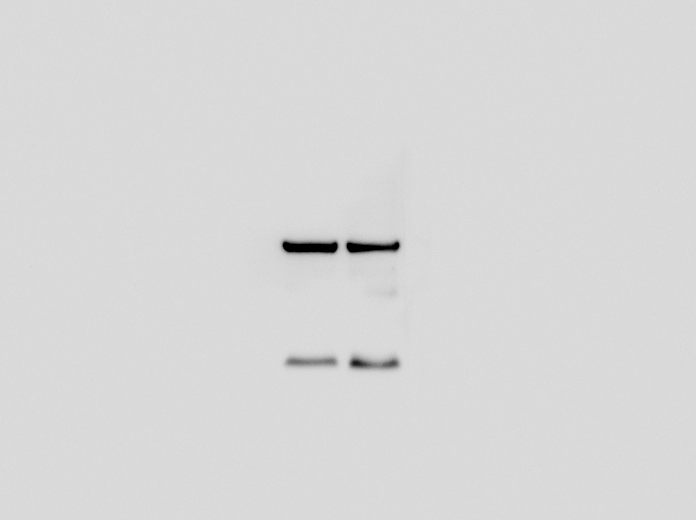

Supplement: S2 File — The raw data are presented in Raw data.zip. (ZIP) [file pone.0339611.s002.zip › Raw data/Figure 2/WB-IL1RA cell line/C4-2B-OE/tubulin/oe3'.tif]

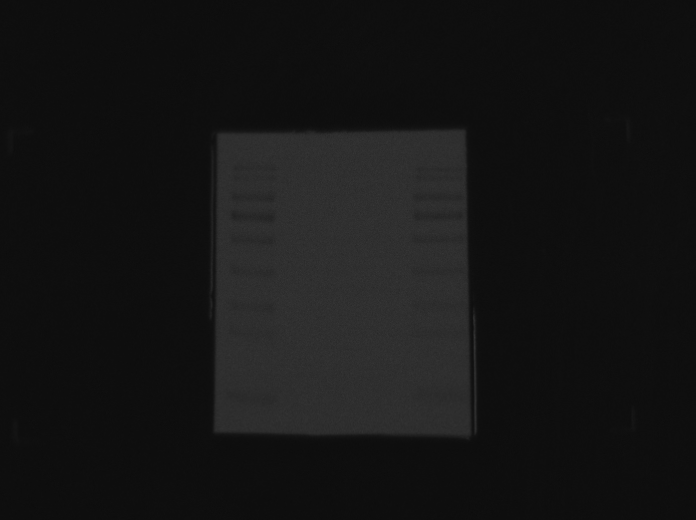

Supplement: S2 File — The raw data are presented in Raw data.zip. (ZIP) [file pone.0339611.s002.zip › Raw data/Figure 2/WB-IL1RA cell line/LNCap-OE/target/oe'.tif]

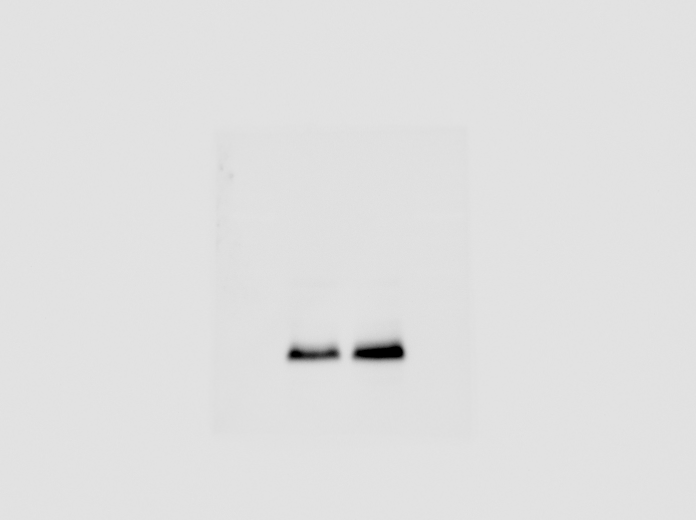

Supplement: S2 File — The raw data are presented in Raw data.zip. (ZIP) [file pone.0339611.s002.zip › Raw data/Figure 2/WB-IL1RA cell line/LNCap-OE/target/oe.tif]

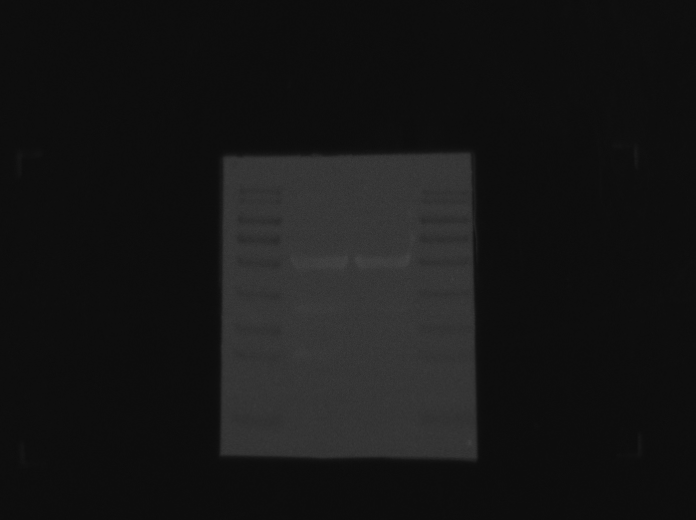

Supplement: S2 File — The raw data are presented in Raw data.zip. (ZIP) [file pone.0339611.s002.zip › Raw data/Figure 2/WB-IL1RA cell line/LNCap-OE/tubulin/oe'.tif]

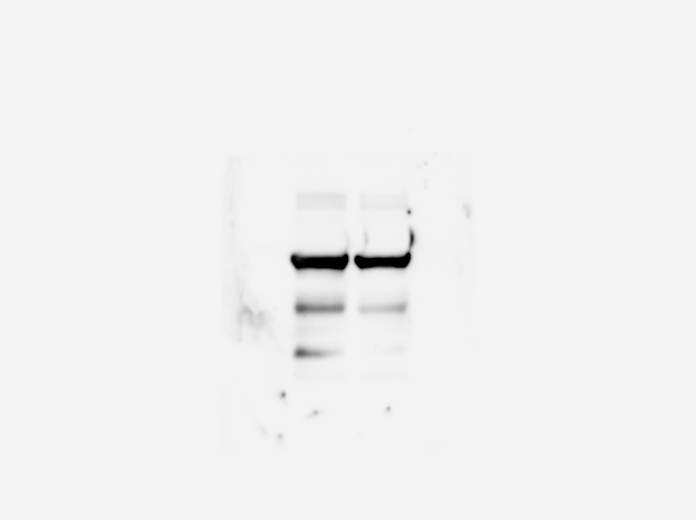

Supplement: S2 File — The raw data are presented in Raw data.zip. (ZIP) [file pone.0339611.s002.zip › Raw data/Figure 2/WB-IL1RA cell line/LNCap-OE/tubulin/oe.tif]

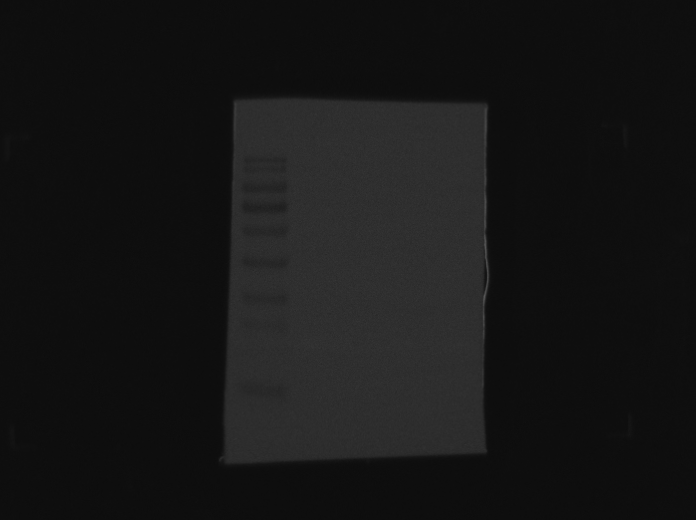

Supplement: S2 File — The raw data are presented in Raw data.zip. (ZIP) [file pone.0339611.s002.zip › Raw data/Figure 2/WB-IL1RA cell line/sh/target/sh'.tif]

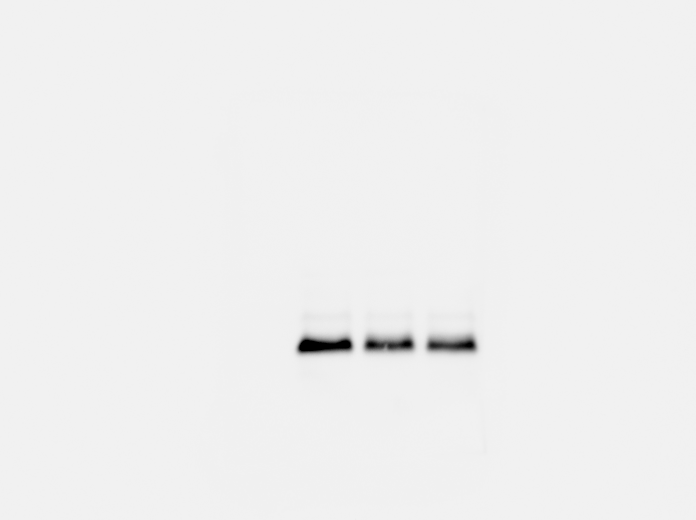

Supplement: S2 File — The raw data are presented in Raw data.zip. (ZIP) [file pone.0339611.s002.zip › Raw data/Figure 2/WB-IL1RA cell line/sh/target/sh.tif]

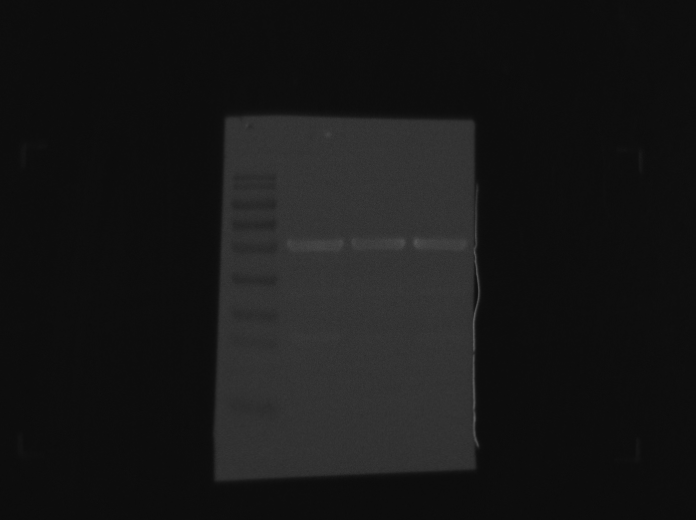

Supplement: S2 File — The raw data are presented in Raw data.zip. (ZIP) [file pone.0339611.s002.zip › Raw data/Figure 2/WB-IL1RA cell line/sh/tubulin/sh'.tif]

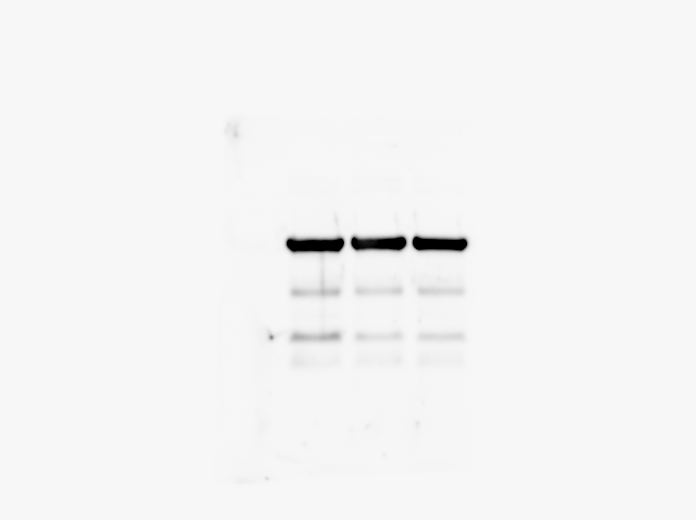

Supplement: S2 File — The raw data are presented in Raw data.zip. (ZIP) [file pone.0339611.s002.zip › Raw data/Figure 2/WB-IL1RA cell line/sh/tubulin/sh.tif]

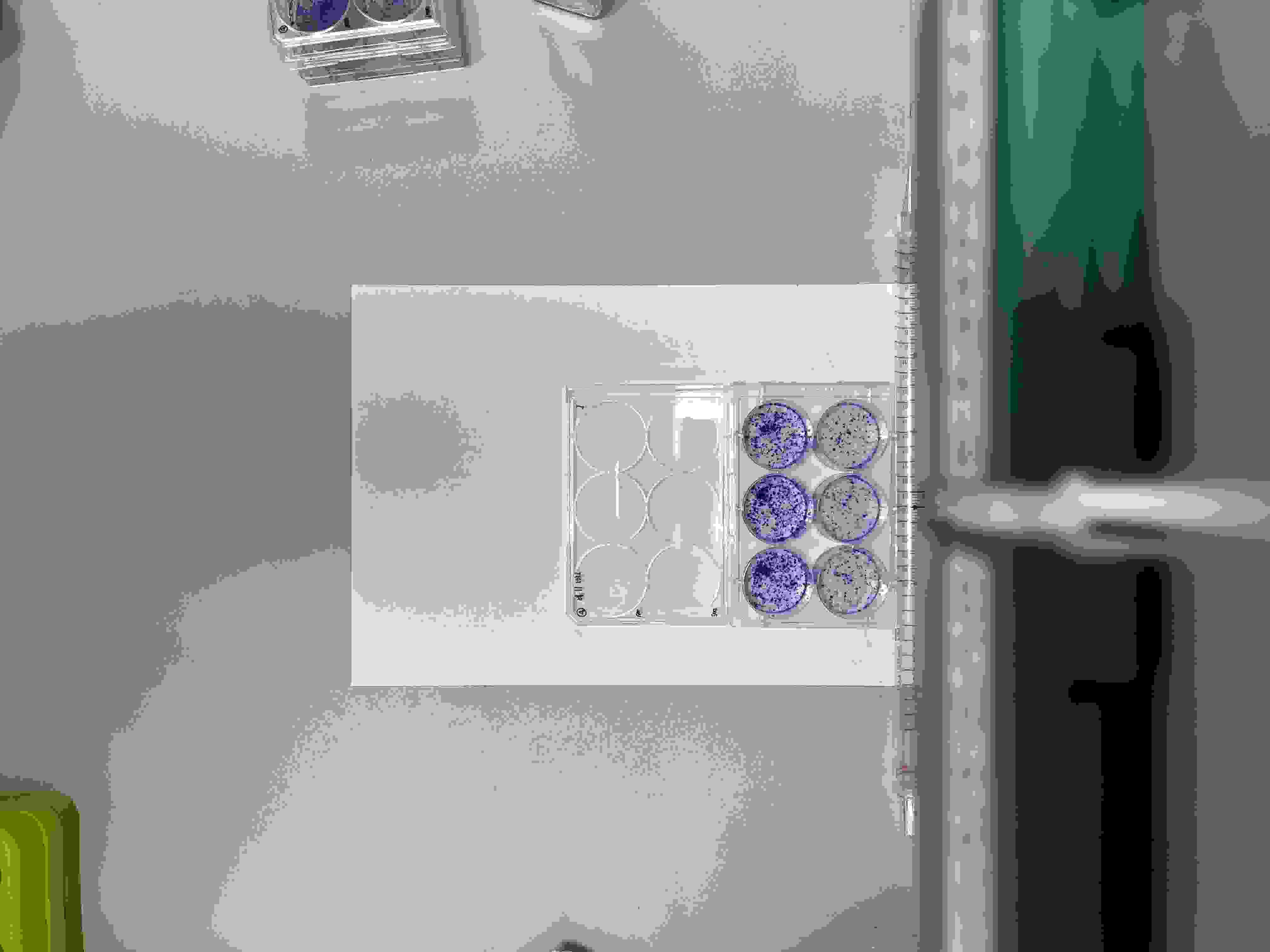

Supplement: S2 File — The raw data are presented in Raw data.zip. (ZIP) [file pone.0339611.s002.zip › Raw data/Figure 3/colony-IL1RA/picture_202212081223481.jpg]

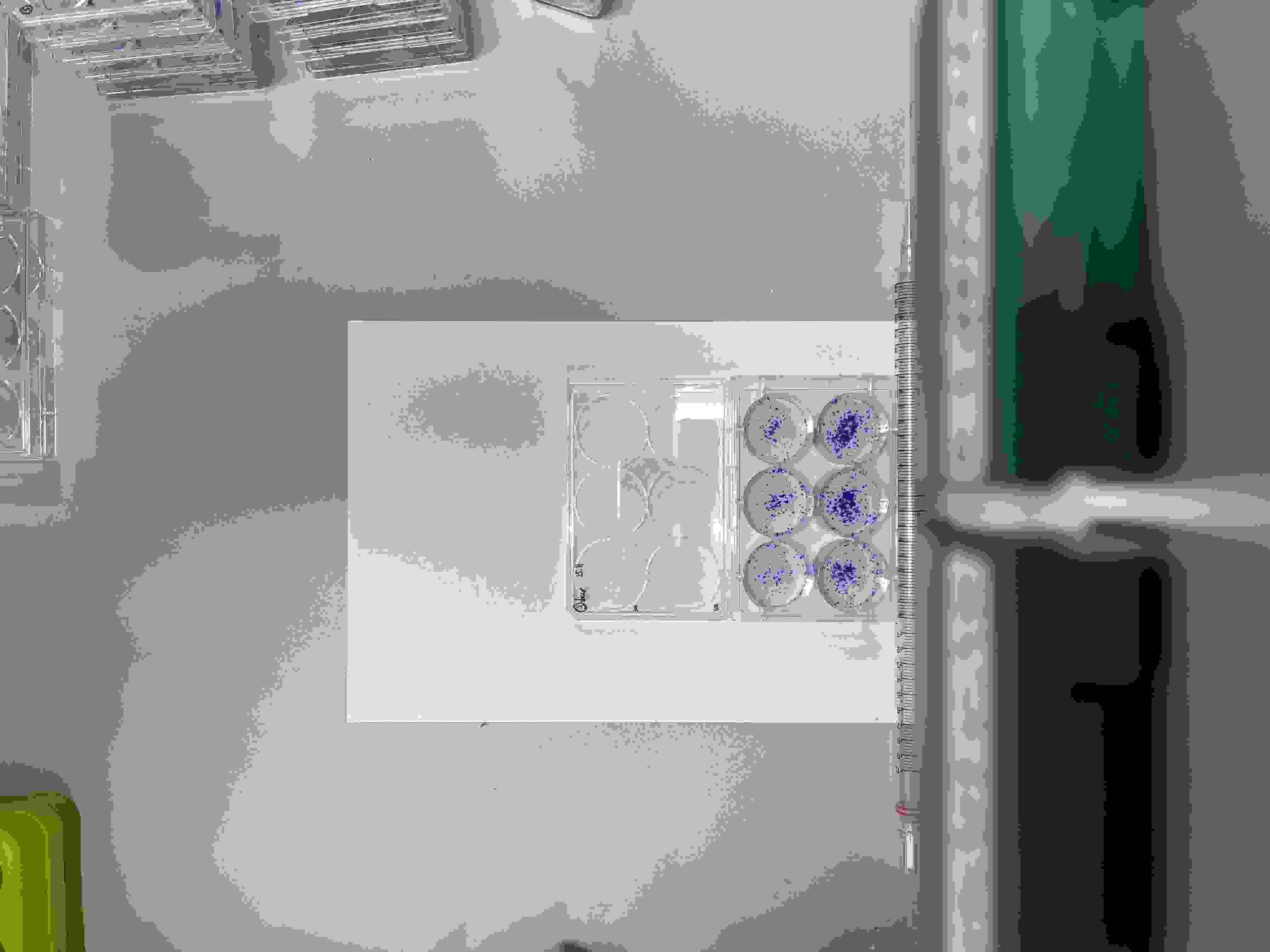

Supplement: S2 File — The raw data are presented in Raw data.zip. (ZIP) [file pone.0339611.s002.zip › Raw data/Figure 3/colony-IL1RA/picture_202212081223485.jpg]

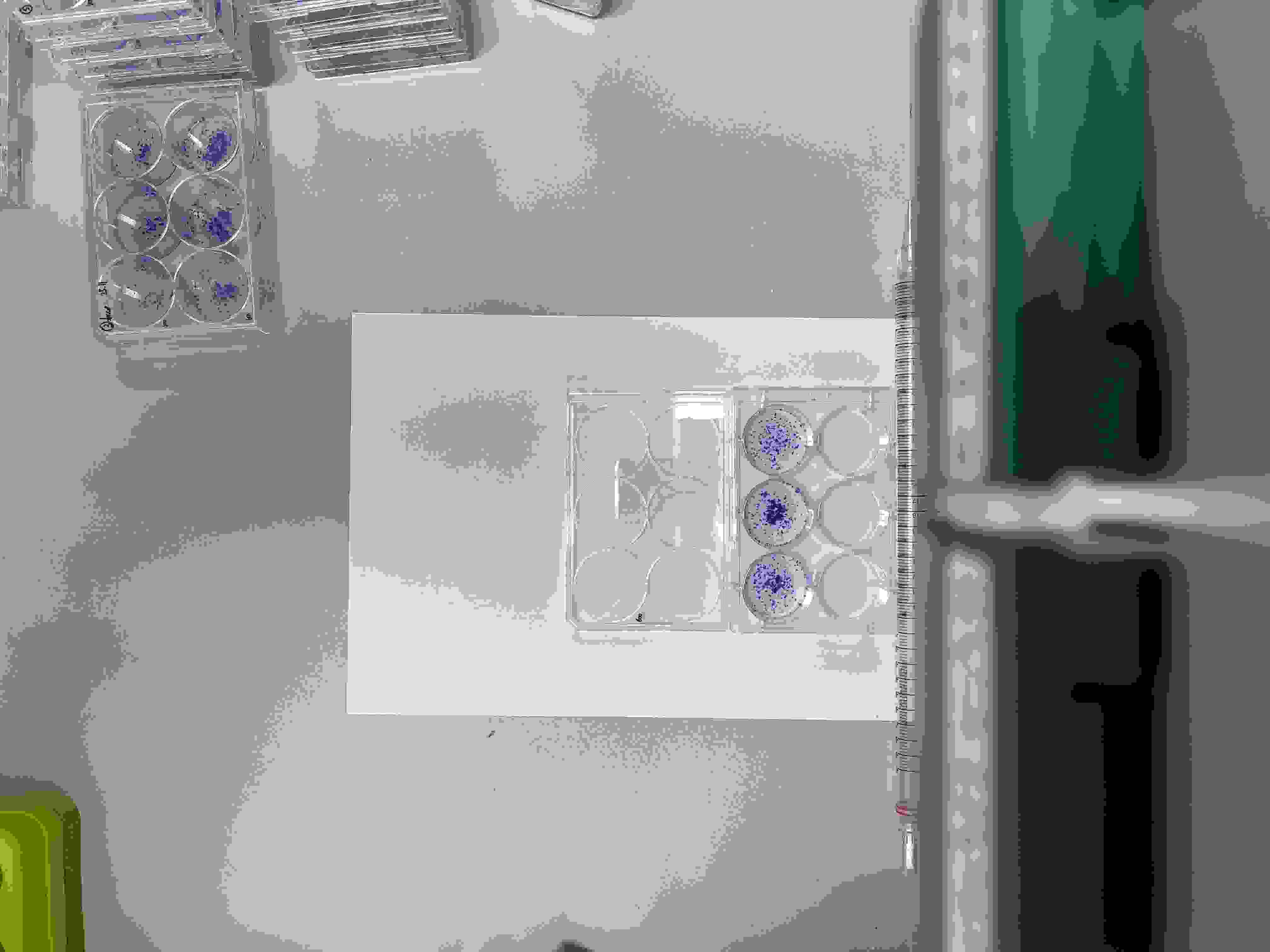

Supplement: S2 File — The raw data are presented in Raw data.zip. (ZIP) [file pone.0339611.s002.zip › Raw data/Figure 3/colony-IL1RA/picture_202212081223486.jpg]

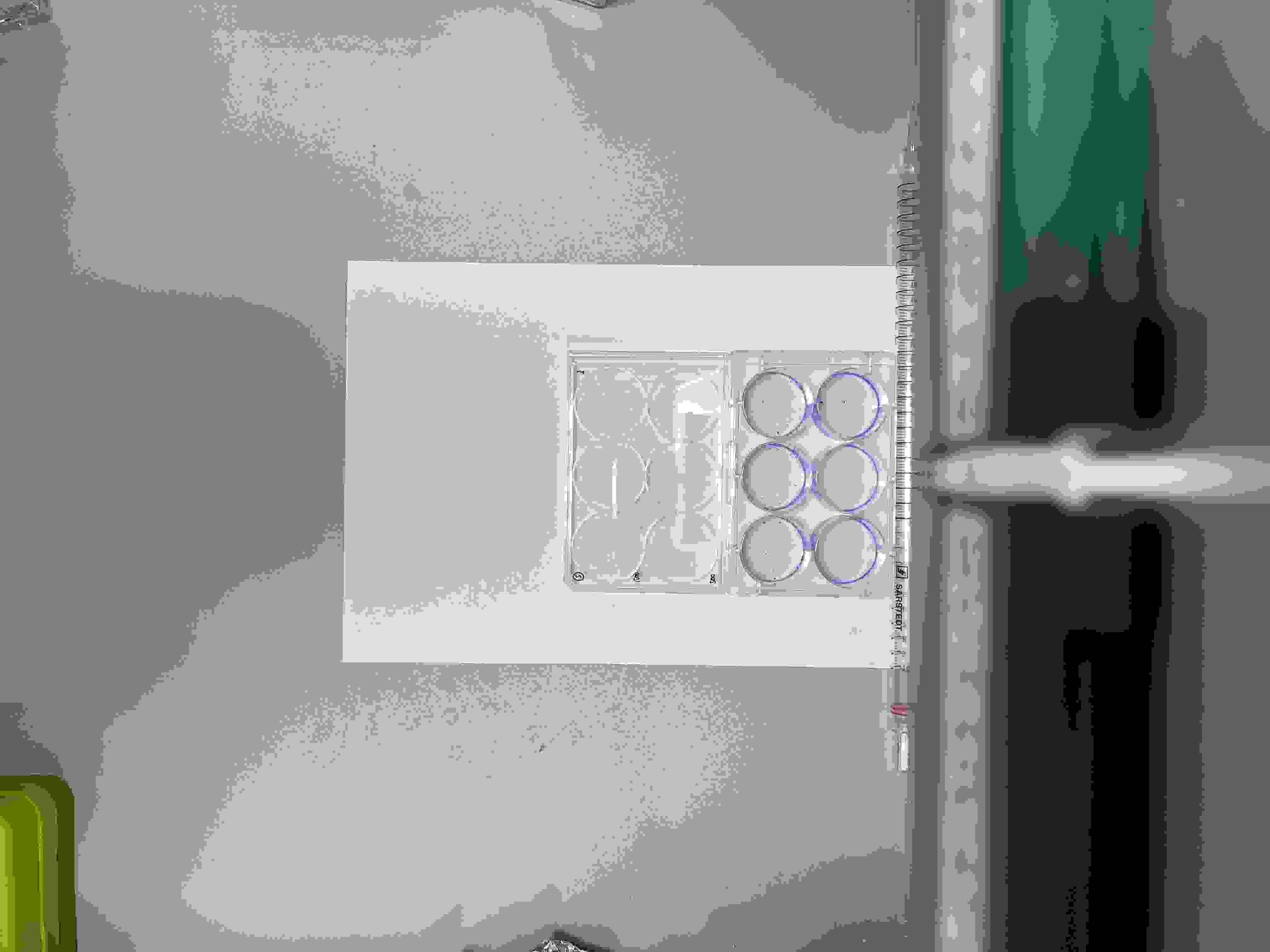

Supplement: S2 File — The raw data are presented in Raw data.zip. (ZIP) [file pone.0339611.s002.zip › Raw data/Figure 3/colony-IL1RA/picture_20221208124436.jpg]

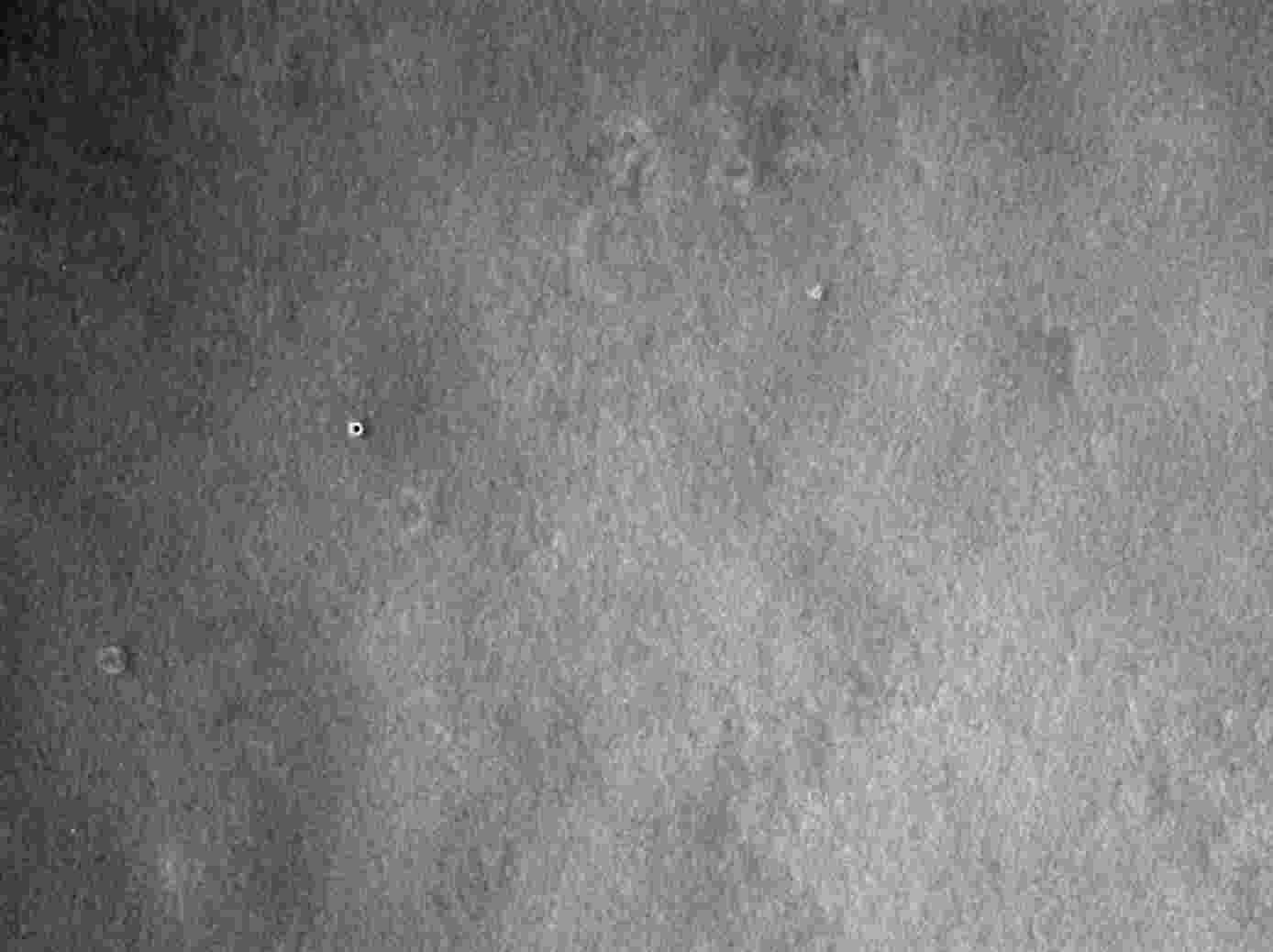

Supplement: S2 File — The raw data are presented in Raw data.zip. (ZIP) [file pone.0339611.s002.zip › Raw data/Figure 4/soft agar/day 1/3+shI-1-day1 (10).jpg]

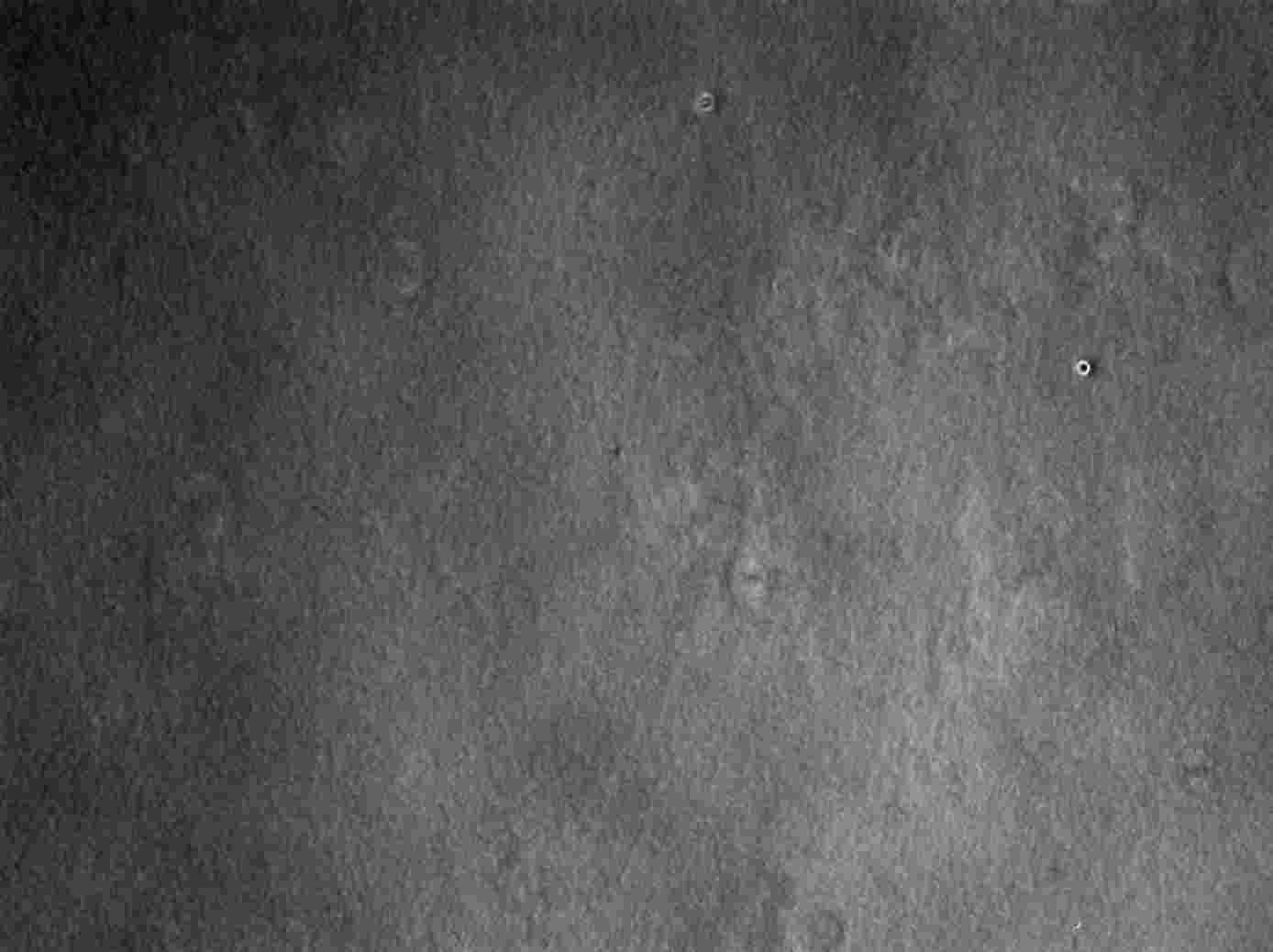

Supplement: S2 File — The raw data are presented in Raw data.zip. (ZIP) [file pone.0339611.s002.zip › Raw data/Figure 4/soft agar/day 1/3+shI-1-day1 (11).jpg]

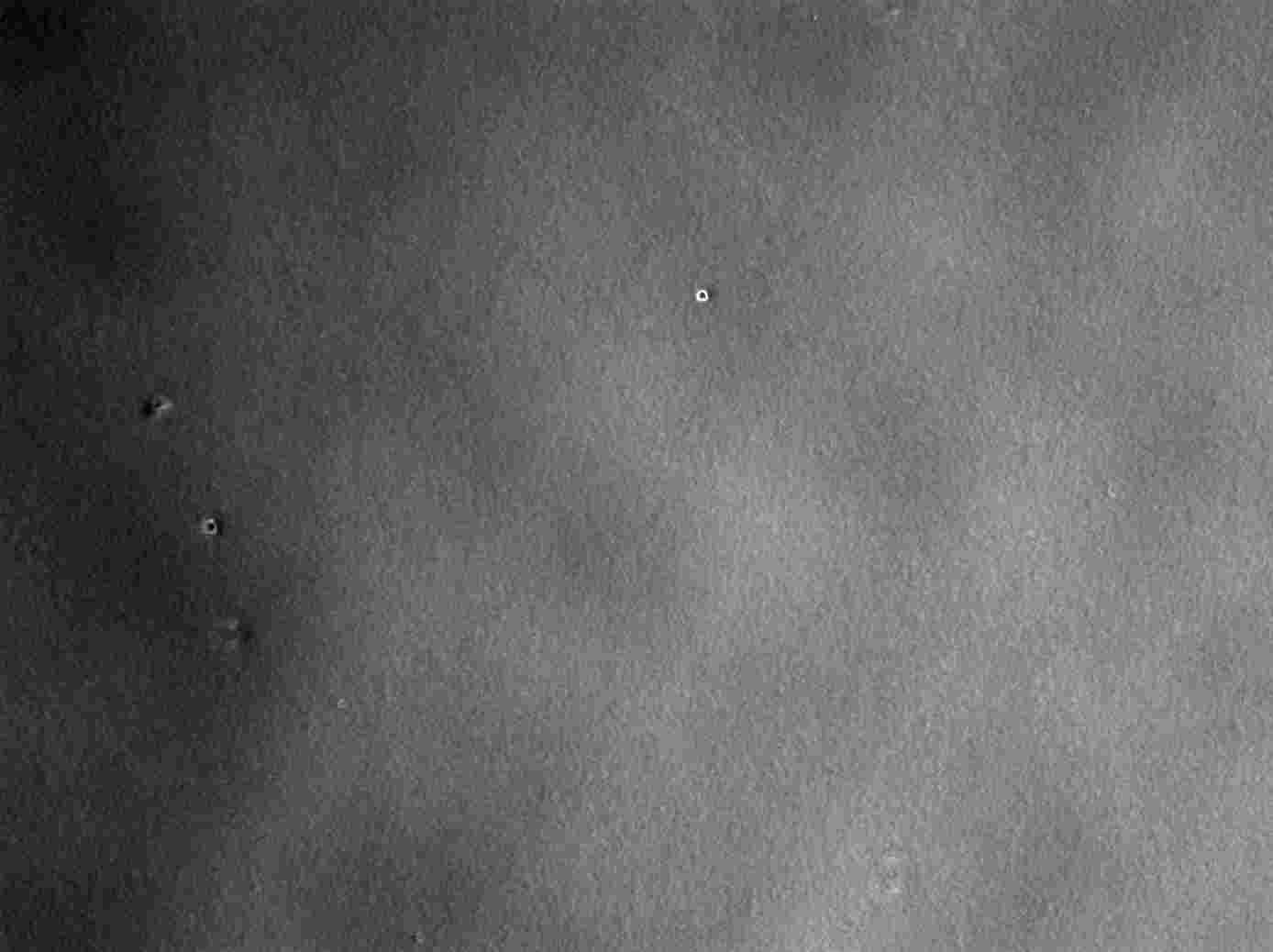

Supplement: S2 File — The raw data are presented in Raw data.zip. (ZIP) [file pone.0339611.s002.zip › Raw data/Figure 4/soft agar/day 1/3+shI-1-day1 (12).jpg]

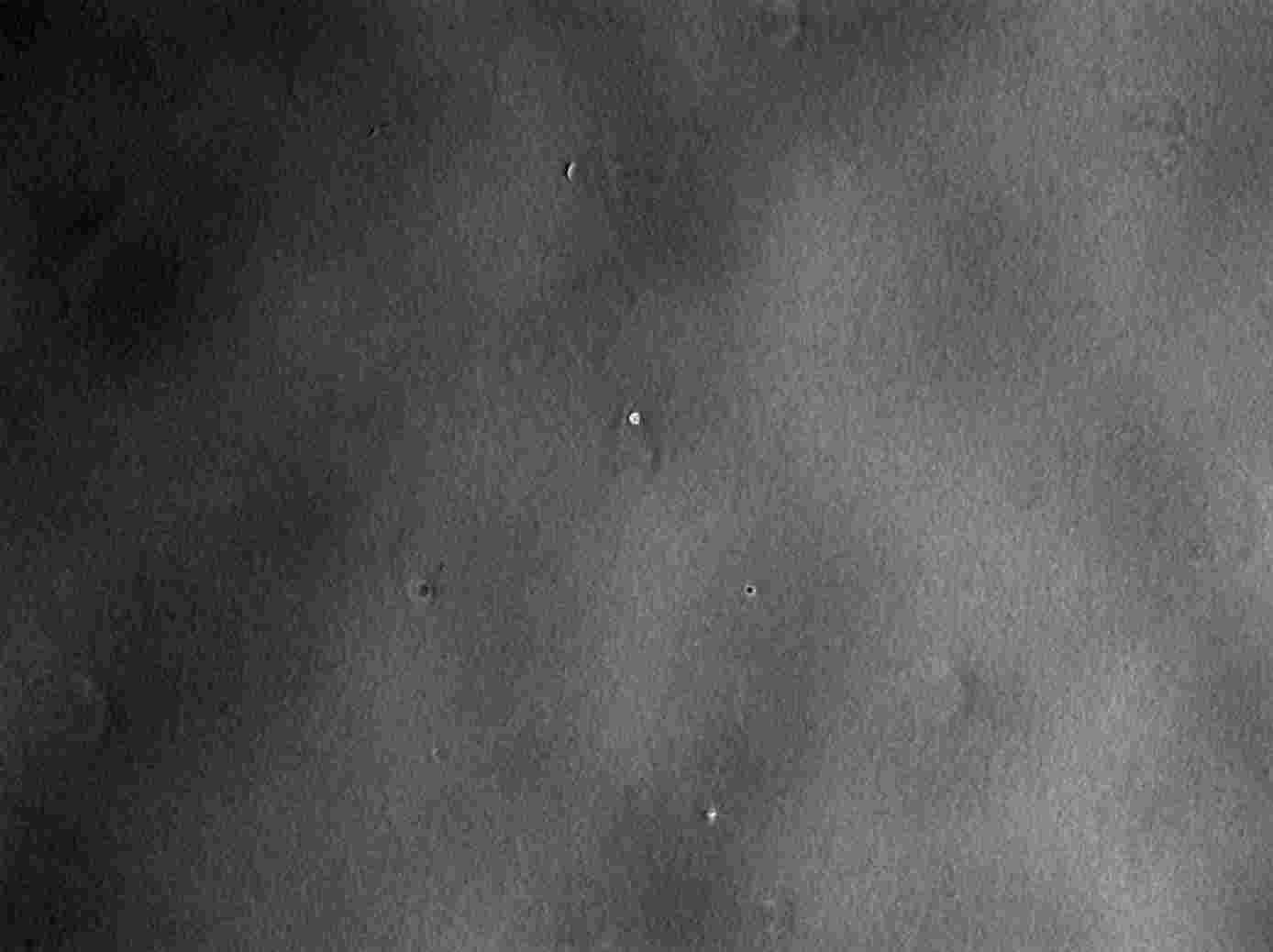

Supplement: S2 File — The raw data are presented in Raw data.zip. (ZIP) [file pone.0339611.s002.zip › Raw data/Figure 4/soft agar/day 1/3+shI-1-day1 (13).jpg]

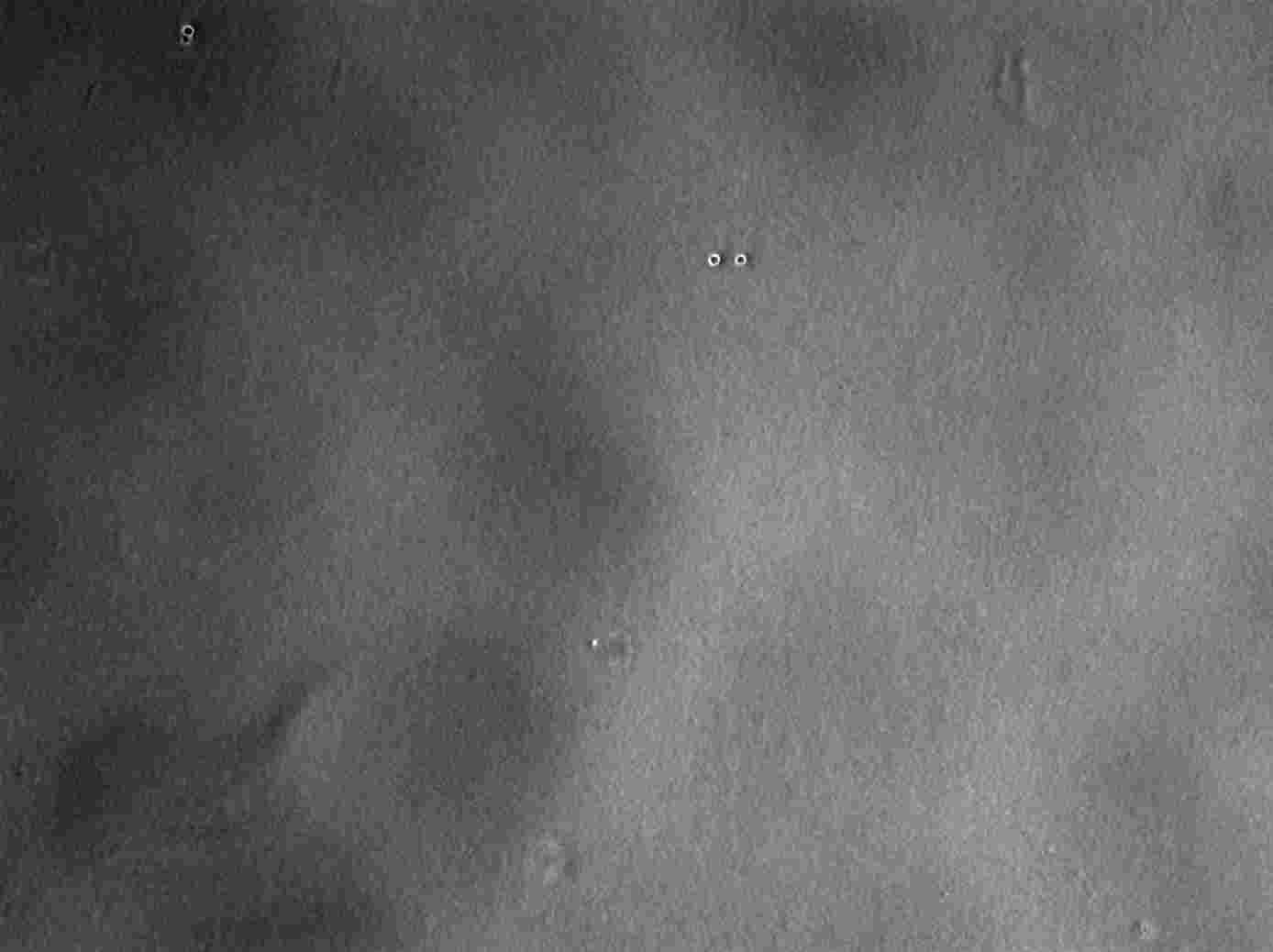

Supplement: S2 File — The raw data are presented in Raw data.zip. (ZIP) [file pone.0339611.s002.zip › Raw data/Figure 4/soft agar/day 1/3+shI-1-day1 (14).jpg]

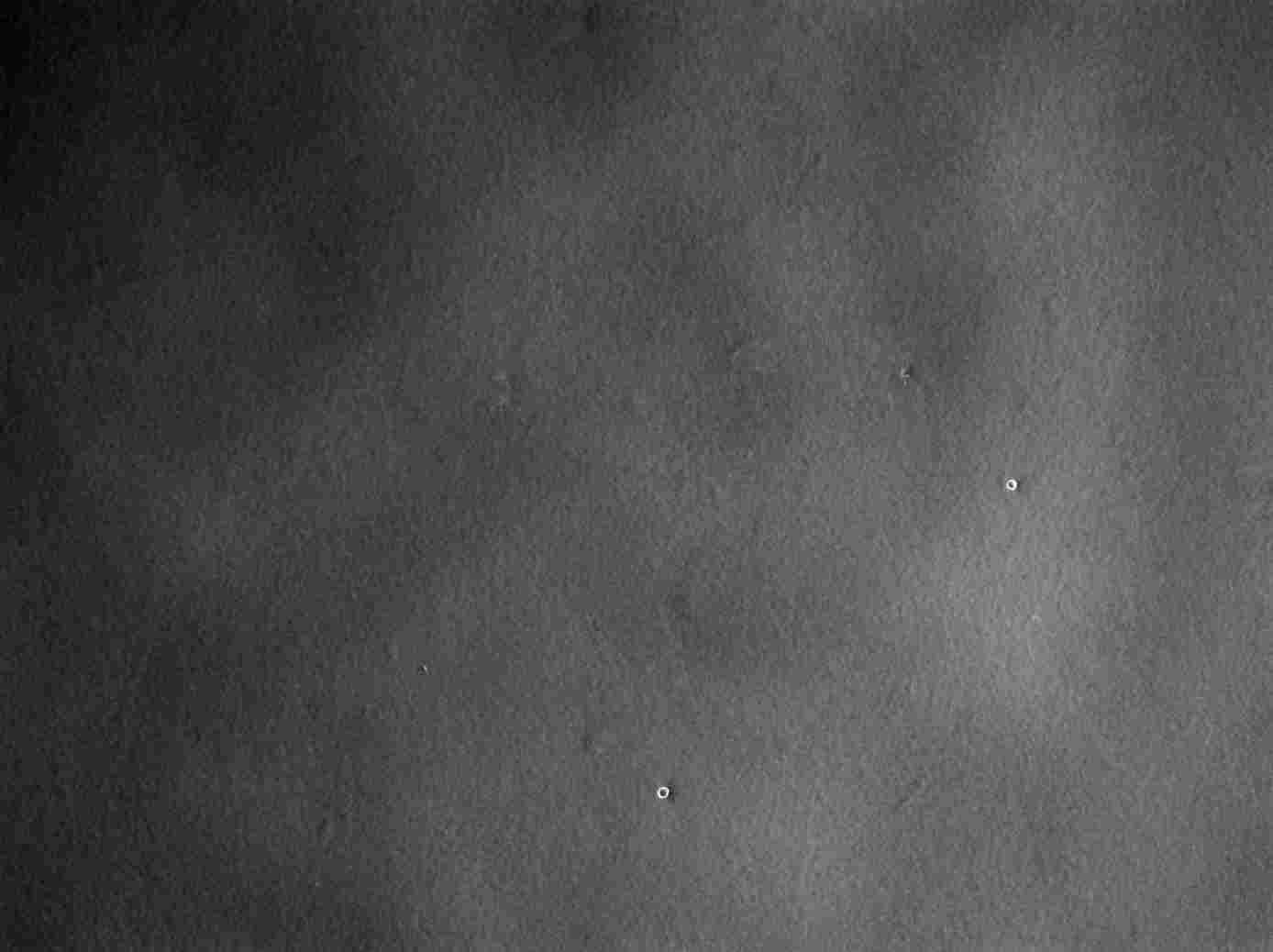

Supplement: S2 File — The raw data are presented in Raw data.zip. (ZIP) [file pone.0339611.s002.zip › Raw data/Figure 4/soft agar/day 1/3+shI-1-day1 (15).jpg]

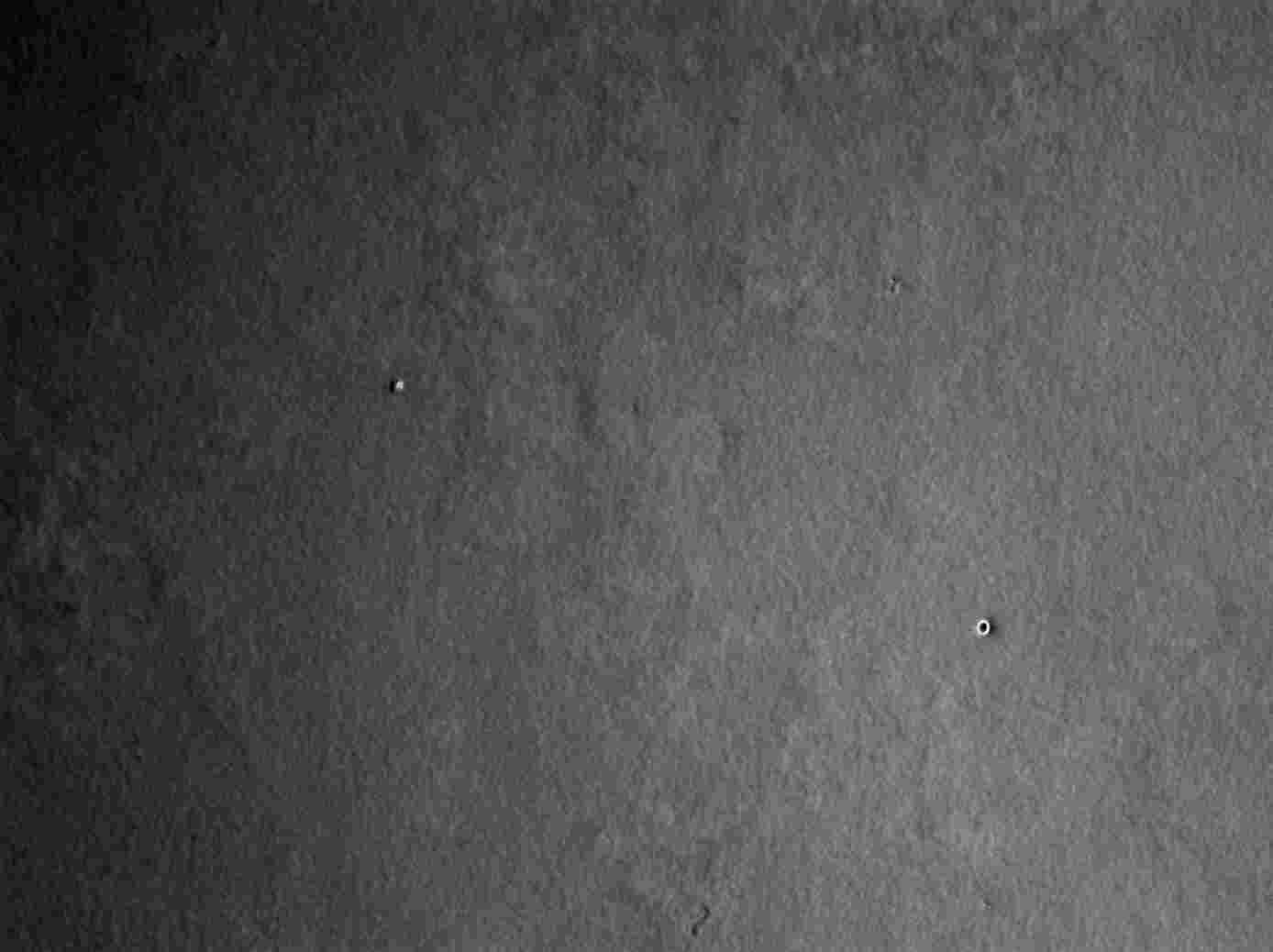

Supplement: S2 File — The raw data are presented in Raw data.zip. (ZIP) [file pone.0339611.s002.zip › Raw data/Figure 4/soft agar/day 1/3+shI-1-day1 (2).jpg]

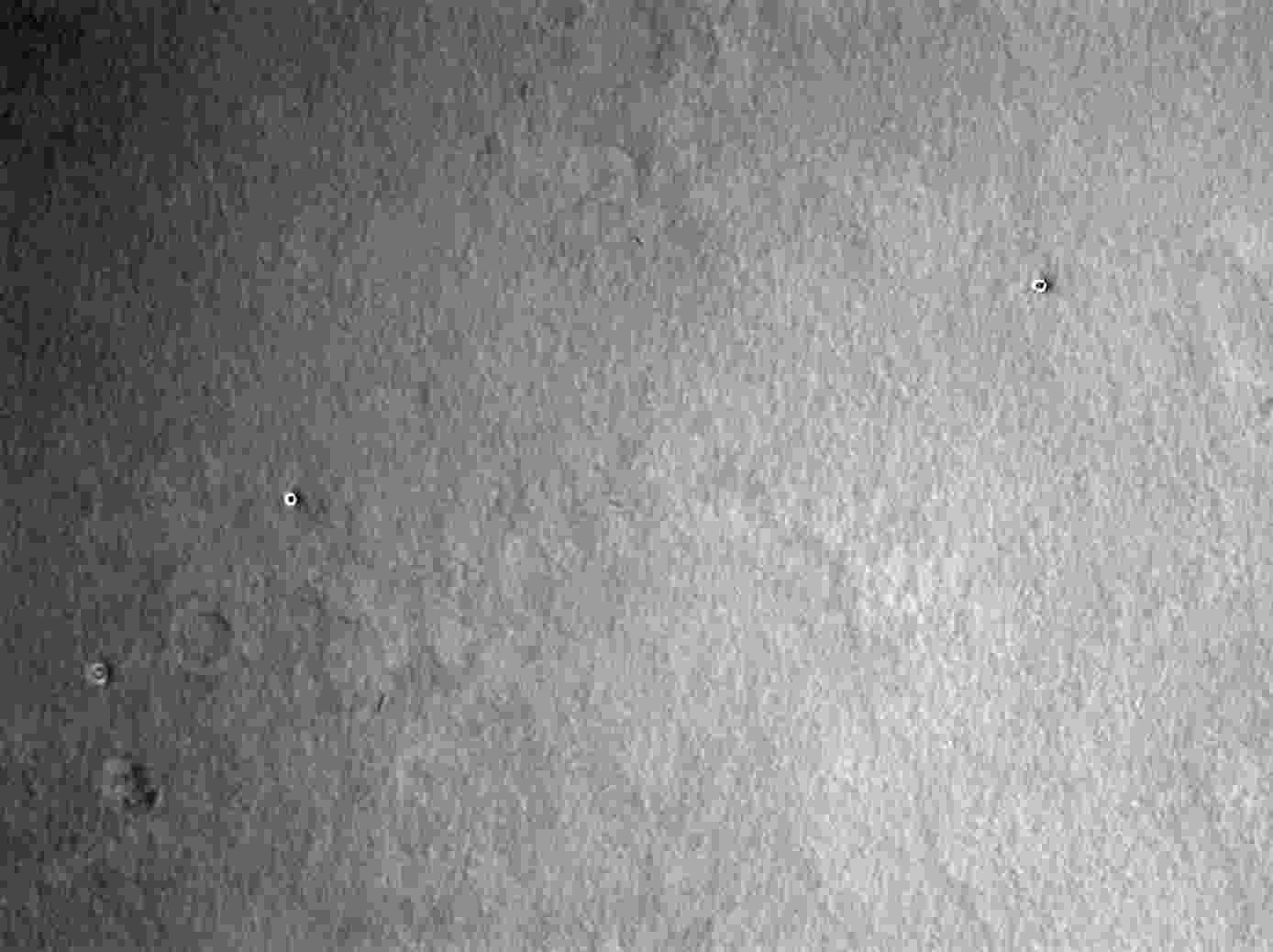

Supplement: S2 File — The raw data are presented in Raw data.zip. (ZIP) [file pone.0339611.s002.zip › Raw data/Figure 4/soft agar/day 1/3+shI-1-day1 (3).jpg]

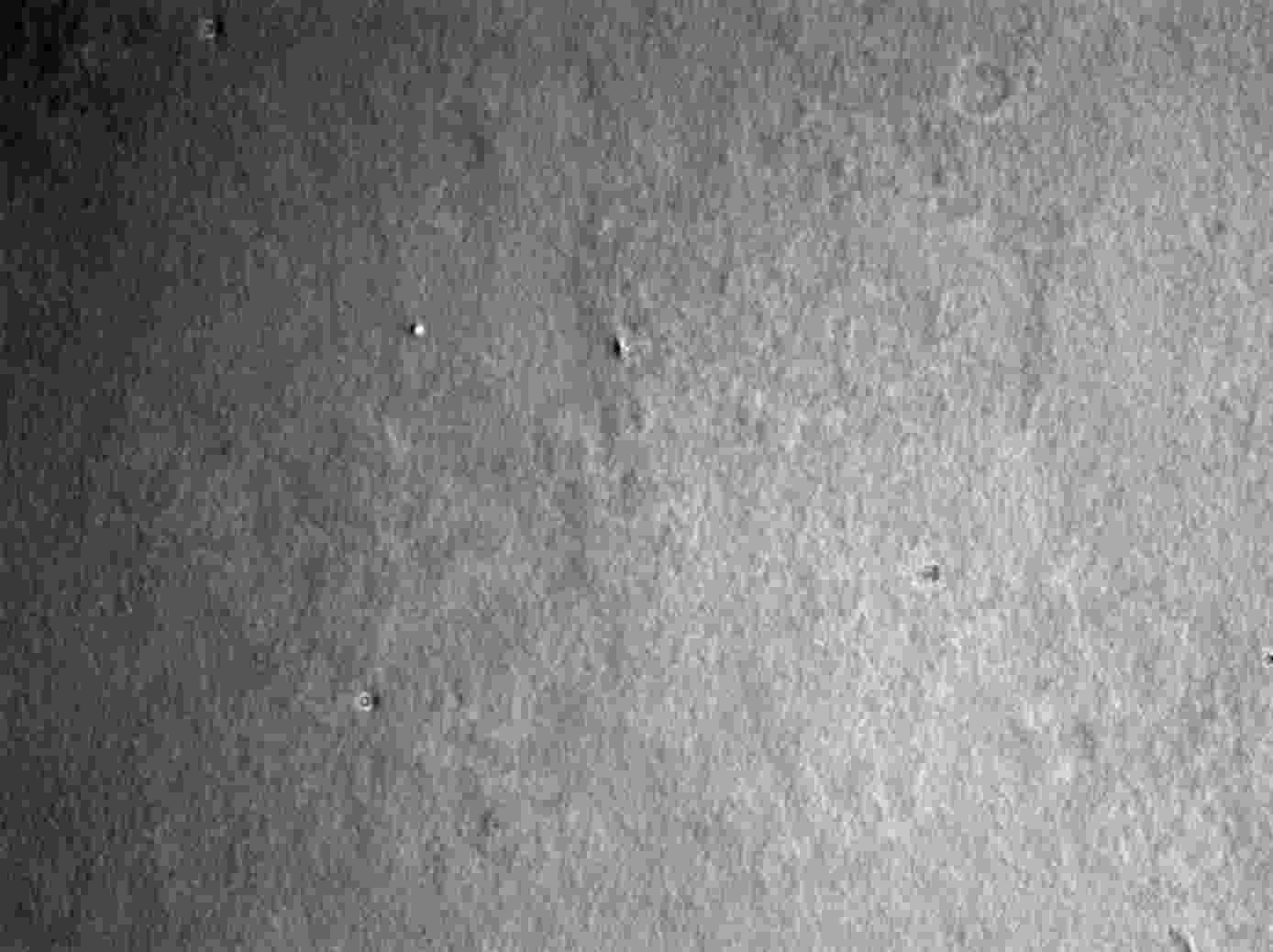

Supplement: S2 File — The raw data are presented in Raw data.zip. (ZIP) [file pone.0339611.s002.zip › Raw data/Figure 4/soft agar/day 1/3+shI-1-day1 (4).jpg]

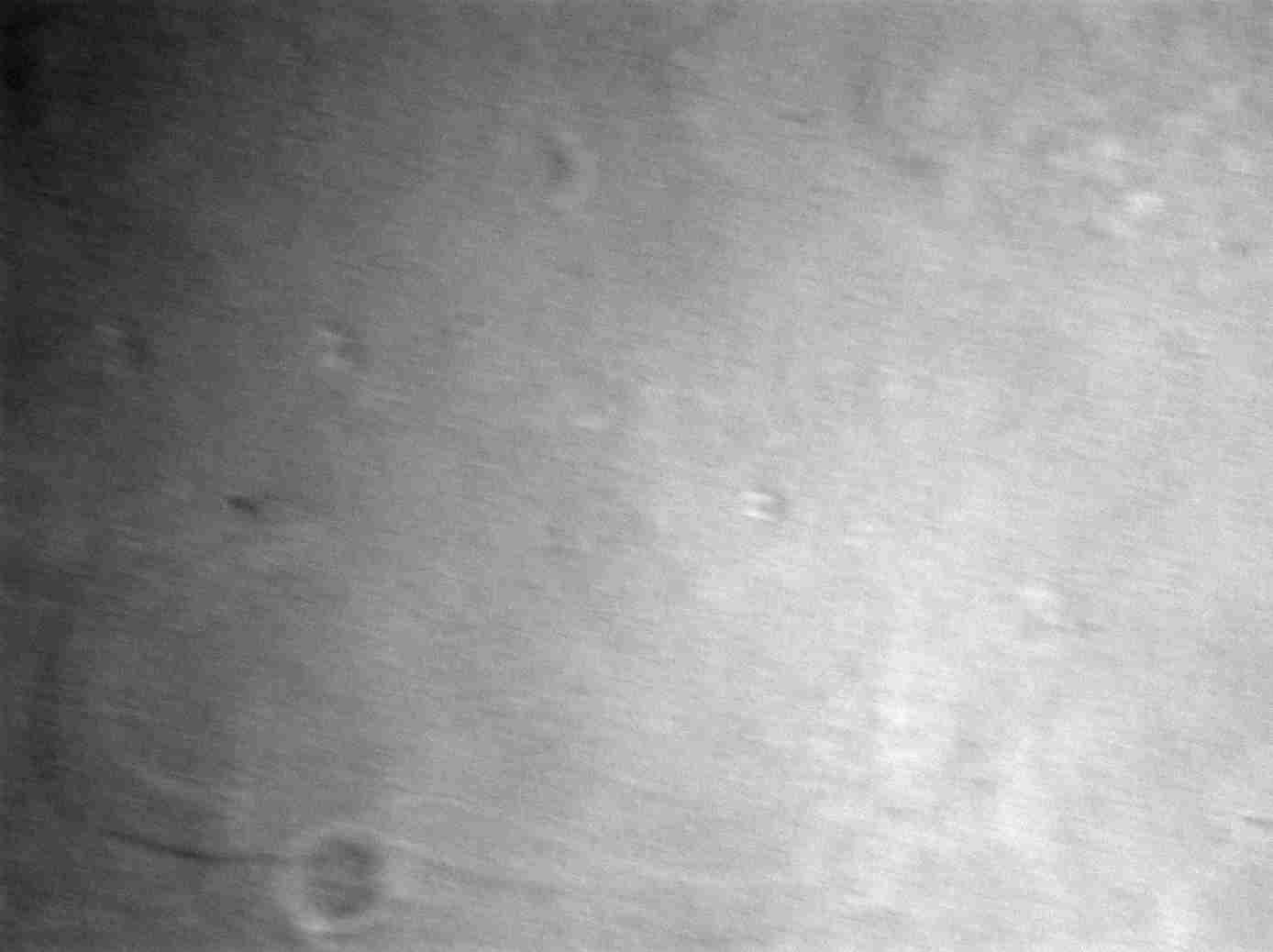

Supplement: S2 File — The raw data are presented in Raw data.zip. (ZIP) [file pone.0339611.s002.zip › Raw data/Figure 4/soft agar/day 1/3+shI-1-day1 (5).jpg]

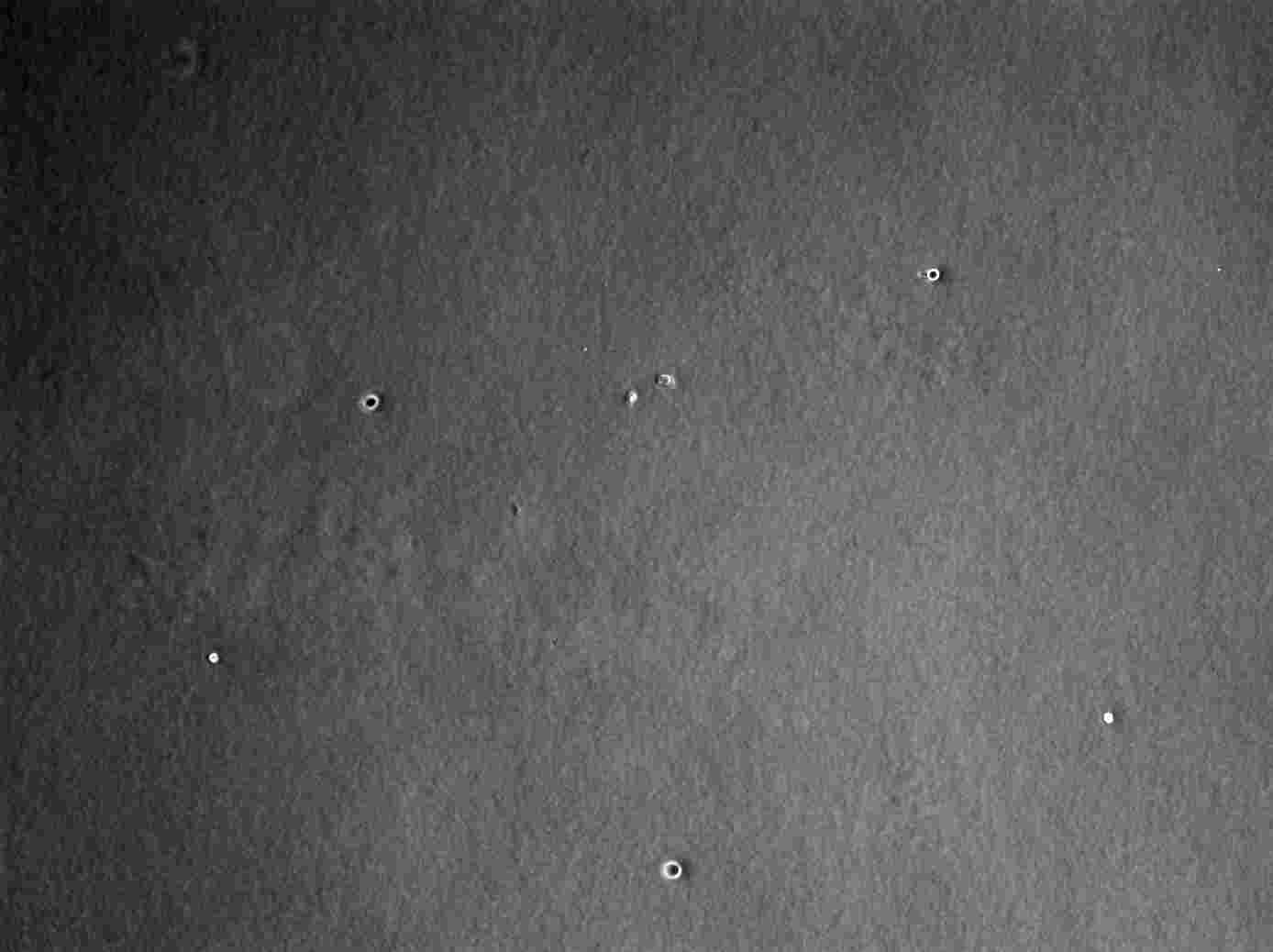

Supplement: S2 File — The raw data are presented in Raw data.zip. (ZIP) [file pone.0339611.s002.zip › Raw data/Figure 4/soft agar/day 1/3+shI-1-day1 (6).jpg]

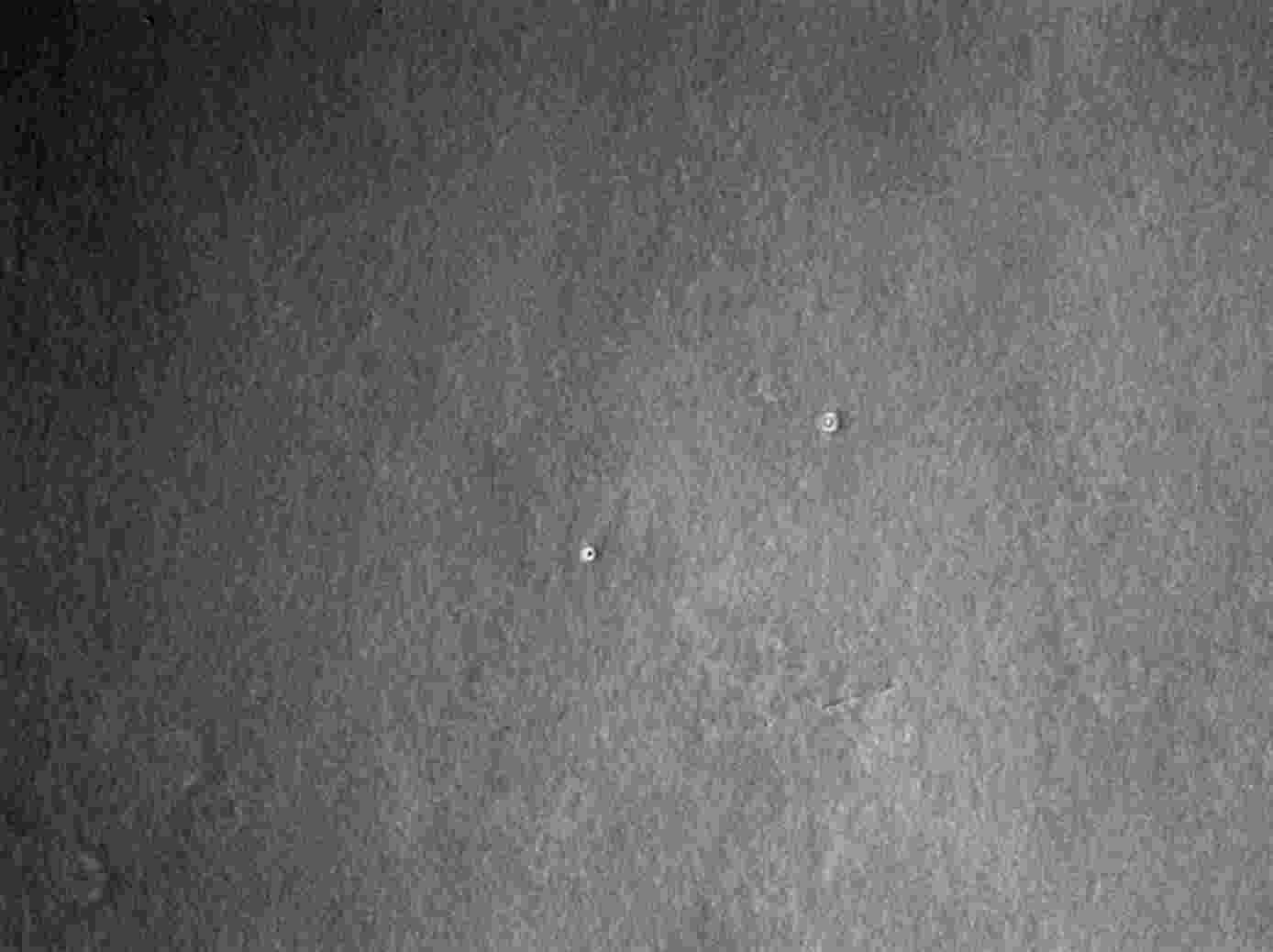

Supplement: S2 File — The raw data are presented in Raw data.zip. (ZIP) [file pone.0339611.s002.zip › Raw data/Figure 4/soft agar/day 1/3+shI-1-day1 (7).jpg]

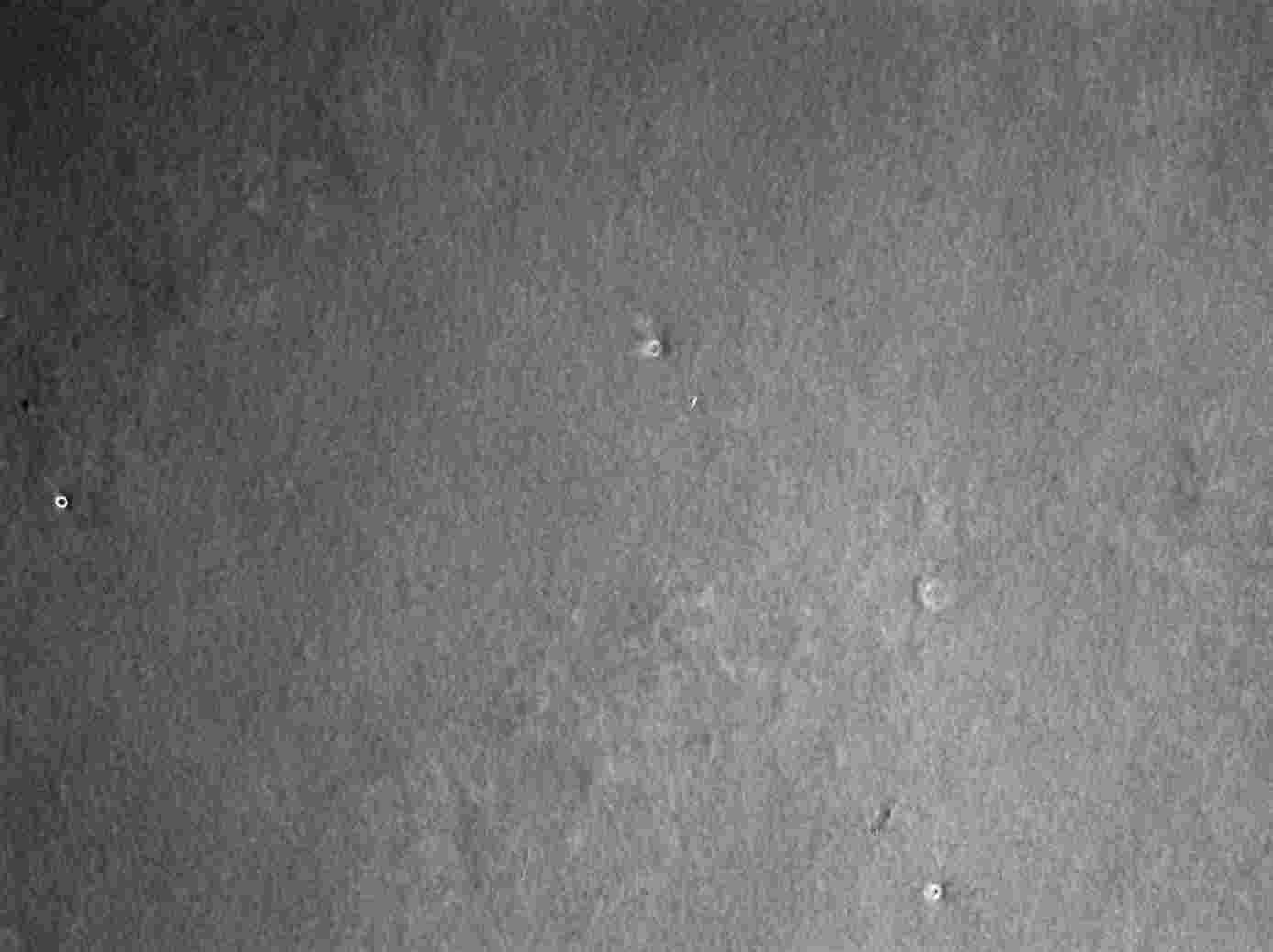

Supplement: S2 File — The raw data are presented in Raw data.zip. (ZIP) [file pone.0339611.s002.zip › Raw data/Figure 4/soft agar/day 1/3+shI-1-day1 (8).jpg]

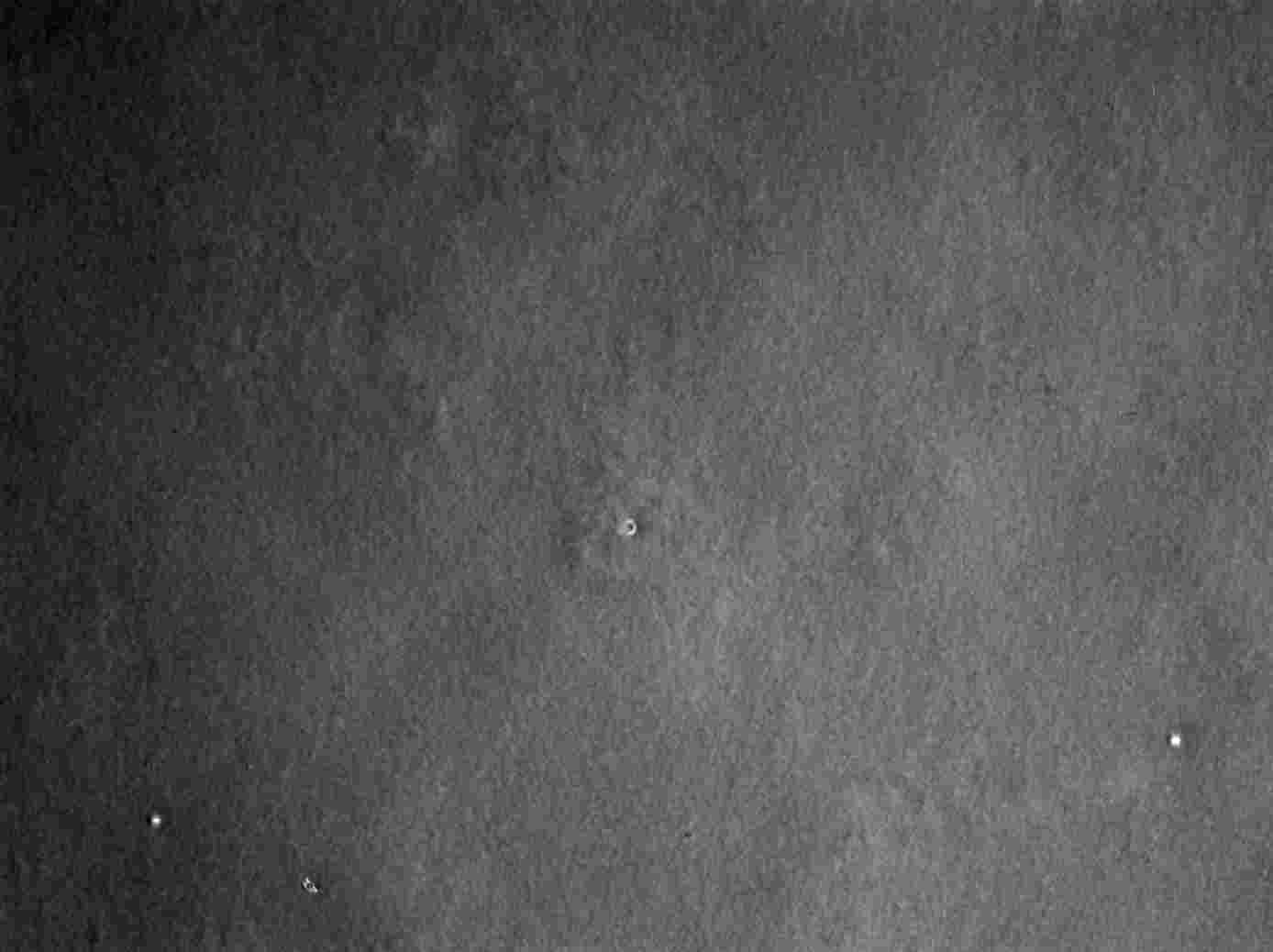

Supplement: S2 File — The raw data are presented in Raw data.zip. (ZIP) [file pone.0339611.s002.zip › Raw data/Figure 4/soft agar/day 1/3+shI-1-day1 (9).jpg]

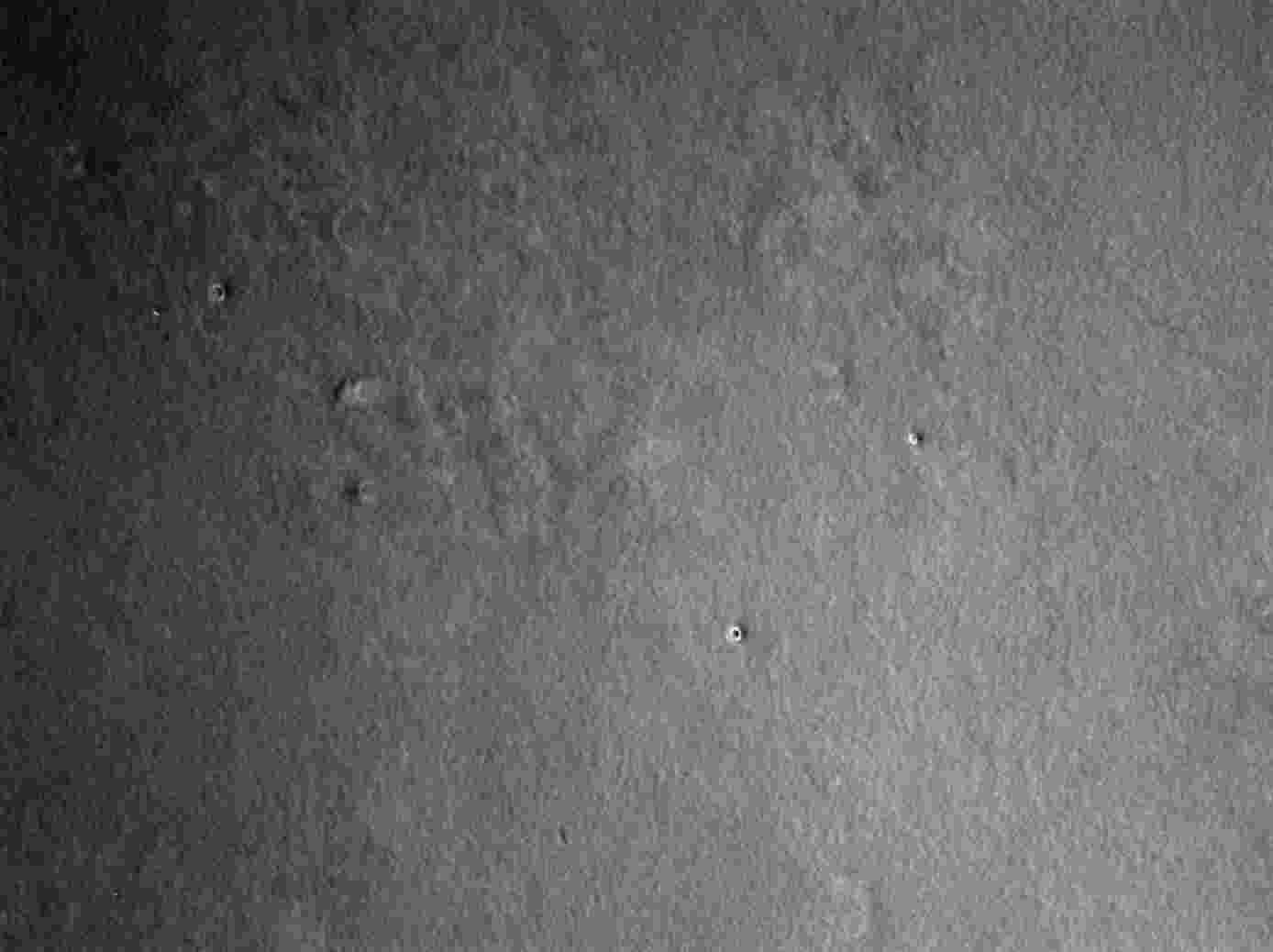

Supplement: S2 File — The raw data are presented in Raw data.zip. (ZIP) [file pone.0339611.s002.zip › Raw data/Figure 4/soft agar/day 1/3+shI-1-day1.jpg]

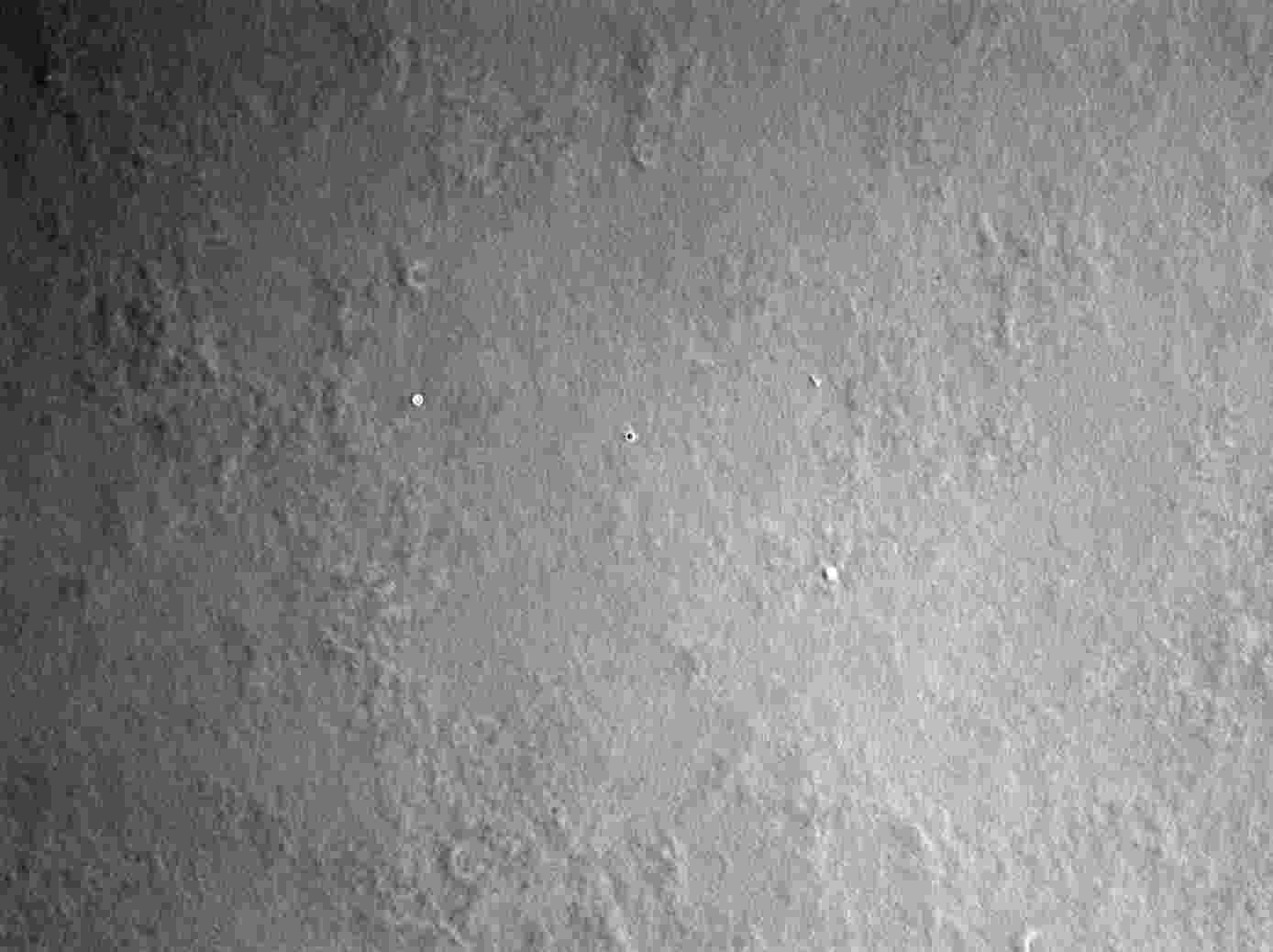

Supplement: S2 File — The raw data are presented in Raw data.zip. (ZIP) [file pone.0339611.s002.zip › Raw data/Figure 4/soft agar/day 1/3+shI-2-day1 (10).jpg]

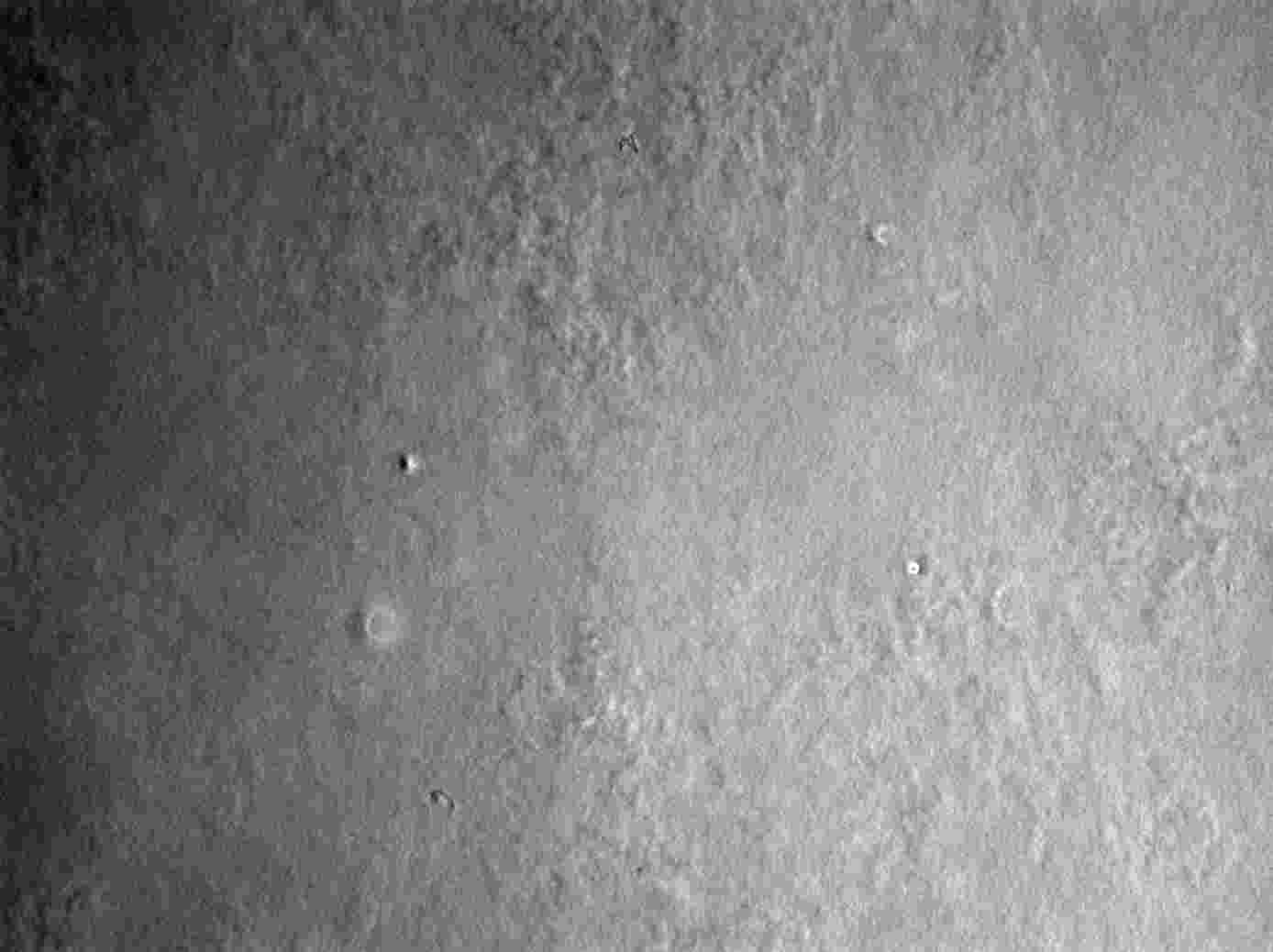

Supplement: S2 File — The raw data are presented in Raw data.zip. (ZIP) [file pone.0339611.s002.zip › Raw data/Figure 4/soft agar/day 1/3+shI-2-day1 (11).jpg]

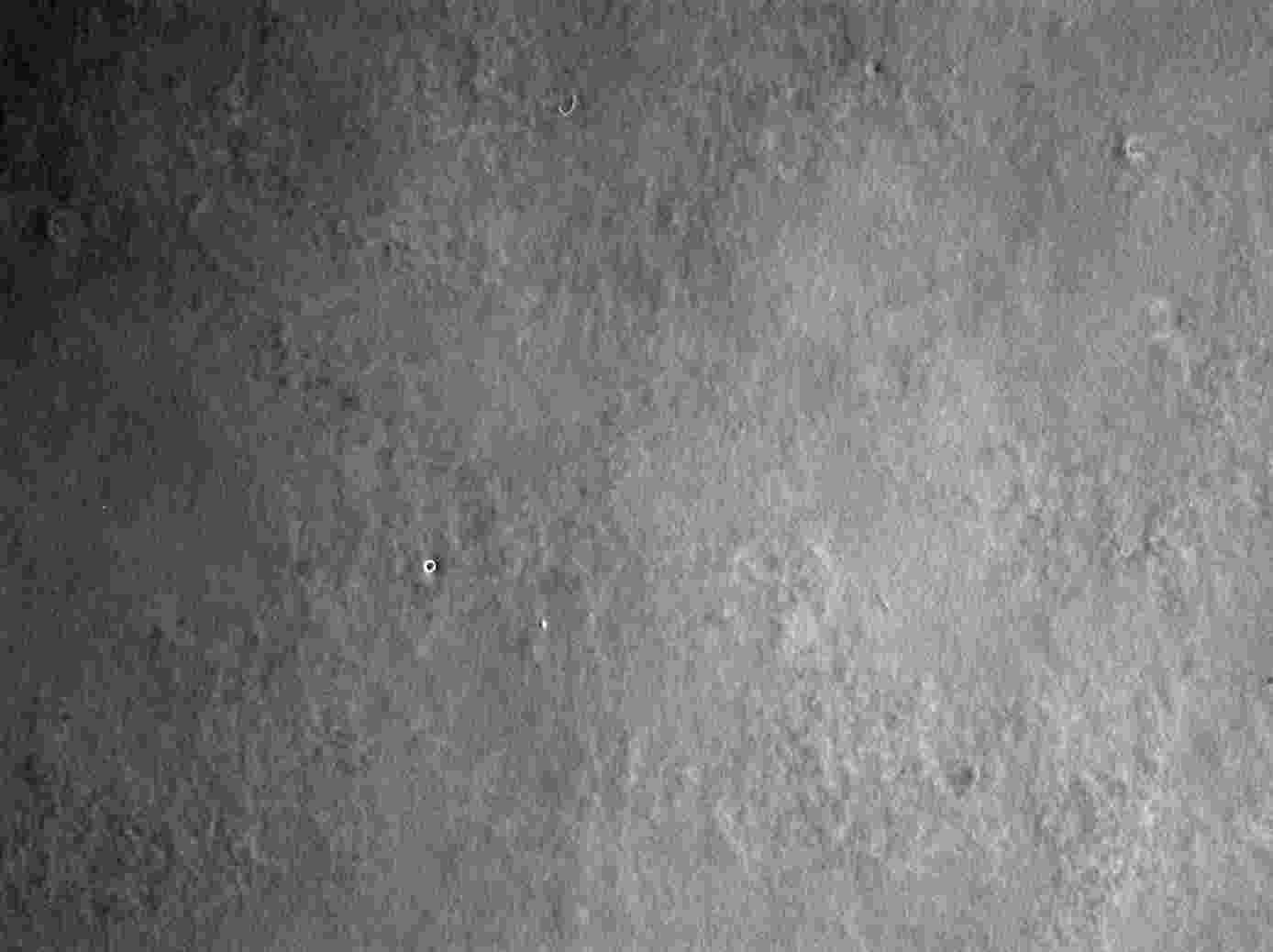

Supplement: S2 File — The raw data are presented in Raw data.zip. (ZIP) [file pone.0339611.s002.zip › Raw data/Figure 4/soft agar/day 1/3+shI-2-day1 (12).jpg]

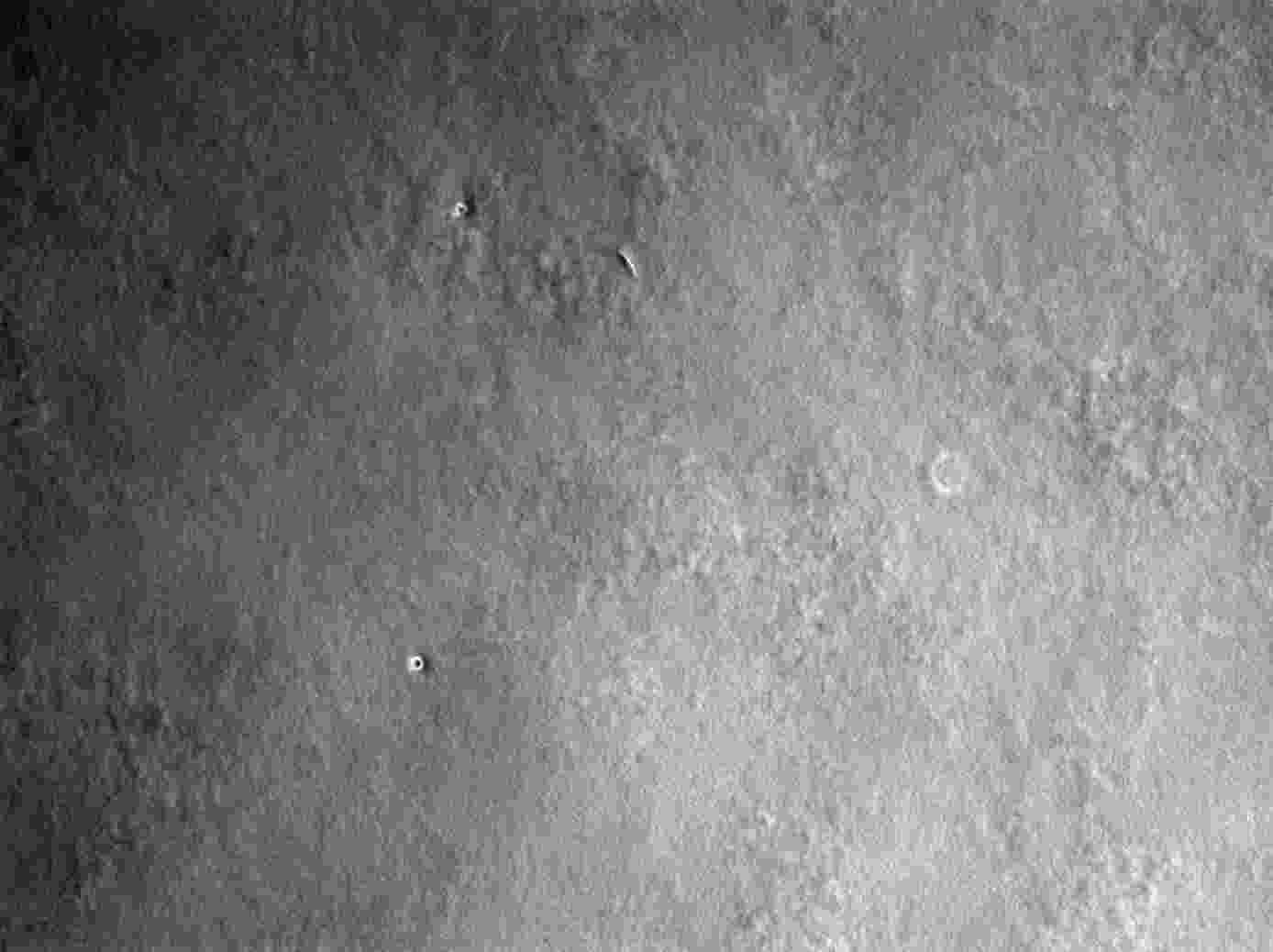

Supplement: S2 File — The raw data are presented in Raw data.zip. (ZIP) [file pone.0339611.s002.zip › Raw data/Figure 4/soft agar/day 1/3+shI-2-day1 (13).jpg]

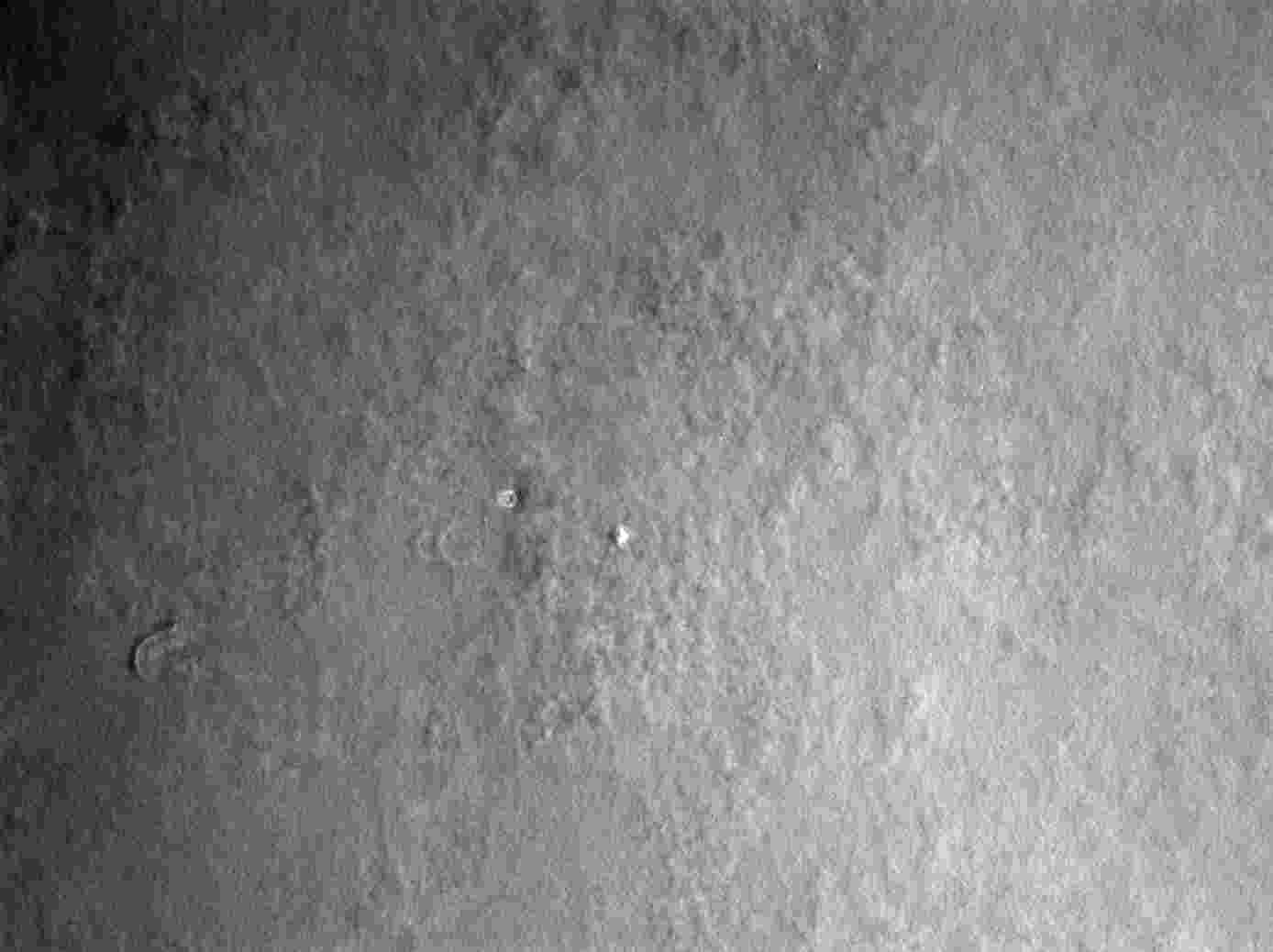

Supplement: S2 File — The raw data are presented in Raw data.zip. (ZIP) [file pone.0339611.s002.zip › Raw data/Figure 4/soft agar/day 1/3+shI-2-day1 (14).jpg]

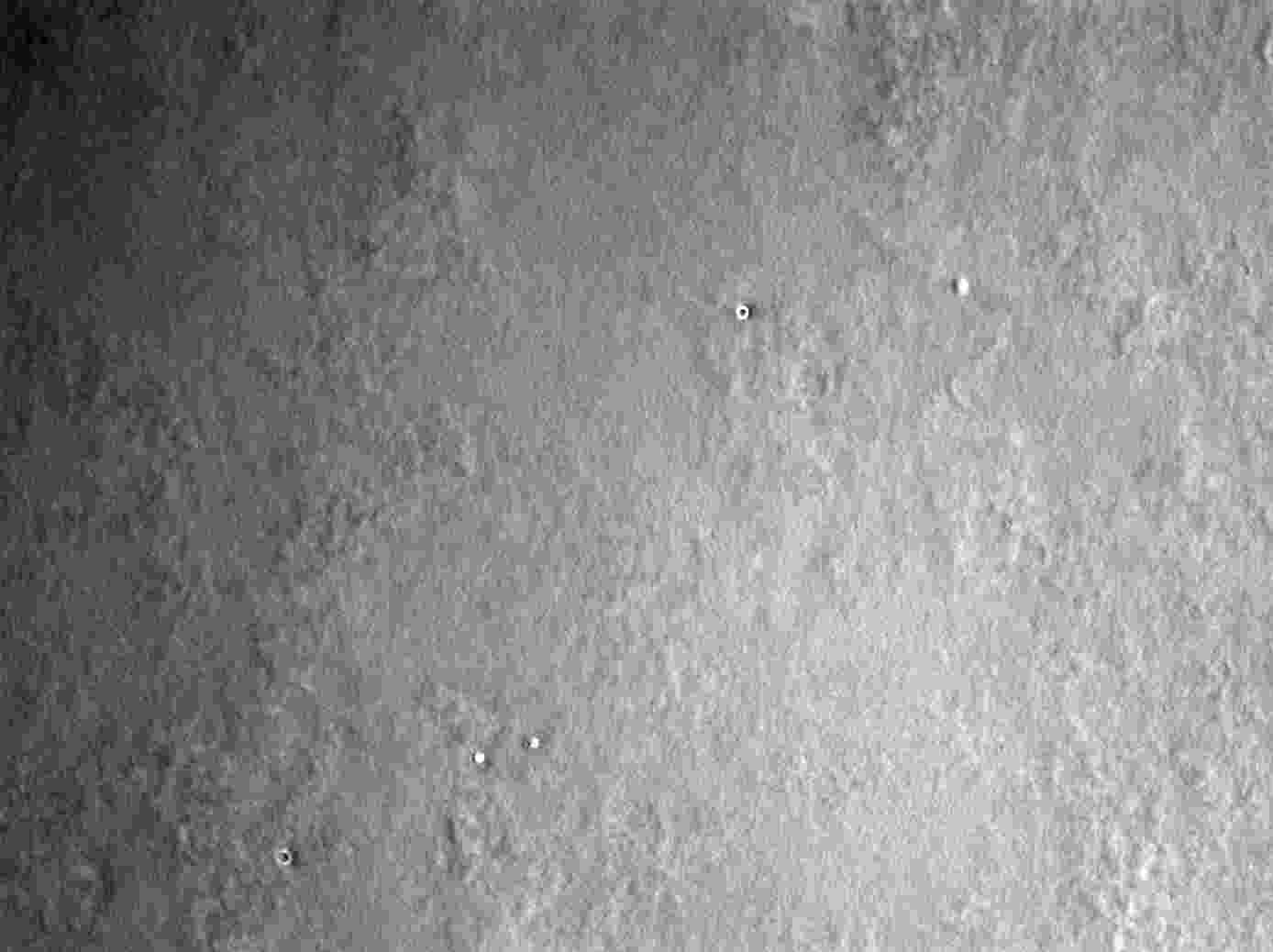

Supplement: S2 File — The raw data are presented in Raw data.zip. (ZIP) [file pone.0339611.s002.zip › Raw data/Figure 4/soft agar/day 1/3+shI-2-day1 (15).jpg]

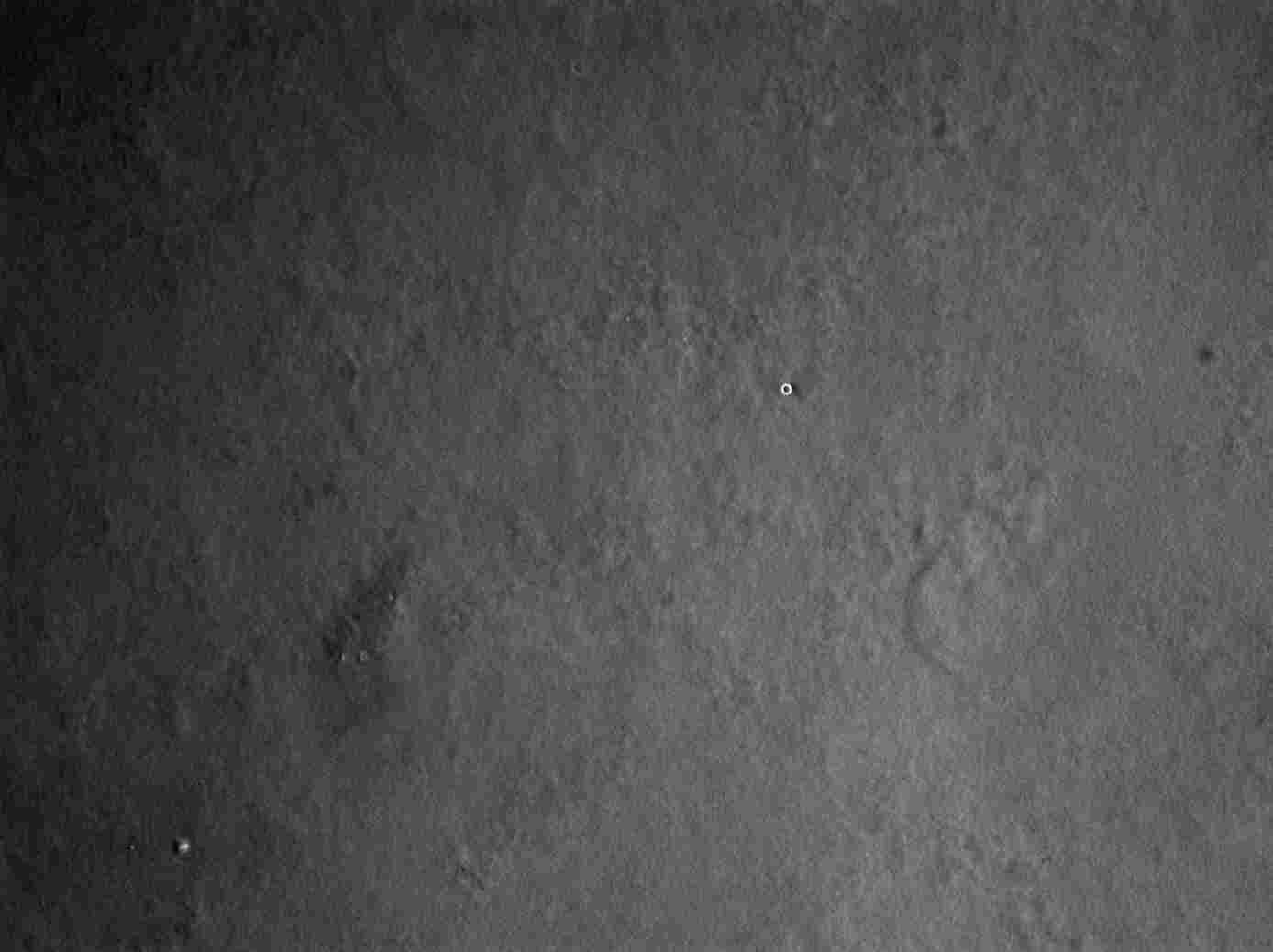

Supplement: S2 File — The raw data are presented in Raw data.zip. (ZIP) [file pone.0339611.s002.zip › Raw data/Figure 4/soft agar/day 1/3+shI-2-day1 (2).jpg]

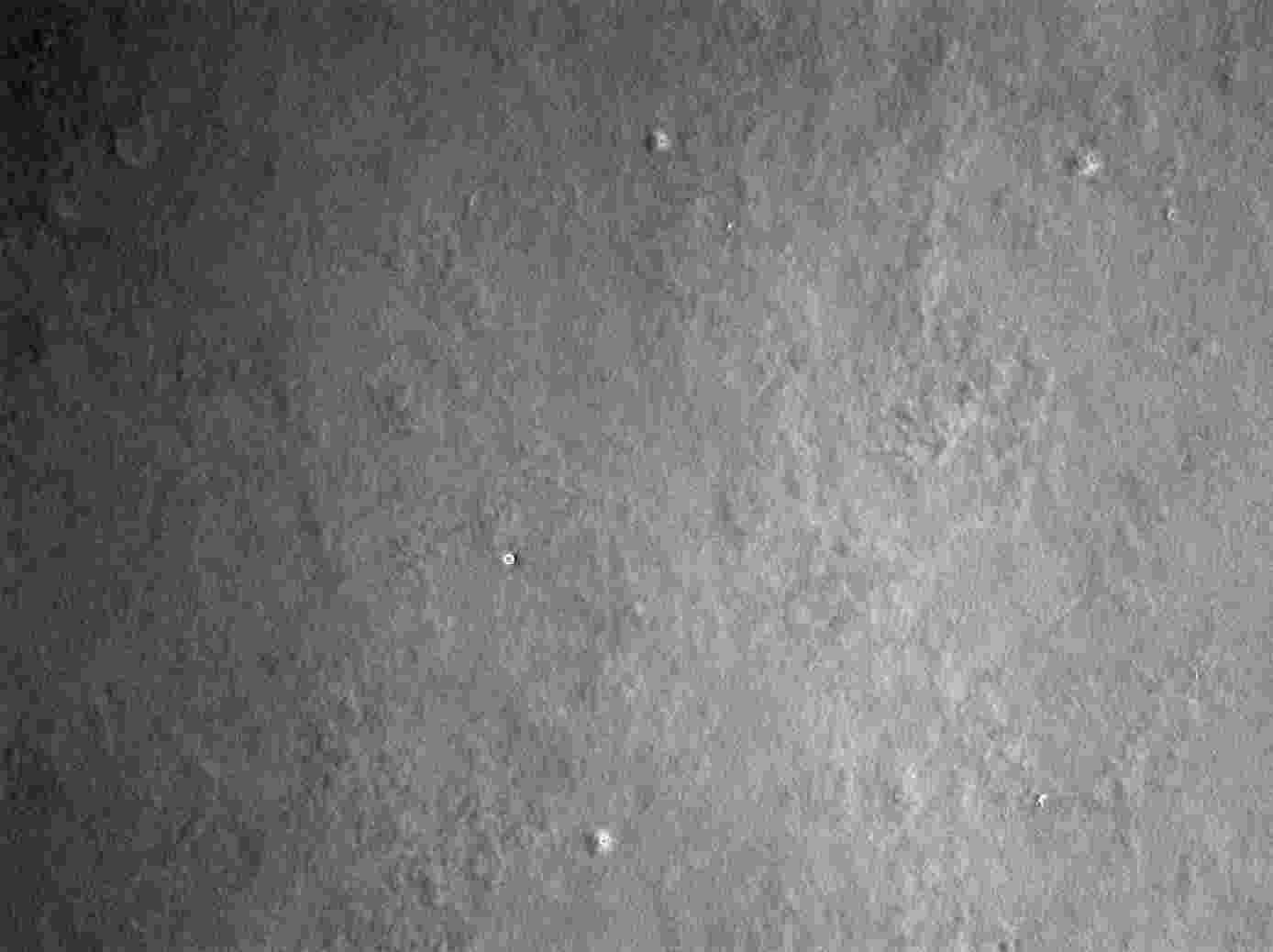

Supplement: S2 File — The raw data are presented in Raw data.zip. (ZIP) [file pone.0339611.s002.zip › Raw data/Figure 4/soft agar/day 1/3+shI-2-day1 (3).jpg]

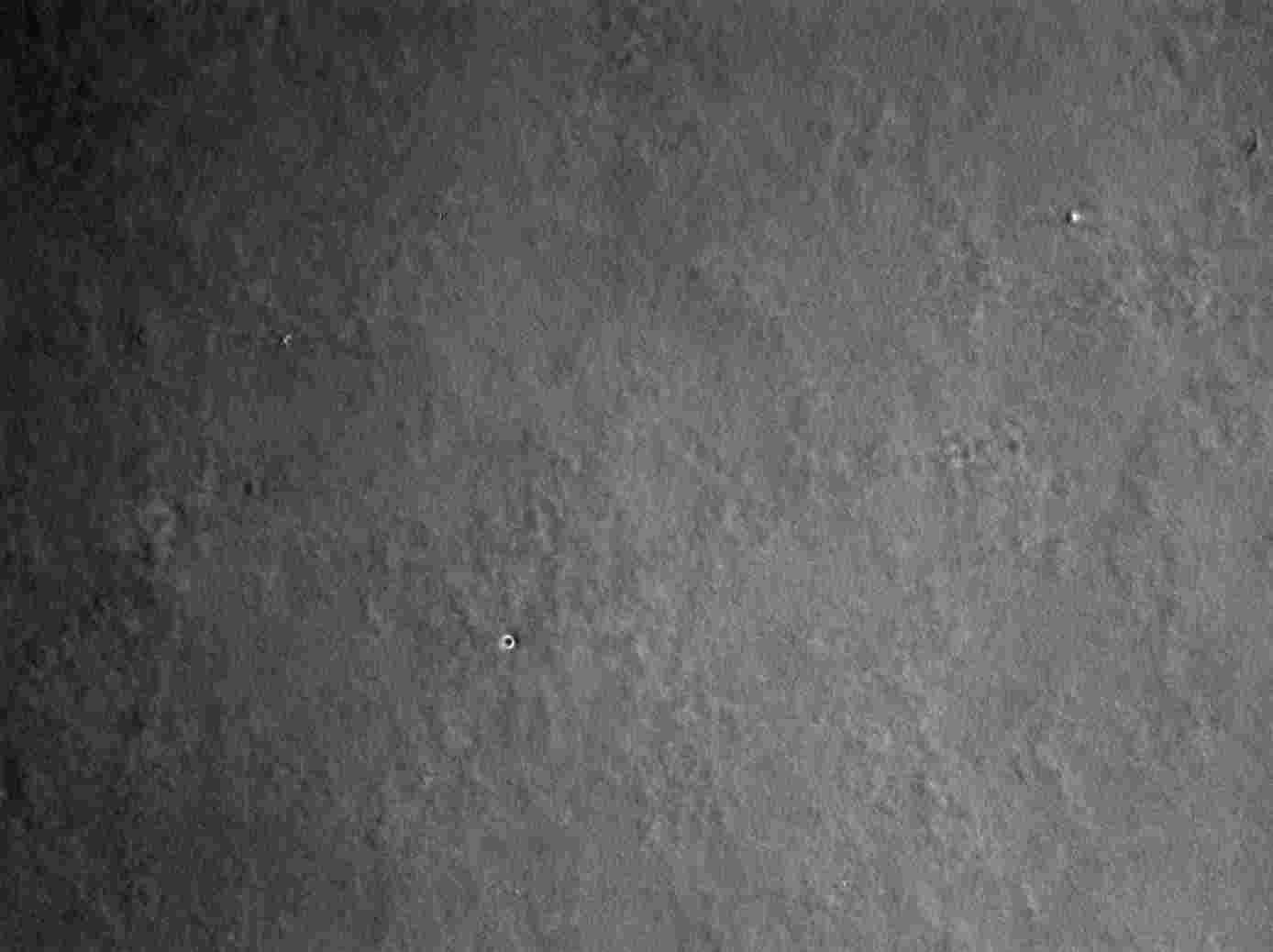

Supplement: S2 File — The raw data are presented in Raw data.zip. (ZIP) [file pone.0339611.s002.zip › Raw data/Figure 4/soft agar/day 1/3+shI-2-day1 (4).jpg]

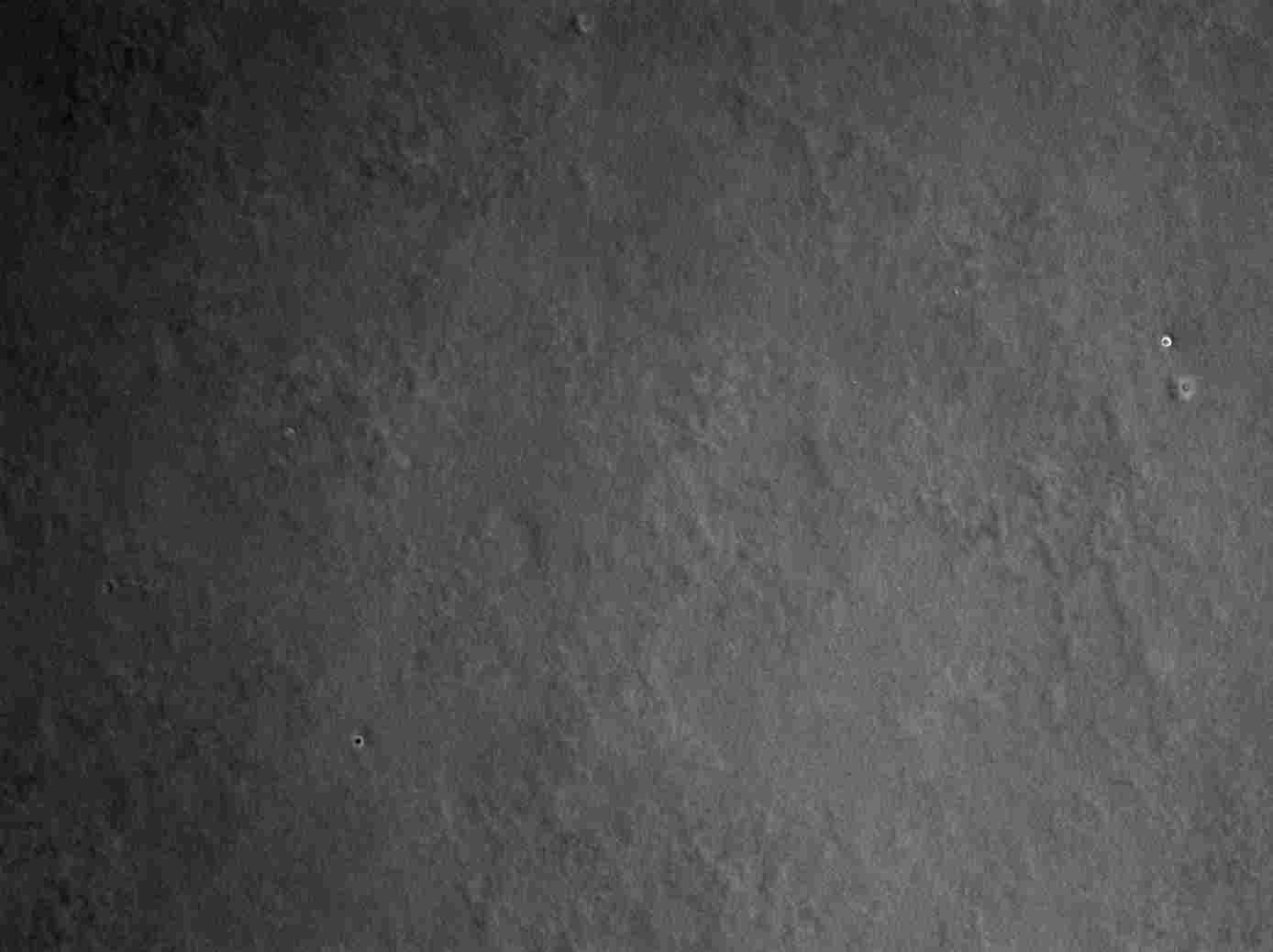

Supplement: S2 File — The raw data are presented in Raw data.zip. (ZIP) [file pone.0339611.s002.zip › Raw data/Figure 4/soft agar/day 1/3+shI-2-day1 (5).jpg]

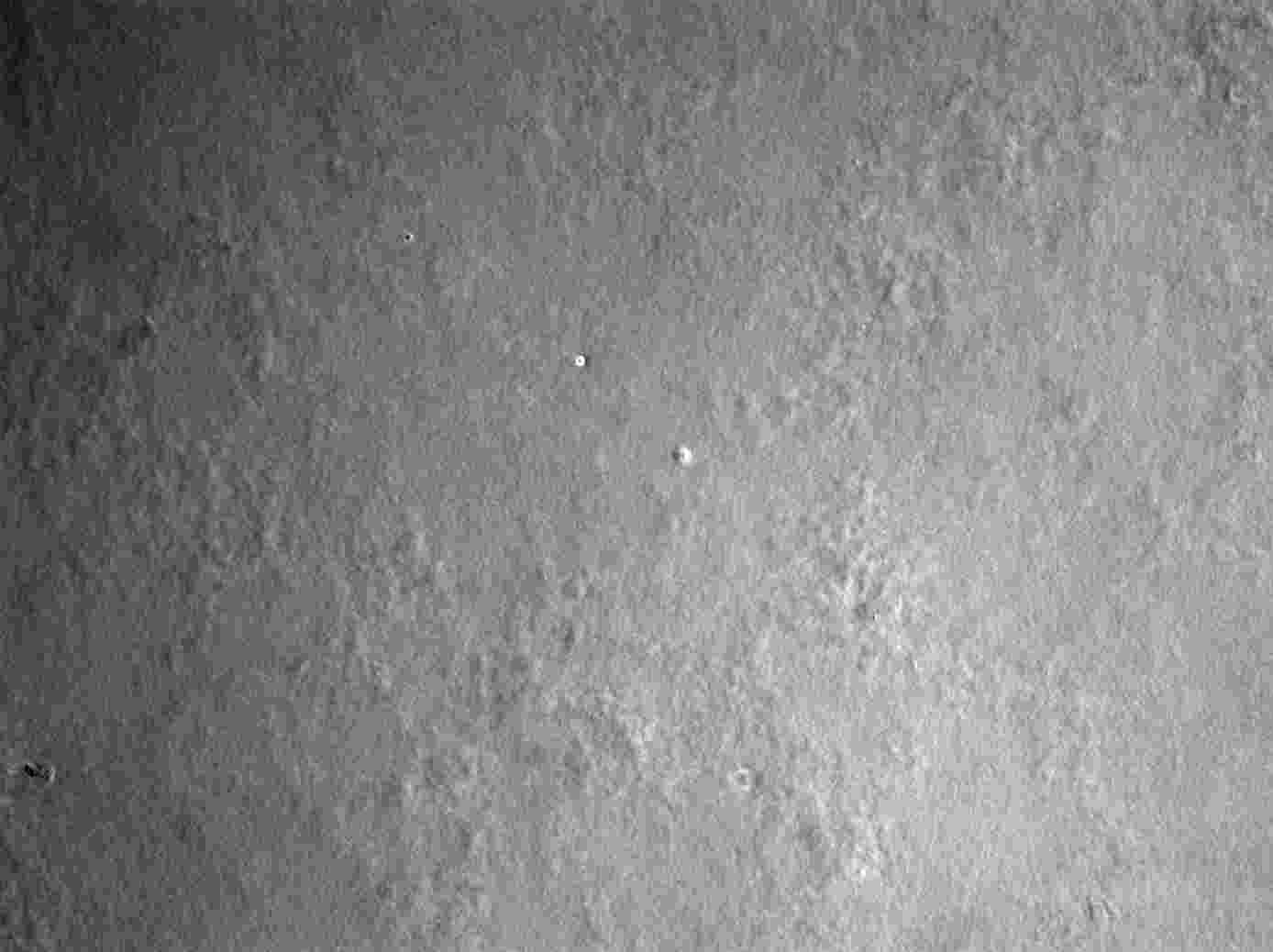

Supplement: S2 File — The raw data are presented in Raw data.zip. (ZIP) [file pone.0339611.s002.zip › Raw data/Figure 4/soft agar/day 1/3+shI-2-day1 (6).jpg]

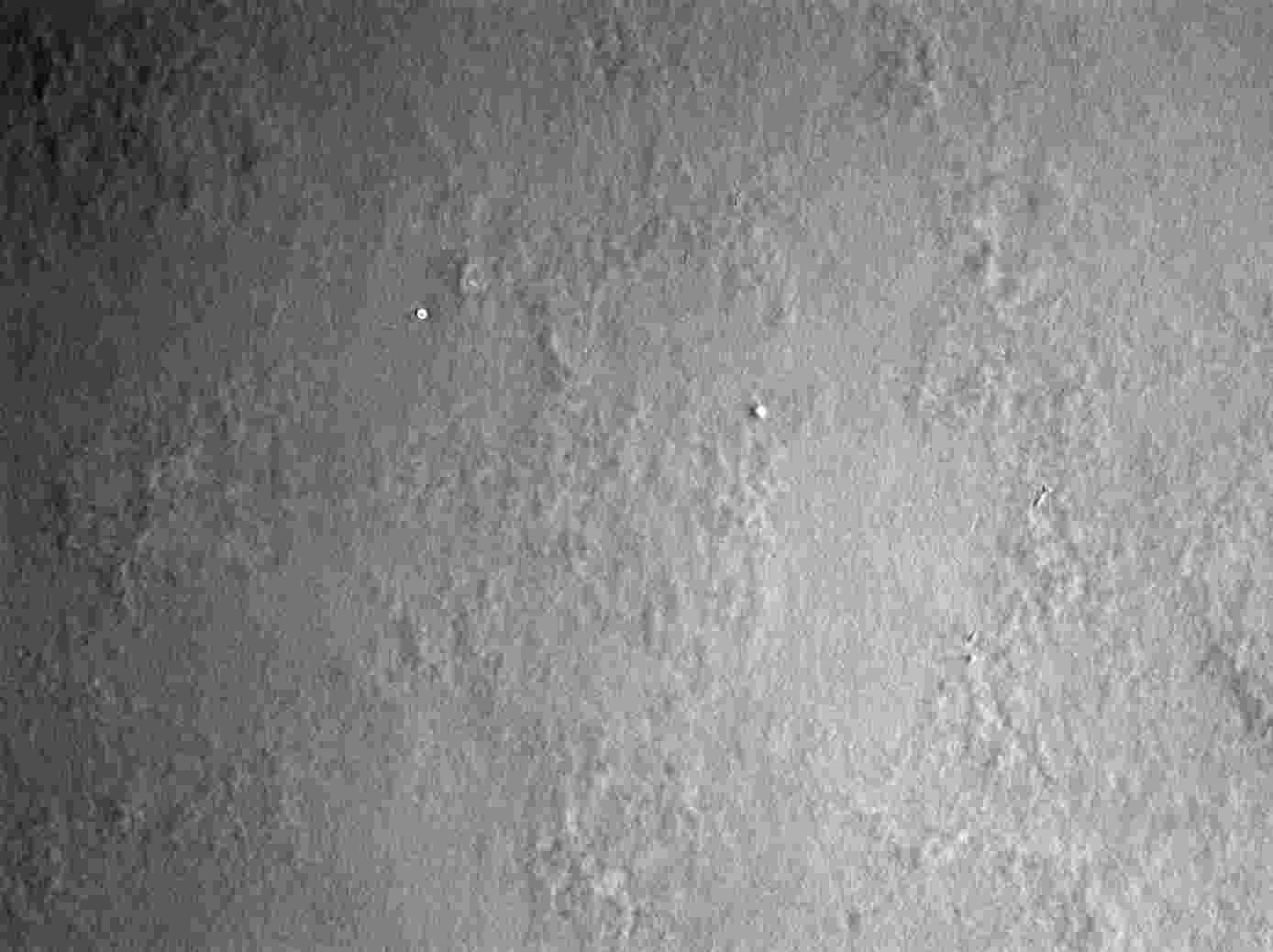

Supplement: S2 File — The raw data are presented in Raw data.zip. (ZIP) [file pone.0339611.s002.zip › Raw data/Figure 4/soft agar/day 1/3+shI-2-day1 (7).jpg]

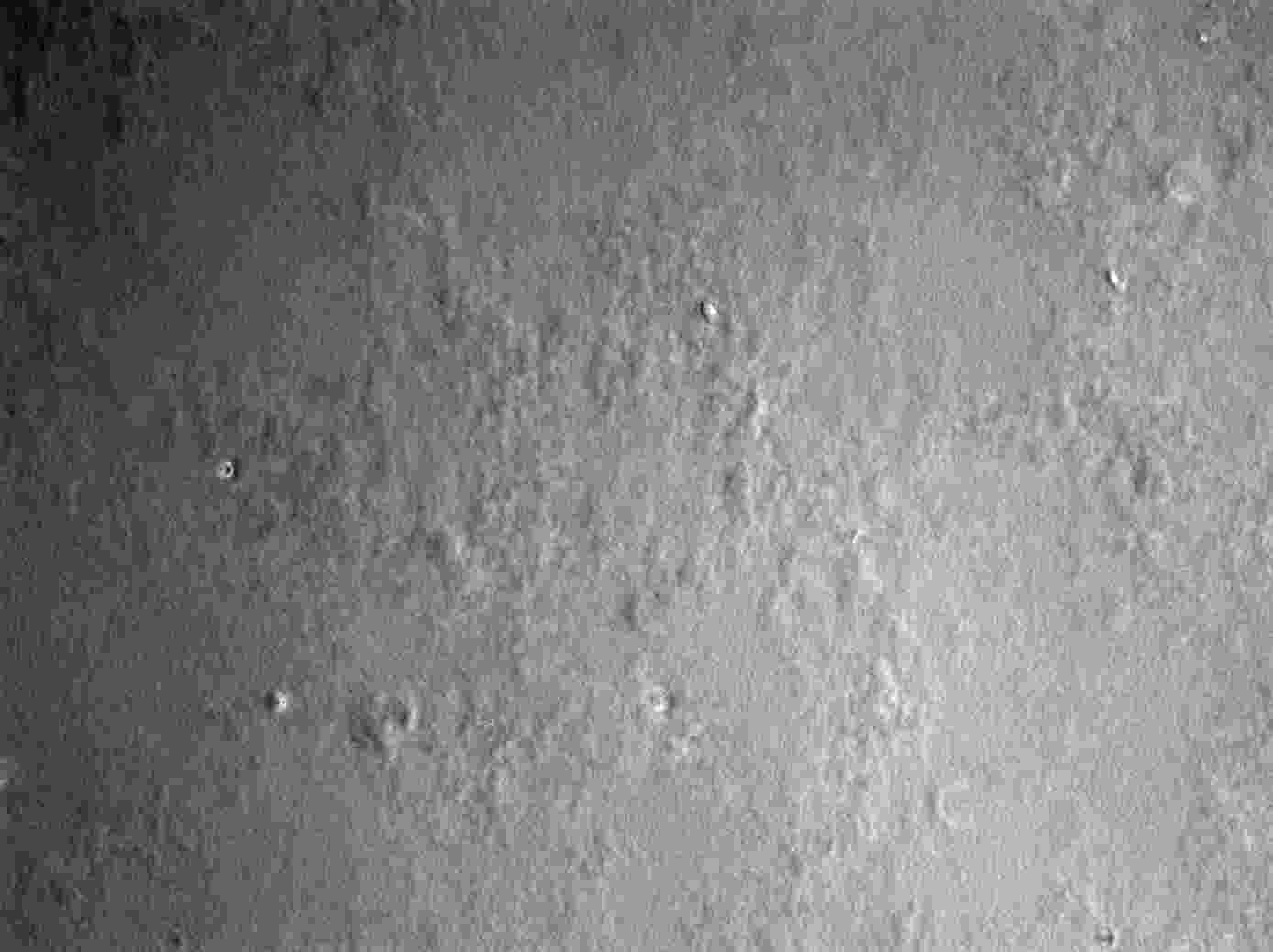

Supplement: S2 File — The raw data are presented in Raw data.zip. (ZIP) [file pone.0339611.s002.zip › Raw data/Figure 4/soft agar/day 1/3+shI-2-day1 (8).jpg]

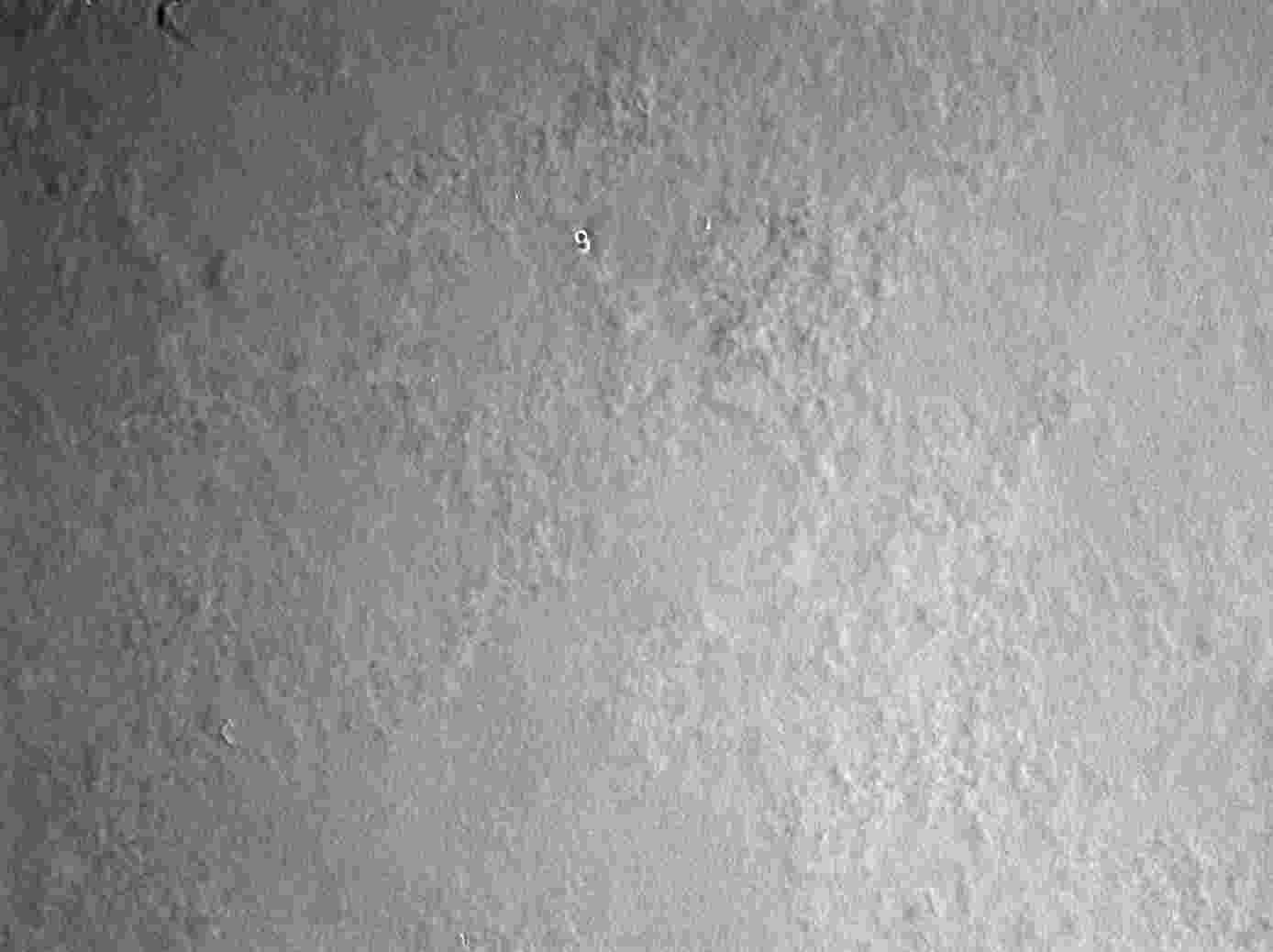

Supplement: S2 File — The raw data are presented in Raw data.zip. (ZIP) [file pone.0339611.s002.zip › Raw data/Figure 4/soft agar/day 1/3+shI-2-day1 (9).jpg]

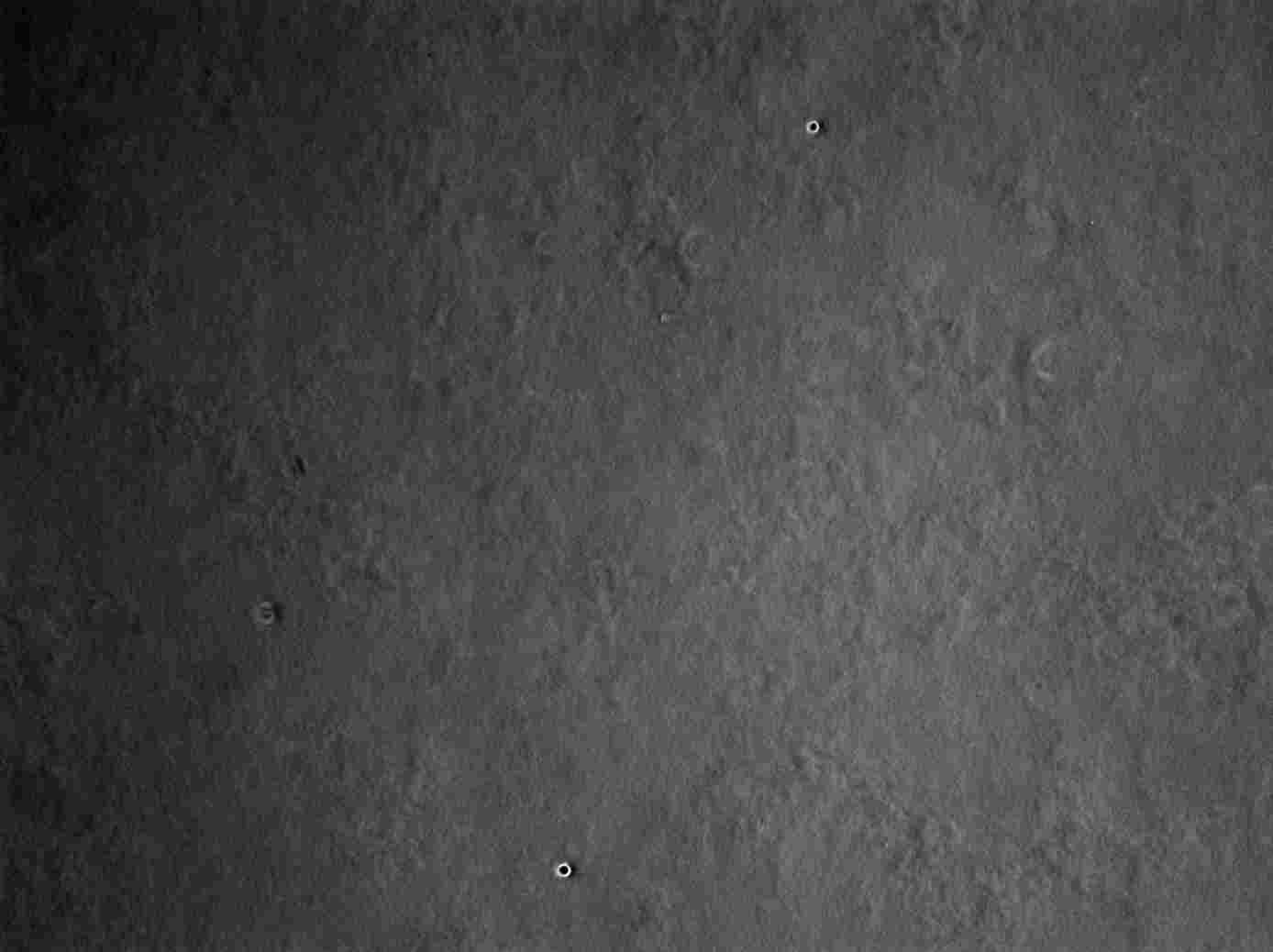

Supplement: S2 File — The raw data are presented in Raw data.zip. (ZIP) [file pone.0339611.s002.zip › Raw data/Figure 4/soft agar/day 1/3+shI-2-day1.jpg]

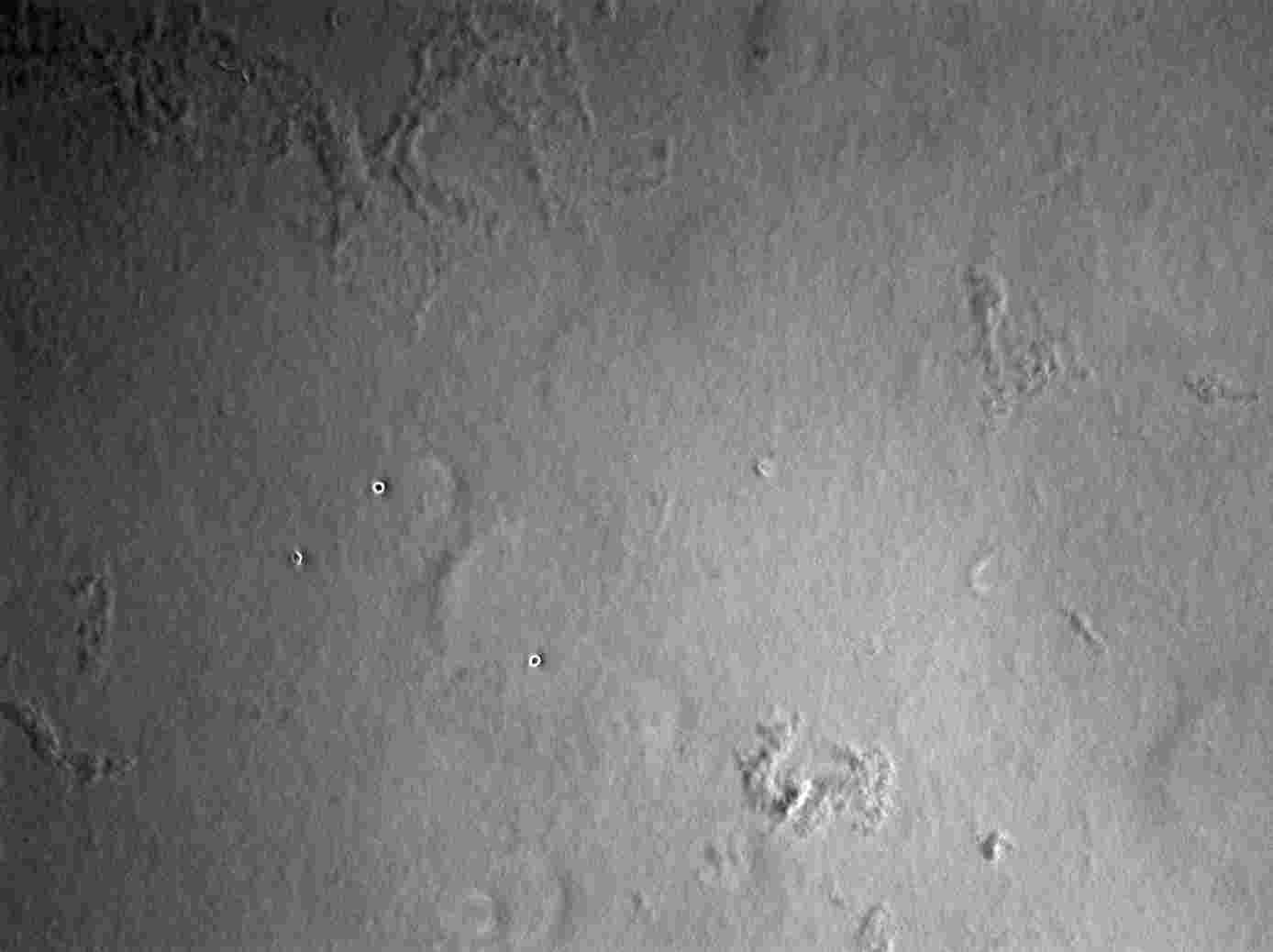

Supplement: S2 File — The raw data are presented in Raw data.zip. (ZIP) [file pone.0339611.s002.zip › Raw data/Figure 4/soft agar/day 1/3+shscr-day1 (10).jpg]

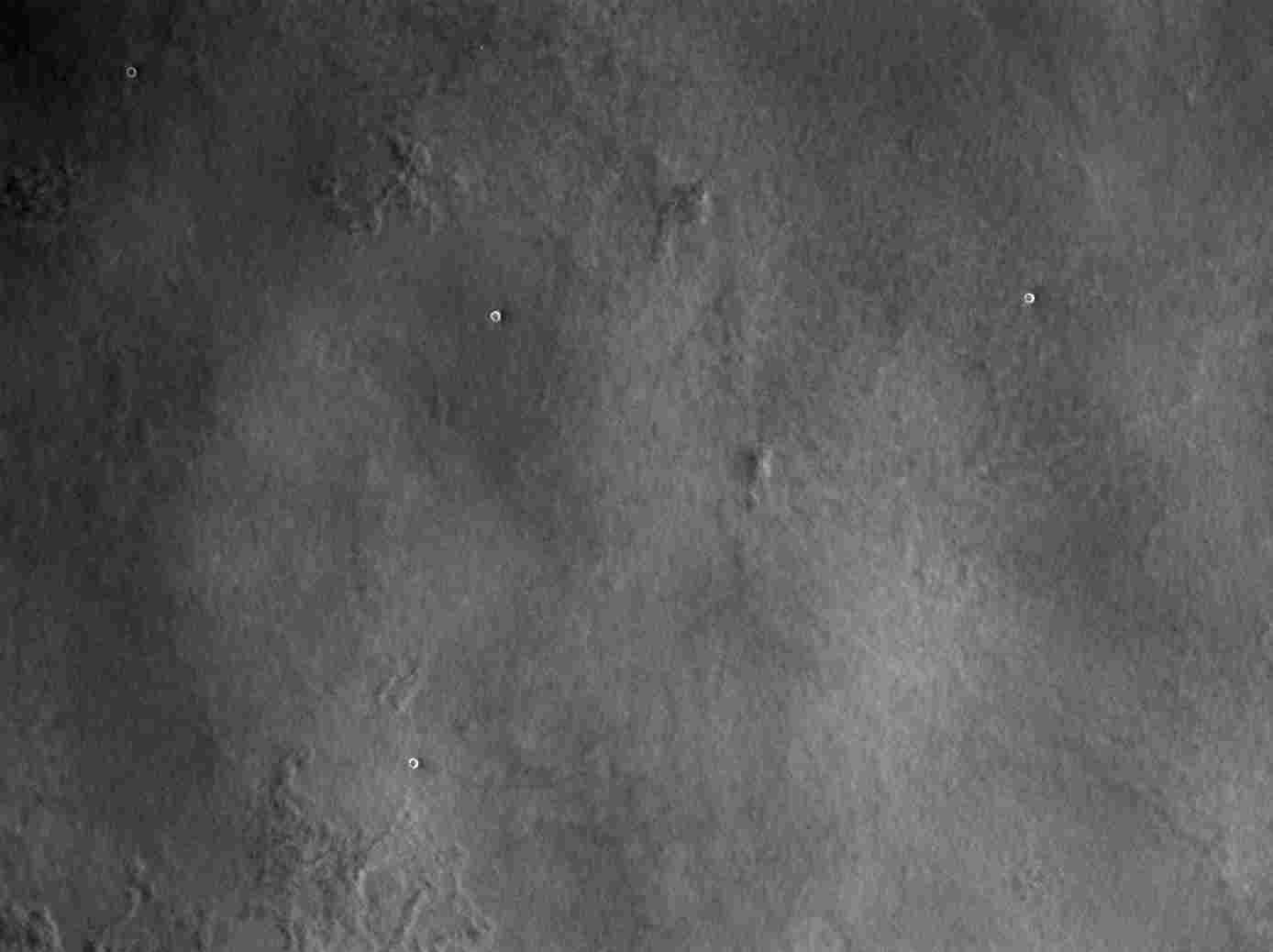

Supplement: S2 File — The raw data are presented in Raw data.zip. (ZIP) [file pone.0339611.s002.zip › Raw data/Figure 4/soft agar/day 1/3+shscr-day1 (11).jpg]

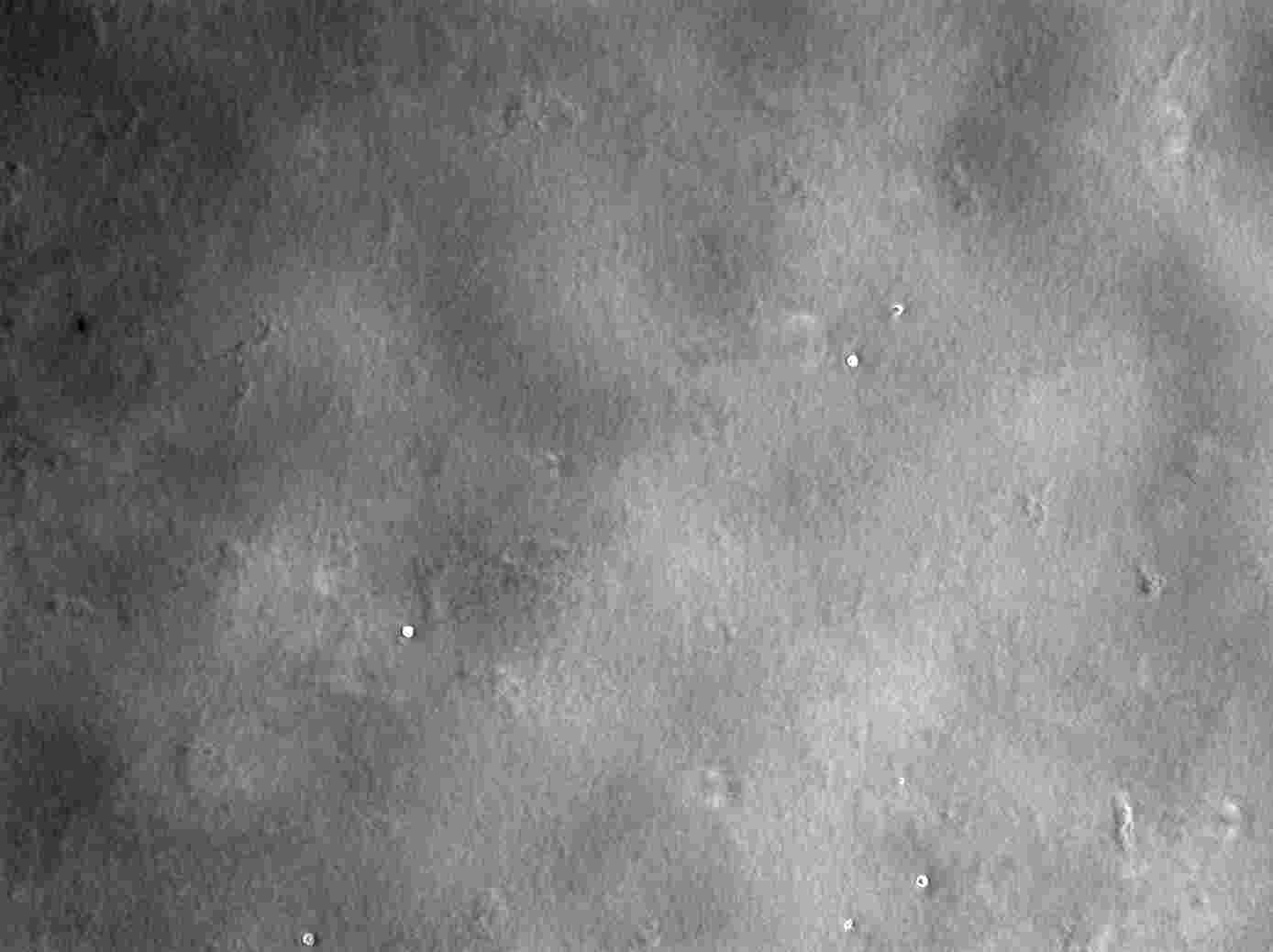

Supplement: S2 File — The raw data are presented in Raw data.zip. (ZIP) [file pone.0339611.s002.zip › Raw data/Figure 4/soft agar/day 1/3+shscr-day1 (12).jpg]

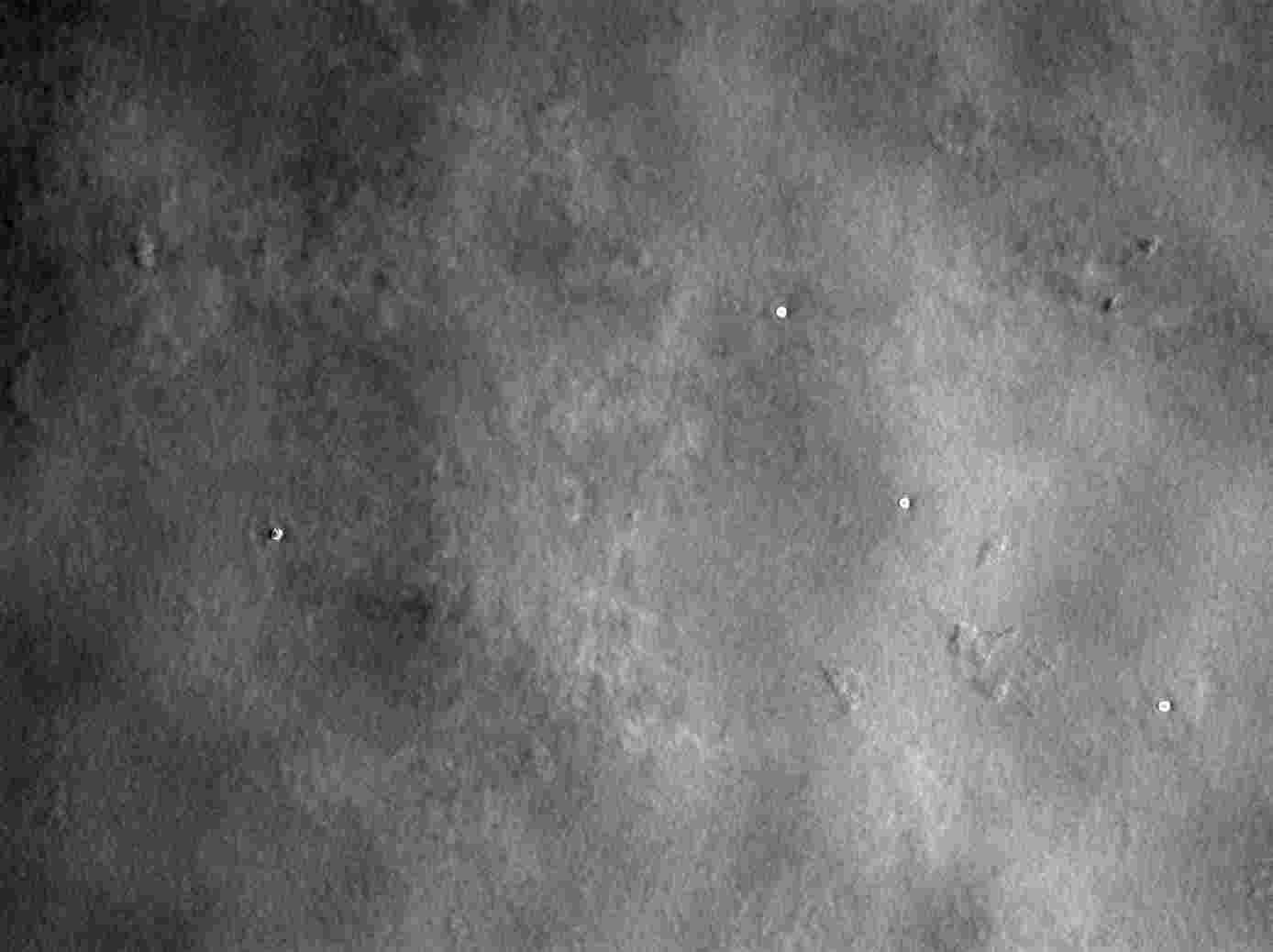

Supplement: S2 File — The raw data are presented in Raw data.zip. (ZIP) [file pone.0339611.s002.zip › Raw data/Figure 4/soft agar/day 1/3+shscr-day1 (13).jpg]

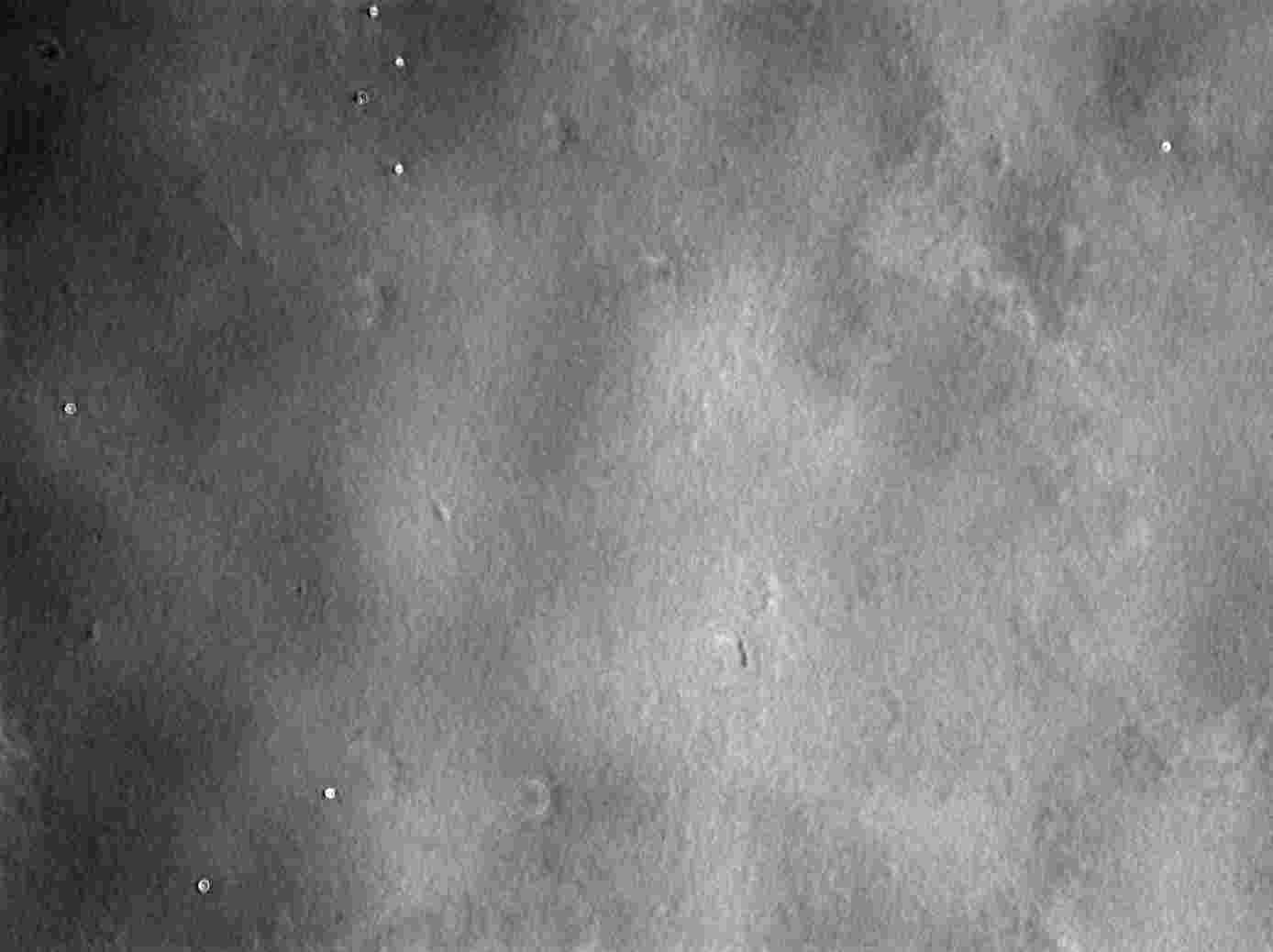

Supplement: S2 File — The raw data are presented in Raw data.zip. (ZIP) [file pone.0339611.s002.zip › Raw data/Figure 4/soft agar/day 1/3+shscr-day1 (14).jpg]

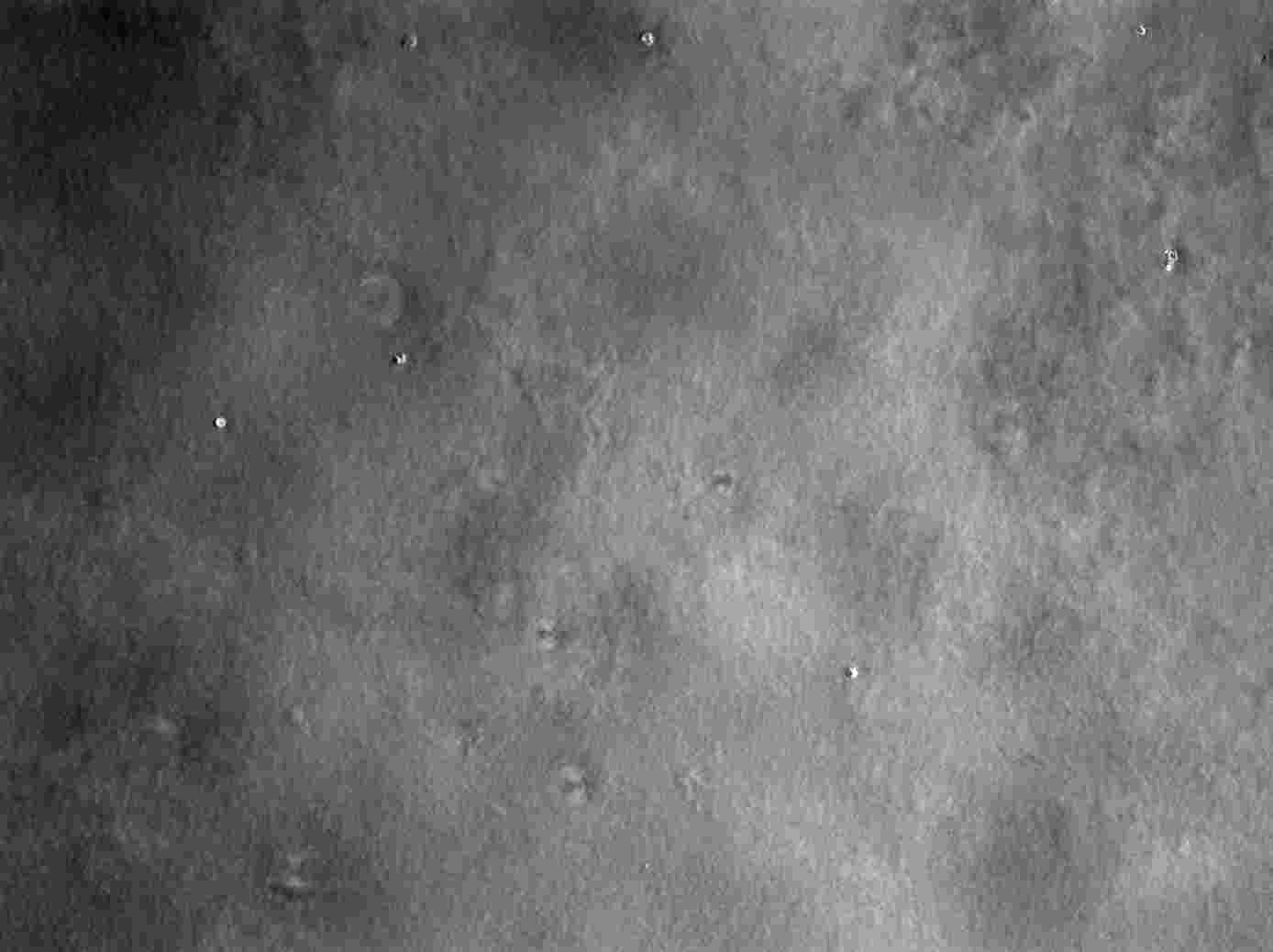

Supplement: S2 File — The raw data are presented in Raw data.zip. (ZIP) [file pone.0339611.s002.zip › Raw data/Figure 4/soft agar/day 1/3+shscr-day1 (15).jpg]

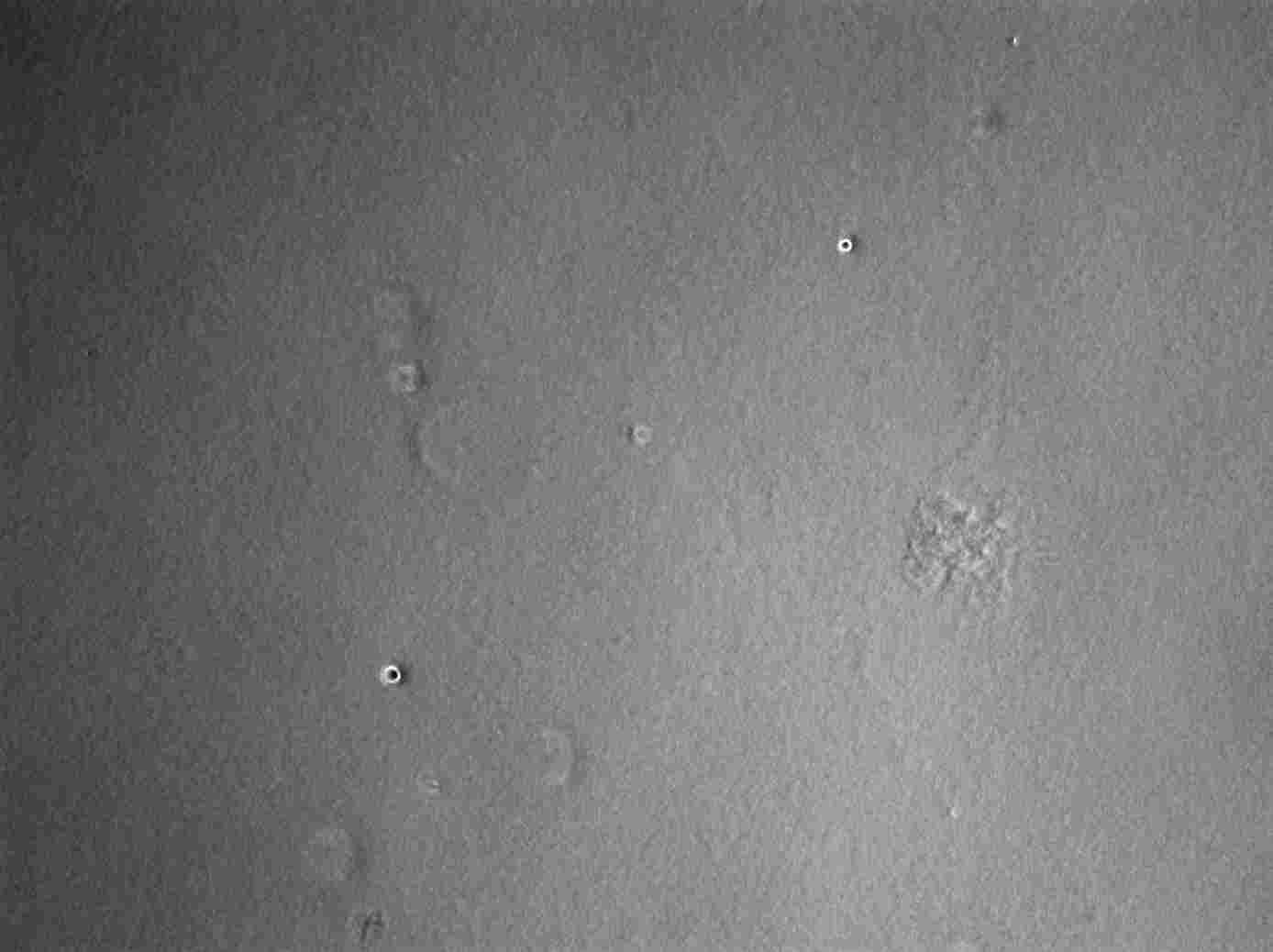

Supplement: S2 File — The raw data are presented in Raw data.zip. (ZIP) [file pone.0339611.s002.zip › Raw data/Figure 4/soft agar/day 1/3+shscr-day1 (2).jpg]

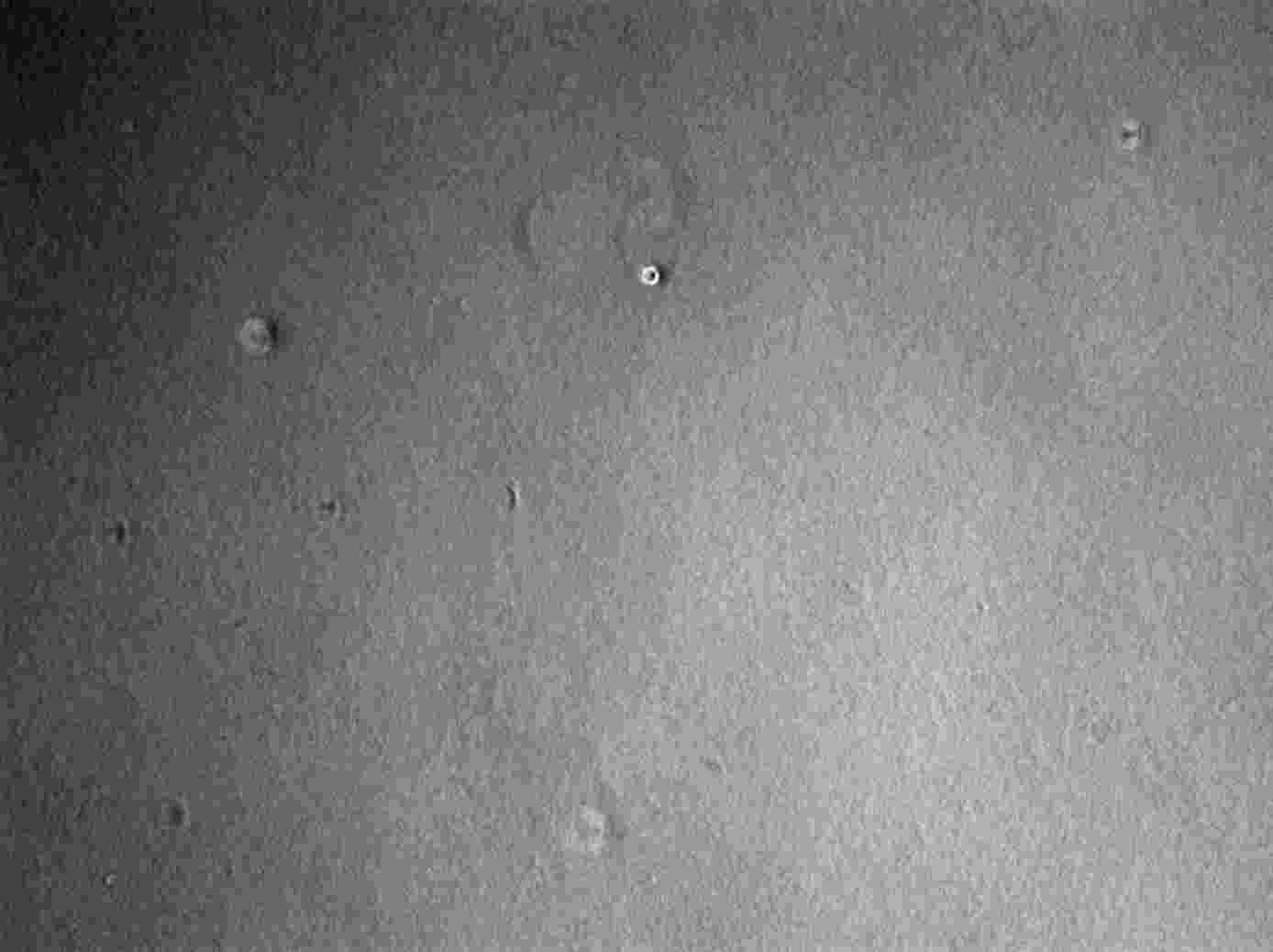

Supplement: S2 File — The raw data are presented in Raw data.zip. (ZIP) [file pone.0339611.s002.zip › Raw data/Figure 4/soft agar/day 1/3+shscr-day1 (3).jpg]

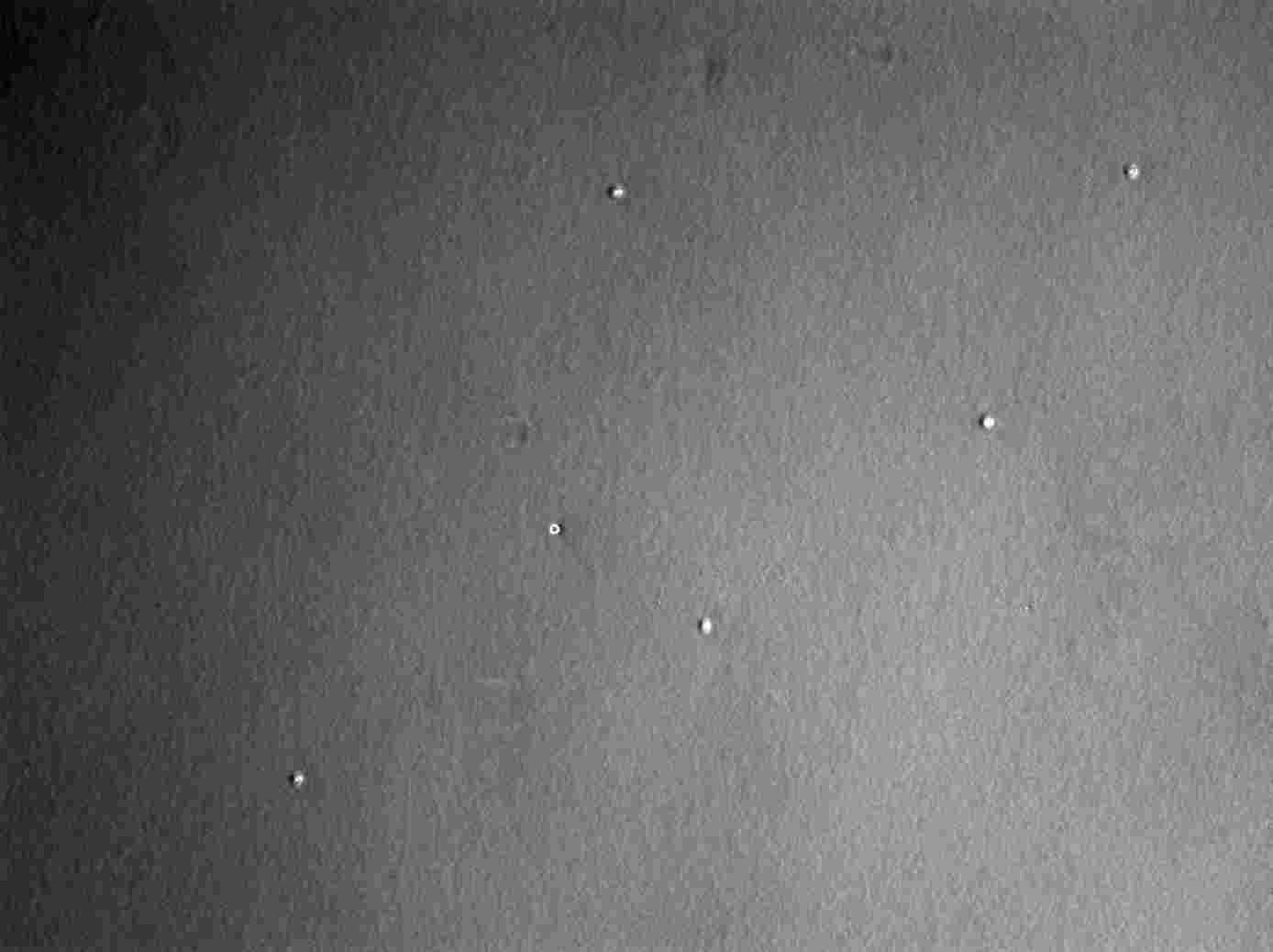

Supplement: S2 File — The raw data are presented in Raw data.zip. (ZIP) [file pone.0339611.s002.zip › Raw data/Figure 4/soft agar/day 1/3+shscr-day1 (4).jpg]

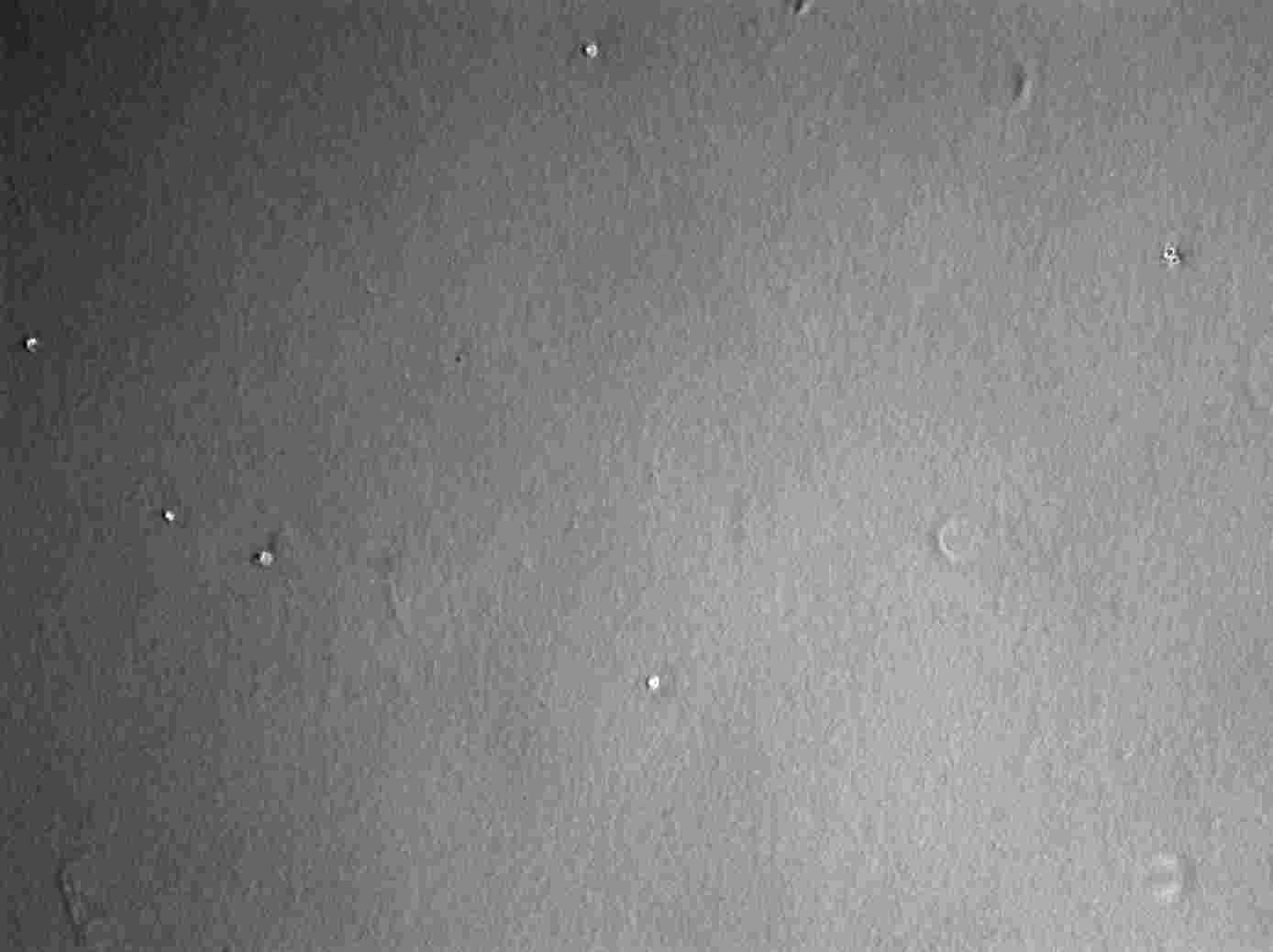

Supplement: S2 File — The raw data are presented in Raw data.zip. (ZIP) [file pone.0339611.s002.zip › Raw data/Figure 4/soft agar/day 1/3+shscr-day1 (5).jpg]

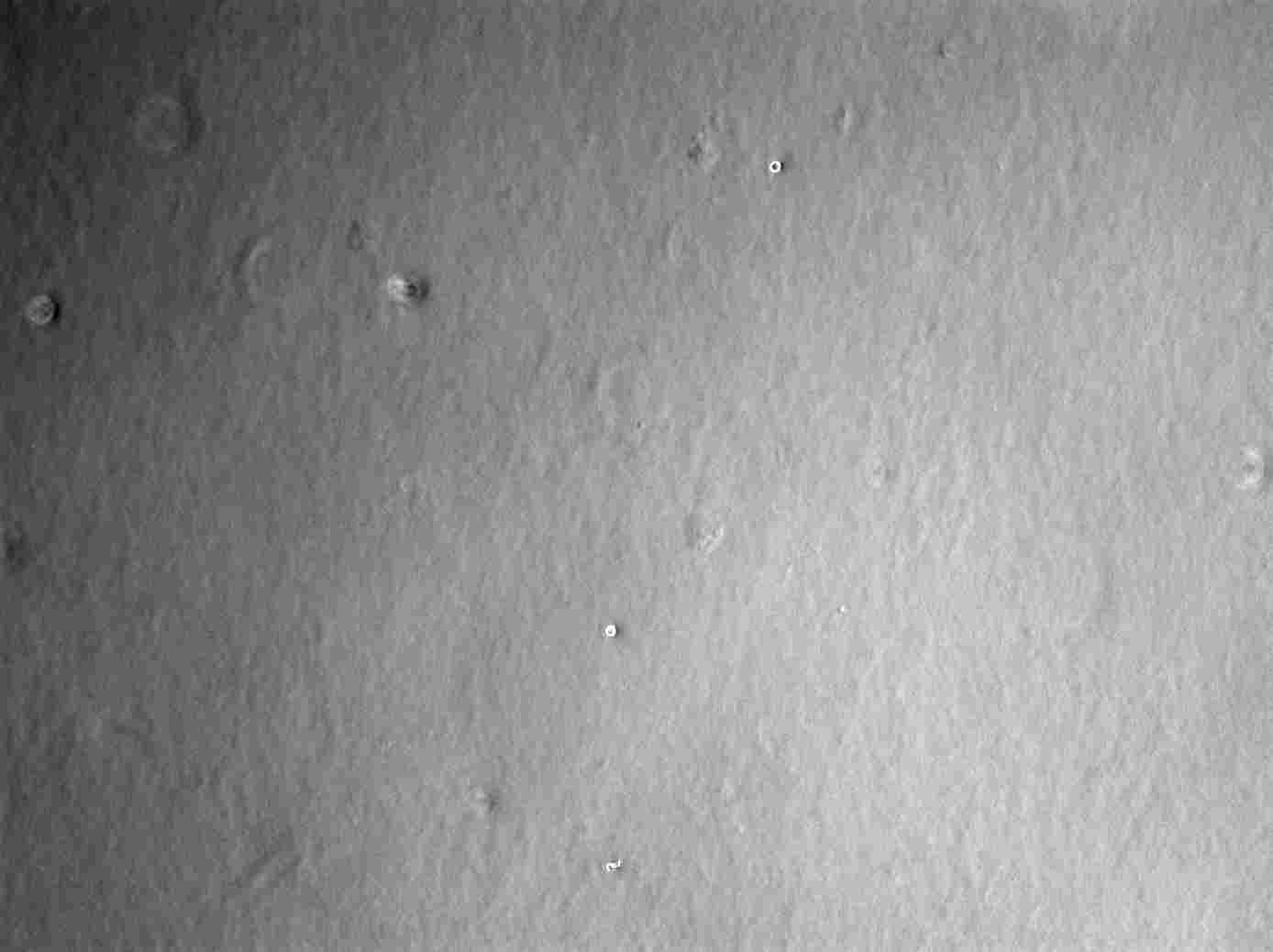

Supplement: S2 File — The raw data are presented in Raw data.zip. (ZIP) [file pone.0339611.s002.zip › Raw data/Figure 4/soft agar/day 1/3+shscr-day1 (6).jpg]

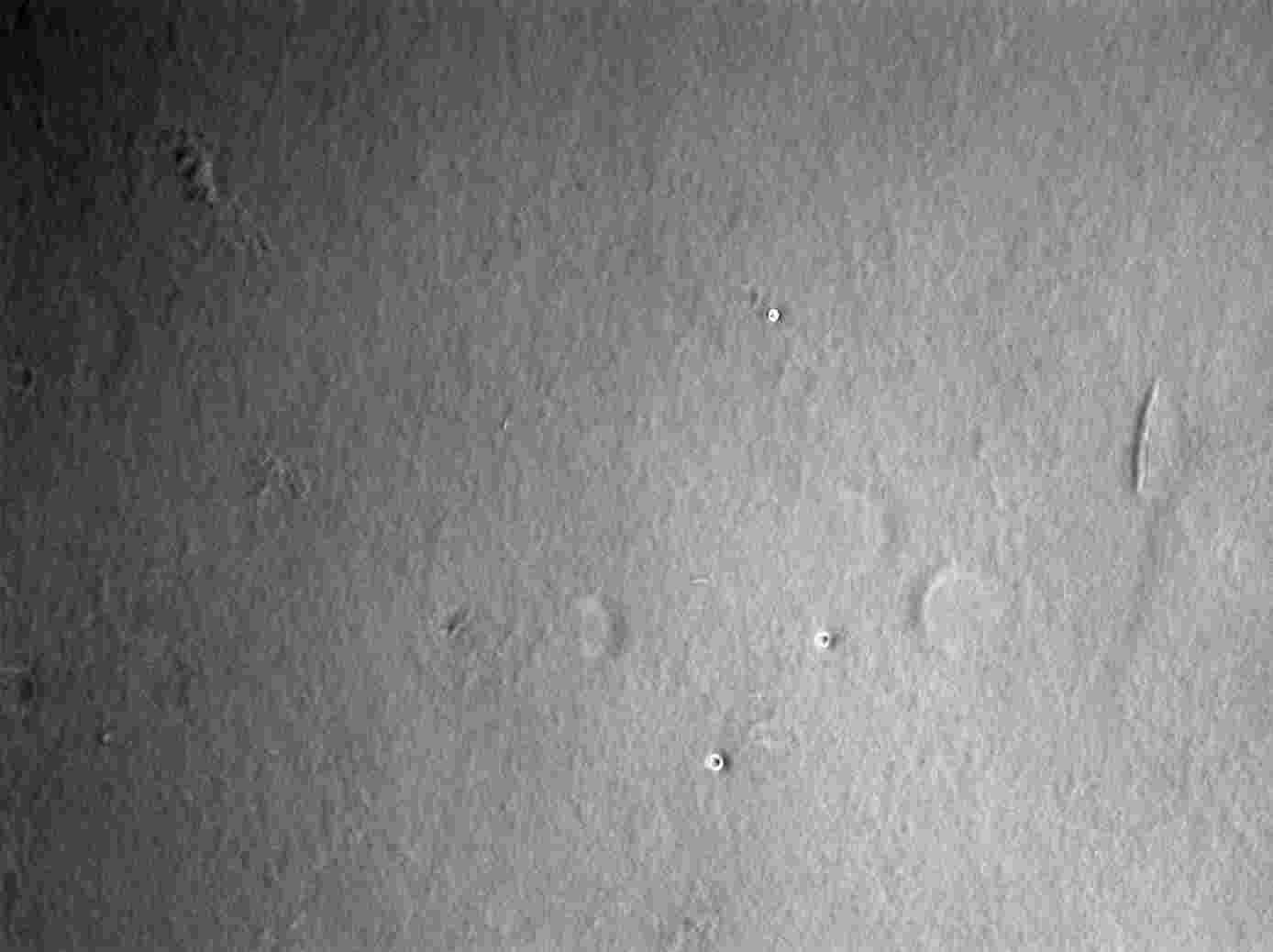

Supplement: S2 File — The raw data are presented in Raw data.zip. (ZIP) [file pone.0339611.s002.zip › Raw data/Figure 4/soft agar/day 1/3+shscr-day1 (7).jpg]

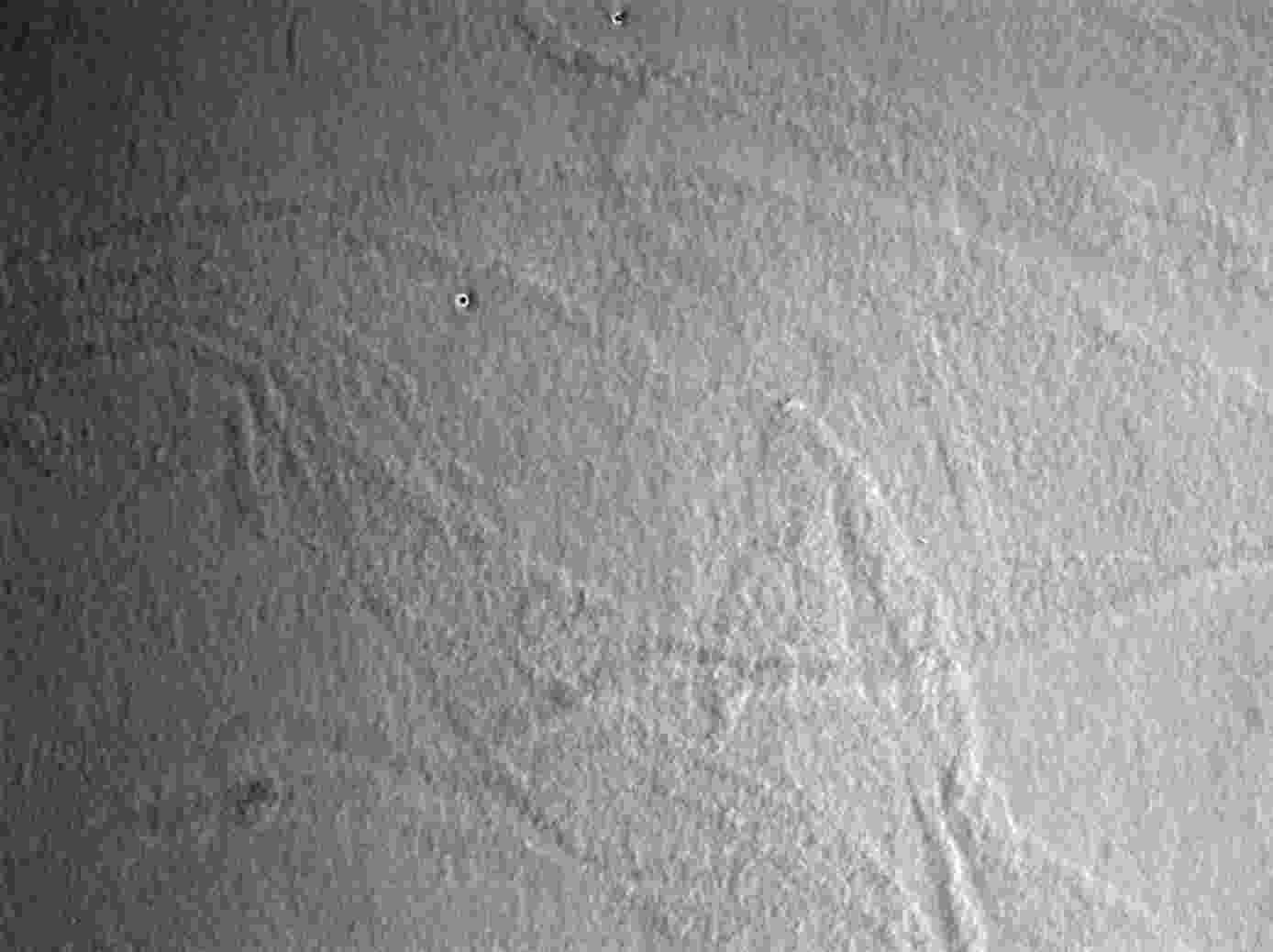

Supplement: S2 File — The raw data are presented in Raw data.zip. (ZIP) [file pone.0339611.s002.zip › Raw data/Figure 4/soft agar/day 1/3+shscr-day1 (8).jpg]

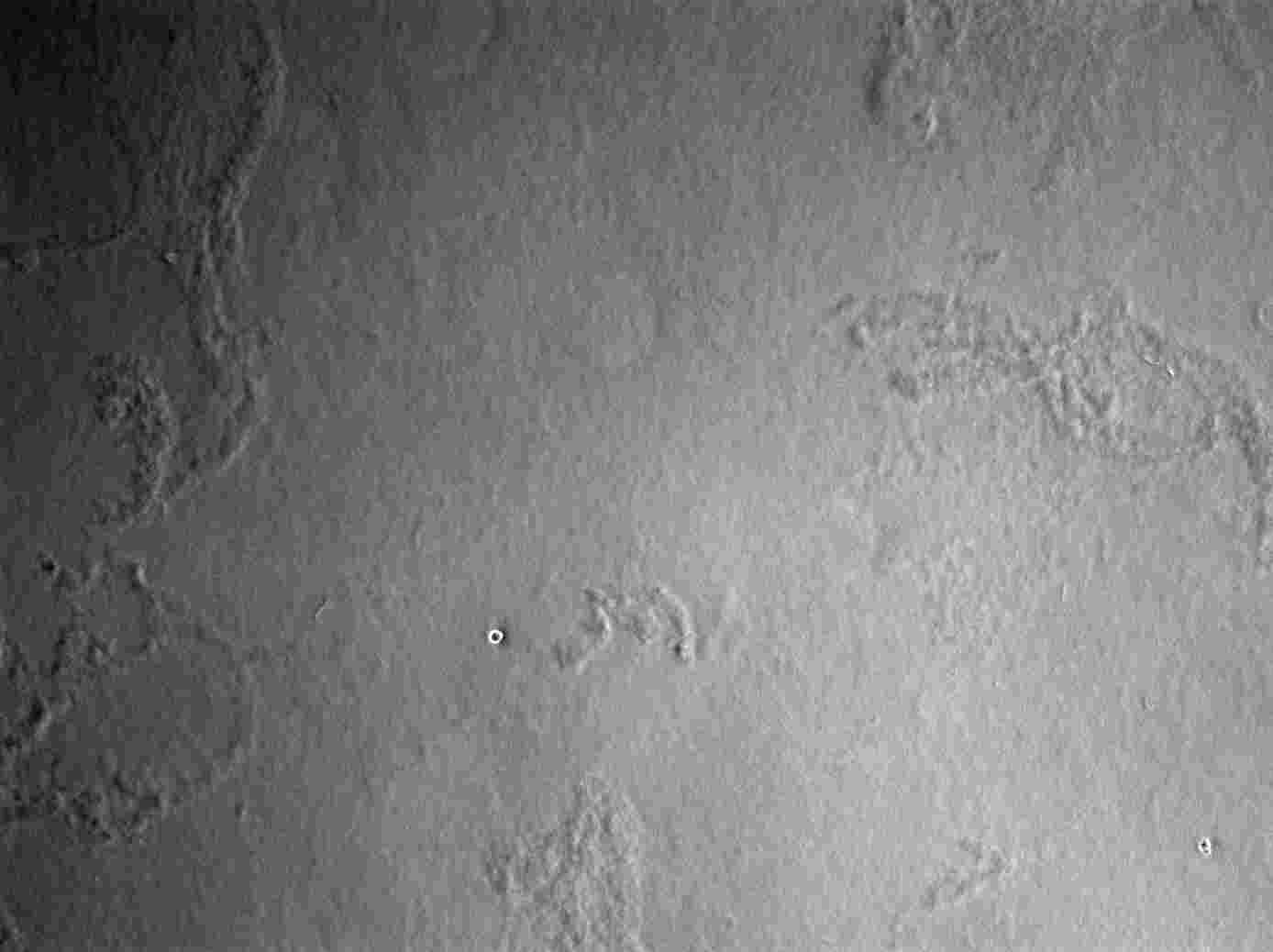

Supplement: S2 File — The raw data are presented in Raw data.zip. (ZIP) [file pone.0339611.s002.zip › Raw data/Figure 4/soft agar/day 1/3+shscr-day1 (9).jpg]

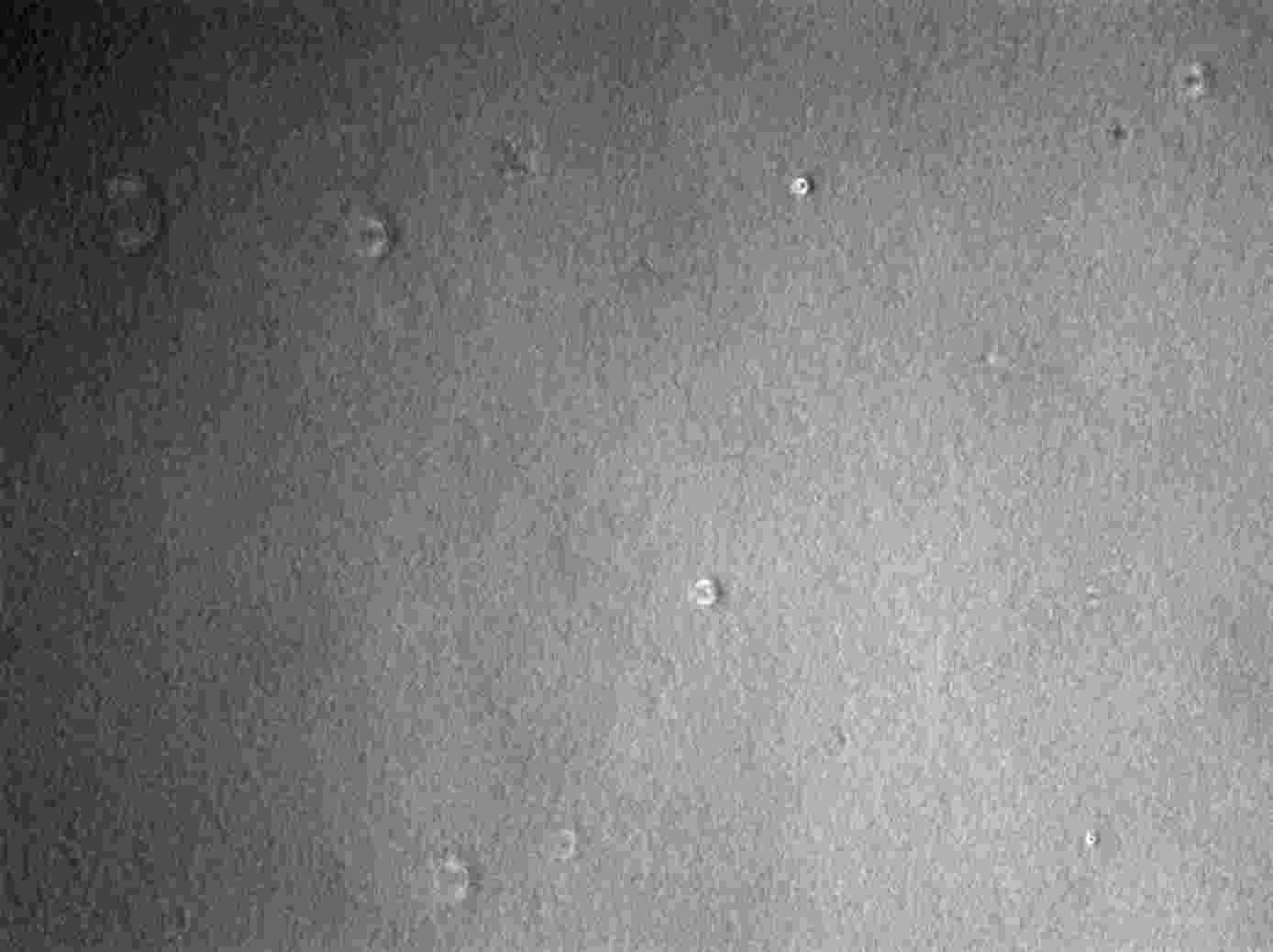

Supplement: S2 File — The raw data are presented in Raw data.zip. (ZIP) [file pone.0339611.s002.zip › Raw data/Figure 4/soft agar/day 1/3+shscr-day1.jpg]

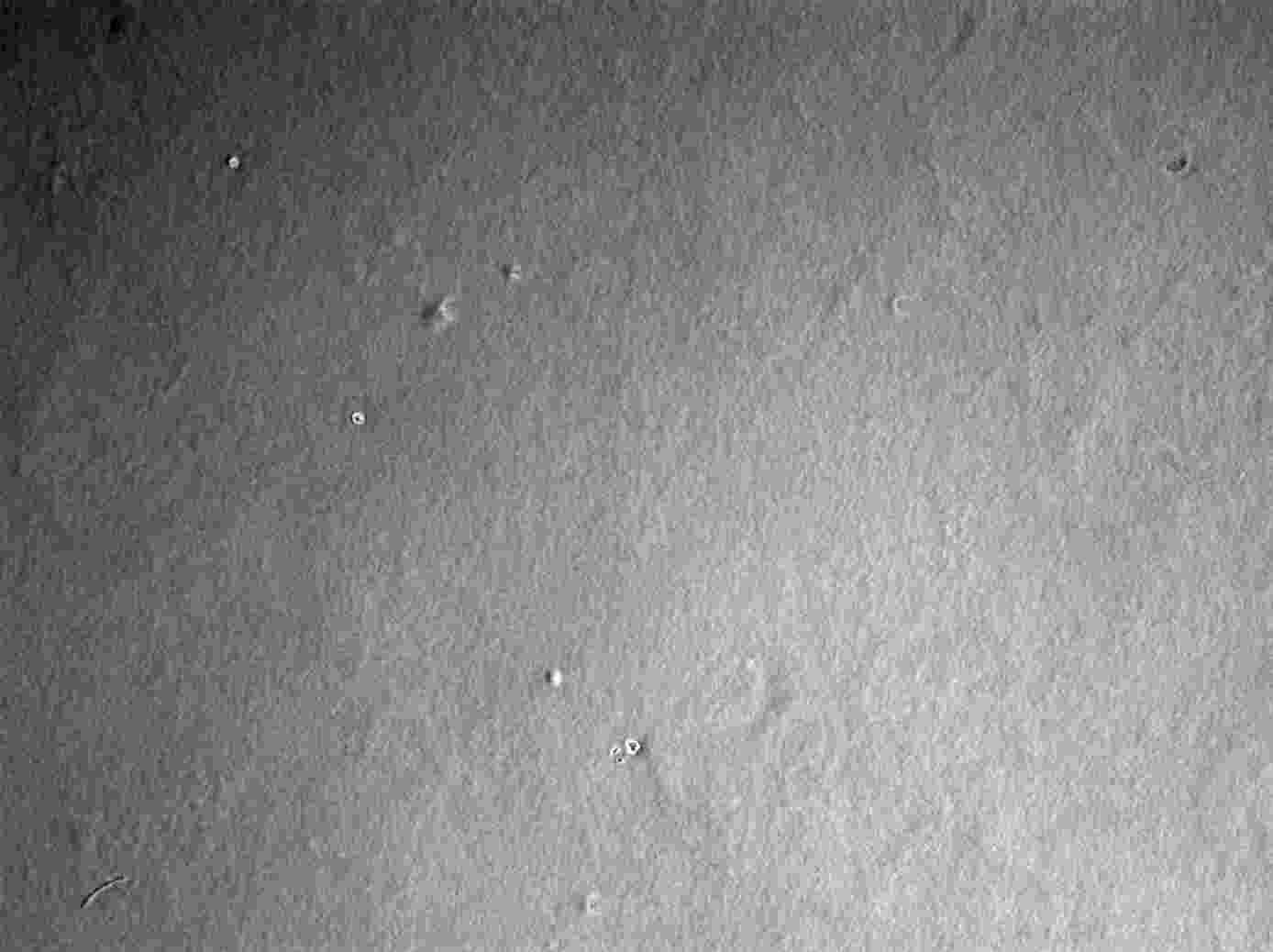

Supplement: S2 File — The raw data are presented in Raw data.zip. (ZIP) [file pone.0339611.s002.zip › Raw data/Figure 4/soft agar/day 1/4+EV-day1 (10).jpg]

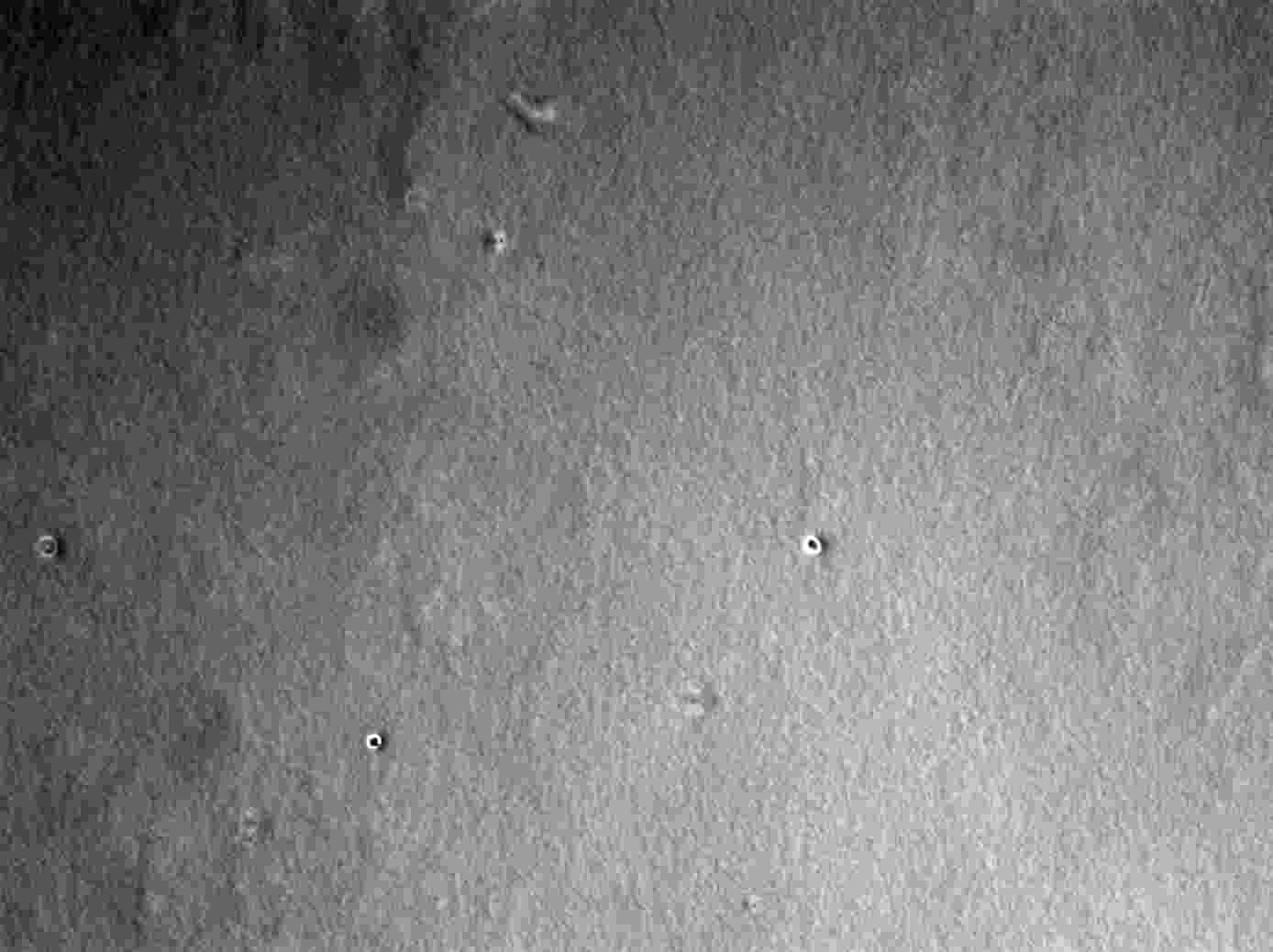

Supplement: S2 File — The raw data are presented in Raw data.zip. (ZIP) [file pone.0339611.s002.zip › Raw data/Figure 4/soft agar/day 1/4+EV-day1 (11).jpg]

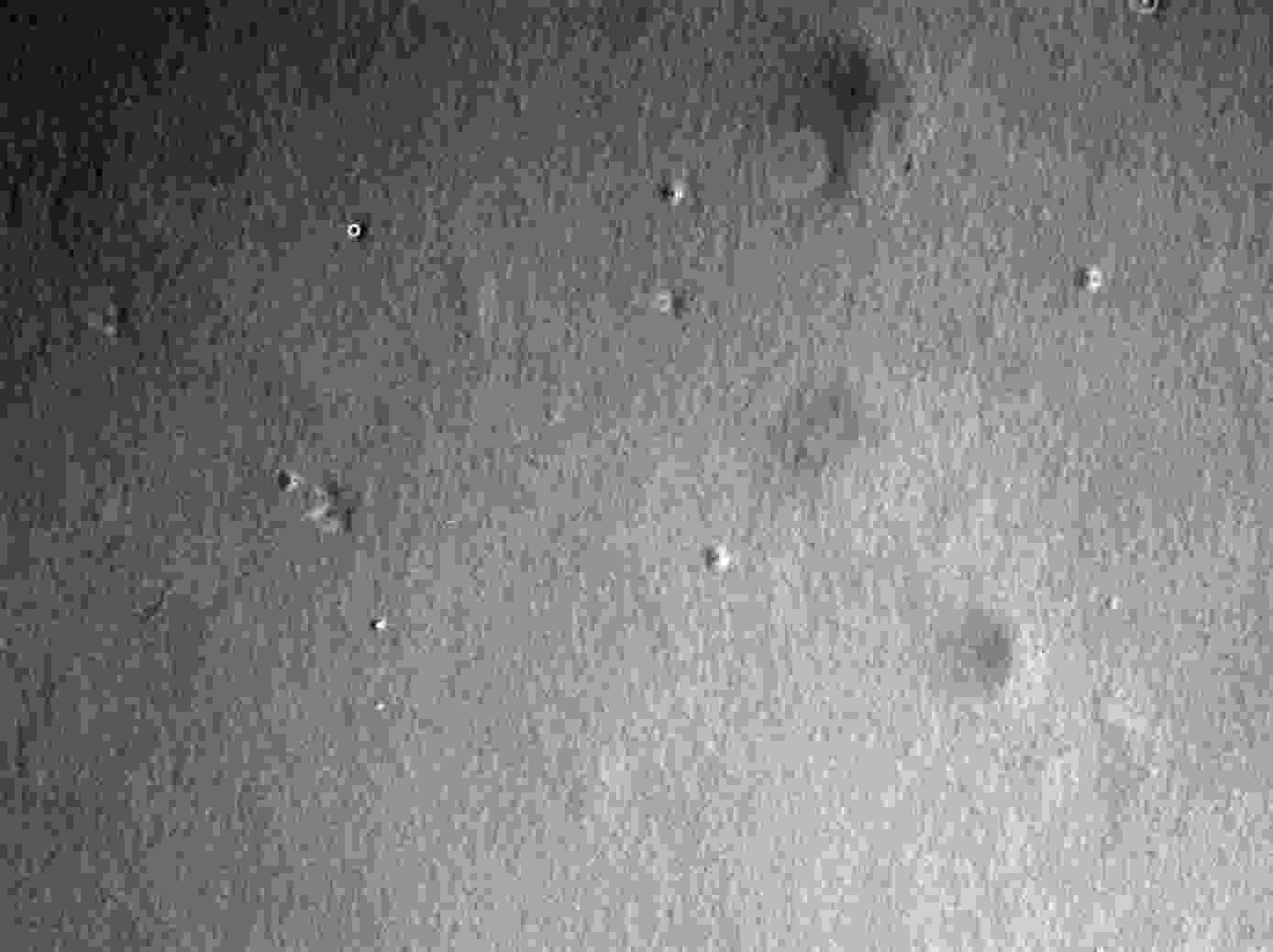

Supplement: S2 File — The raw data are presented in Raw data.zip. (ZIP) [file pone.0339611.s002.zip › Raw data/Figure 4/soft agar/day 1/4+EV-day1 (12).jpg]

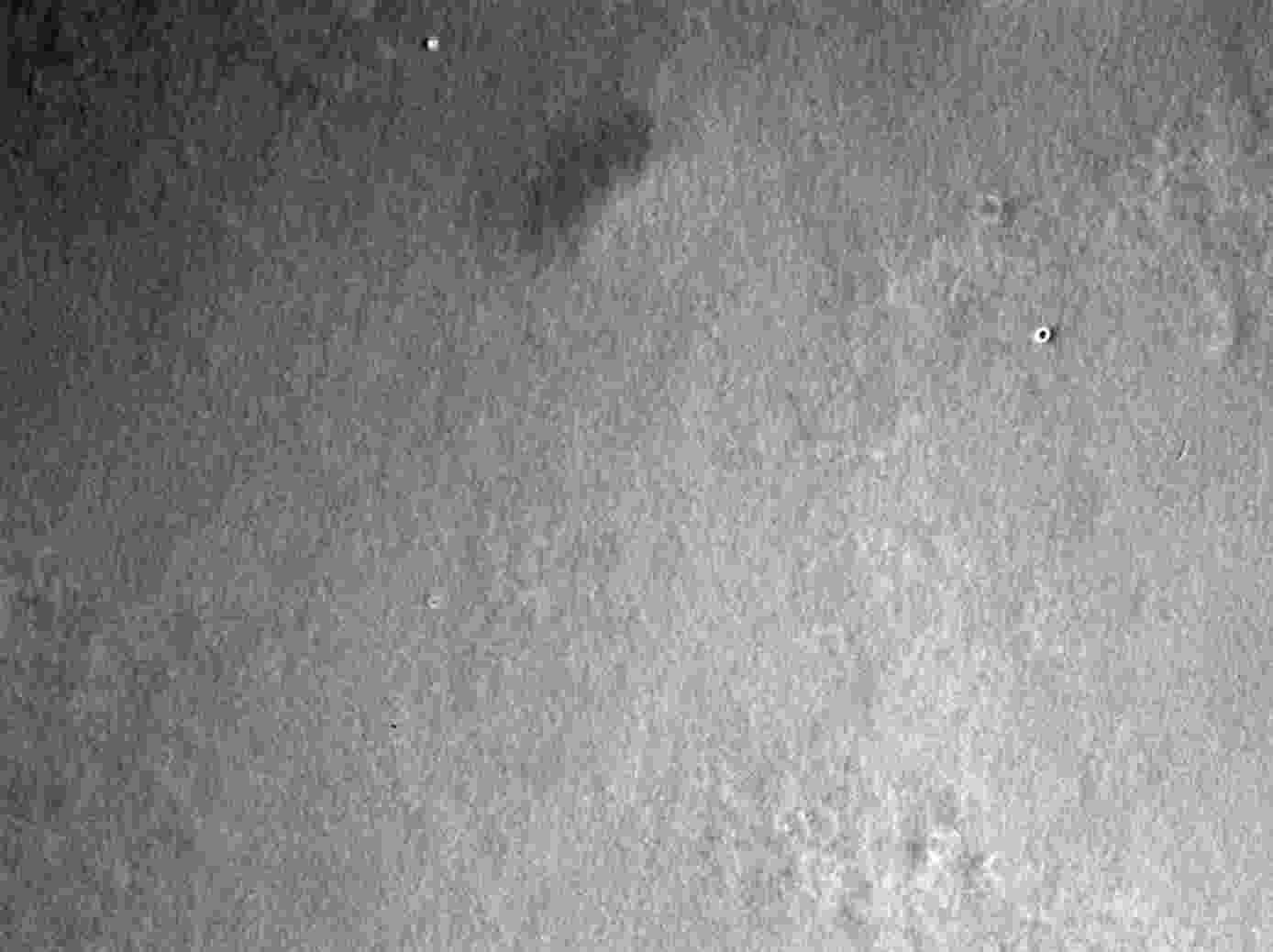

Supplement: S2 File — The raw data are presented in Raw data.zip. (ZIP) [file pone.0339611.s002.zip › Raw data/Figure 4/soft agar/day 1/4+EV-day1 (13).jpg]

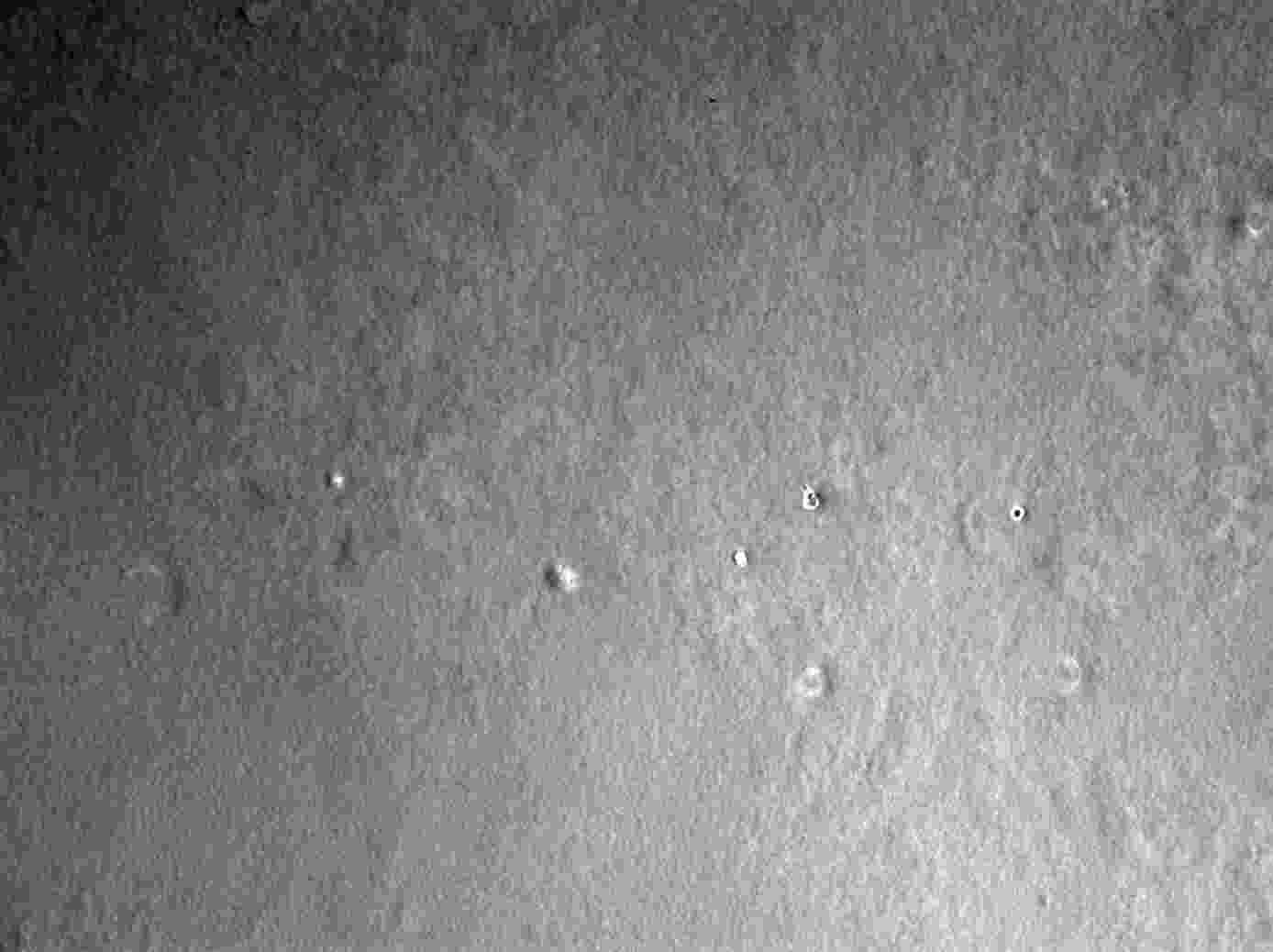

Supplement: S2 File — The raw data are presented in Raw data.zip. (ZIP) [file pone.0339611.s002.zip › Raw data/Figure 4/soft agar/day 1/4+EV-day1 (14).jpg]

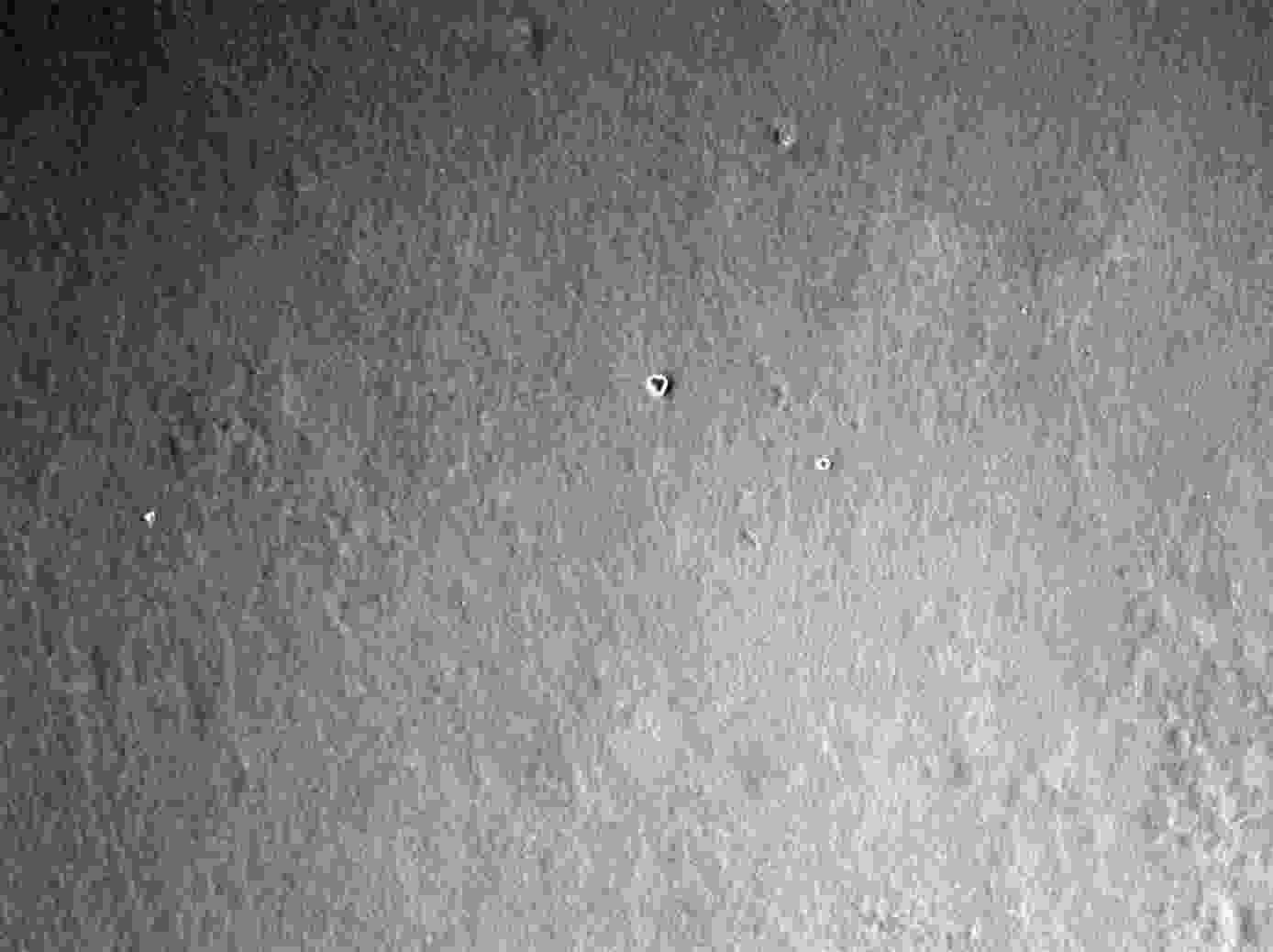

Supplement: S2 File — The raw data are presented in Raw data.zip. (ZIP) [file pone.0339611.s002.zip › Raw data/Figure 4/soft agar/day 1/4+EV-day1 (15).jpg]

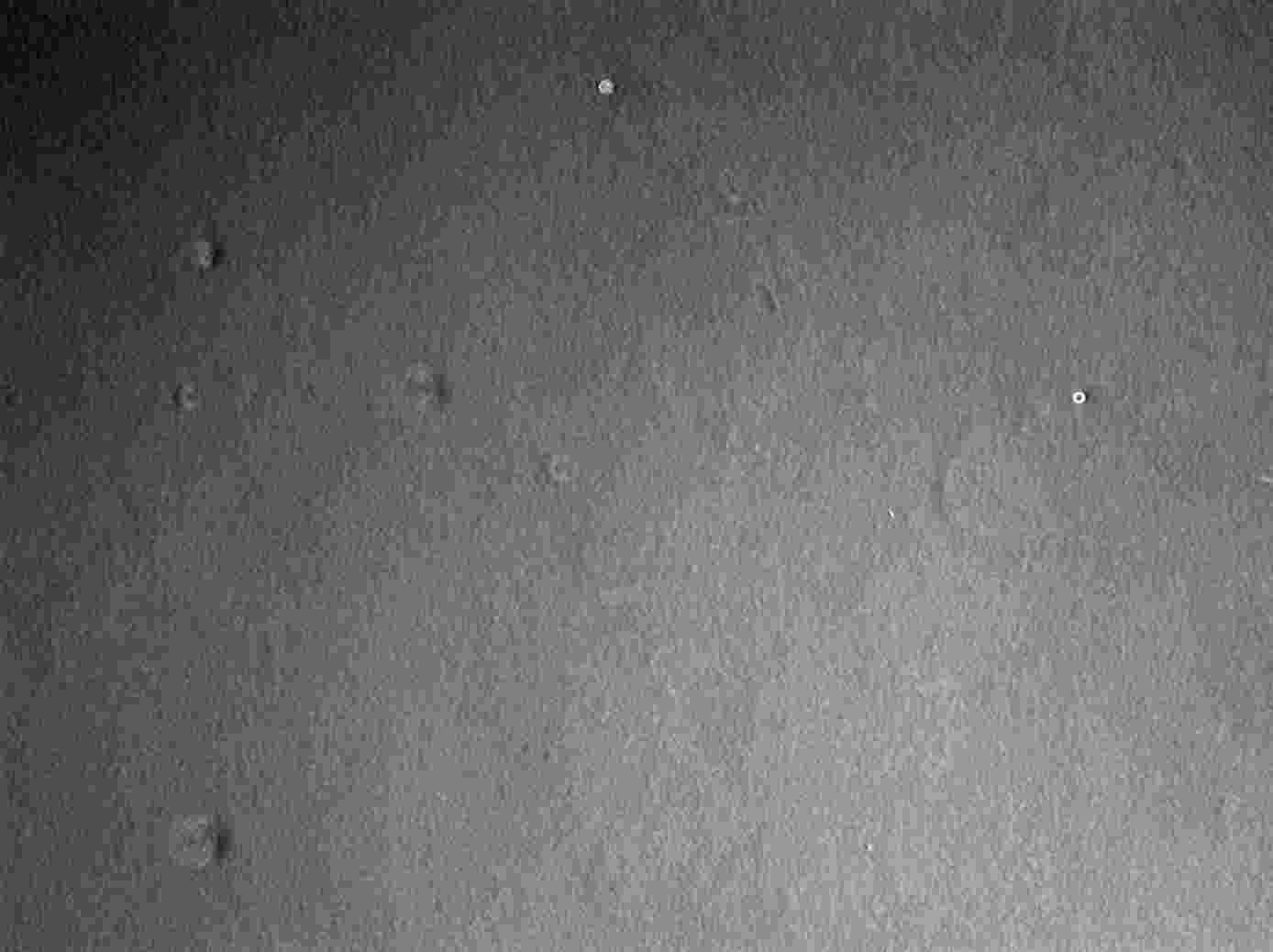

Supplement: S2 File — The raw data are presented in Raw data.zip. (ZIP) [file pone.0339611.s002.zip › Raw data/Figure 4/soft agar/day 1/4+EV-day1 (2).jpg]

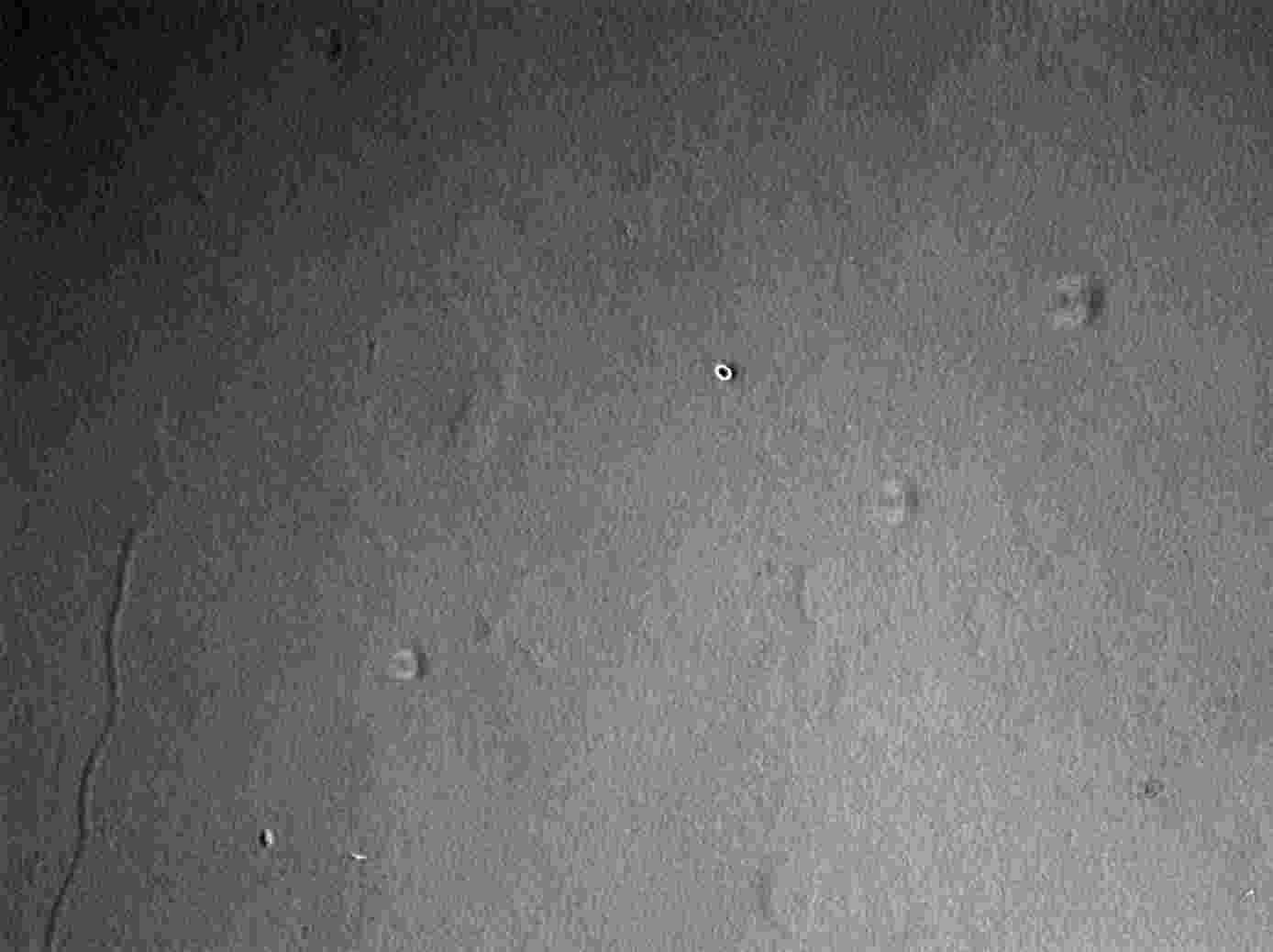

Supplement: S2 File — The raw data are presented in Raw data.zip. (ZIP) [file pone.0339611.s002.zip › Raw data/Figure 4/soft agar/day 1/4+EV-day1 (3).jpg]

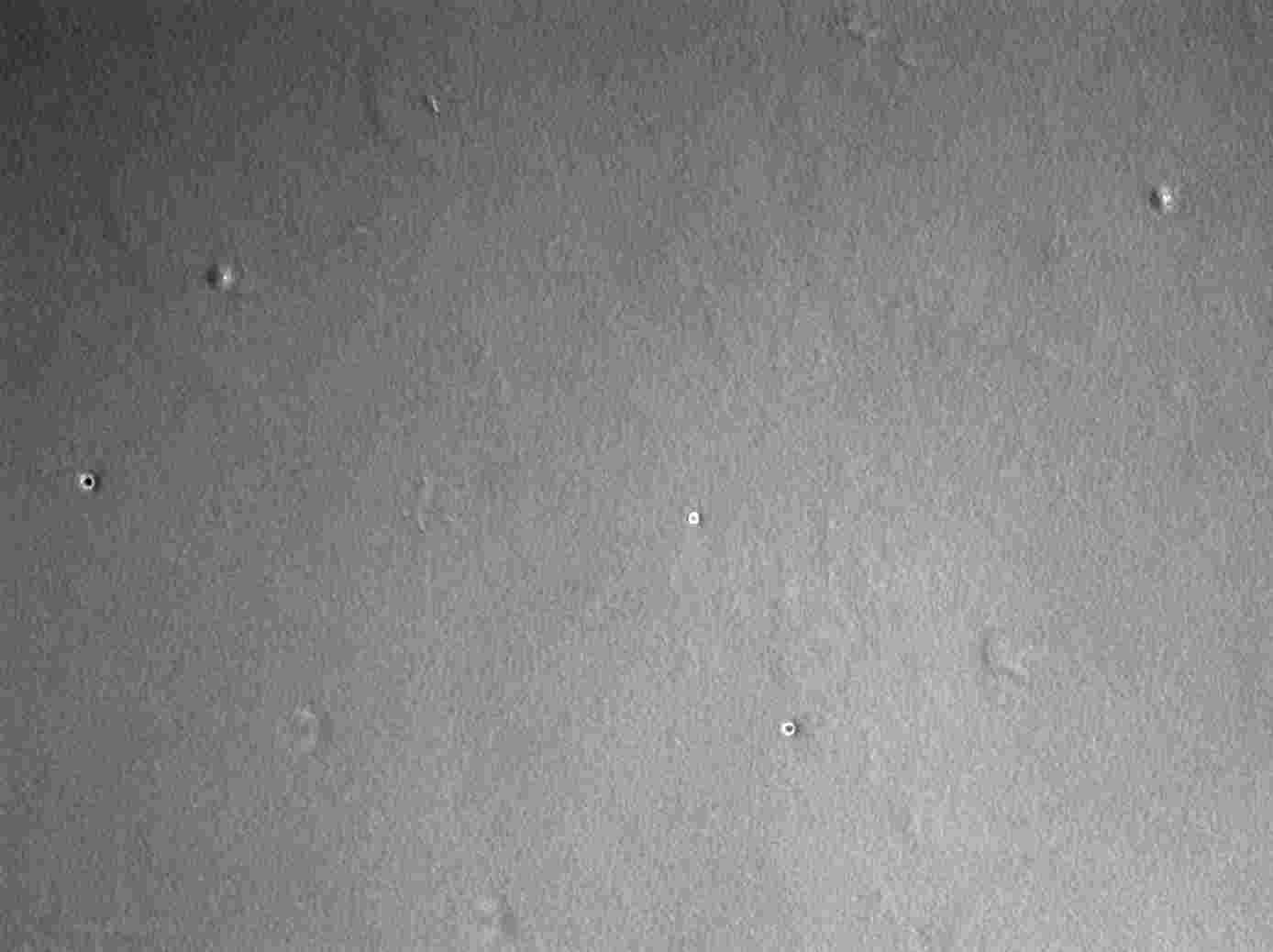

Supplement: S2 File — The raw data are presented in Raw data.zip. (ZIP) [file pone.0339611.s002.zip › Raw data/Figure 4/soft agar/day 1/4+EV-day1 (4).jpg]

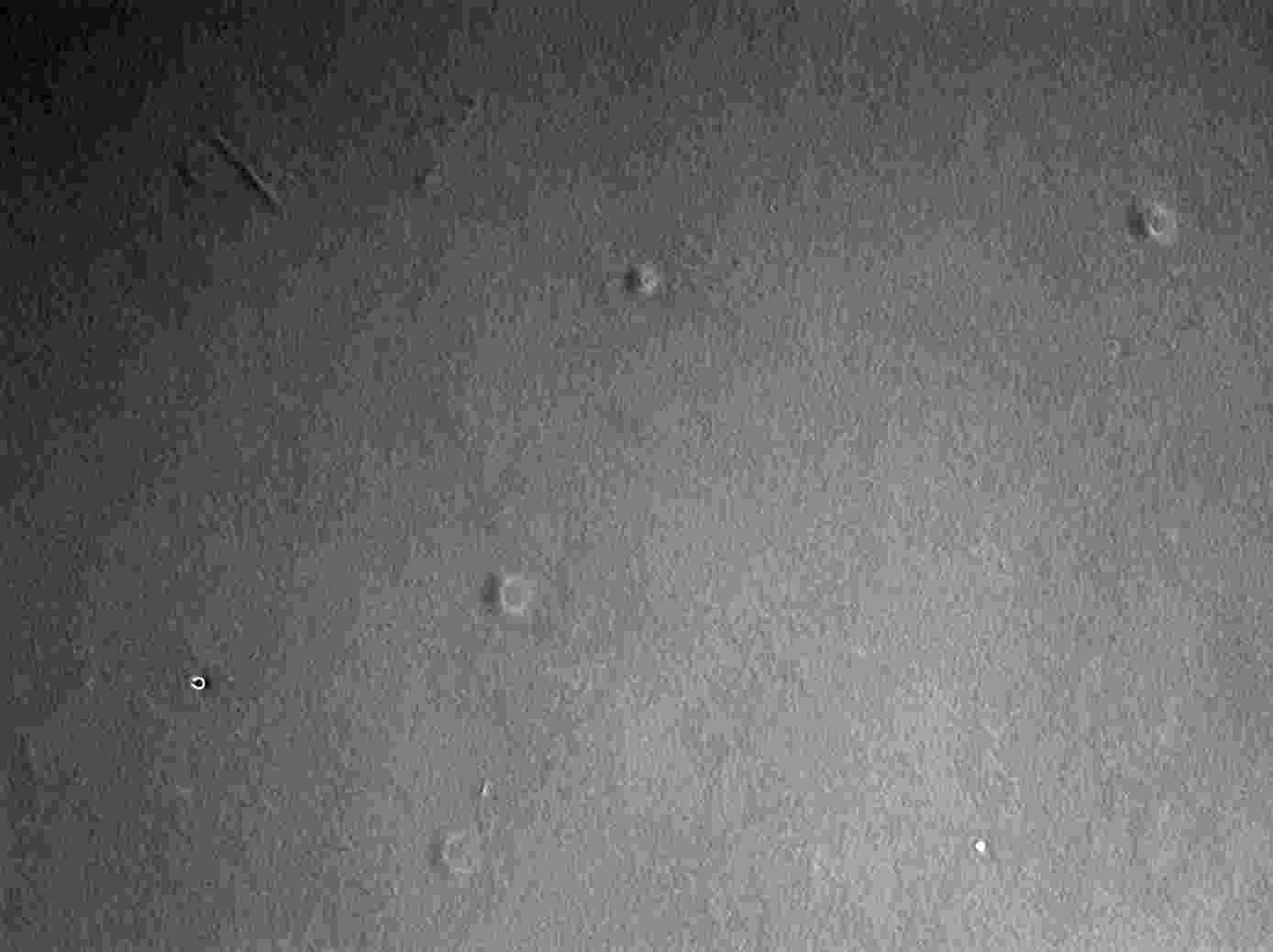

Supplement: S2 File — The raw data are presented in Raw data.zip. (ZIP) [file pone.0339611.s002.zip › Raw data/Figure 4/soft agar/day 1/4+EV-day1 (5).jpg]

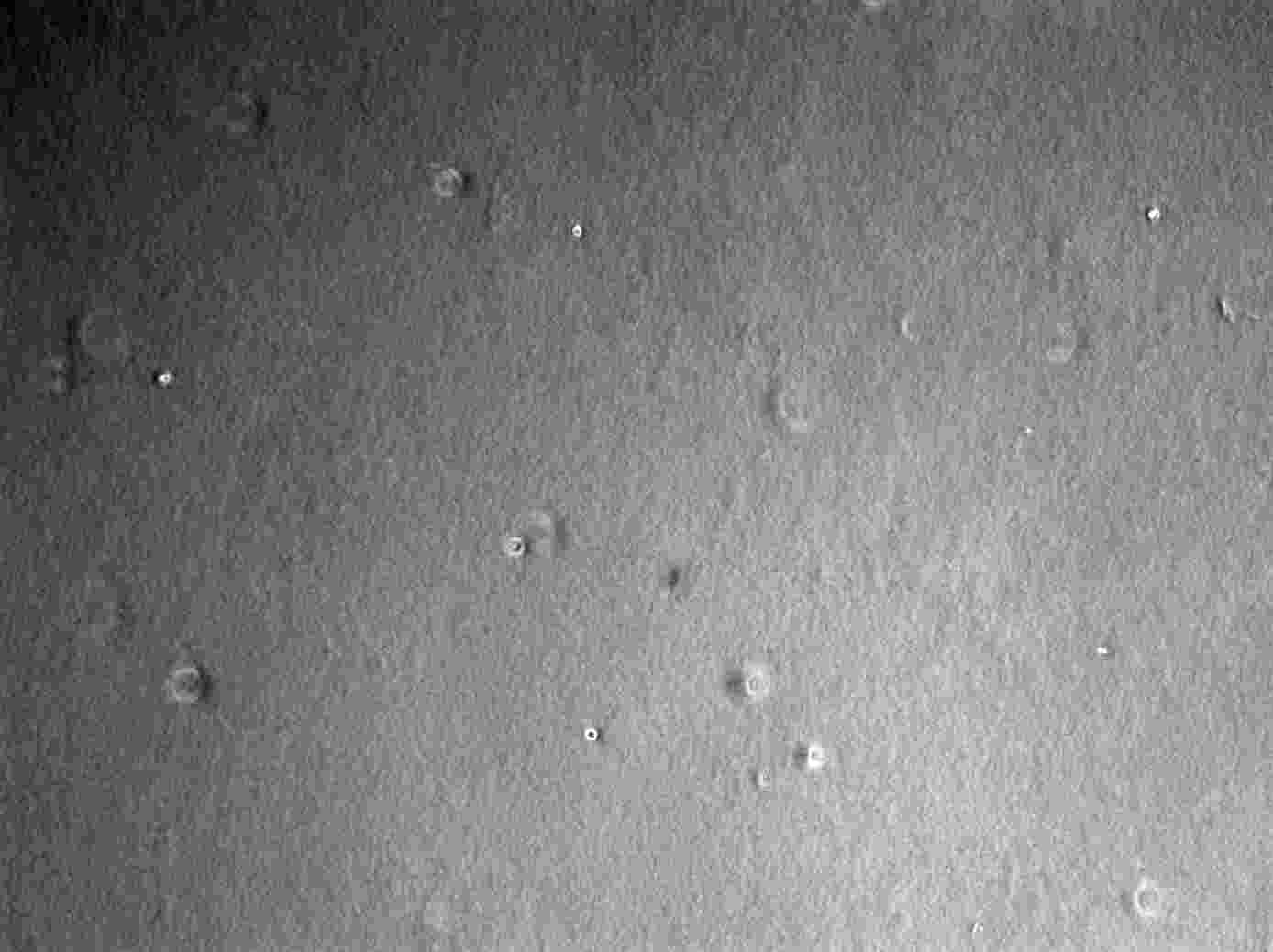

Supplement: S2 File — The raw data are presented in Raw data.zip. (ZIP) [file pone.0339611.s002.zip › Raw data/Figure 4/soft agar/day 1/4+EV-day1 (6).jpg]

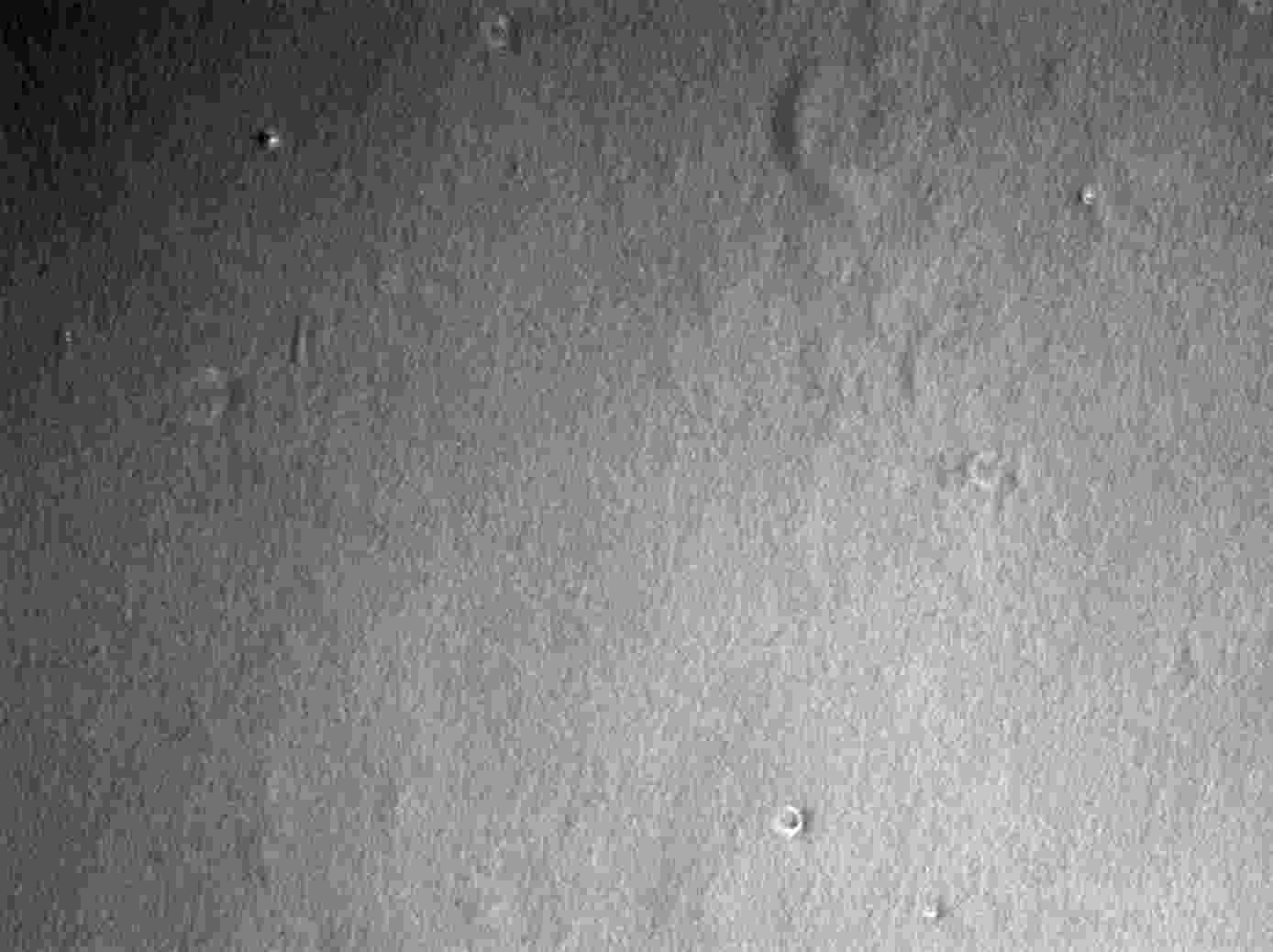

Supplement: S2 File — The raw data are presented in Raw data.zip. (ZIP) [file pone.0339611.s002.zip › Raw data/Figure 4/soft agar/day 1/4+EV-day1 (7).jpg]

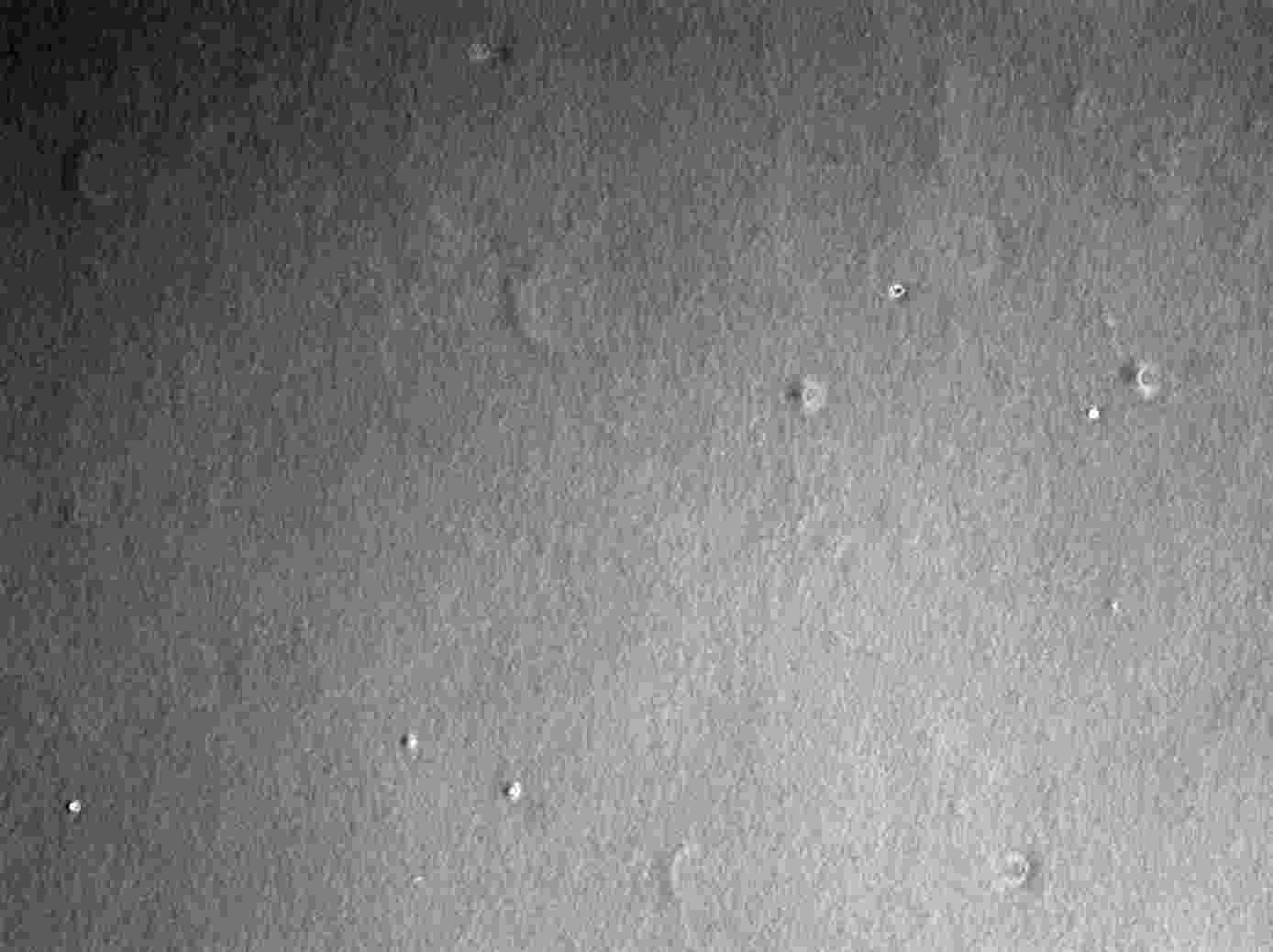

Supplement: S2 File — The raw data are presented in Raw data.zip. (ZIP) [file pone.0339611.s002.zip › Raw data/Figure 4/soft agar/day 1/4+EV-day1 (8).jpg]

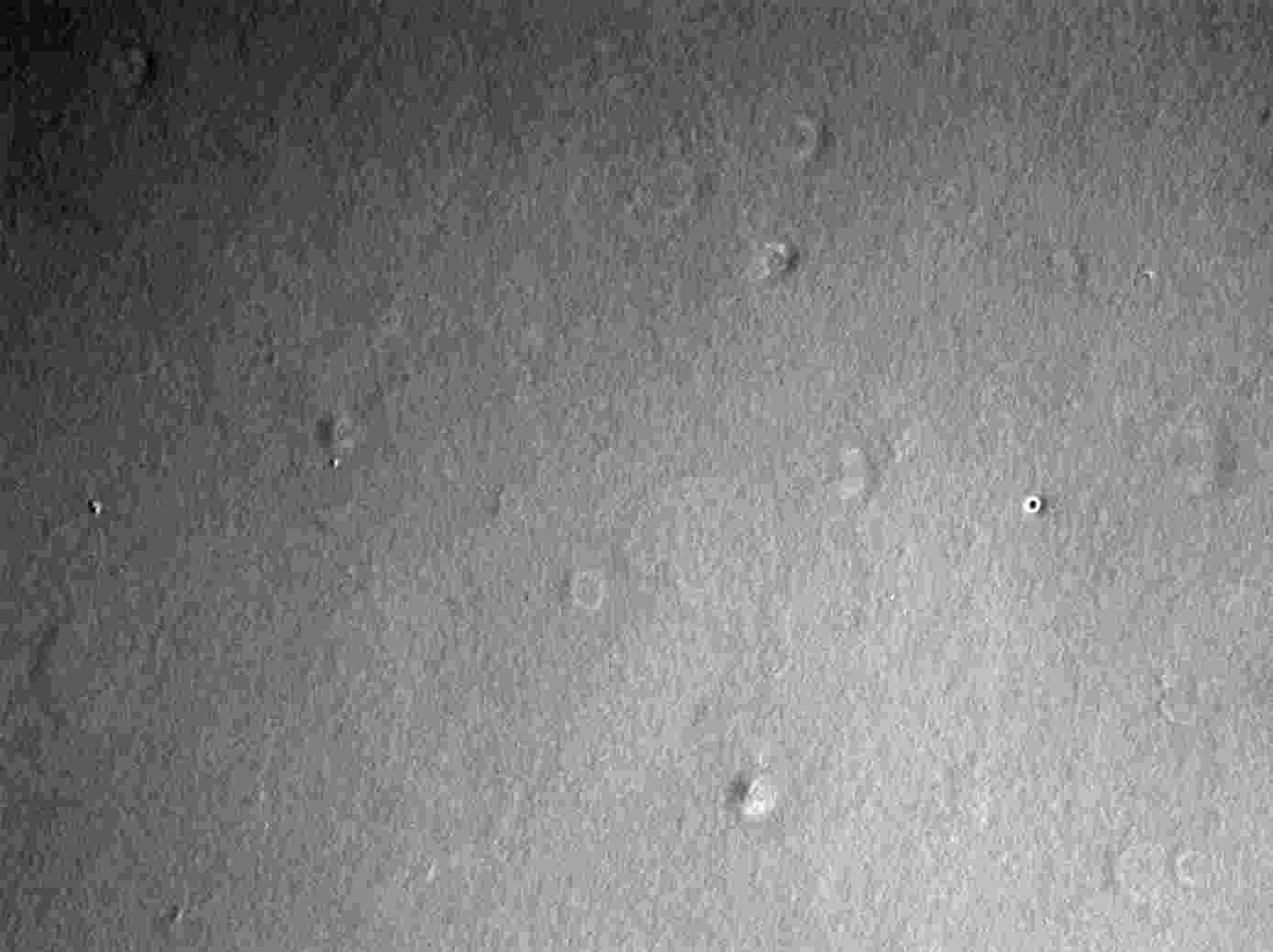

Supplement: S2 File — The raw data are presented in Raw data.zip. (ZIP) [file pone.0339611.s002.zip › Raw data/Figure 4/soft agar/day 1/4+EV-day1 (9).jpg]

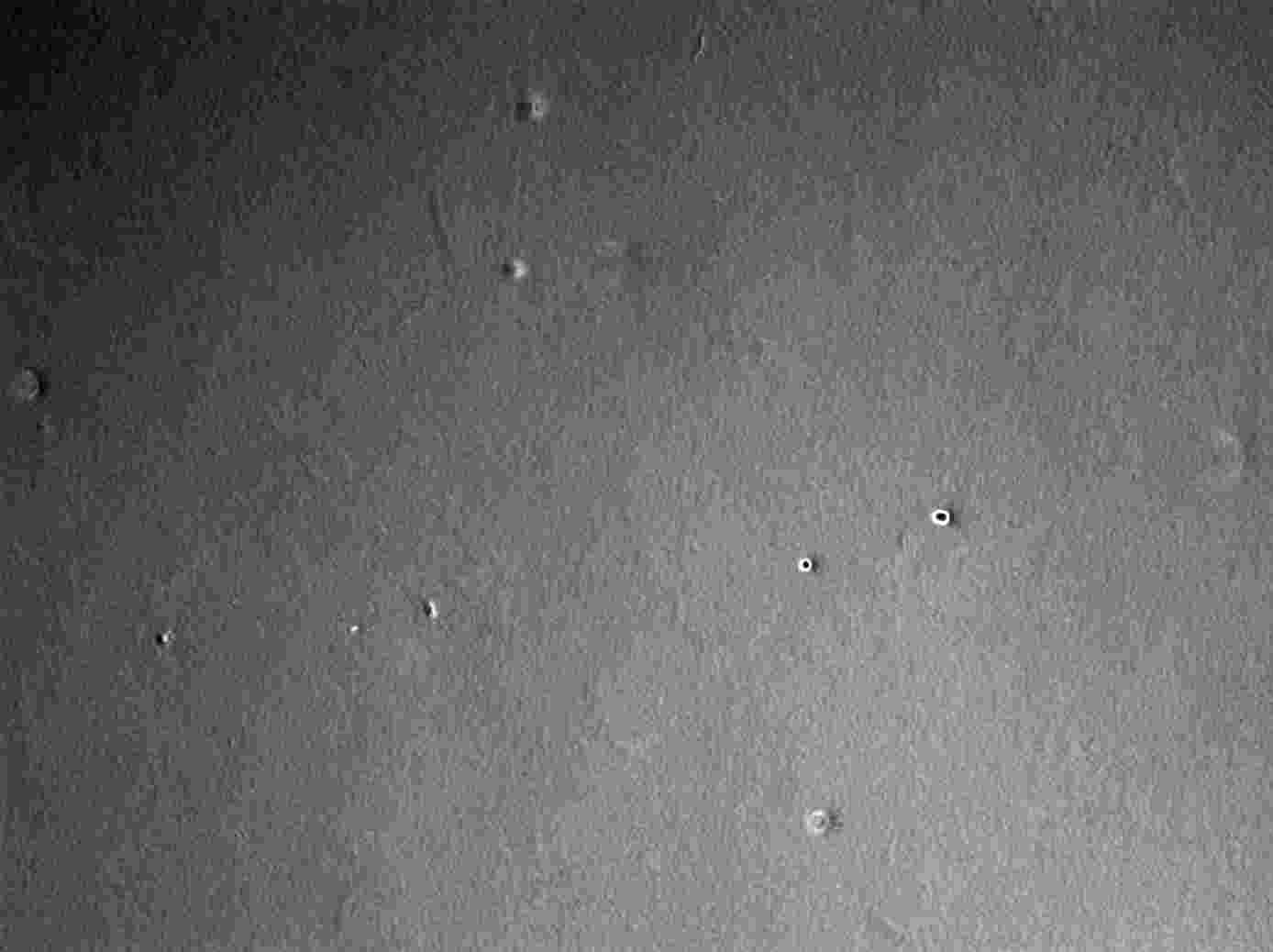

Supplement: S2 File — The raw data are presented in Raw data.zip. (ZIP) [file pone.0339611.s002.zip › Raw data/Figure 4/soft agar/day 1/4+EV-day1.jpg]

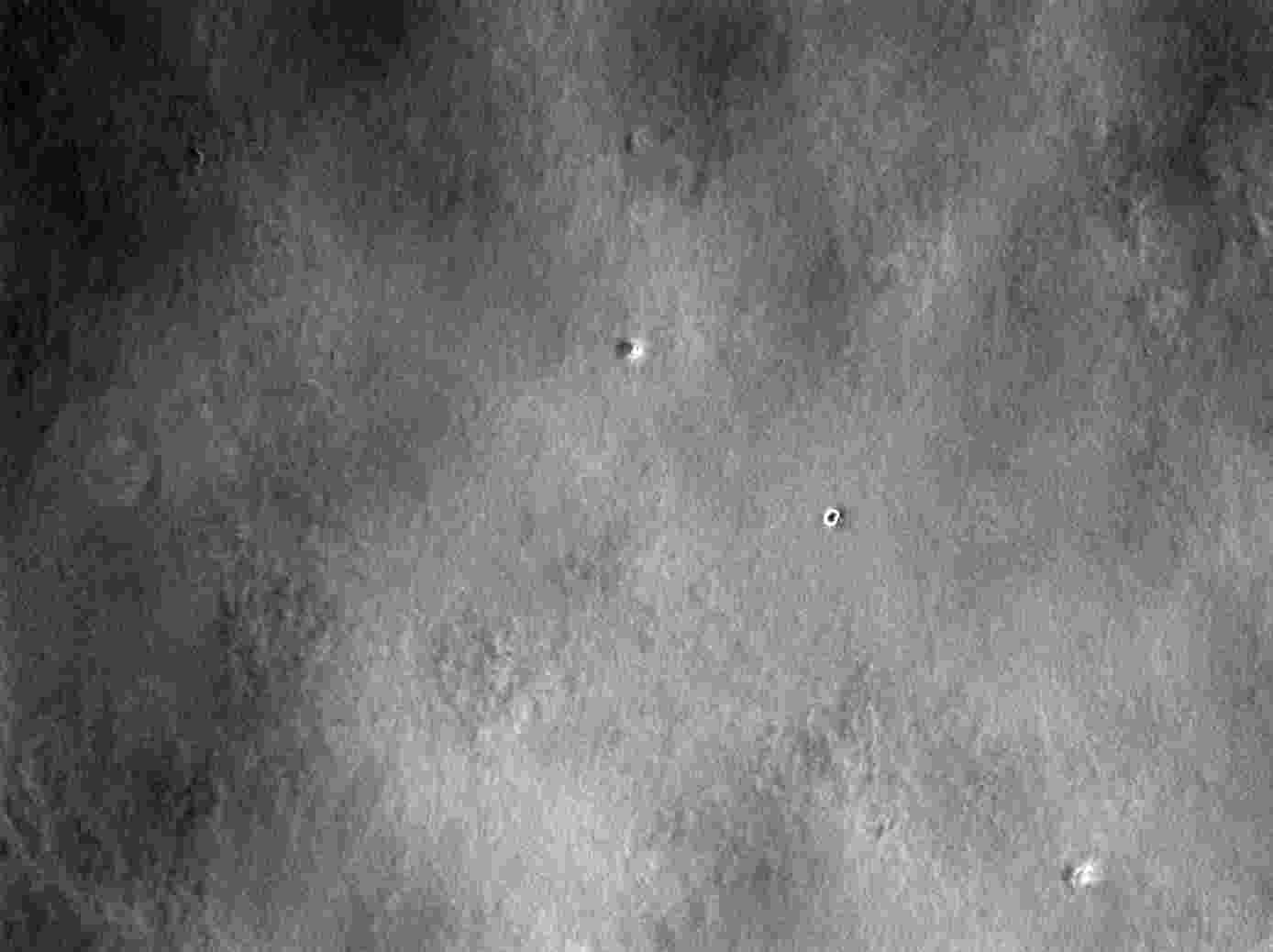

Supplement: S2 File — The raw data are presented in Raw data.zip. (ZIP) [file pone.0339611.s002.zip › Raw data/Figure 4/soft agar/day 1/4+OE-day1 (10).jpg]

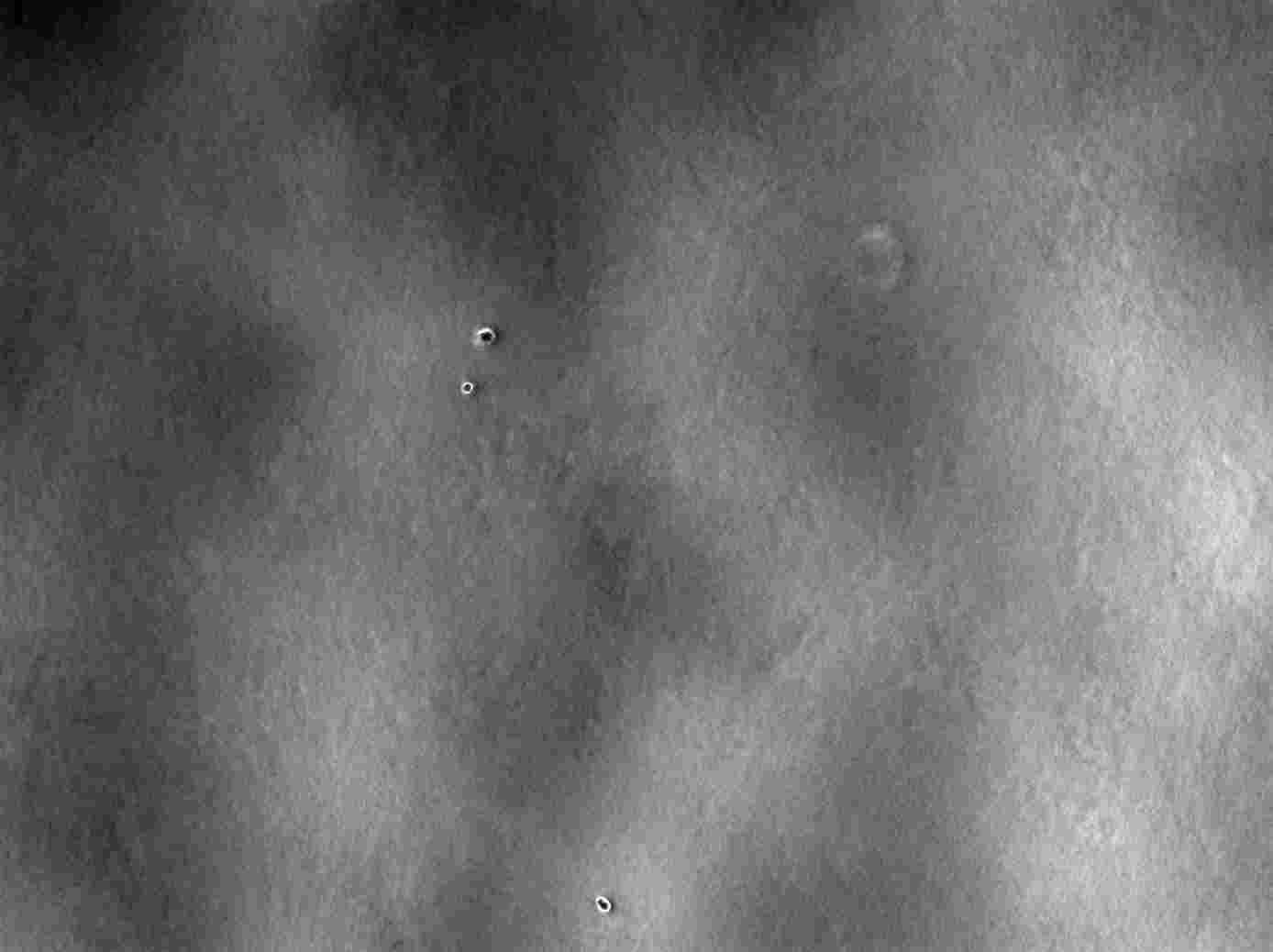

Supplement: S2 File — The raw data are presented in Raw data.zip. (ZIP) [file pone.0339611.s002.zip › Raw data/Figure 4/soft agar/day 1/4+OE-day1 (11).jpg]

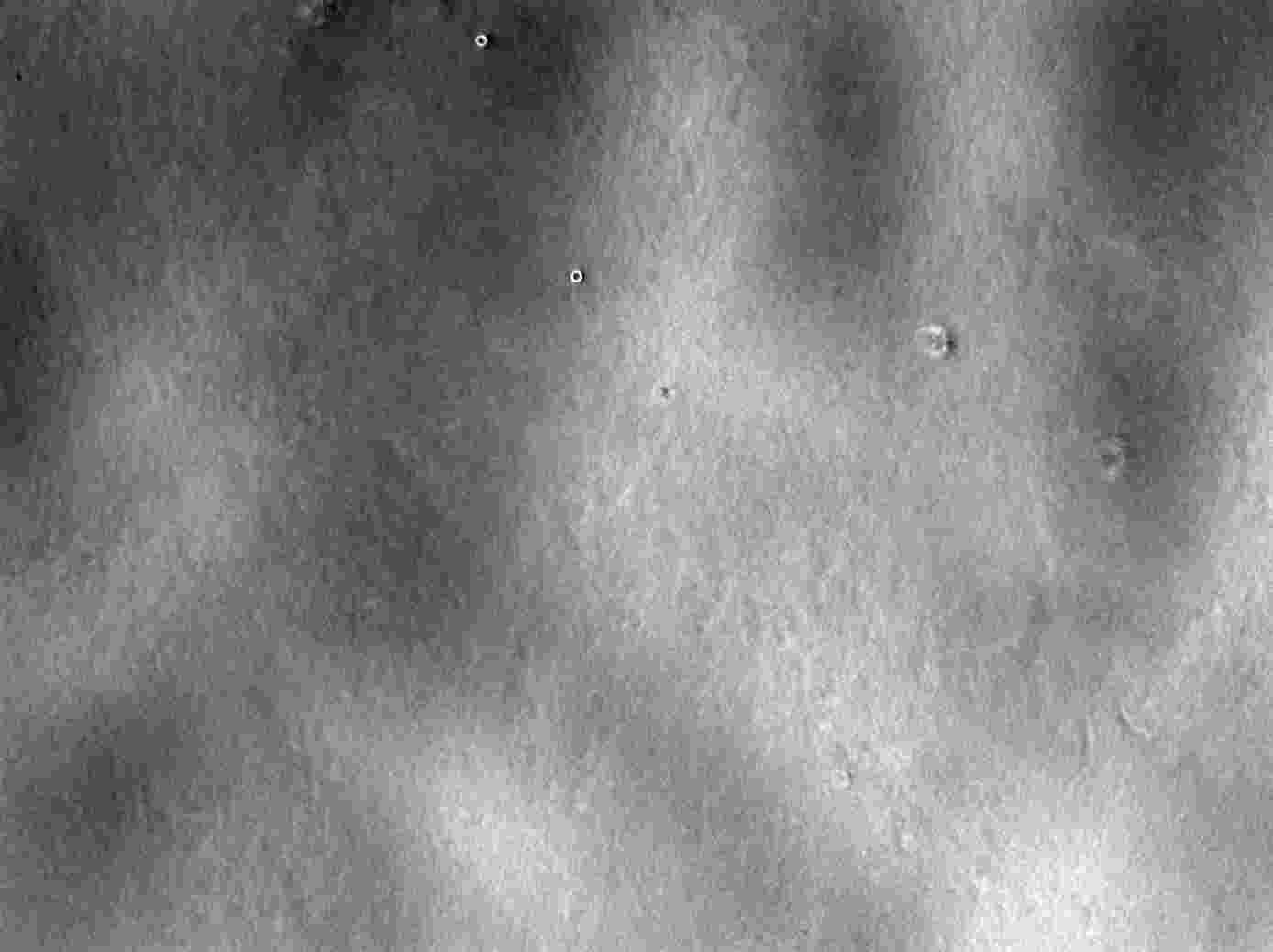

Supplement: S2 File — The raw data are presented in Raw data.zip. (ZIP) [file pone.0339611.s002.zip › Raw data/Figure 4/soft agar/day 1/4+OE-day1 (12).jpg]

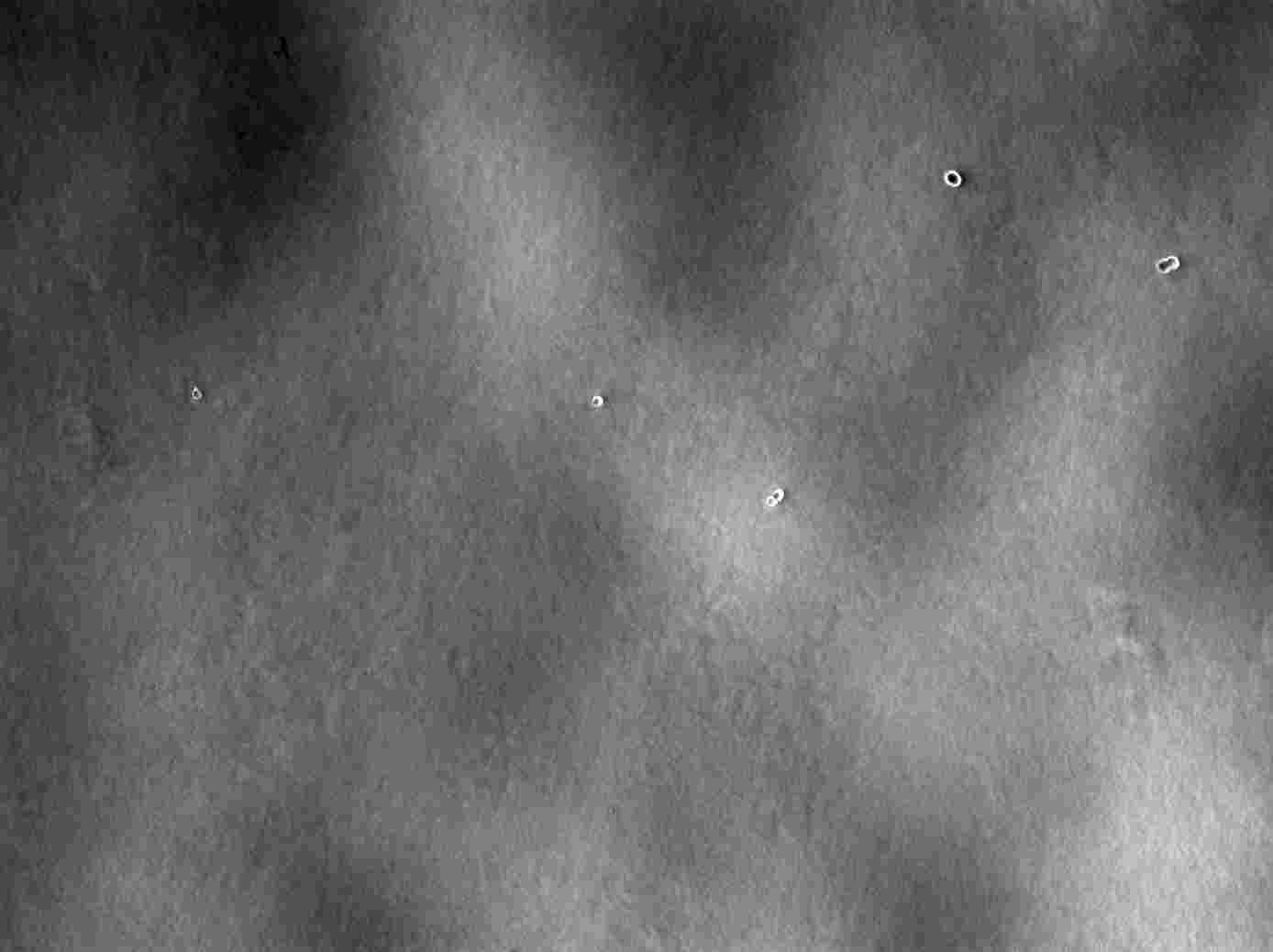

Supplement: S2 File — The raw data are presented in Raw data.zip. (ZIP) [file pone.0339611.s002.zip › Raw data/Figure 4/soft agar/day 1/4+OE-day1 (13).jpg]

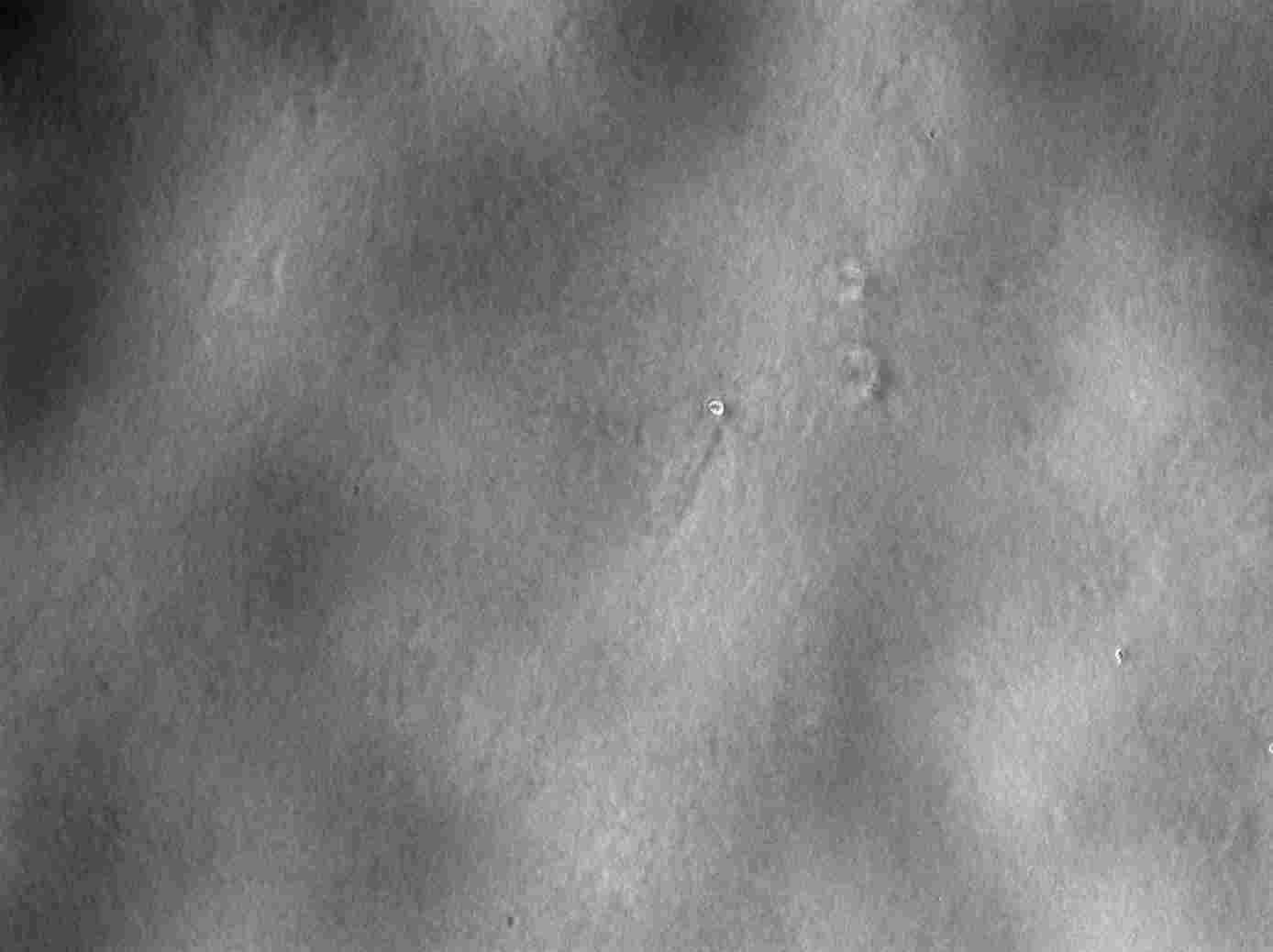

Supplement: S2 File — The raw data are presented in Raw data.zip. (ZIP) [file pone.0339611.s002.zip › Raw data/Figure 4/soft agar/day 1/4+OE-day1 (14).jpg]

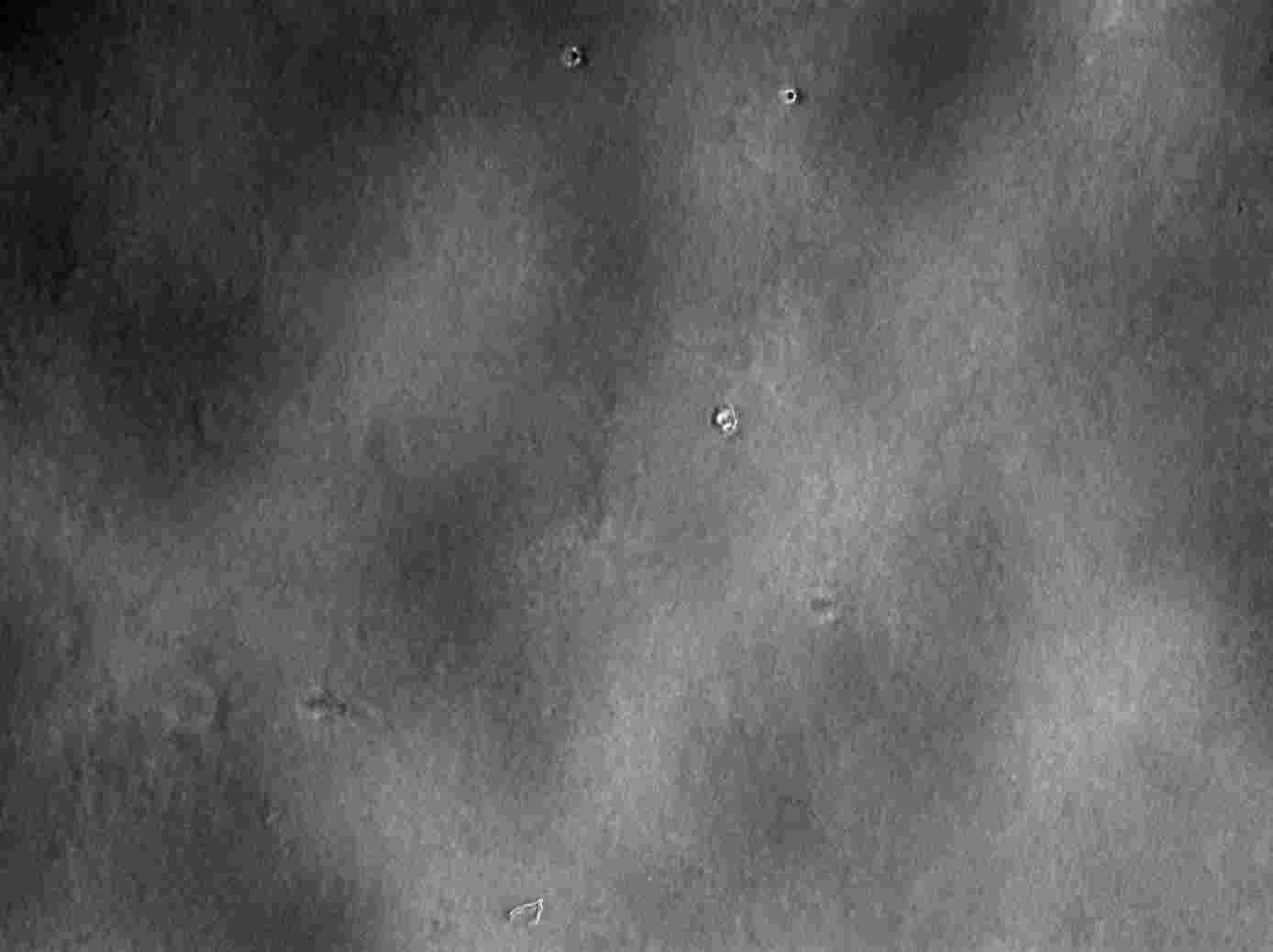

Supplement: S2 File — The raw data are presented in Raw data.zip. (ZIP) [file pone.0339611.s002.zip › Raw data/Figure 4/soft agar/day 1/4+OE-day1 (15).jpg]

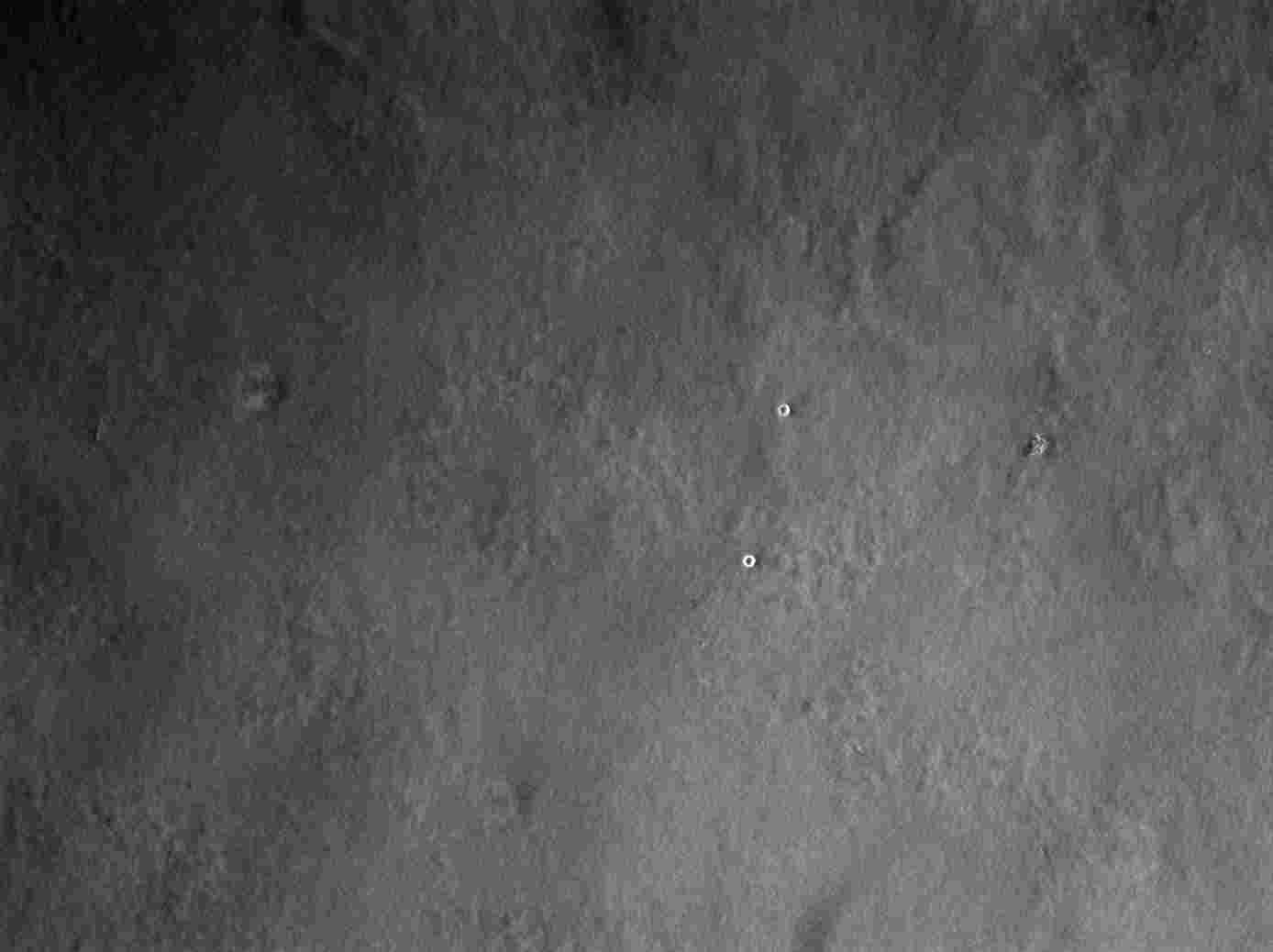

Supplement: S2 File — The raw data are presented in Raw data.zip. (ZIP) [file pone.0339611.s002.zip › Raw data/Figure 4/soft agar/day 1/4+OE-day1 (2).jpg]

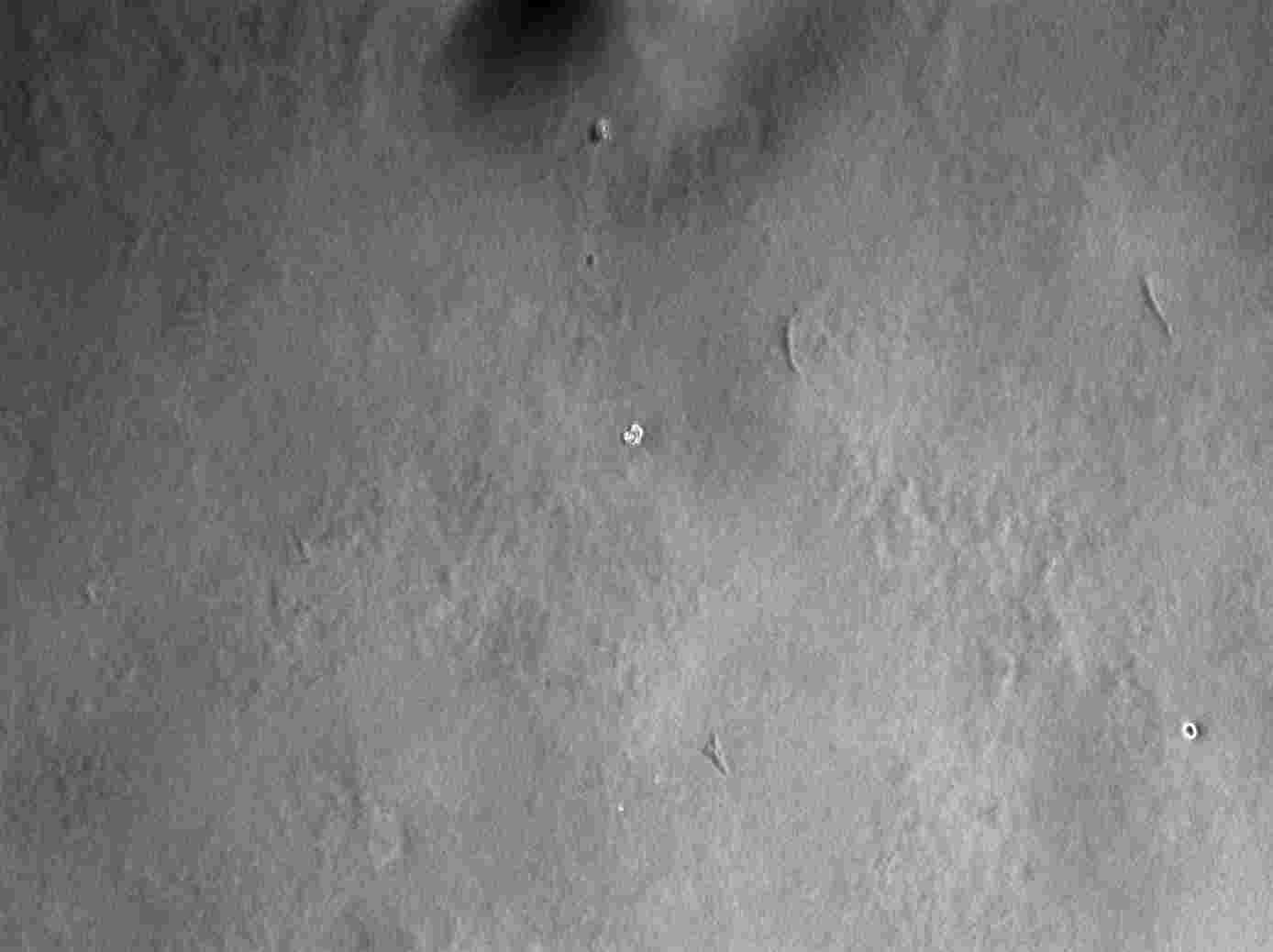

Supplement: S2 File — The raw data are presented in Raw data.zip. (ZIP) [file pone.0339611.s002.zip › Raw data/Figure 4/soft agar/day 1/4+OE-day1 (3).jpg]

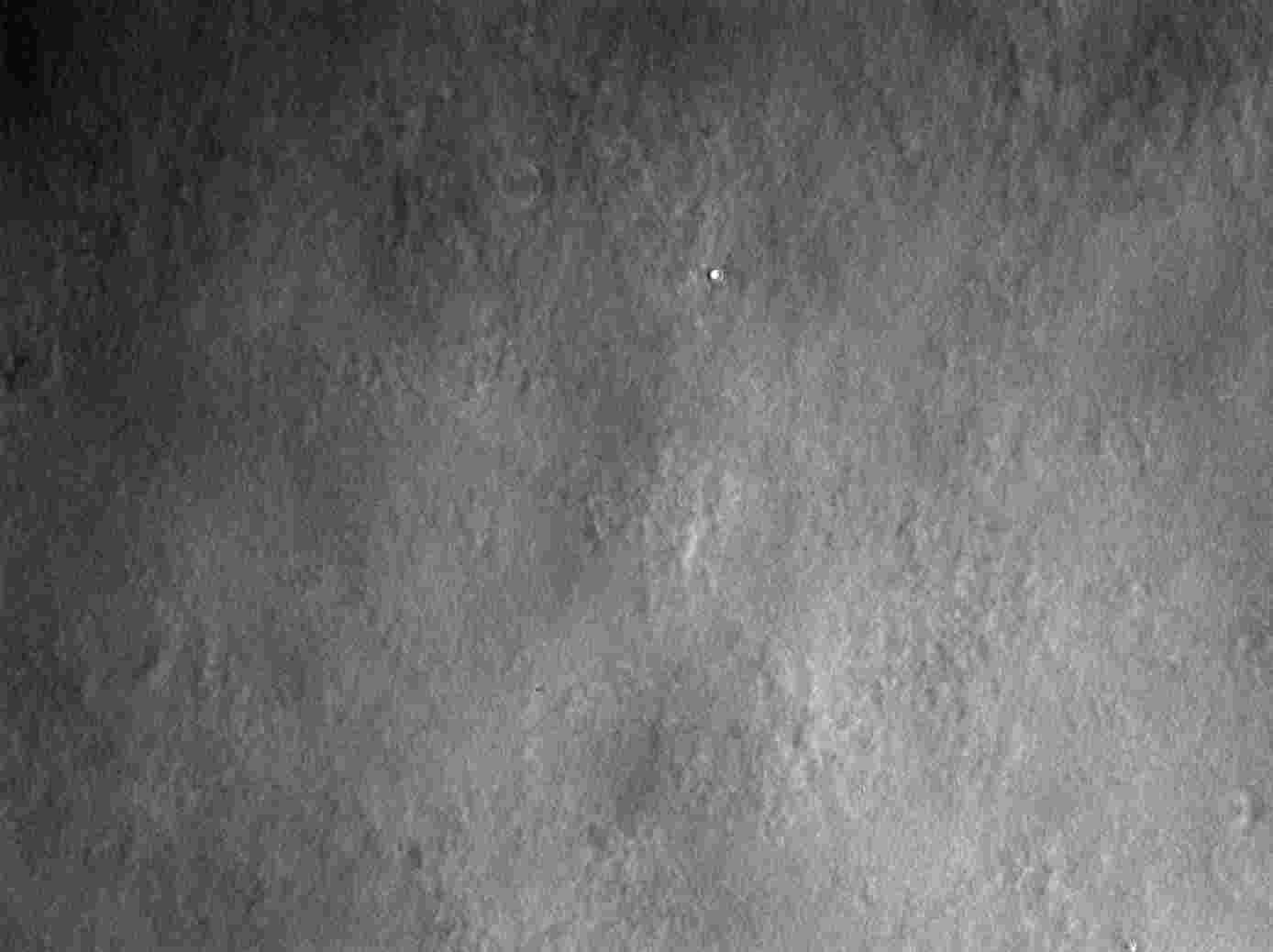

Supplement: S2 File — The raw data are presented in Raw data.zip. (ZIP) [file pone.0339611.s002.zip › Raw data/Figure 4/soft agar/day 1/4+OE-day1 (4).jpg]

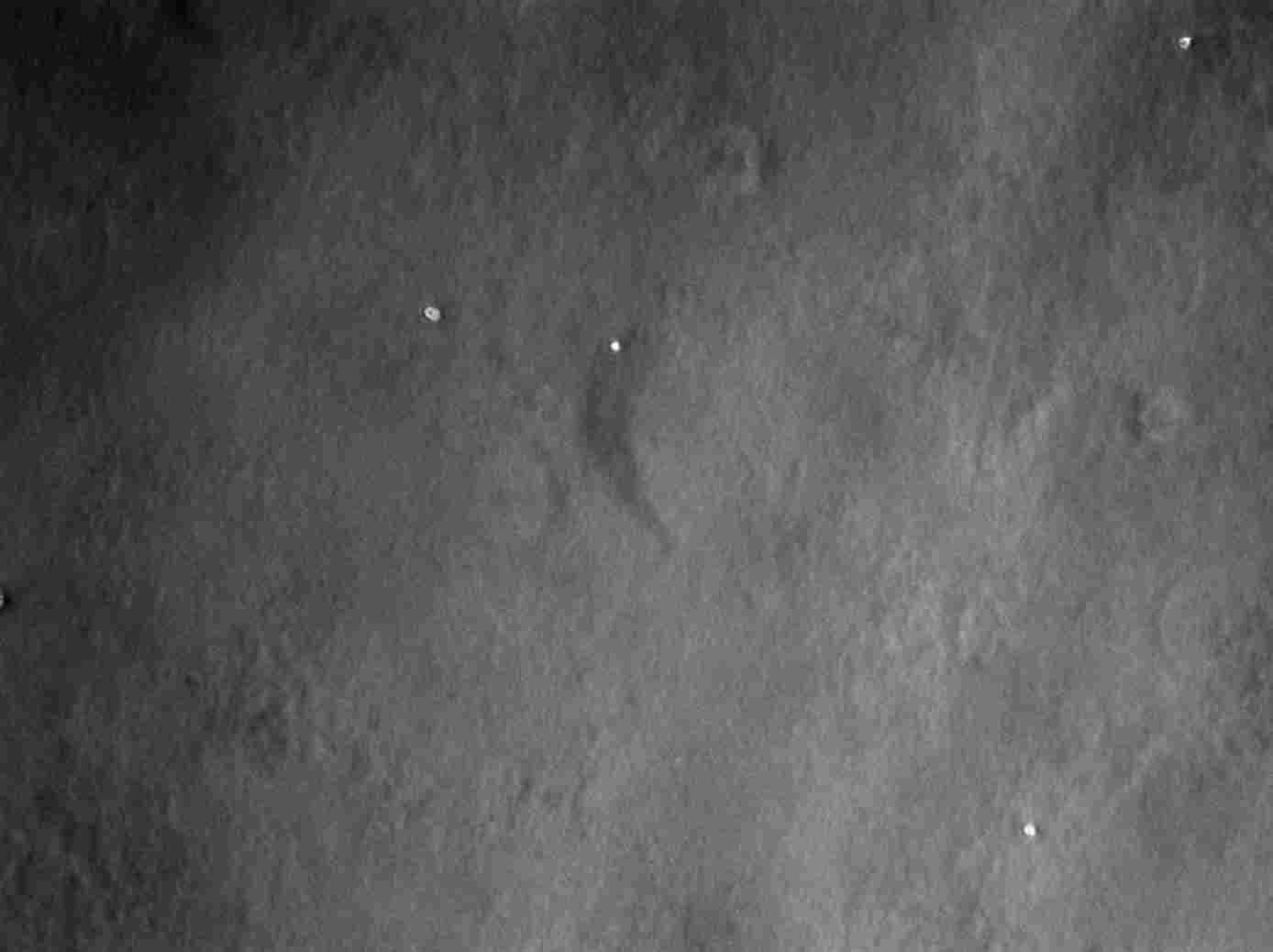

Supplement: S2 File — The raw data are presented in Raw data.zip. (ZIP) [file pone.0339611.s002.zip › Raw data/Figure 4/soft agar/day 1/4+OE-day1 (5).jpg]

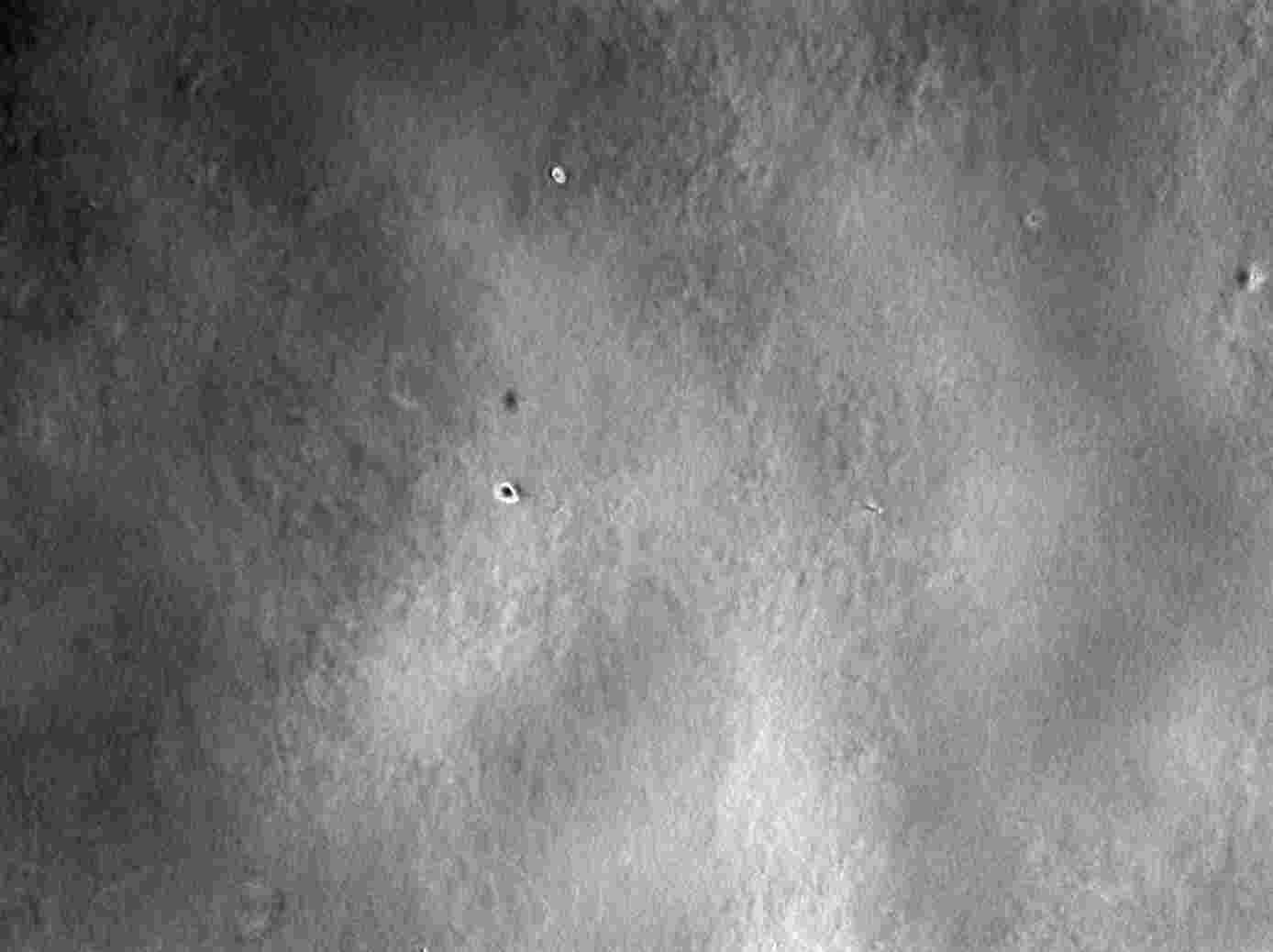

Supplement: S2 File — The raw data are presented in Raw data.zip. (ZIP) [file pone.0339611.s002.zip › Raw data/Figure 4/soft agar/day 1/4+OE-day1 (6).jpg]

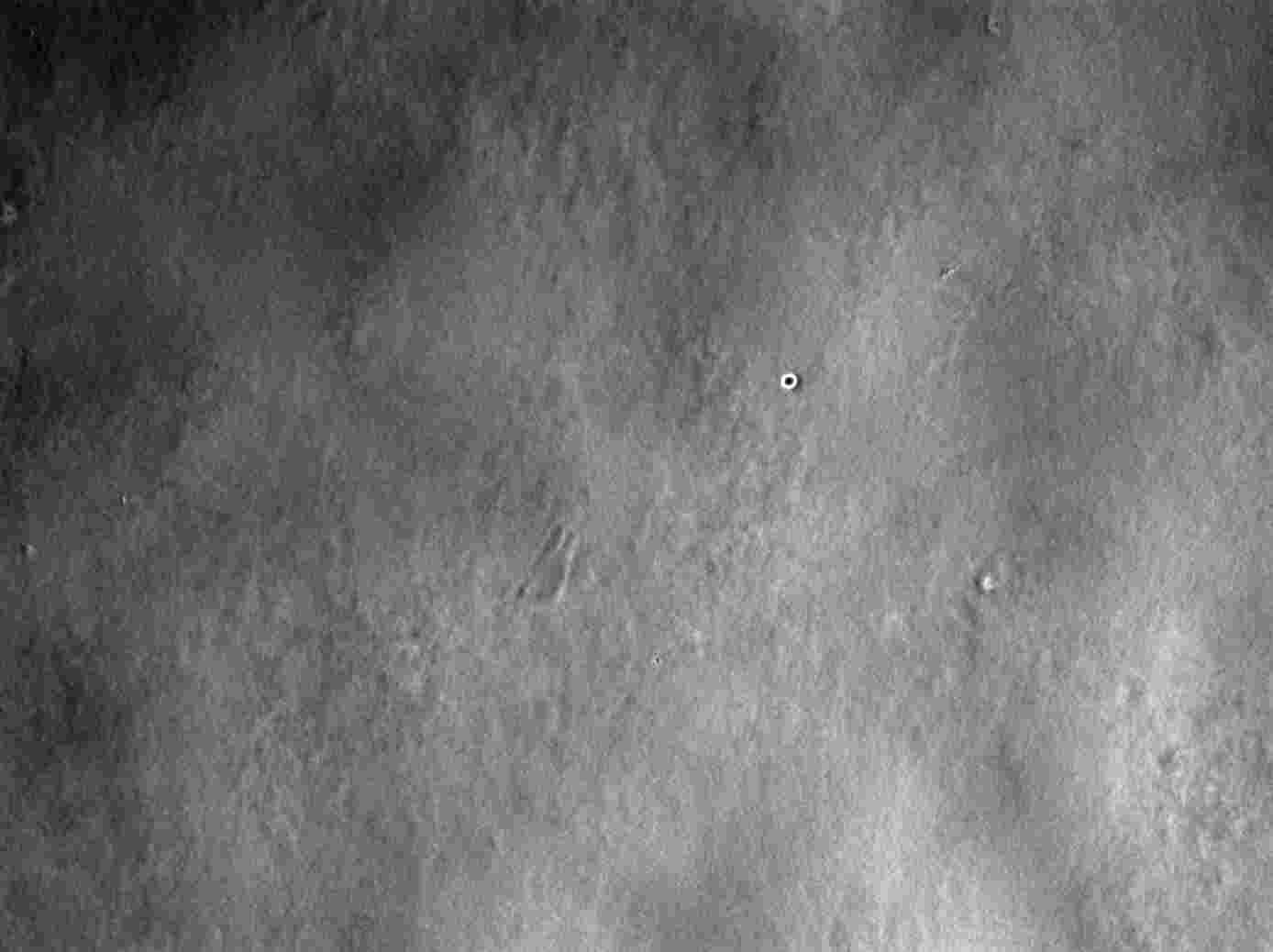

Supplement: S2 File — The raw data are presented in Raw data.zip. (ZIP) [file pone.0339611.s002.zip › Raw data/Figure 4/soft agar/day 1/4+OE-day1 (7).jpg]

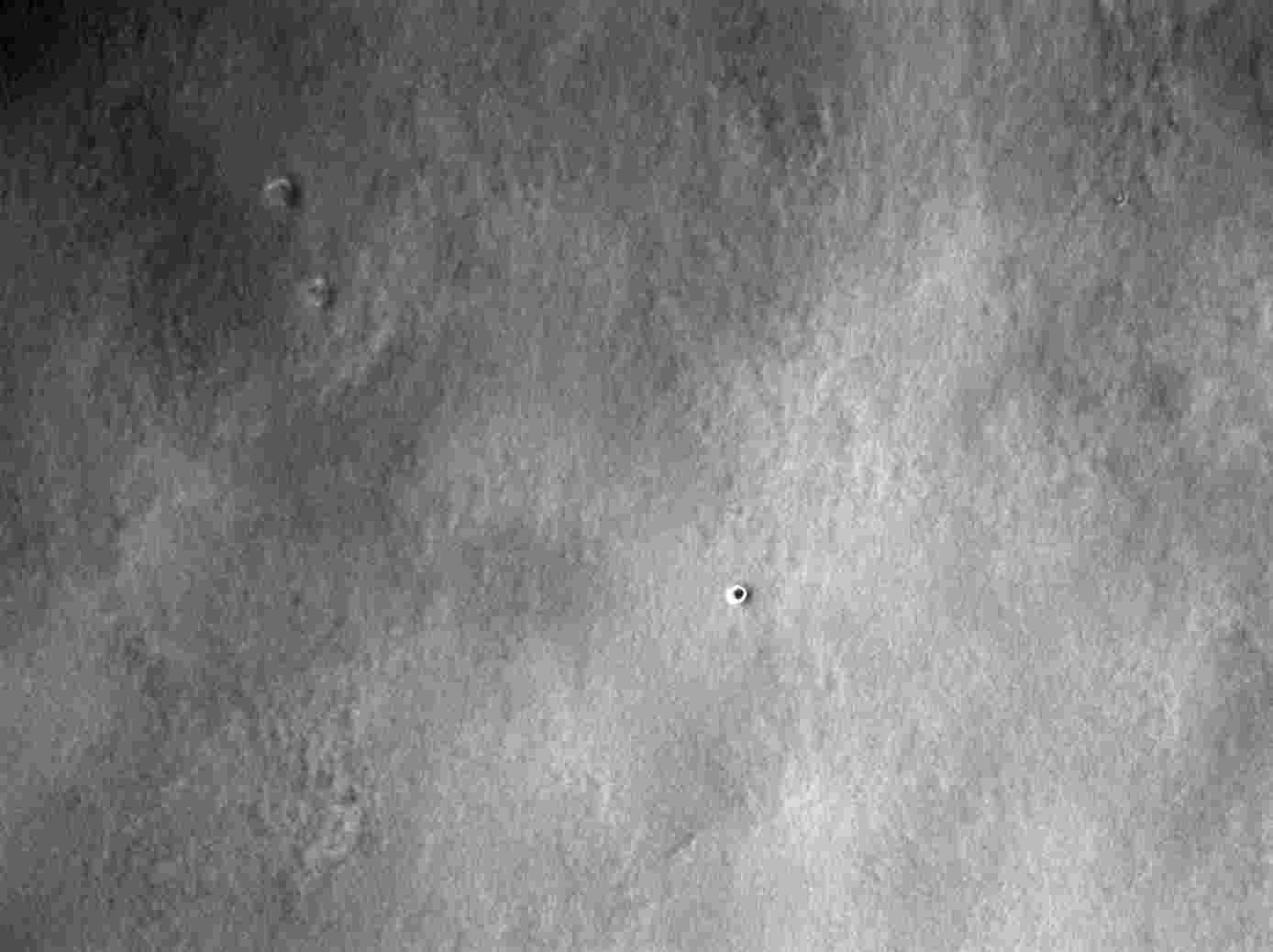

Supplement: S2 File — The raw data are presented in Raw data.zip. (ZIP) [file pone.0339611.s002.zip › Raw data/Figure 4/soft agar/day 1/4+OE-day1 (8).jpg]

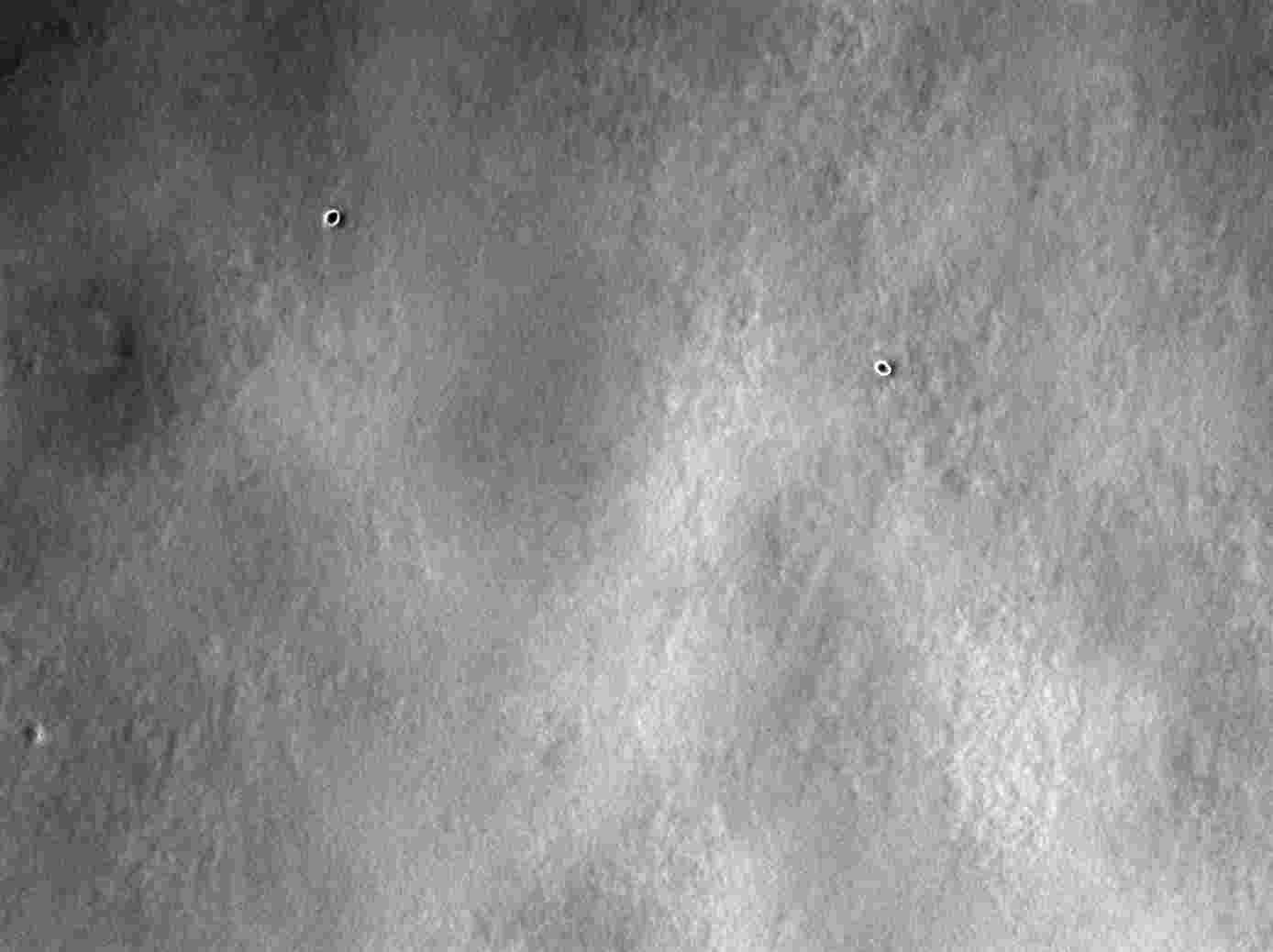

Supplement: S2 File — The raw data are presented in Raw data.zip. (ZIP) [file pone.0339611.s002.zip › Raw data/Figure 4/soft agar/day 1/4+OE-day1 (9).jpg]

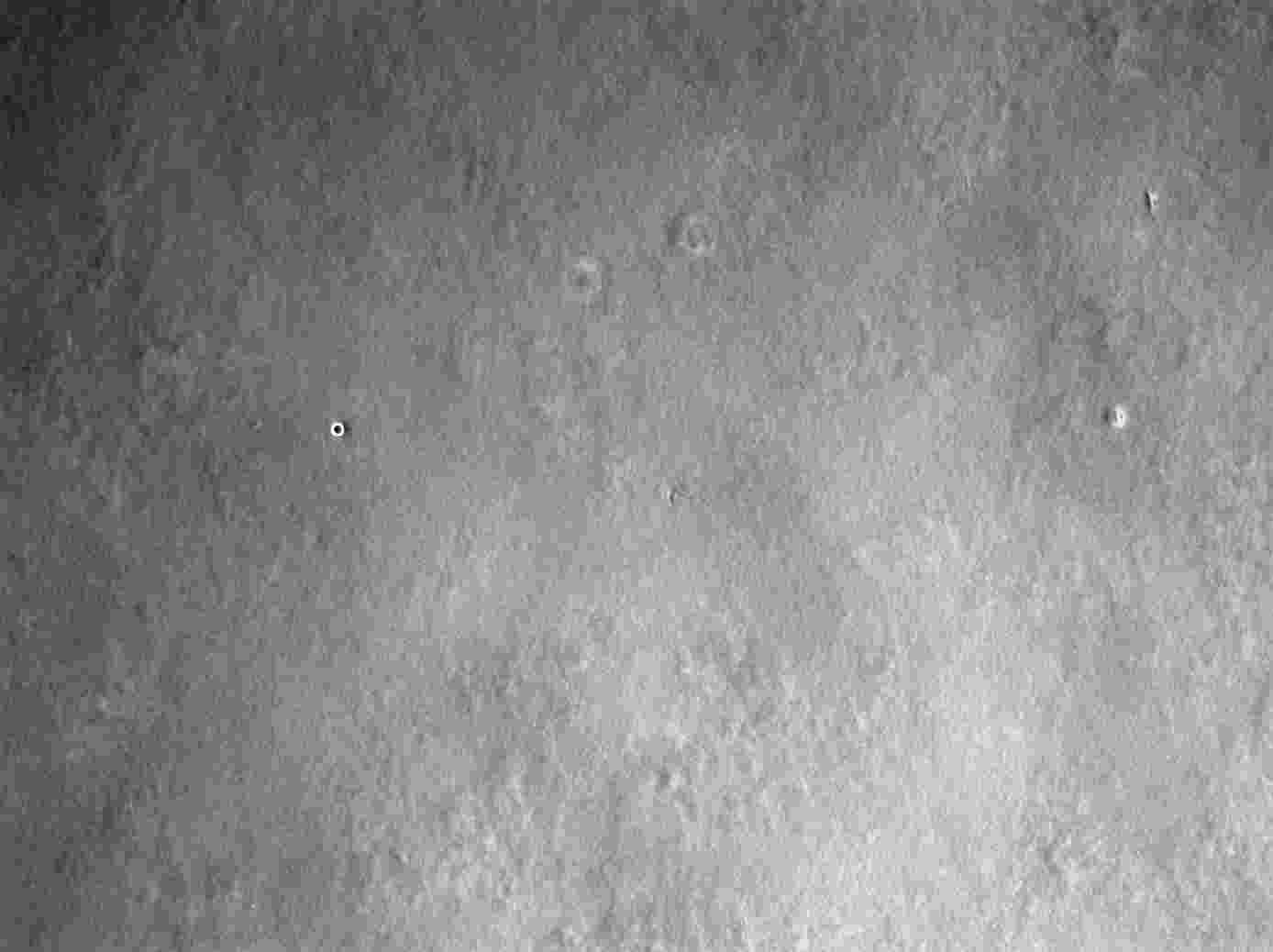

Supplement: S2 File — The raw data are presented in Raw data.zip. (ZIP) [file pone.0339611.s002.zip › Raw data/Figure 4/soft agar/day 1/4+OE-day1.jpg]

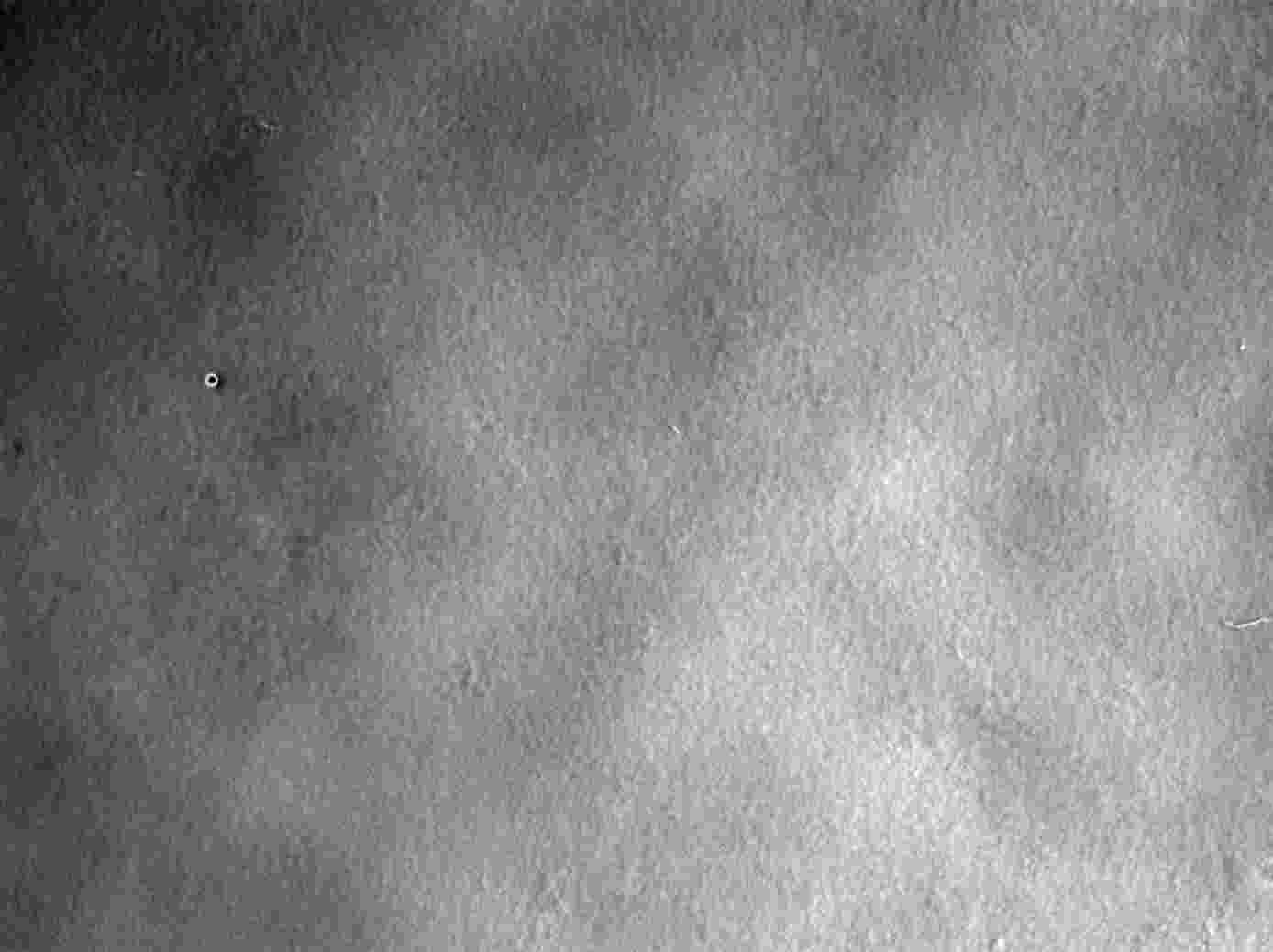

Supplement: S2 File — The raw data are presented in Raw data.zip. (ZIP) [file pone.0339611.s002.zip › Raw data/Figure 4/soft agar/day 1/5+EV-day1 (10).jpg]

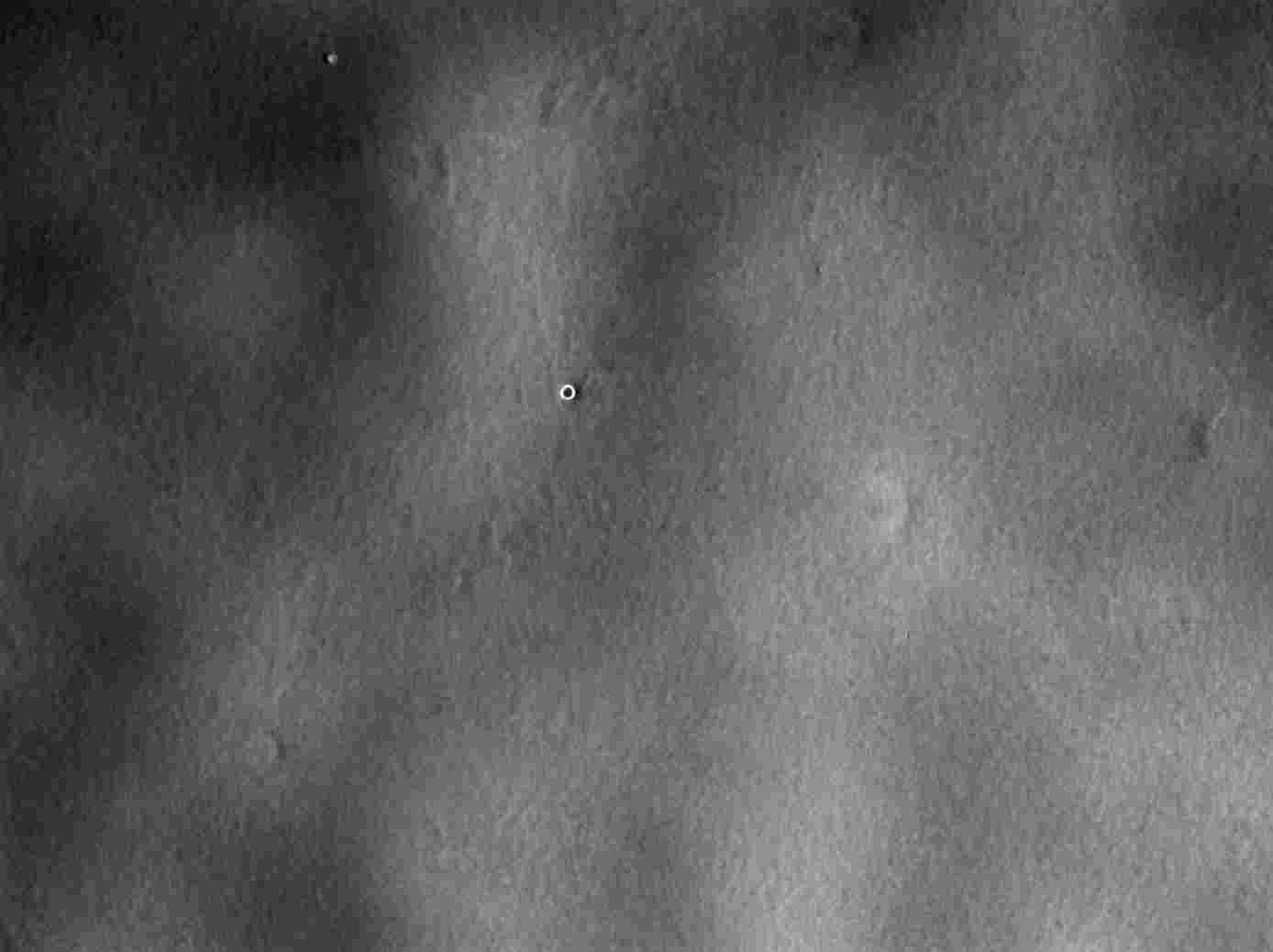

Supplement: S2 File — The raw data are presented in Raw data.zip. (ZIP) [file pone.0339611.s002.zip › Raw data/Figure 4/soft agar/day 1/5+EV-day1 (11).jpg]

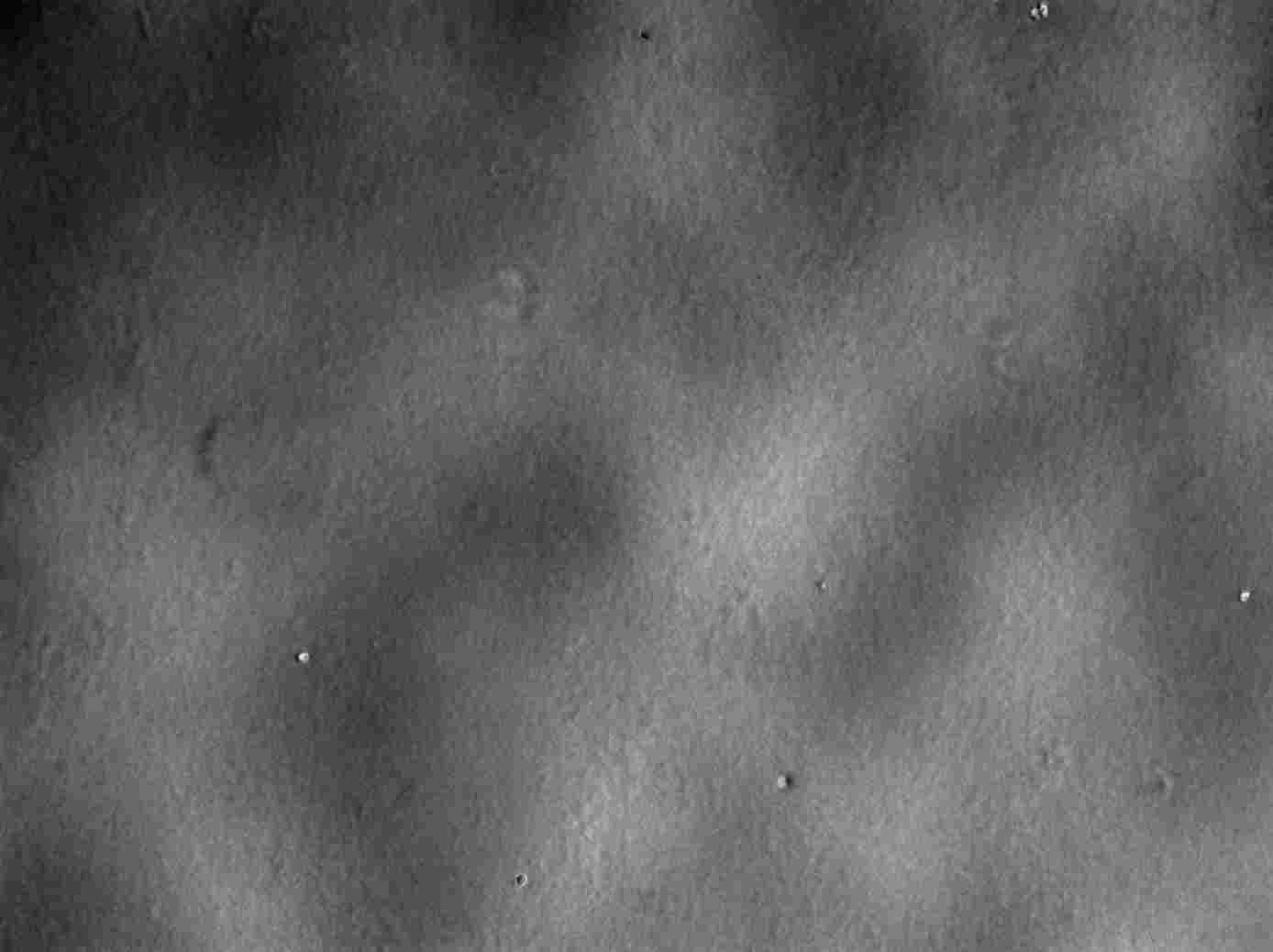

Supplement: S2 File — The raw data are presented in Raw data.zip. (ZIP) [file pone.0339611.s002.zip › Raw data/Figure 4/soft agar/day 1/5+EV-day1 (12).jpg]

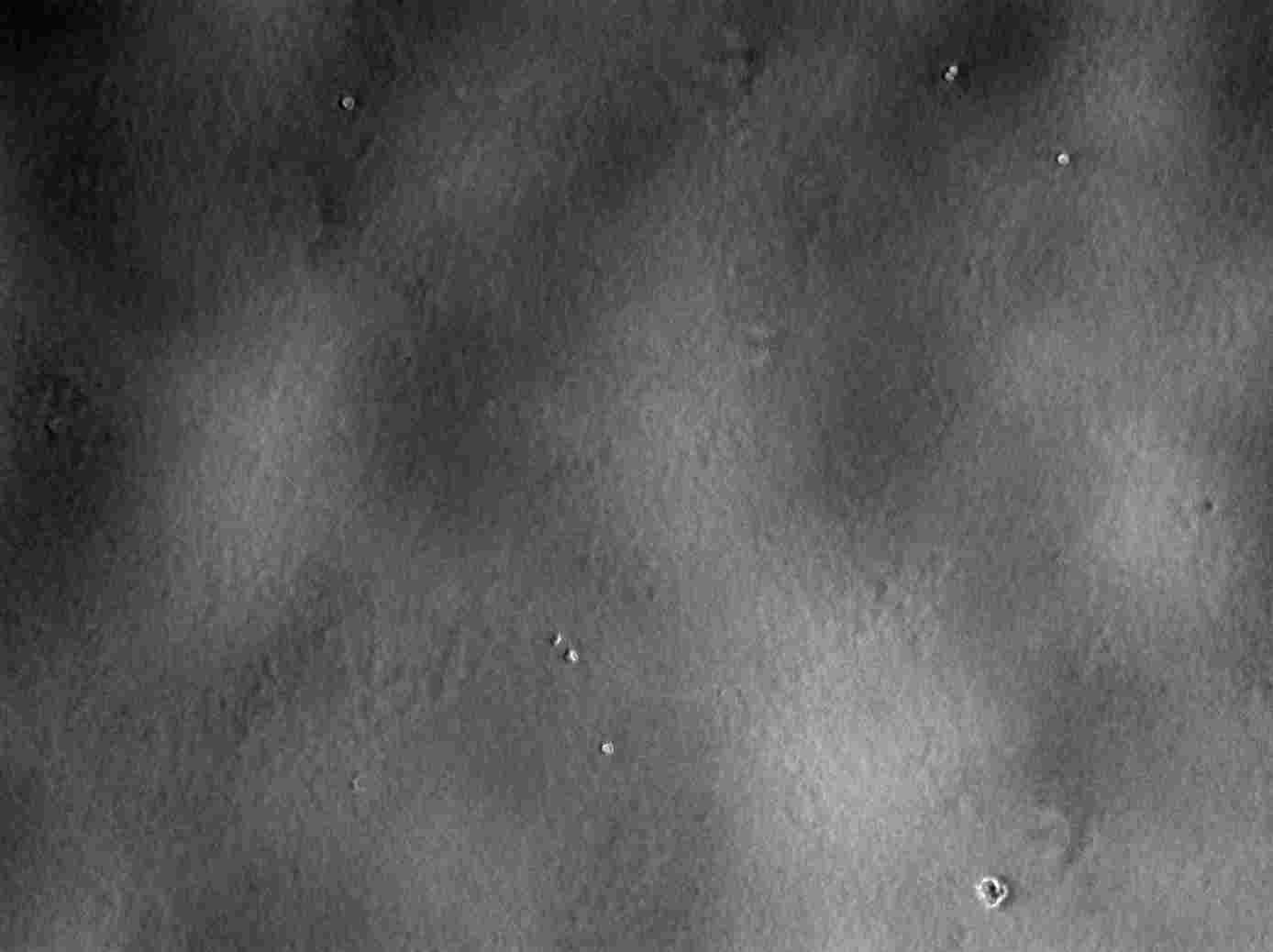

Supplement: S2 File — The raw data are presented in Raw data.zip. (ZIP) [file pone.0339611.s002.zip › Raw data/Figure 4/soft agar/day 1/5+EV-day1 (13).jpg]
