# Supplementary figures and images for: Loss of IL1RA promotes prostate cancer growth and metastasis by activating Akt signaling pathway (part 2 of 2)
Source: PLoS One. 2026 Feb 2;21(2):e0339611. doi: 10.1371/journal.pone.0339611 (PMC12863537; doi:10.1371/journal.pone.0339611)

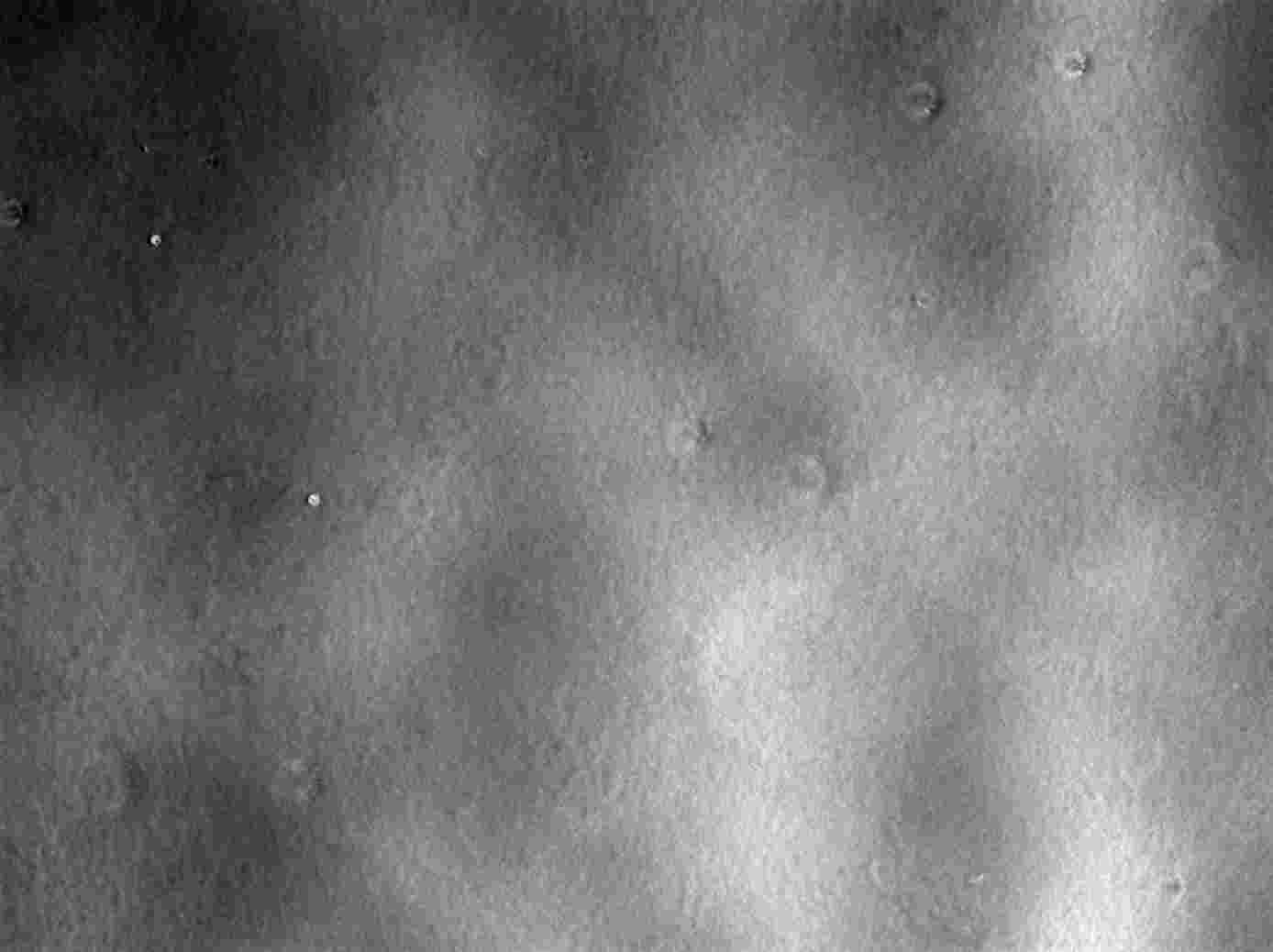

Supplement: S2 File — The raw data are presented in Raw data.zip. (ZIP) [file pone.0339611.s002.zip › Raw data/Figure 4/soft agar/day 1/5+EV-day1 (14).jpg]

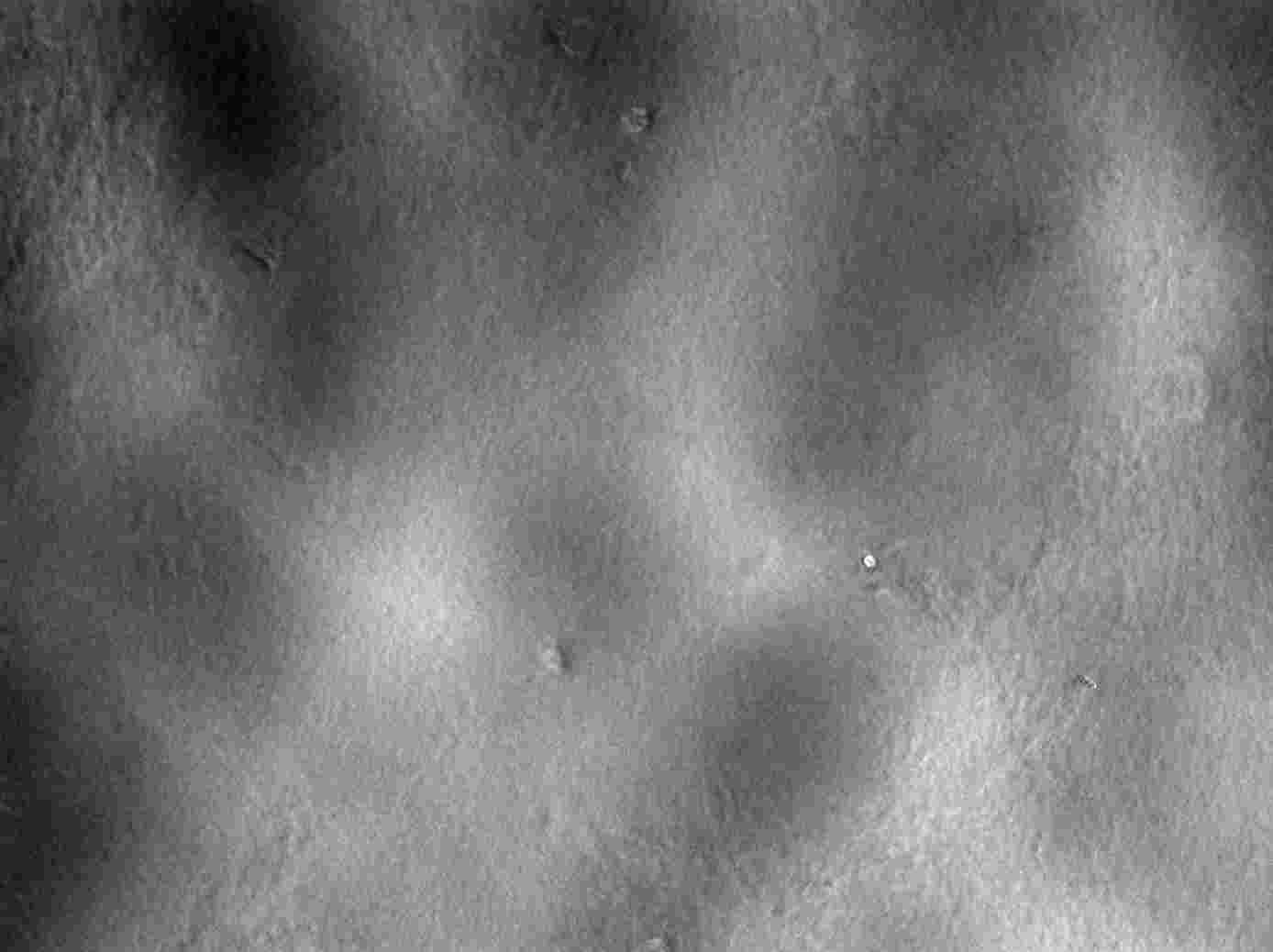

Supplement: S2 File — The raw data are presented in Raw data.zip. (ZIP) [file pone.0339611.s002.zip › Raw data/Figure 4/soft agar/day 1/5+EV-day1 (15).jpg]

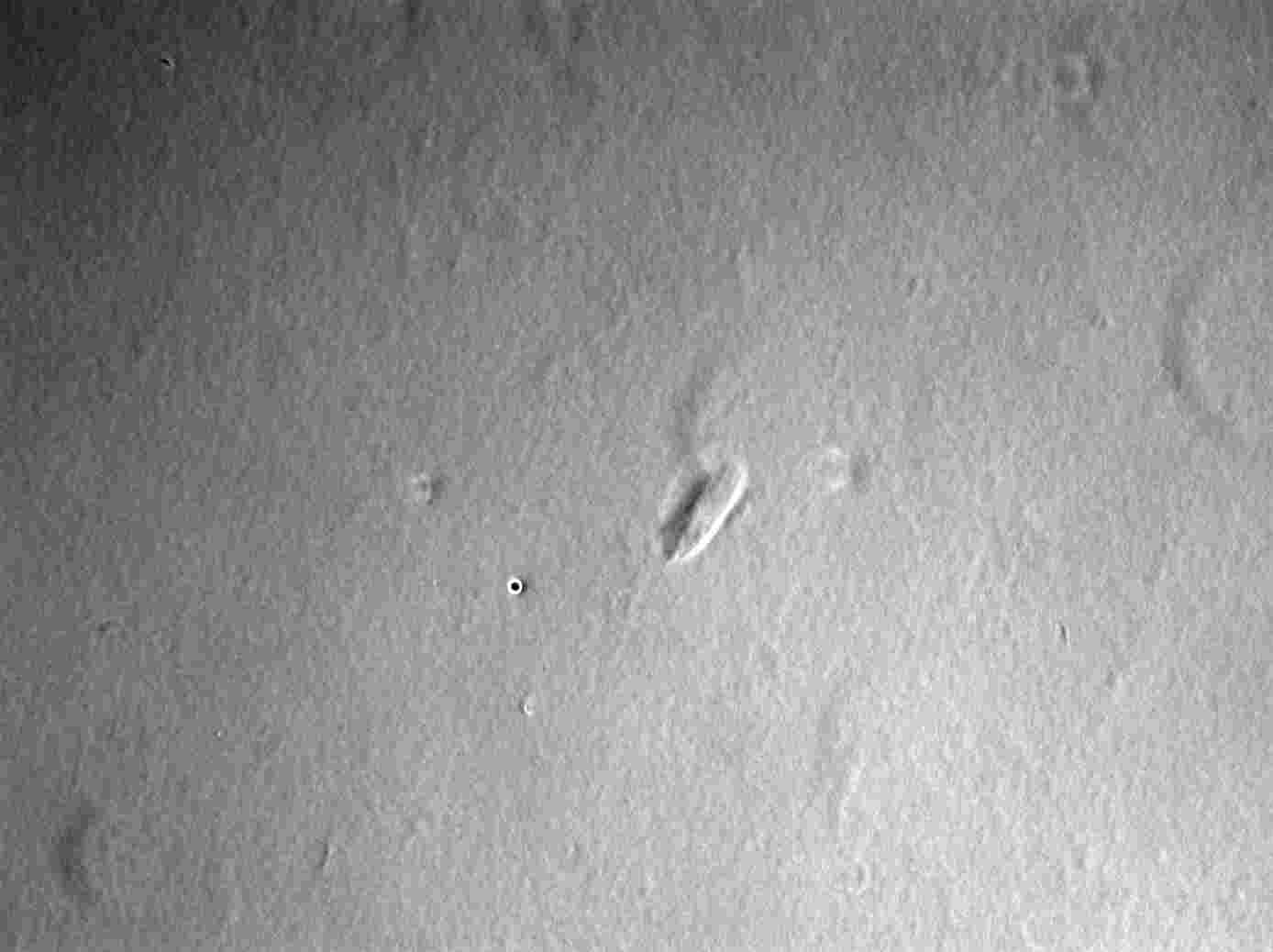

Supplement: S2 File — The raw data are presented in Raw data.zip. (ZIP) [file pone.0339611.s002.zip › Raw data/Figure 4/soft agar/day 1/5+EV-day1 (2).jpg]

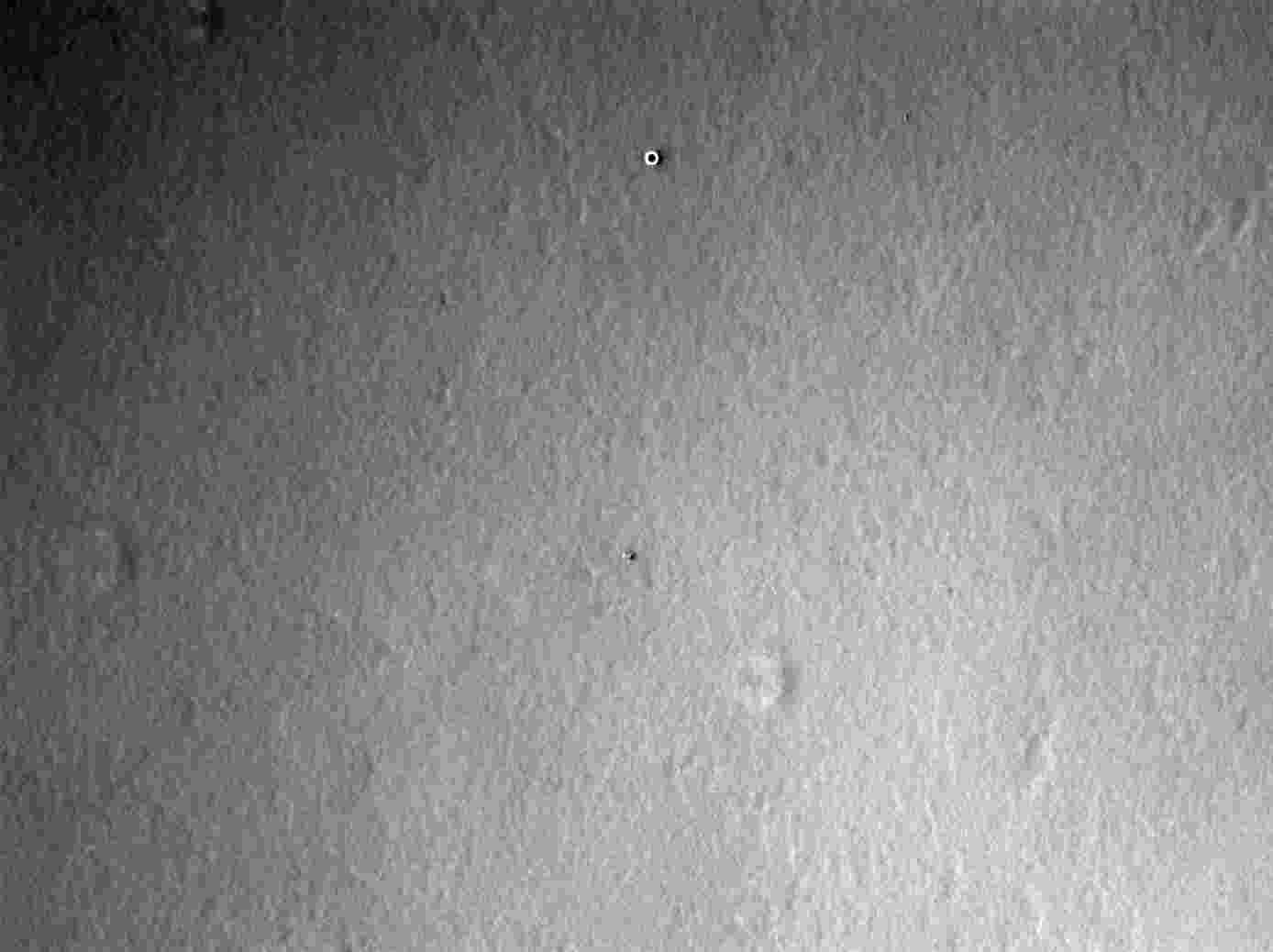

Supplement: S2 File — The raw data are presented in Raw data.zip. (ZIP) [file pone.0339611.s002.zip › Raw data/Figure 4/soft agar/day 1/5+EV-day1 (3).jpg]

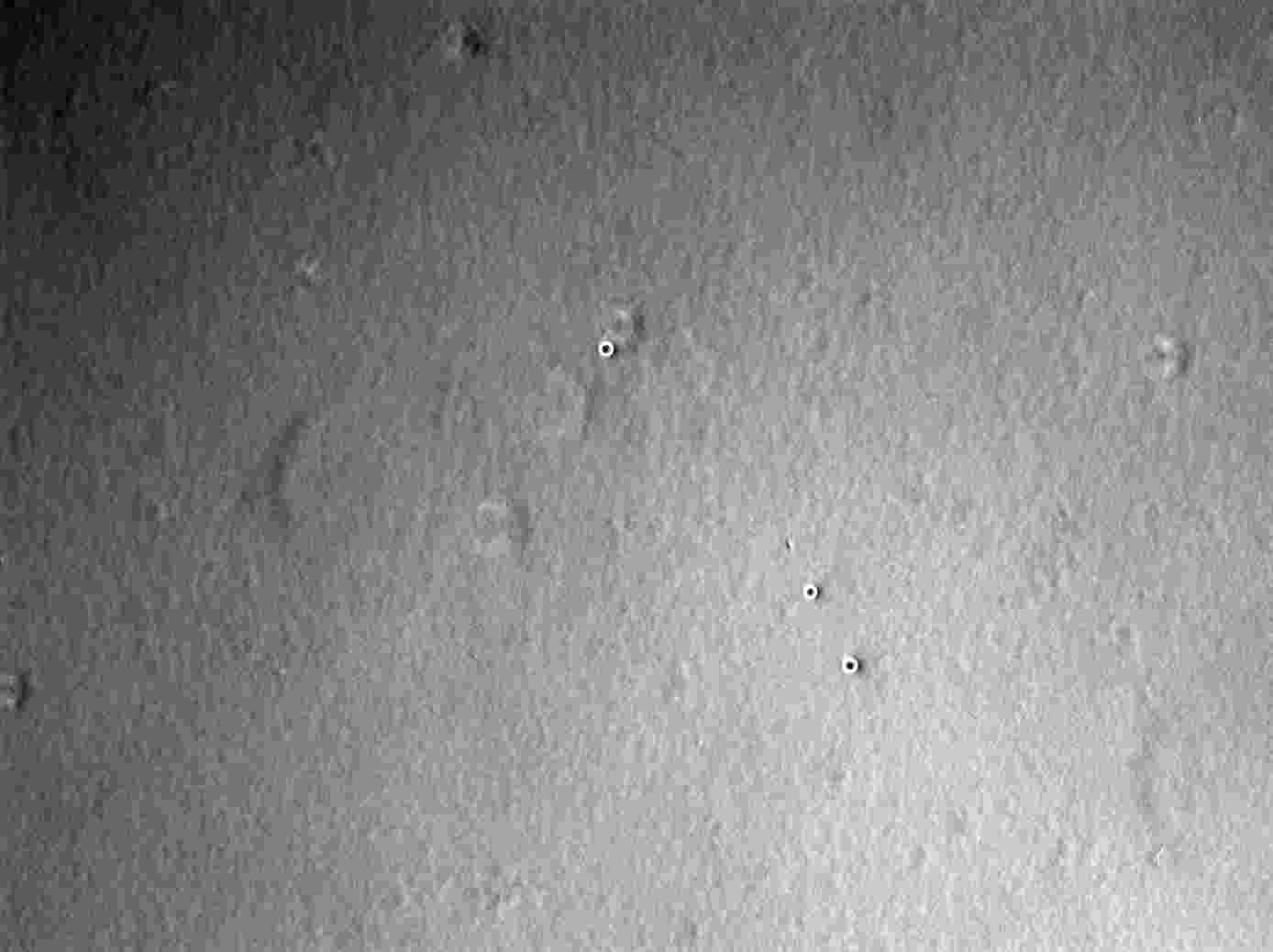

Supplement: S2 File — The raw data are presented in Raw data.zip. (ZIP) [file pone.0339611.s002.zip › Raw data/Figure 4/soft agar/day 1/5+EV-day1 (4).jpg]

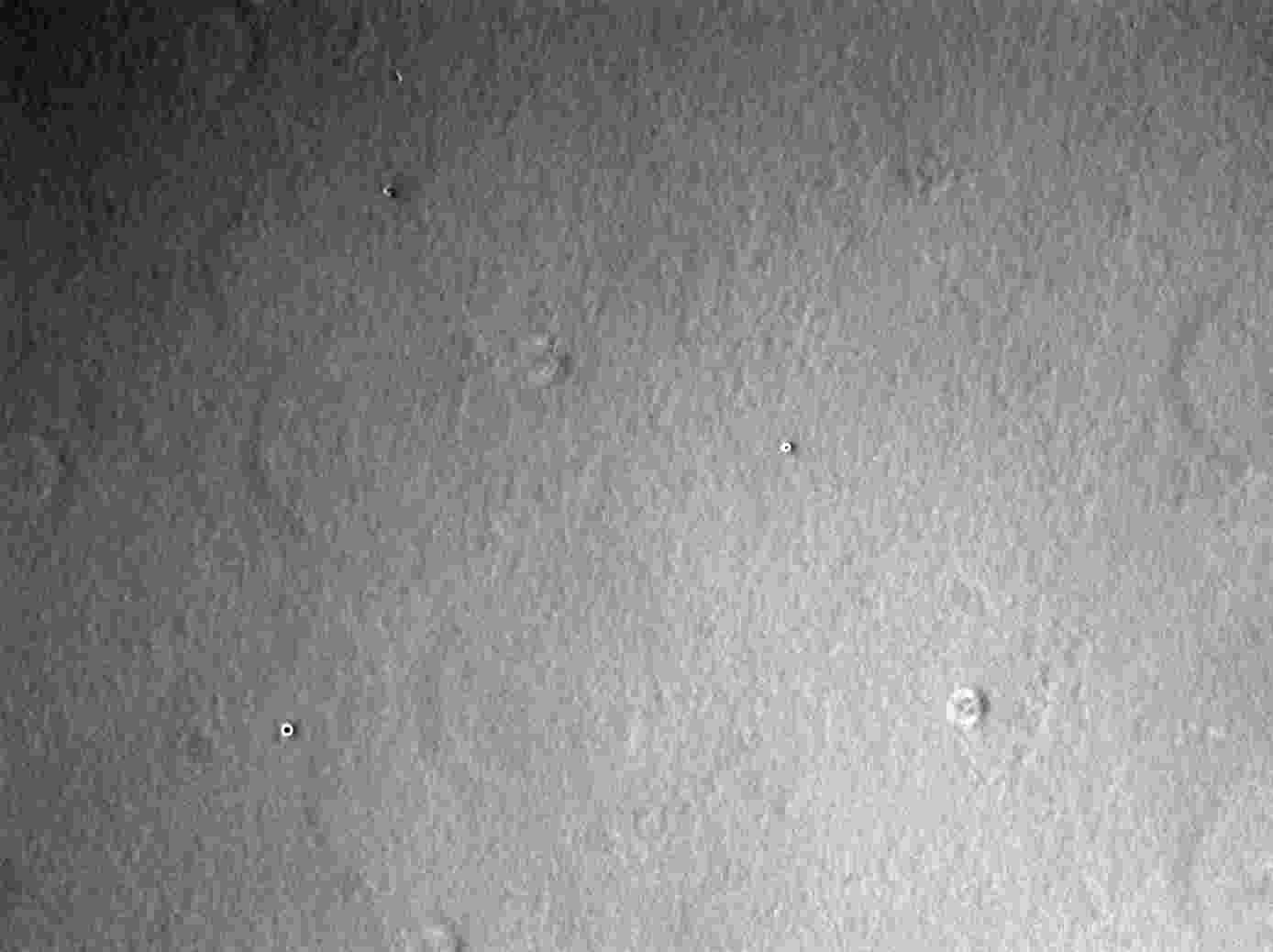

Supplement: S2 File — The raw data are presented in Raw data.zip. (ZIP) [file pone.0339611.s002.zip › Raw data/Figure 4/soft agar/day 1/5+EV-day1 (5).jpg]

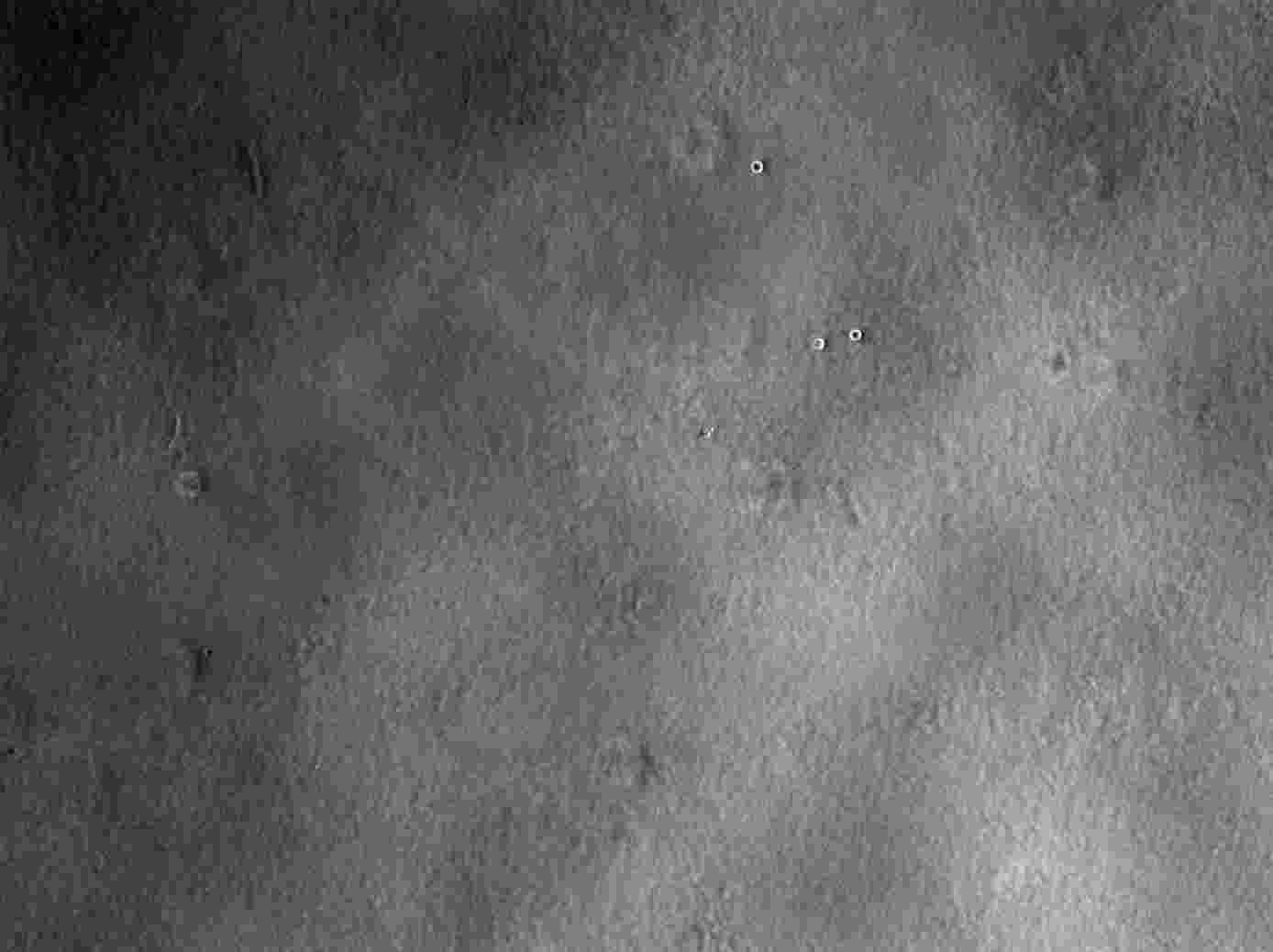

Supplement: S2 File — The raw data are presented in Raw data.zip. (ZIP) [file pone.0339611.s002.zip › Raw data/Figure 4/soft agar/day 1/5+EV-day1 (6).jpg]

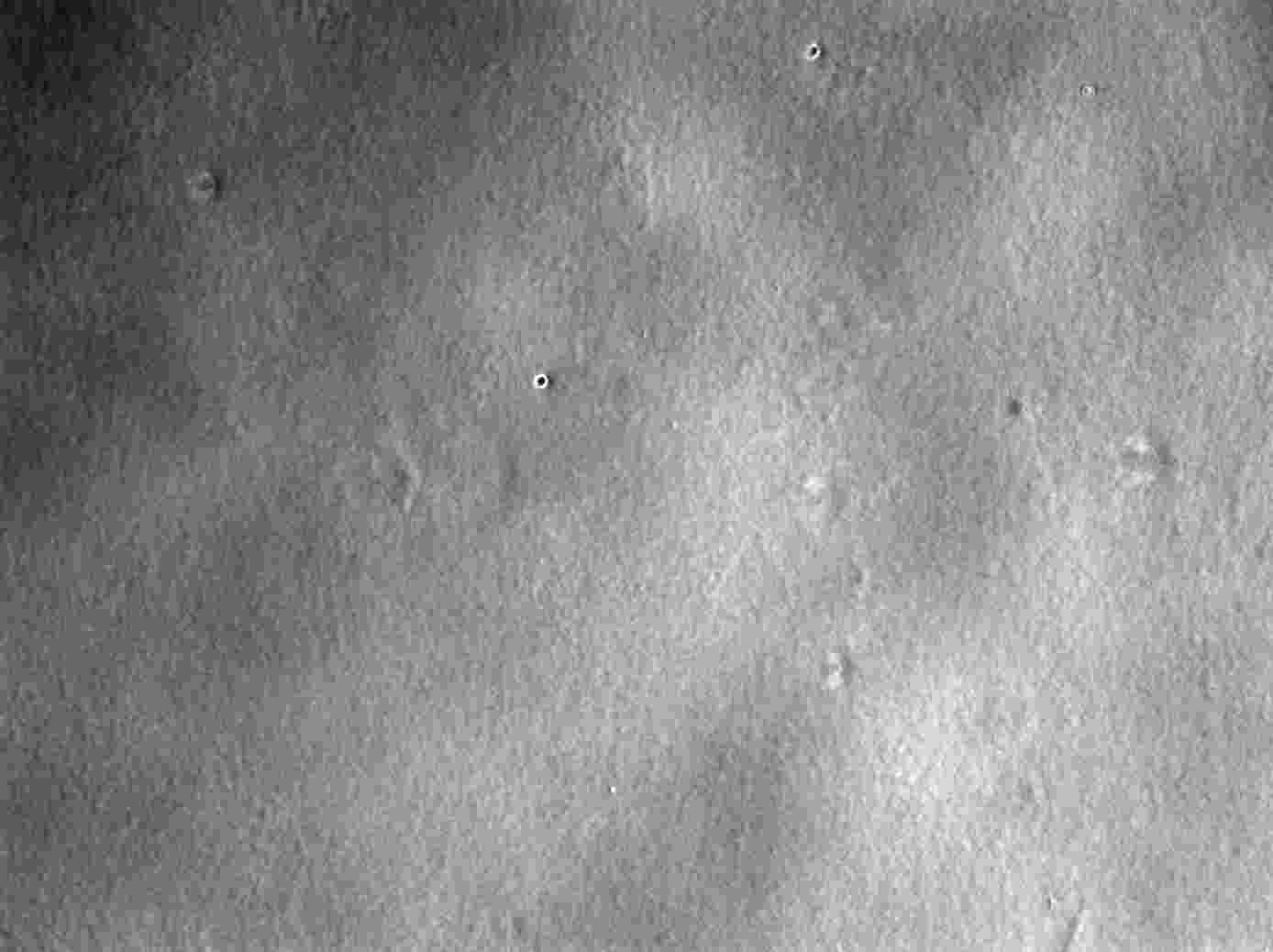

Supplement: S2 File — The raw data are presented in Raw data.zip. (ZIP) [file pone.0339611.s002.zip › Raw data/Figure 4/soft agar/day 1/5+EV-day1 (7).jpg]

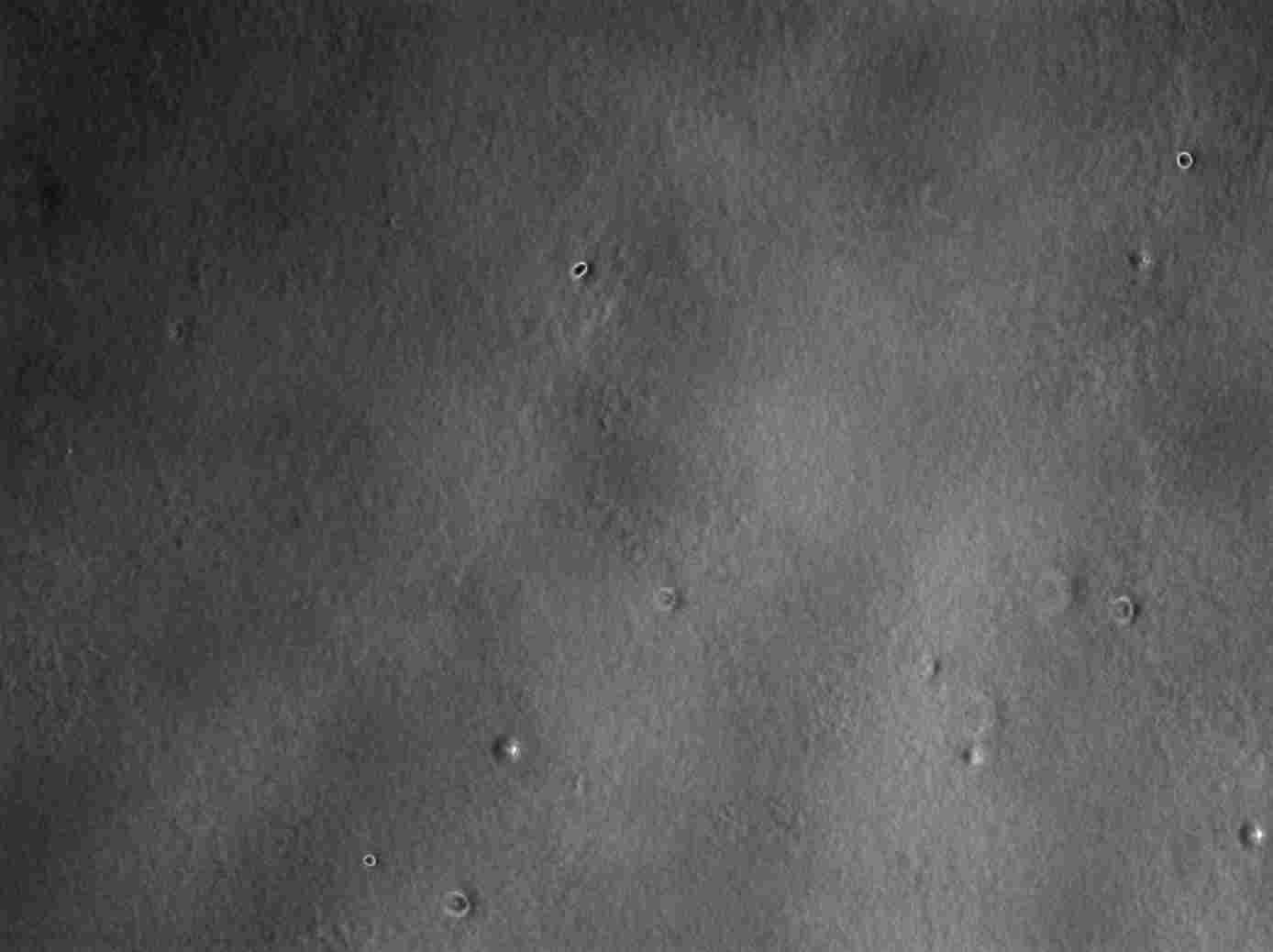

Supplement: S2 File — The raw data are presented in Raw data.zip. (ZIP) [file pone.0339611.s002.zip › Raw data/Figure 4/soft agar/day 1/5+EV-day1 (8).jpg]

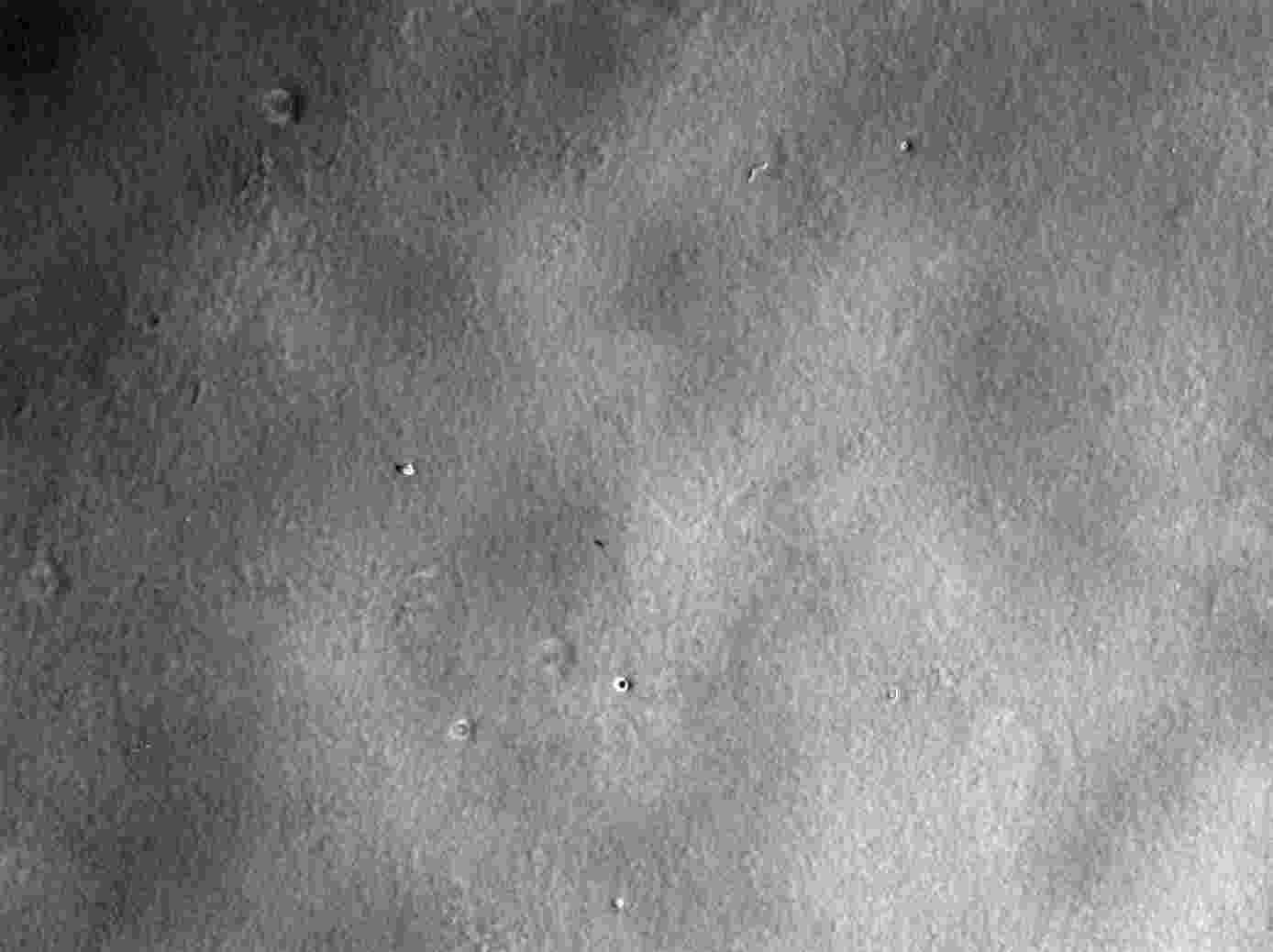

Supplement: S2 File — The raw data are presented in Raw data.zip. (ZIP) [file pone.0339611.s002.zip › Raw data/Figure 4/soft agar/day 1/5+EV-day1 (9).jpg]

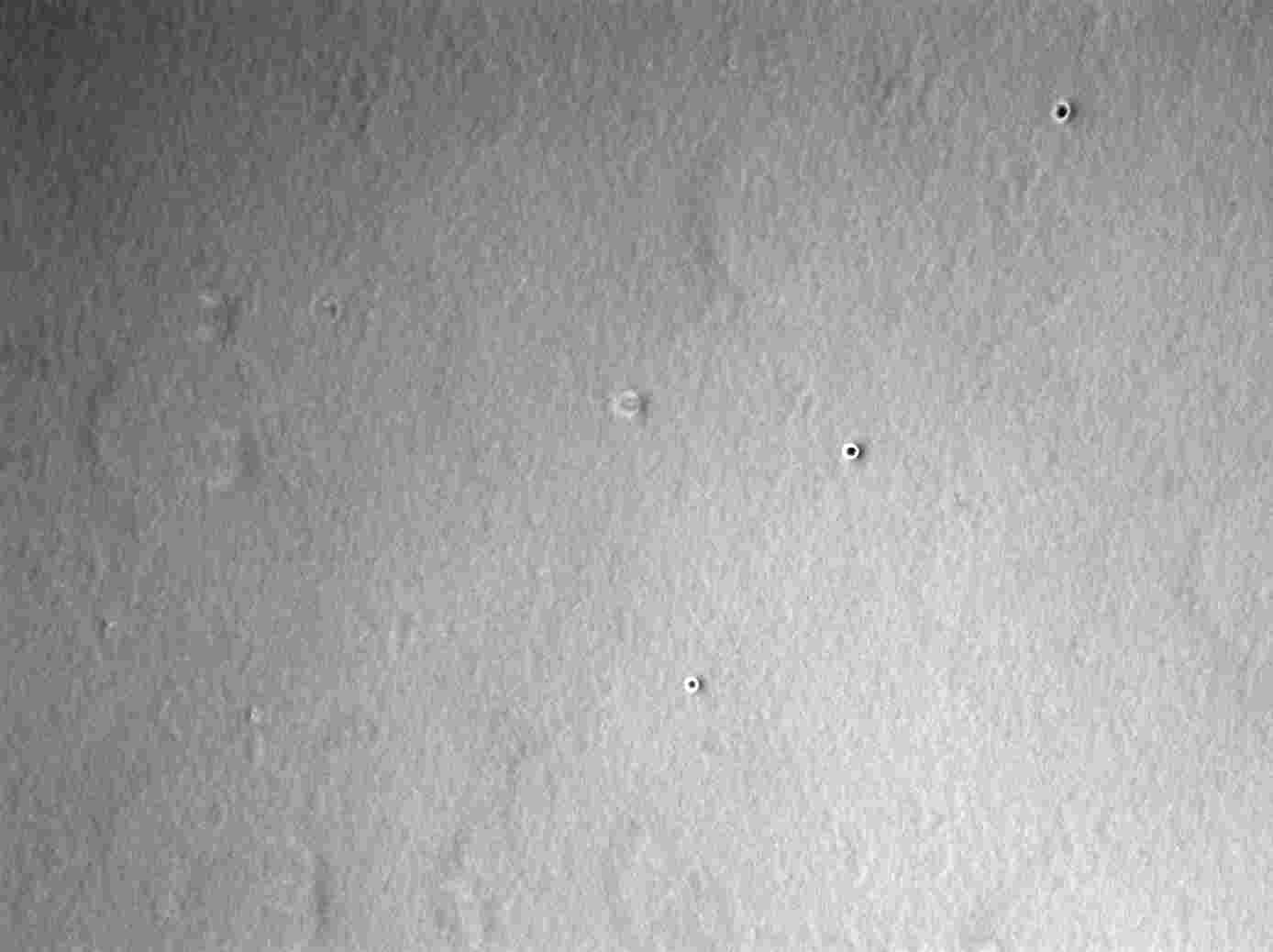

Supplement: S2 File — The raw data are presented in Raw data.zip. (ZIP) [file pone.0339611.s002.zip › Raw data/Figure 4/soft agar/day 1/5+EV-day1.jpg]

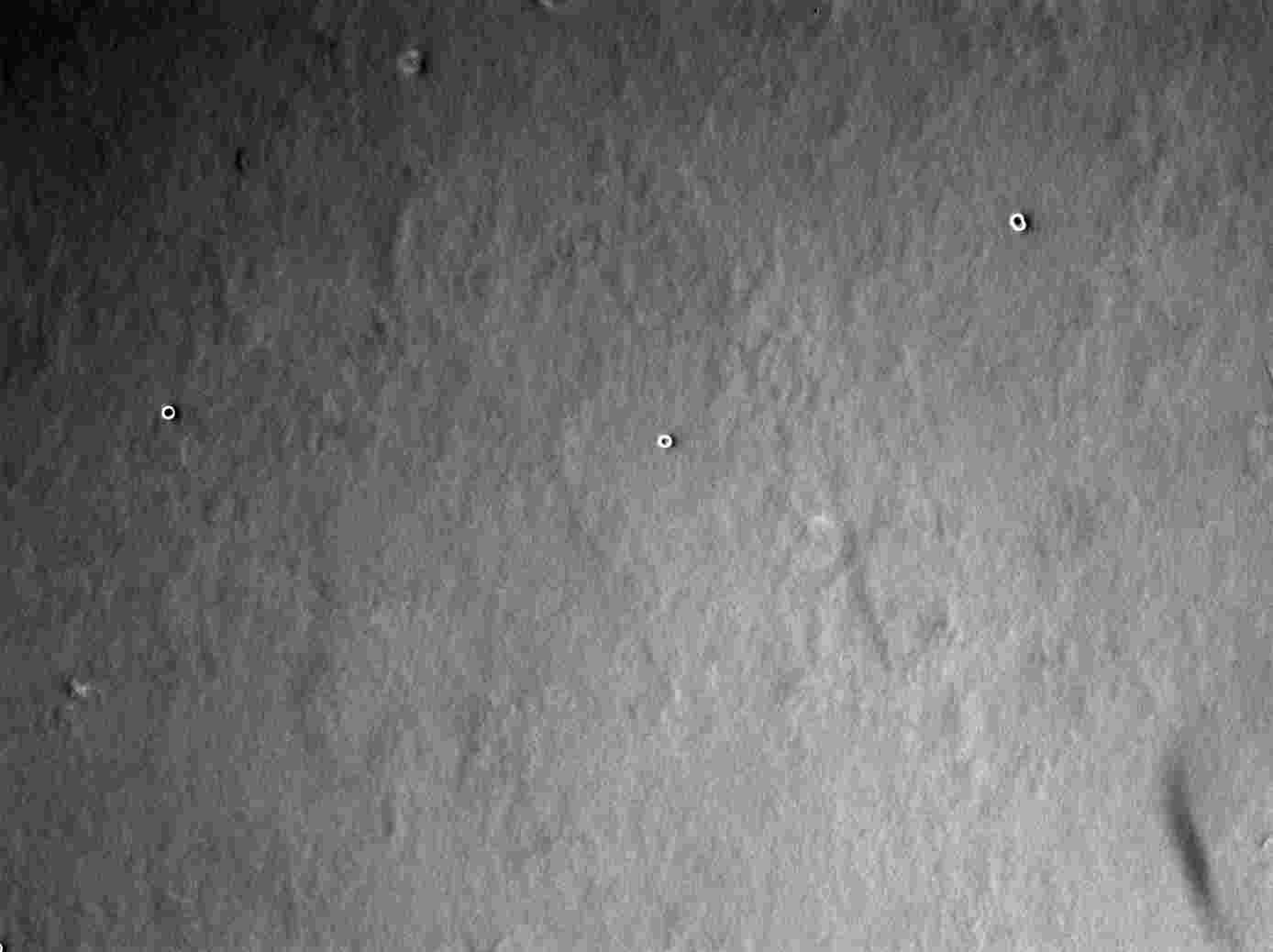

Supplement: S2 File — The raw data are presented in Raw data.zip. (ZIP) [file pone.0339611.s002.zip › Raw data/Figure 4/soft agar/day 1/5+OE-day1 (10).jpg]

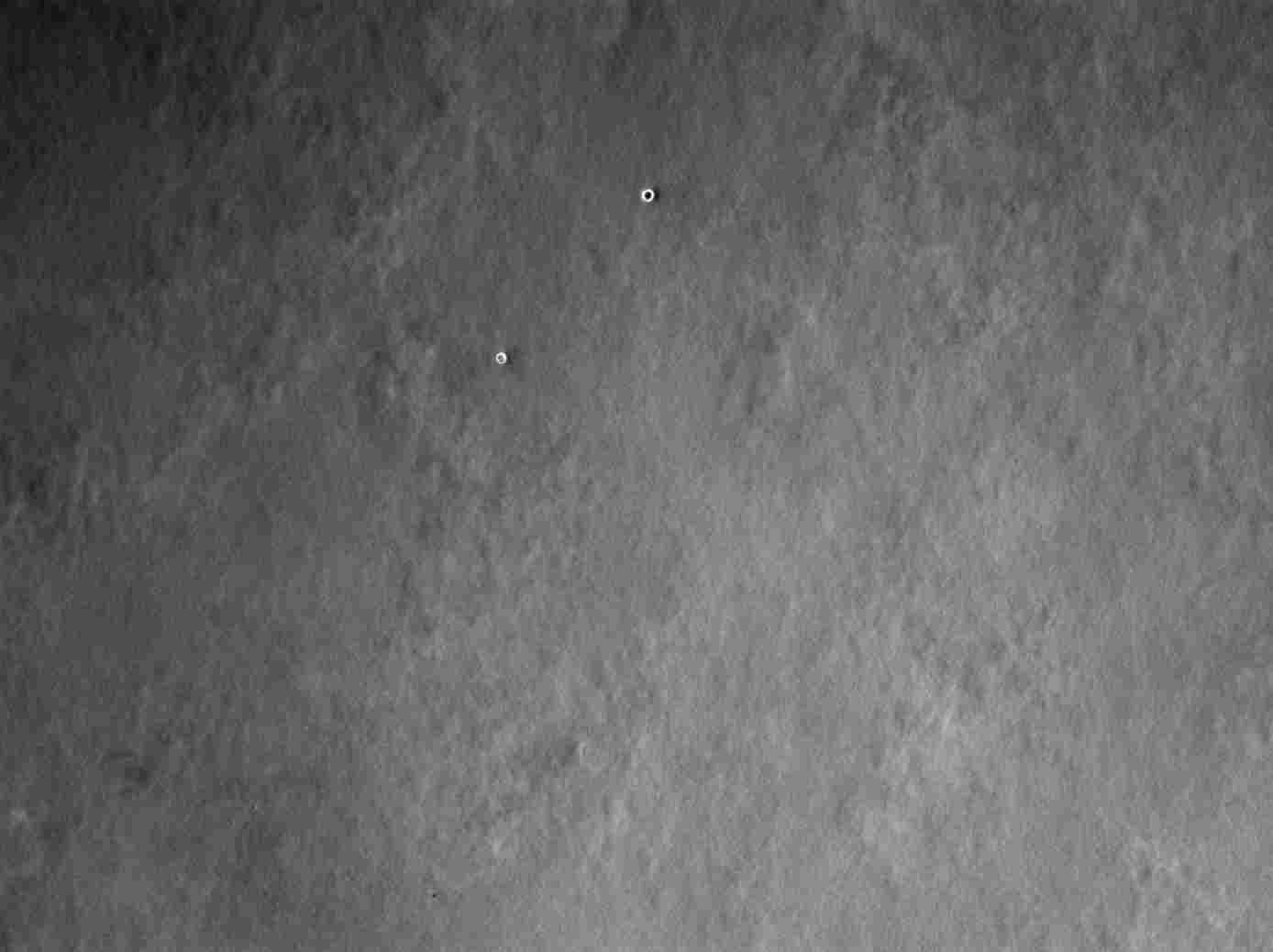

Supplement: S2 File — The raw data are presented in Raw data.zip. (ZIP) [file pone.0339611.s002.zip › Raw data/Figure 4/soft agar/day 1/5+OE-day1 (11).jpg]

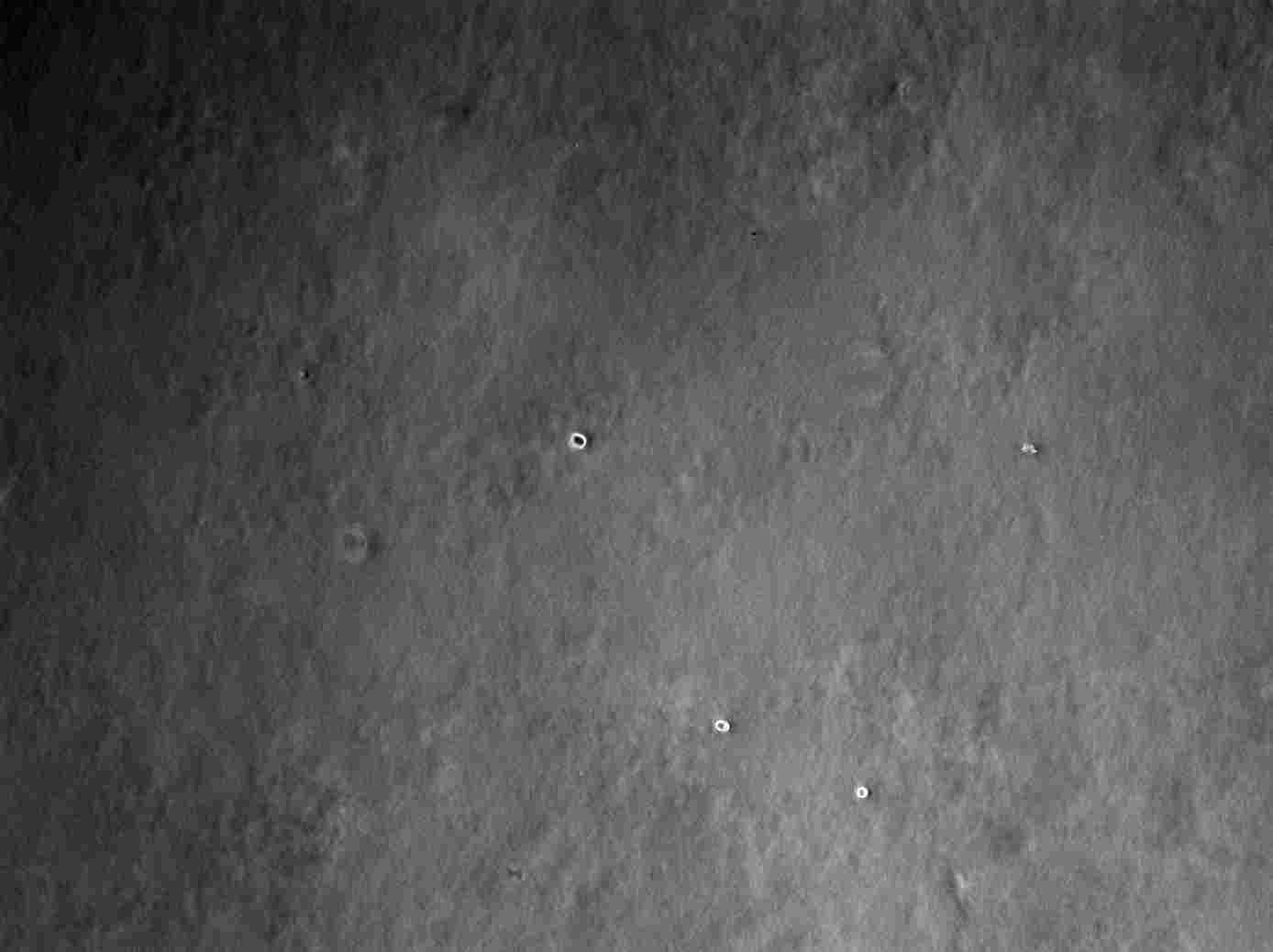

Supplement: S2 File — The raw data are presented in Raw data.zip. (ZIP) [file pone.0339611.s002.zip › Raw data/Figure 4/soft agar/day 1/5+OE-day1 (12).jpg]

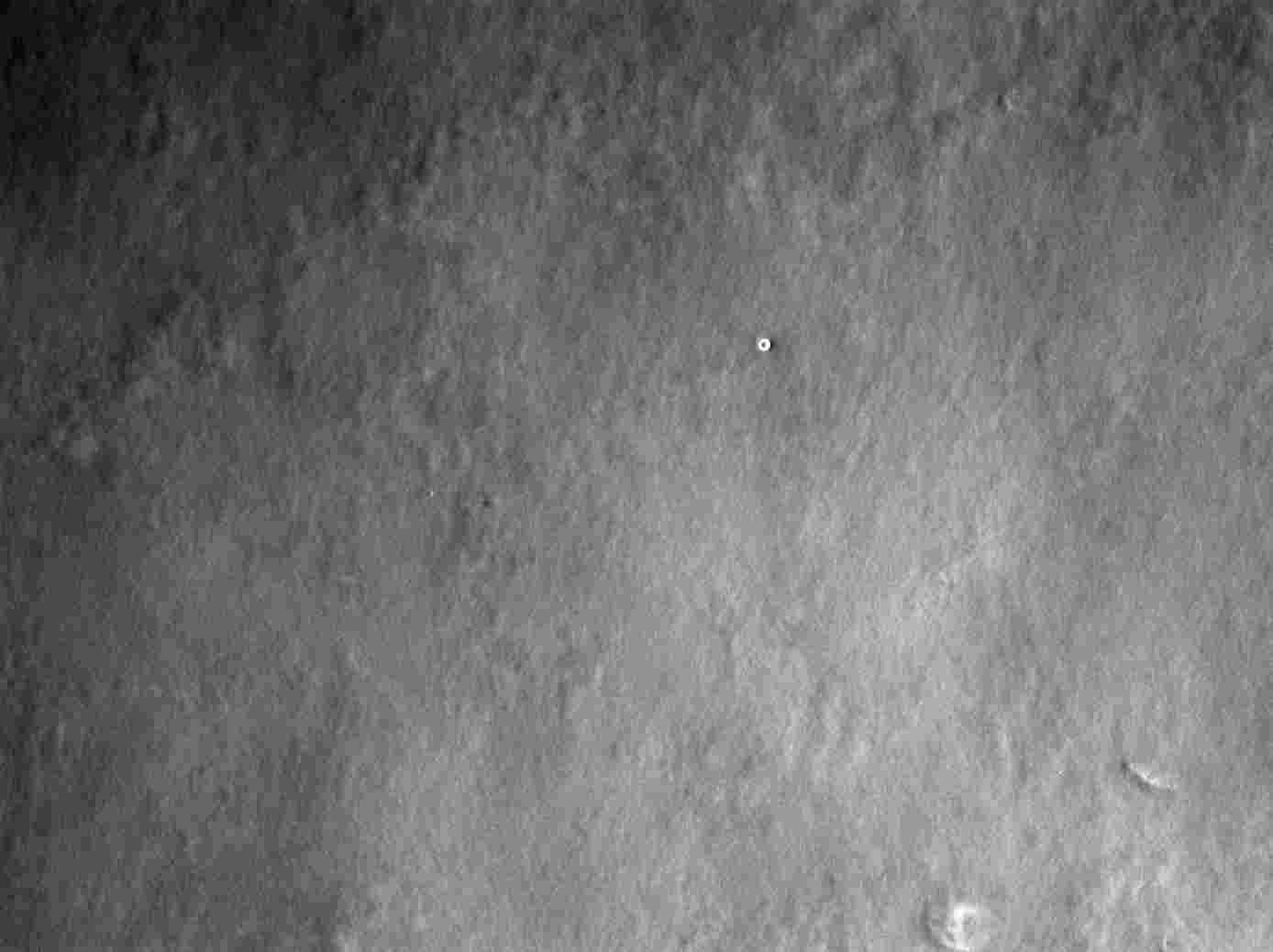

Supplement: S2 File — The raw data are presented in Raw data.zip. (ZIP) [file pone.0339611.s002.zip › Raw data/Figure 4/soft agar/day 1/5+OE-day1 (13).jpg]

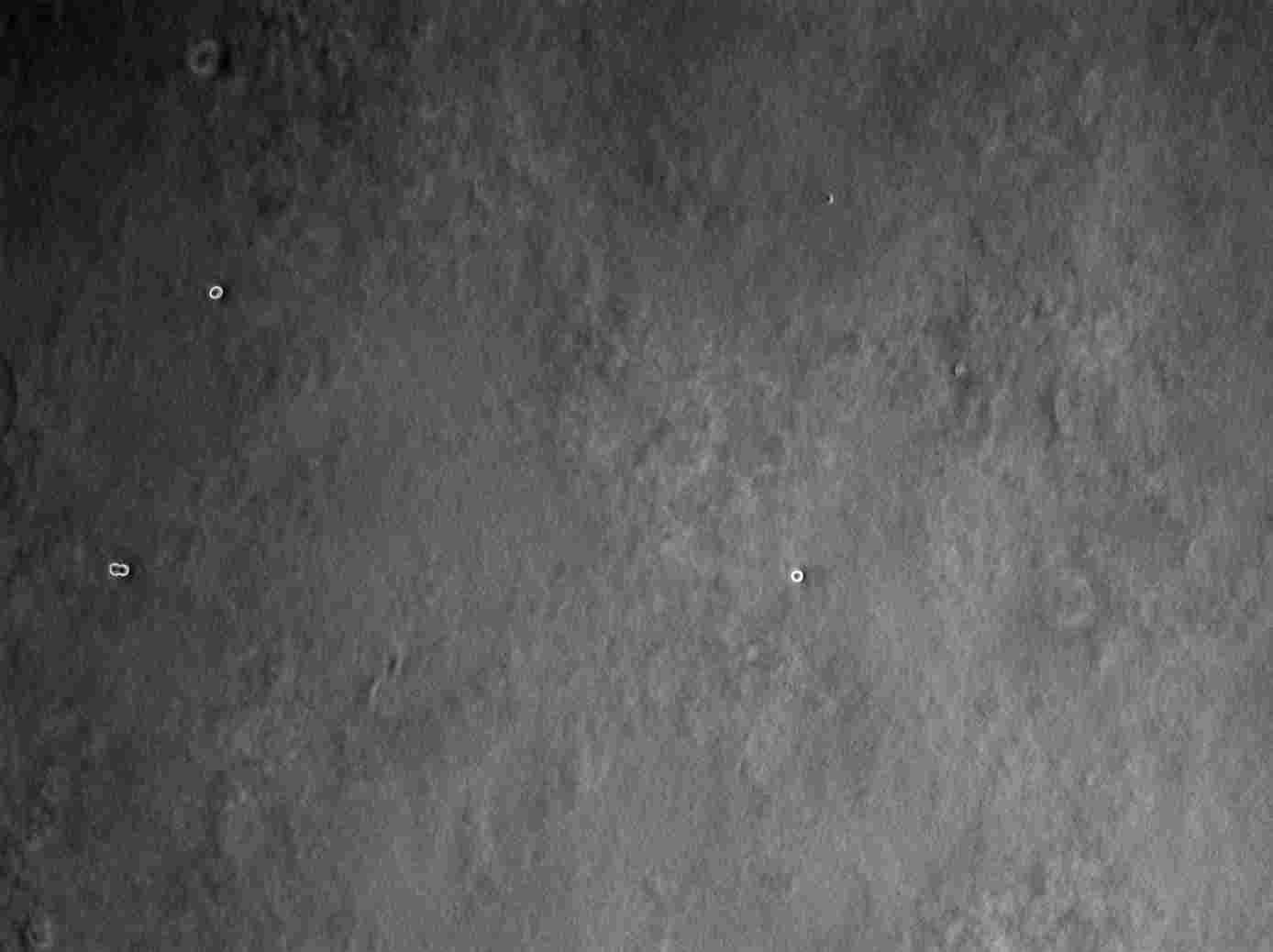

Supplement: S2 File — The raw data are presented in Raw data.zip. (ZIP) [file pone.0339611.s002.zip › Raw data/Figure 4/soft agar/day 1/5+OE-day1 (14).jpg]

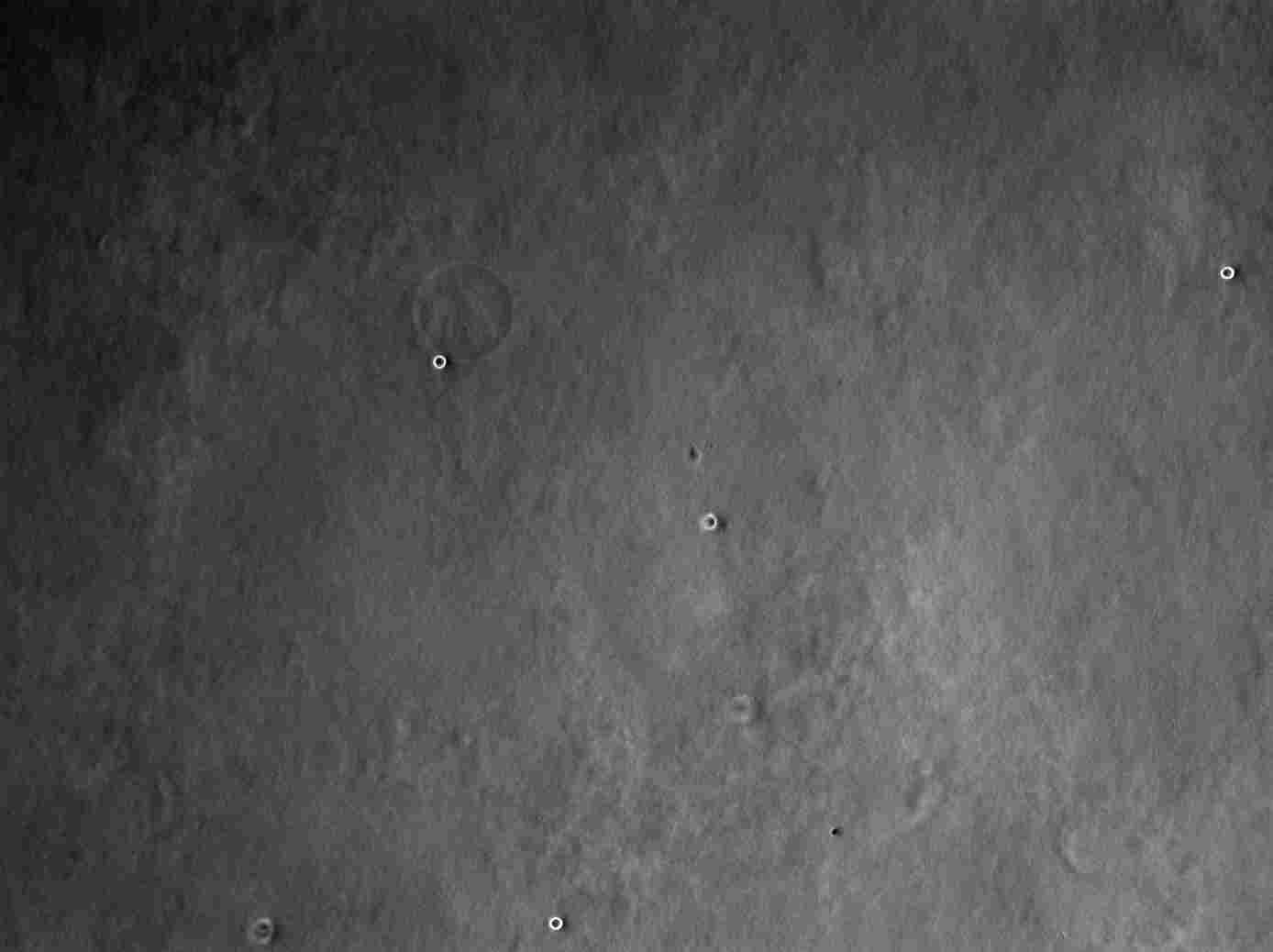

Supplement: S2 File — The raw data are presented in Raw data.zip. (ZIP) [file pone.0339611.s002.zip › Raw data/Figure 4/soft agar/day 1/5+OE-day1 (15).jpg]

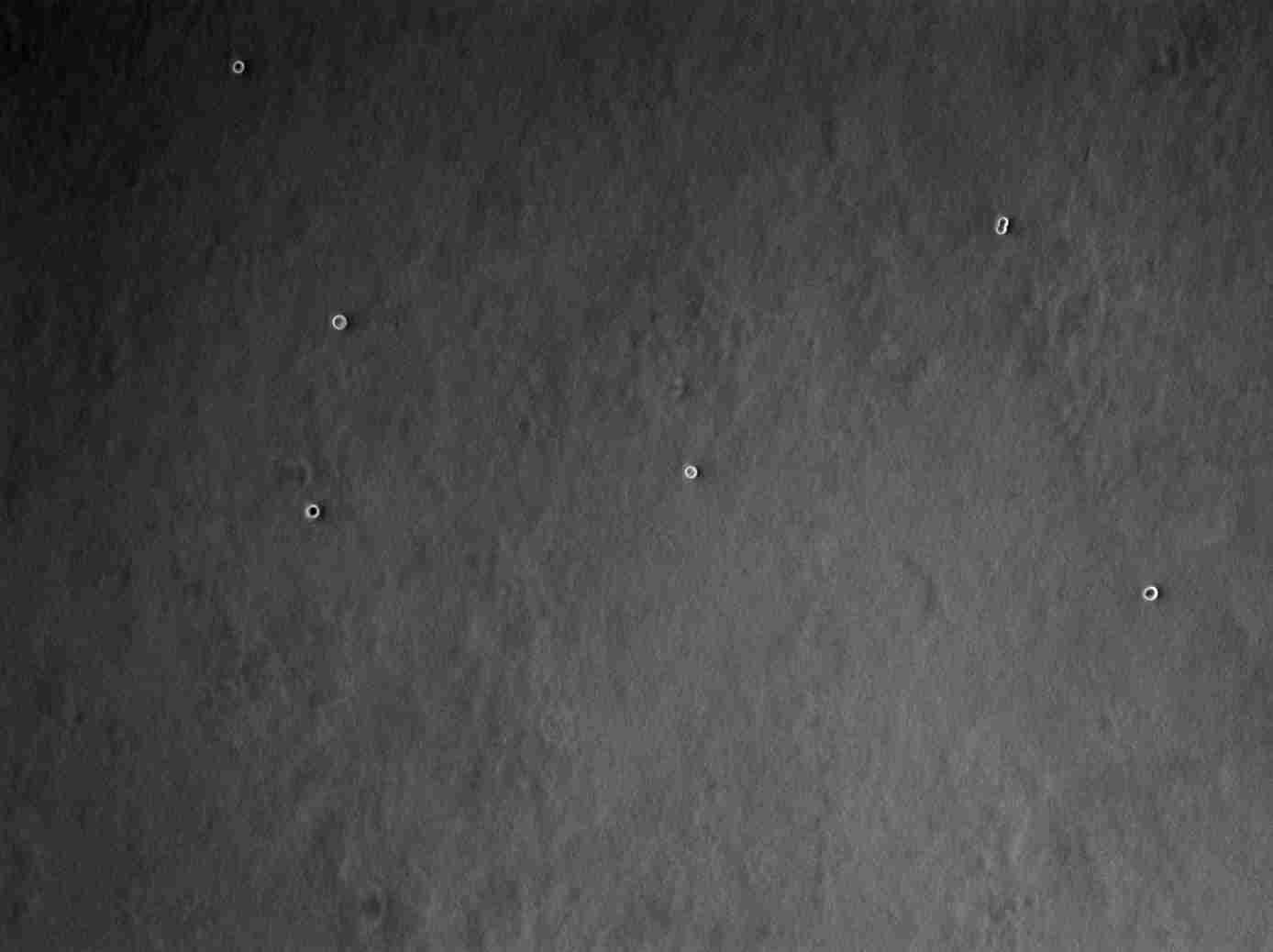

Supplement: S2 File — The raw data are presented in Raw data.zip. (ZIP) [file pone.0339611.s002.zip › Raw data/Figure 4/soft agar/day 1/5+OE-day1 (2).jpg]

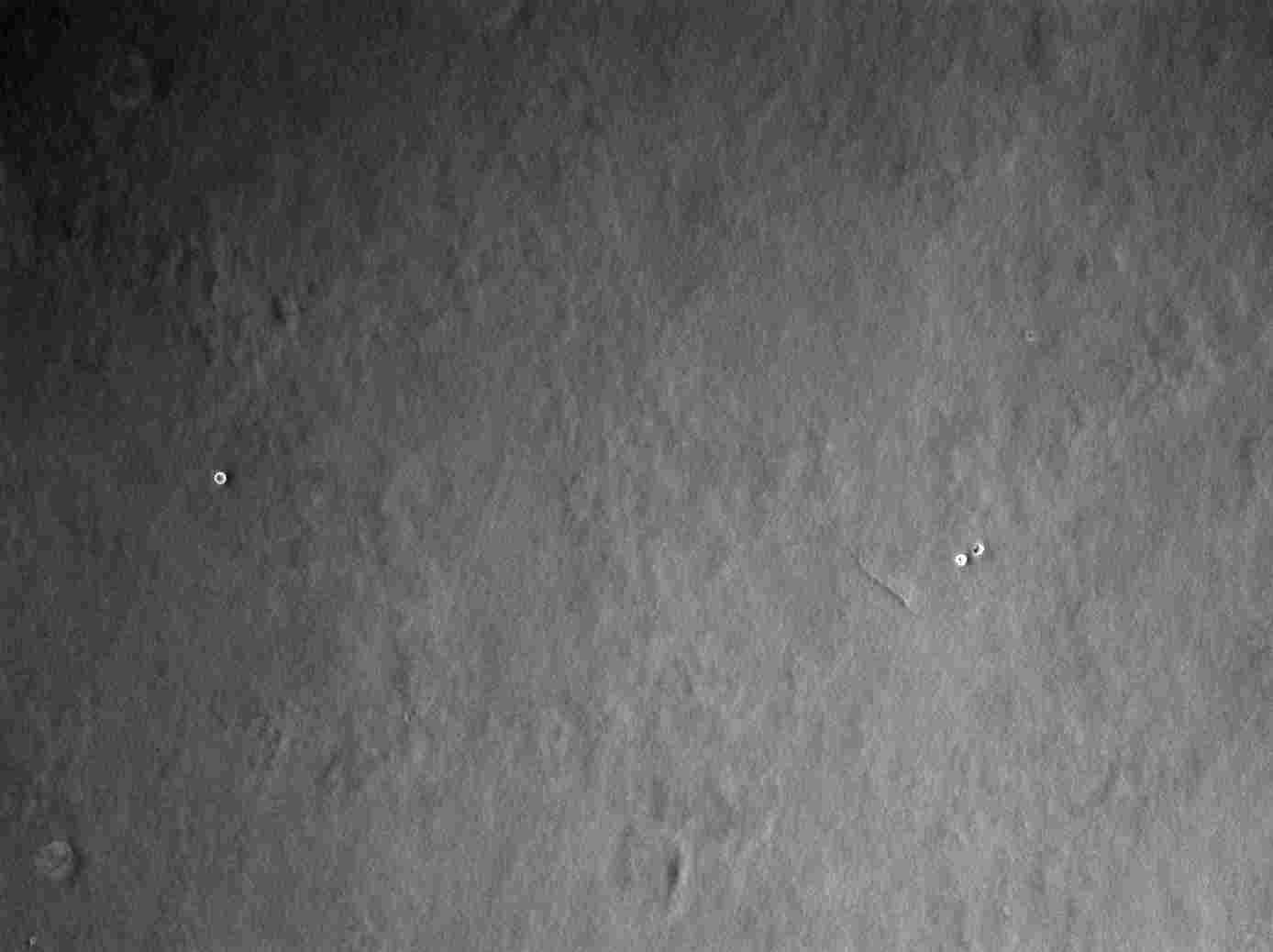

Supplement: S2 File — The raw data are presented in Raw data.zip. (ZIP) [file pone.0339611.s002.zip › Raw data/Figure 4/soft agar/day 1/5+OE-day1 (3).jpg]

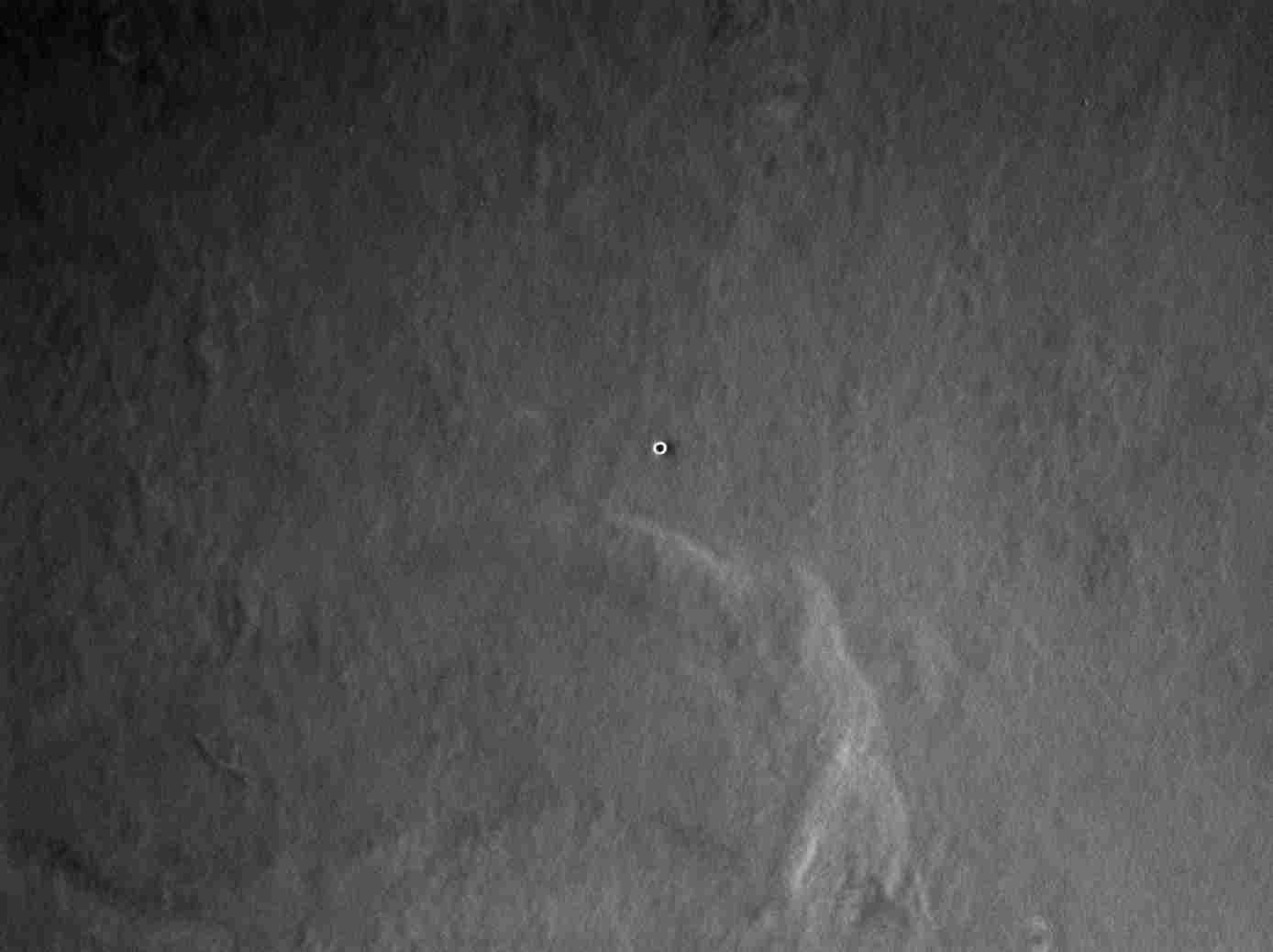

Supplement: S2 File — The raw data are presented in Raw data.zip. (ZIP) [file pone.0339611.s002.zip › Raw data/Figure 4/soft agar/day 1/5+OE-day1 (4).jpg]

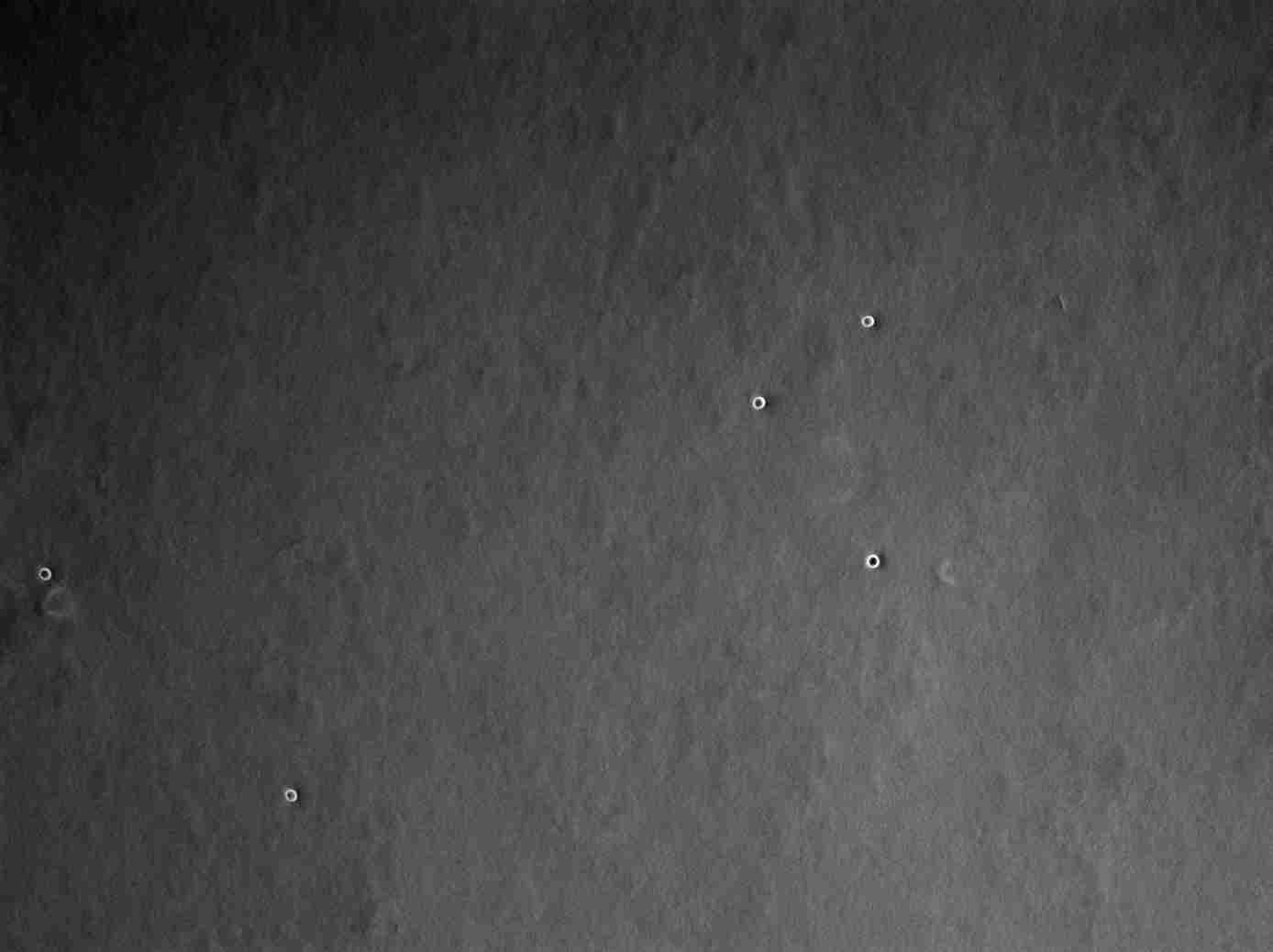

Supplement: S2 File — The raw data are presented in Raw data.zip. (ZIP) [file pone.0339611.s002.zip › Raw data/Figure 4/soft agar/day 1/5+OE-day1 (5).jpg]

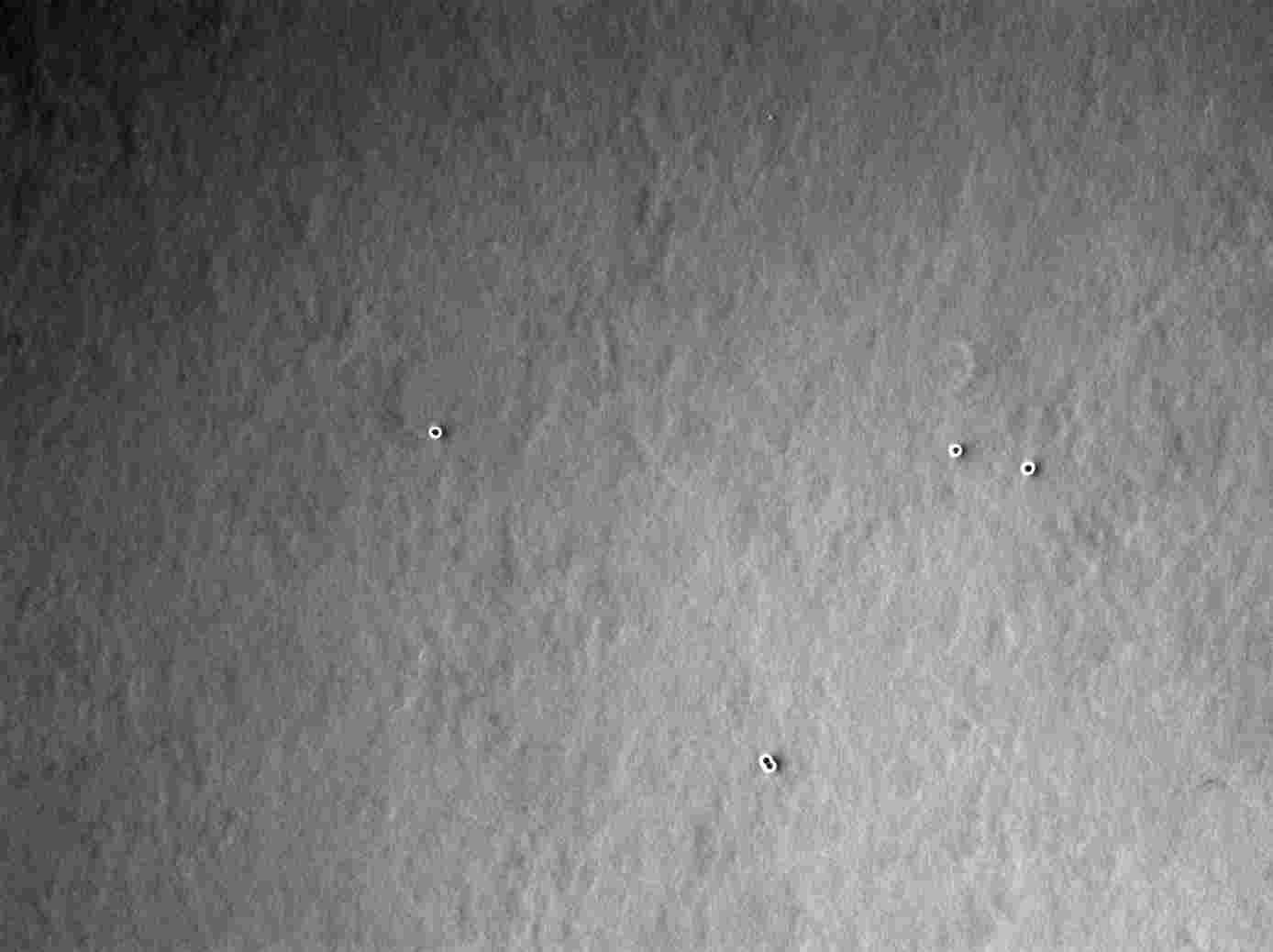

Supplement: S2 File — The raw data are presented in Raw data.zip. (ZIP) [file pone.0339611.s002.zip › Raw data/Figure 4/soft agar/day 1/5+OE-day1 (6).jpg]

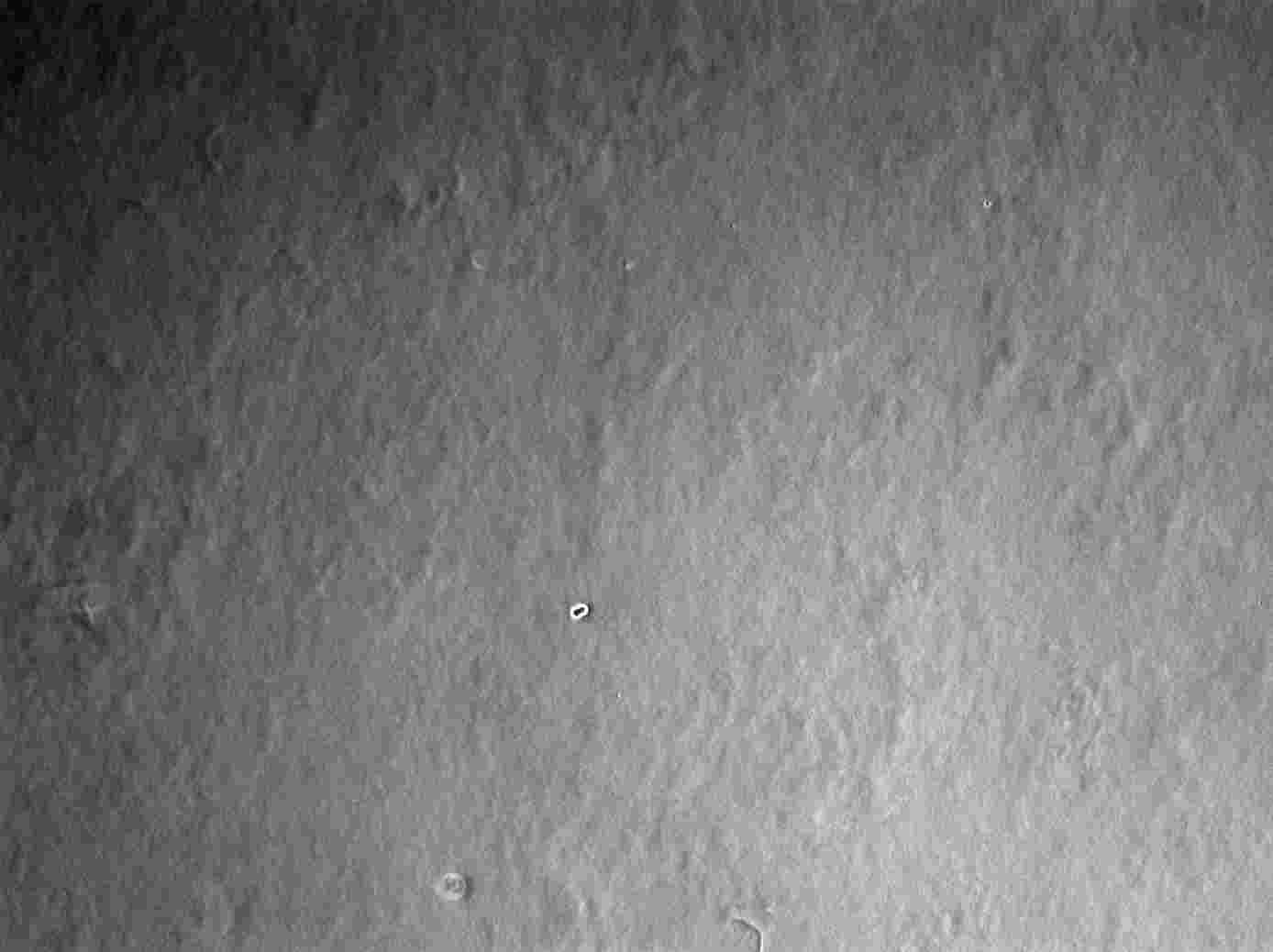

Supplement: S2 File — The raw data are presented in Raw data.zip. (ZIP) [file pone.0339611.s002.zip › Raw data/Figure 4/soft agar/day 1/5+OE-day1 (7).jpg]

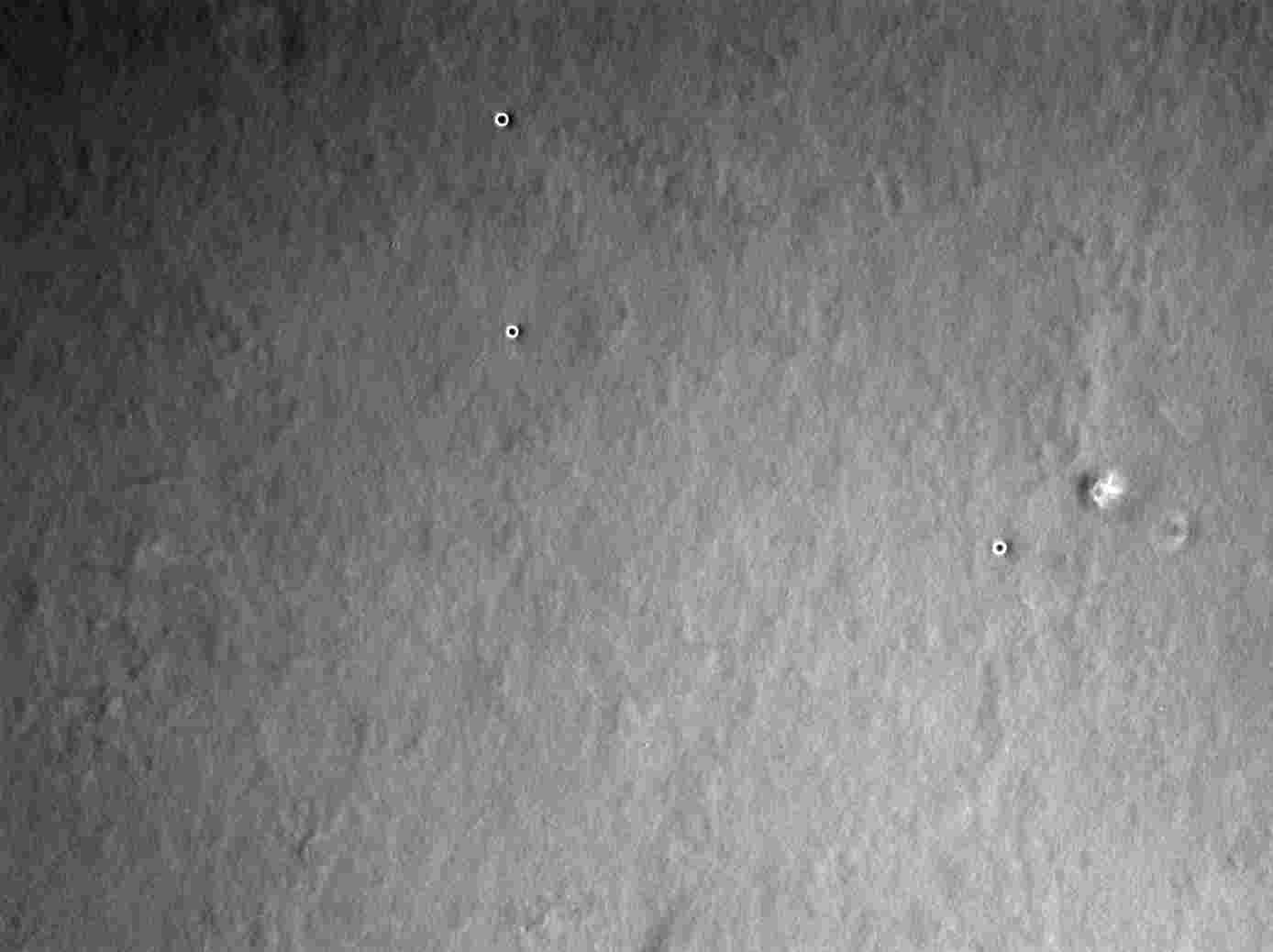

Supplement: S2 File — The raw data are presented in Raw data.zip. (ZIP) [file pone.0339611.s002.zip › Raw data/Figure 4/soft agar/day 1/5+OE-day1 (8).jpg]

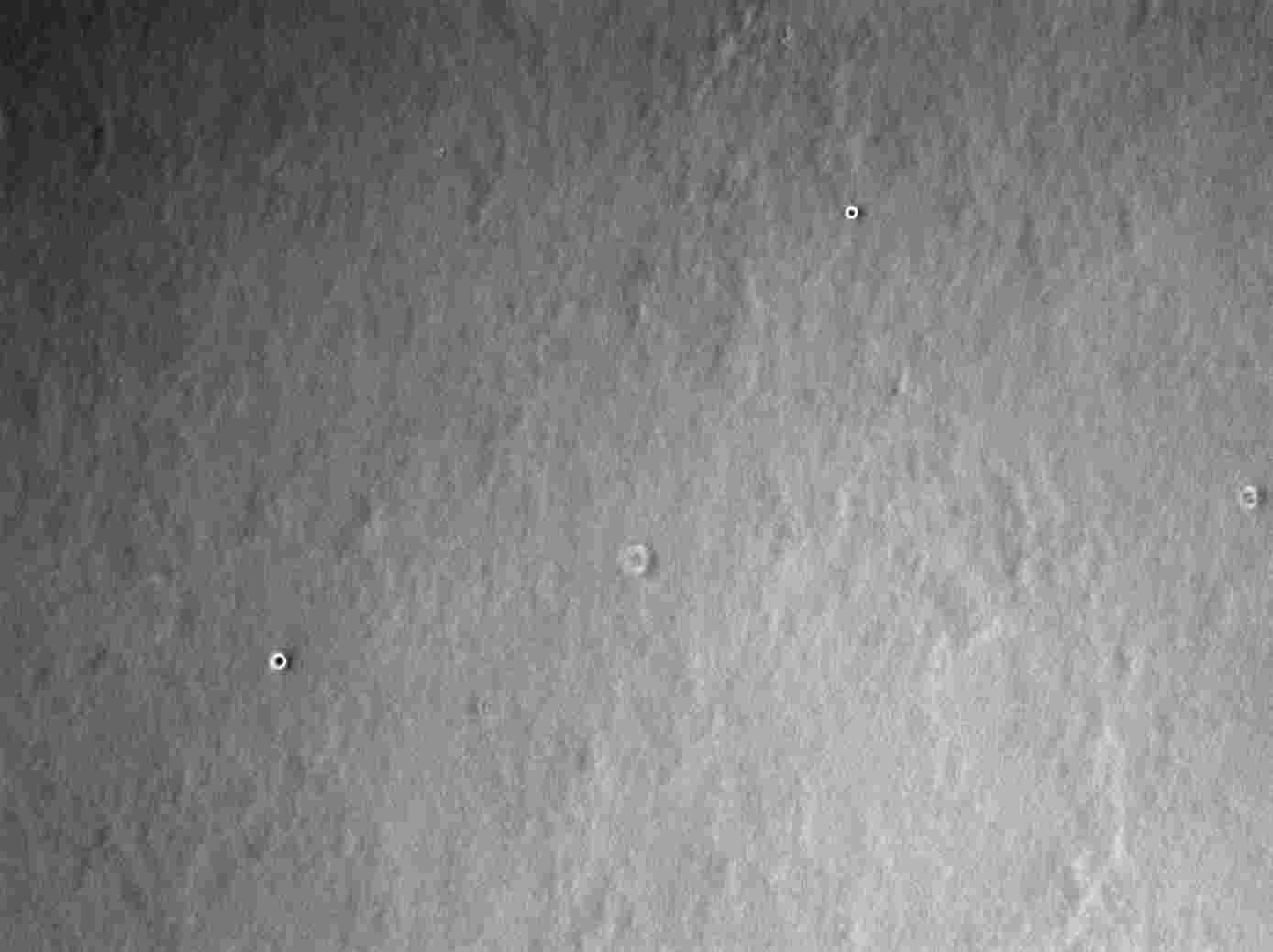

Supplement: S2 File — The raw data are presented in Raw data.zip. (ZIP) [file pone.0339611.s002.zip › Raw data/Figure 4/soft agar/day 1/5+OE-day1 (9).jpg]

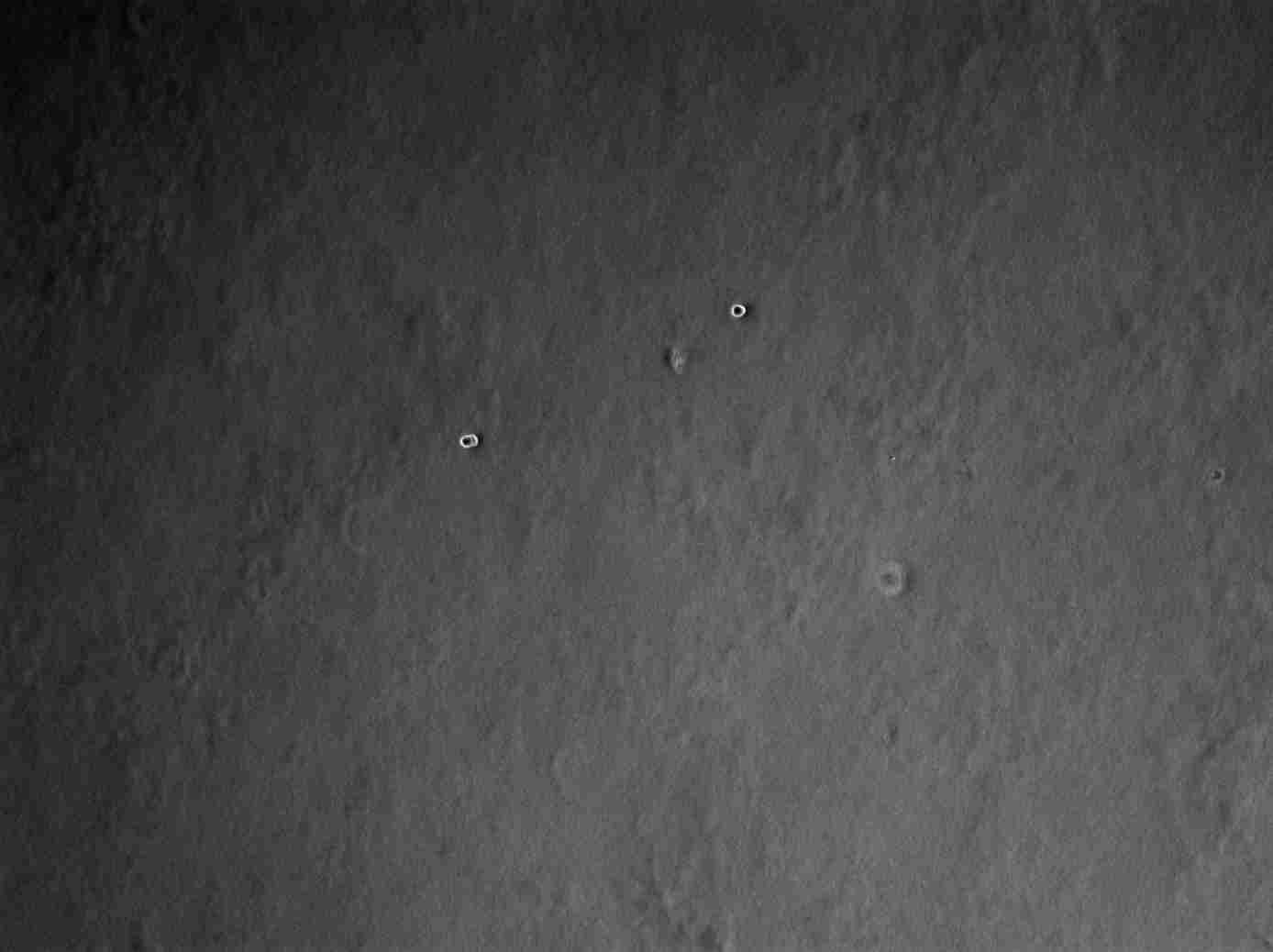

Supplement: S2 File — The raw data are presented in Raw data.zip. (ZIP) [file pone.0339611.s002.zip › Raw data/Figure 4/soft agar/day 1/5+OE-day1.jpg]

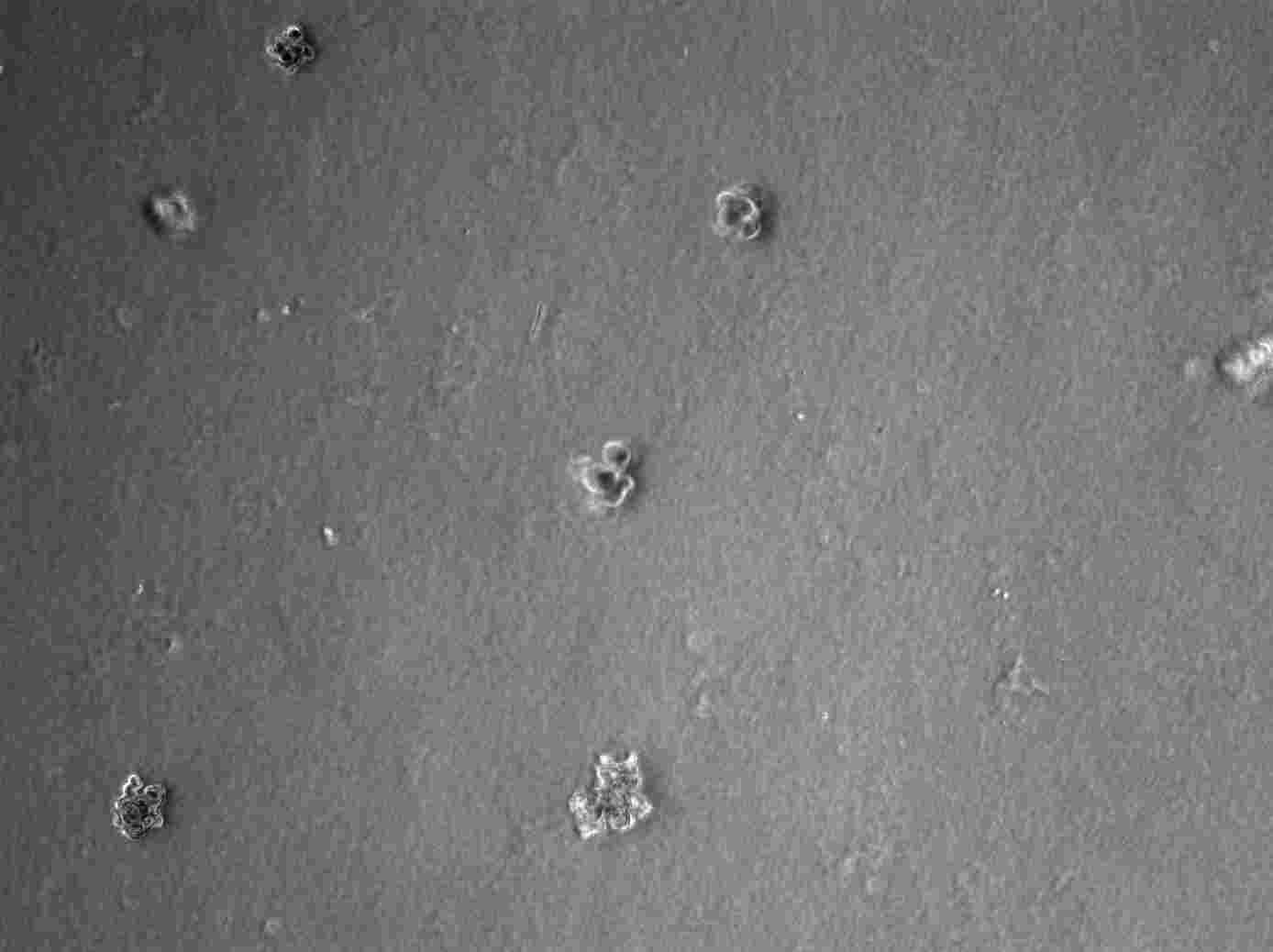

Supplement: S2 File — The raw data are presented in Raw data.zip. (ZIP) [file pone.0339611.s002.zip › Raw data/Figure 4/soft agar/day 14/3+shI-1-day14 (10).jpg]

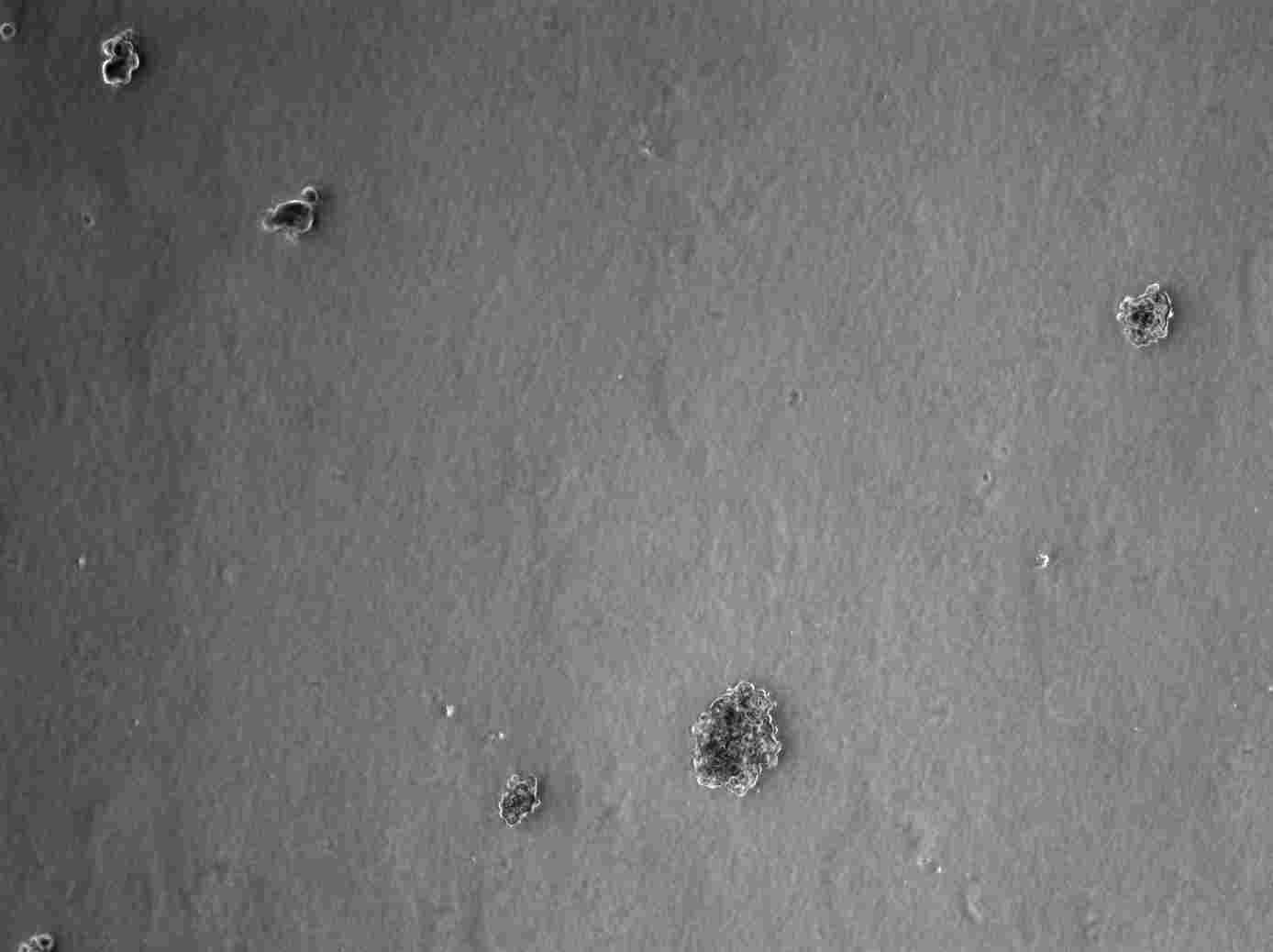

Supplement: S2 File — The raw data are presented in Raw data.zip. (ZIP) [file pone.0339611.s002.zip › Raw data/Figure 4/soft agar/day 14/3+shI-1-day14 (11).jpg]

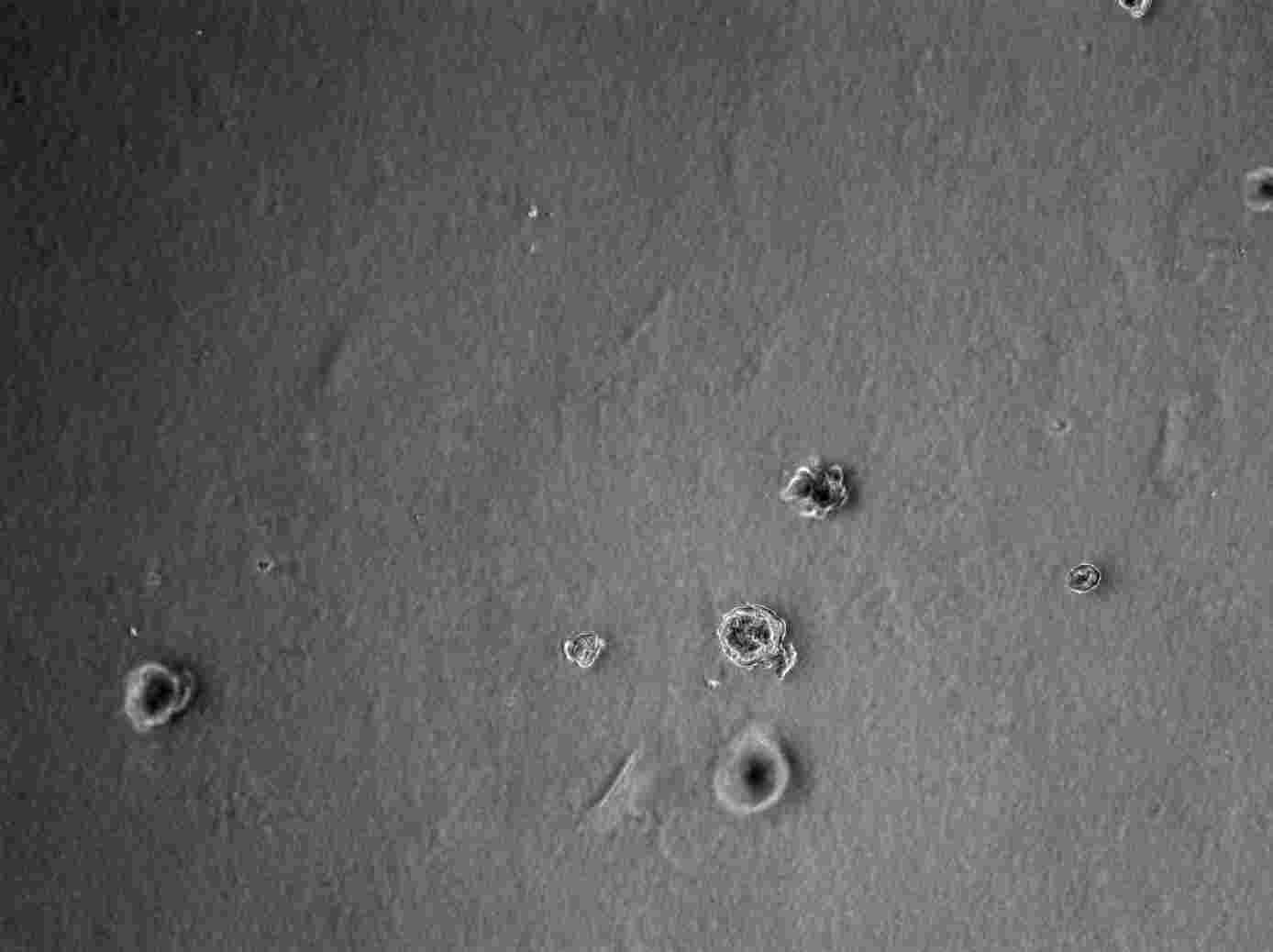

Supplement: S2 File — The raw data are presented in Raw data.zip. (ZIP) [file pone.0339611.s002.zip › Raw data/Figure 4/soft agar/day 14/3+shI-1-day14 (12).jpg]

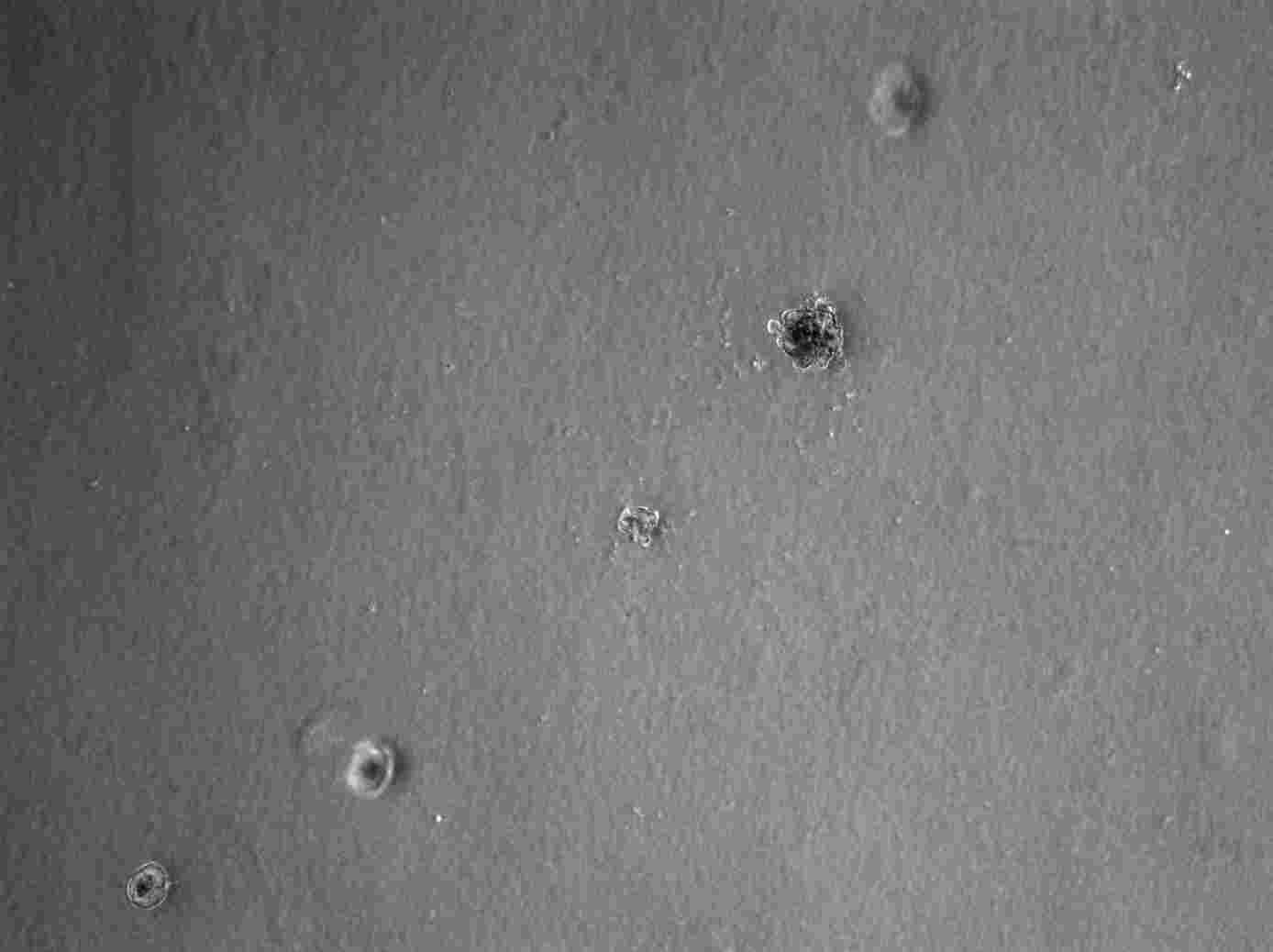

Supplement: S2 File — The raw data are presented in Raw data.zip. (ZIP) [file pone.0339611.s002.zip › Raw data/Figure 4/soft agar/day 14/3+shI-1-day14 (13).jpg]

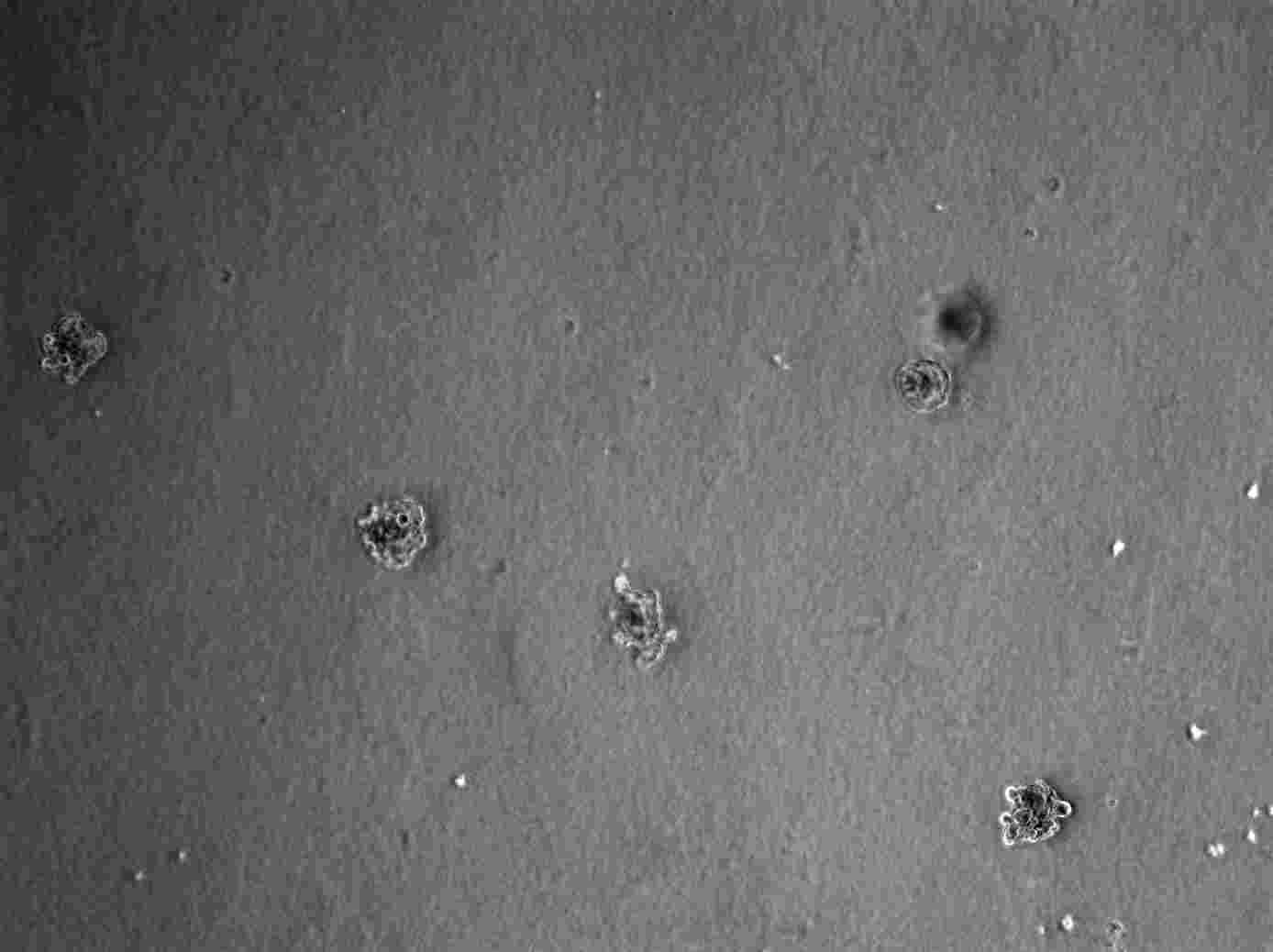

Supplement: S2 File — The raw data are presented in Raw data.zip. (ZIP) [file pone.0339611.s002.zip › Raw data/Figure 4/soft agar/day 14/3+shI-1-day14 (14).jpg]

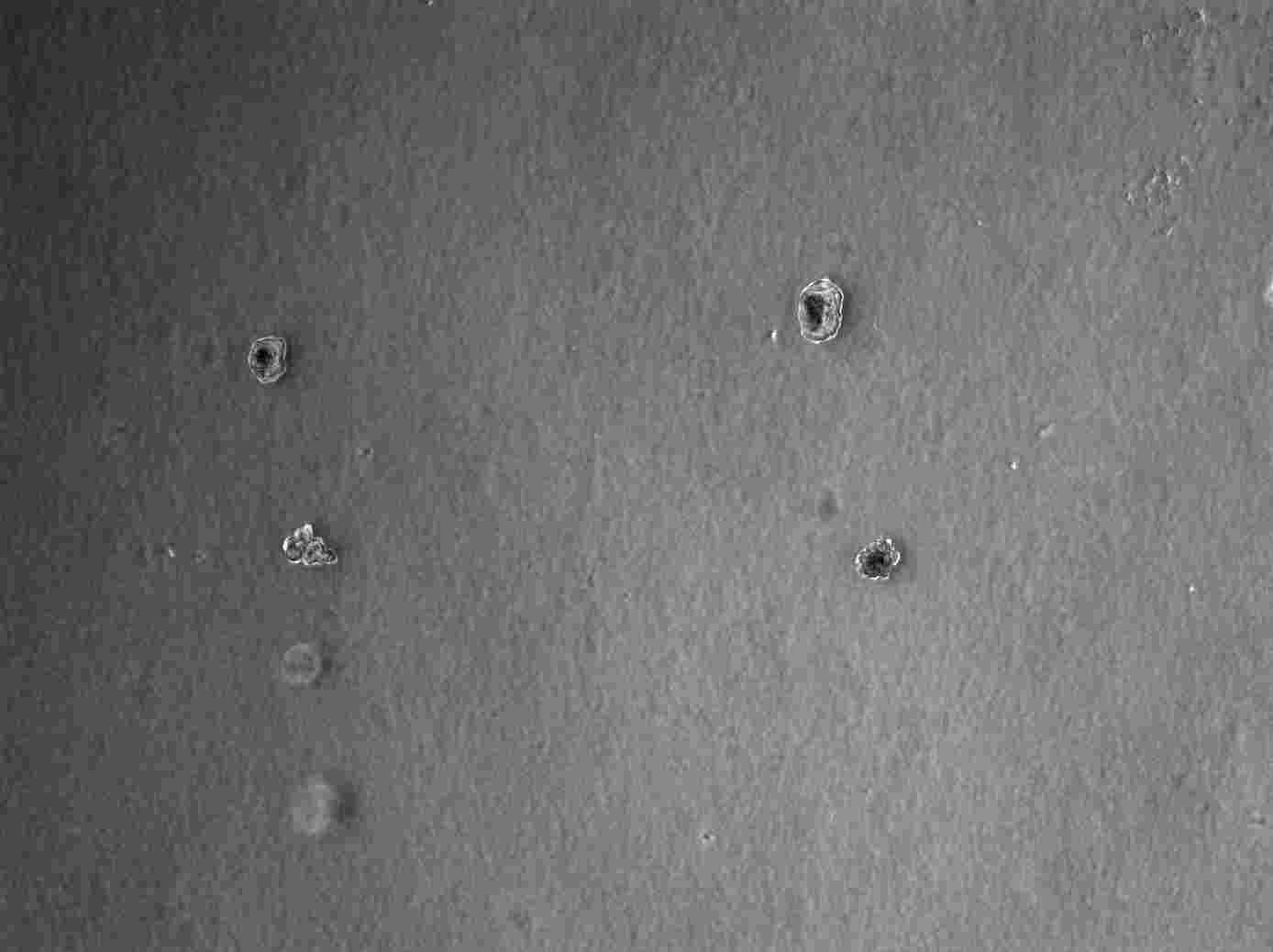

Supplement: S2 File — The raw data are presented in Raw data.zip. (ZIP) [file pone.0339611.s002.zip › Raw data/Figure 4/soft agar/day 14/3+shI-1-day14 (15).jpg]

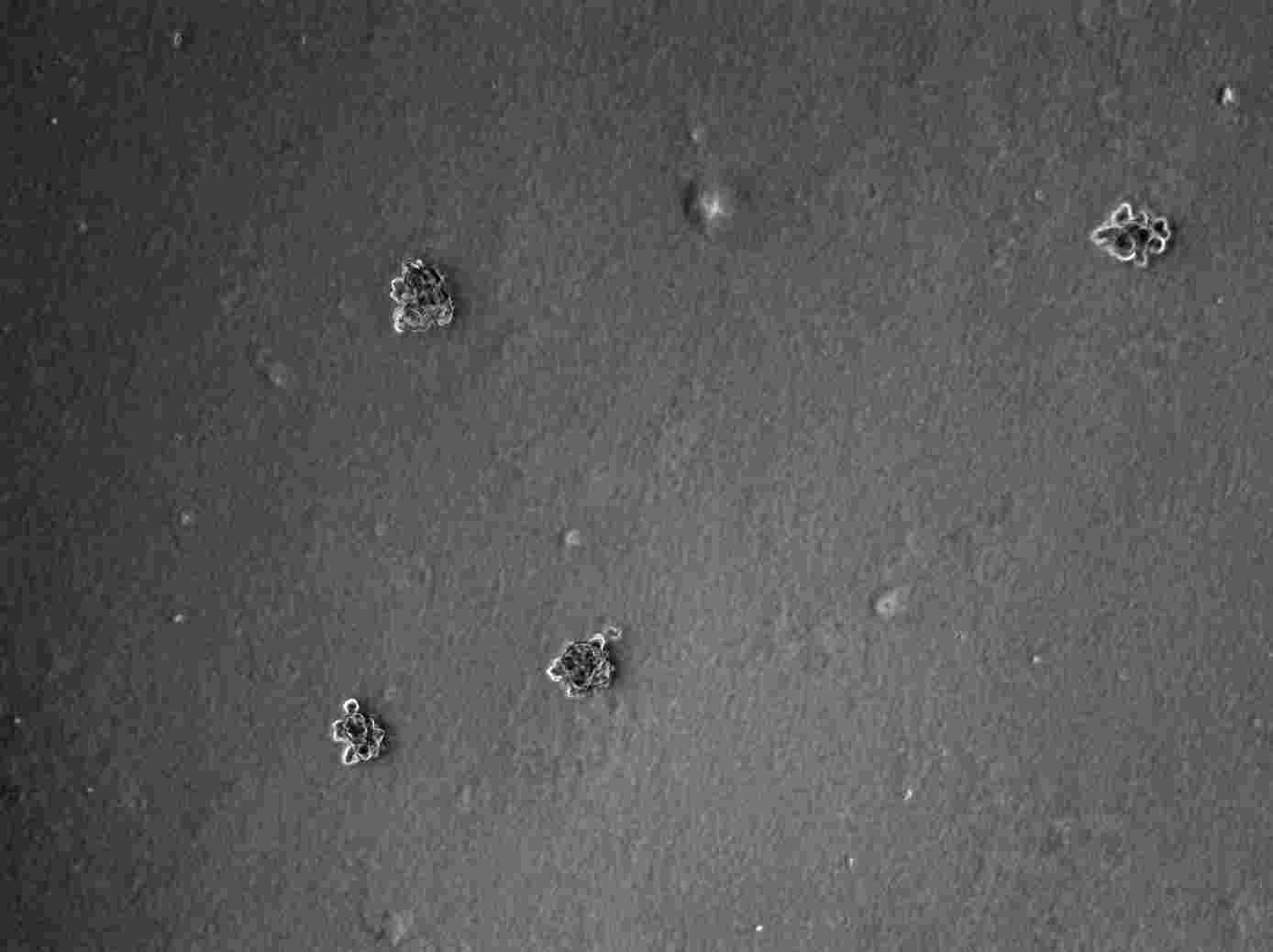

Supplement: S2 File — The raw data are presented in Raw data.zip. (ZIP) [file pone.0339611.s002.zip › Raw data/Figure 4/soft agar/day 14/3+shI-1-day14 (2).jpg]

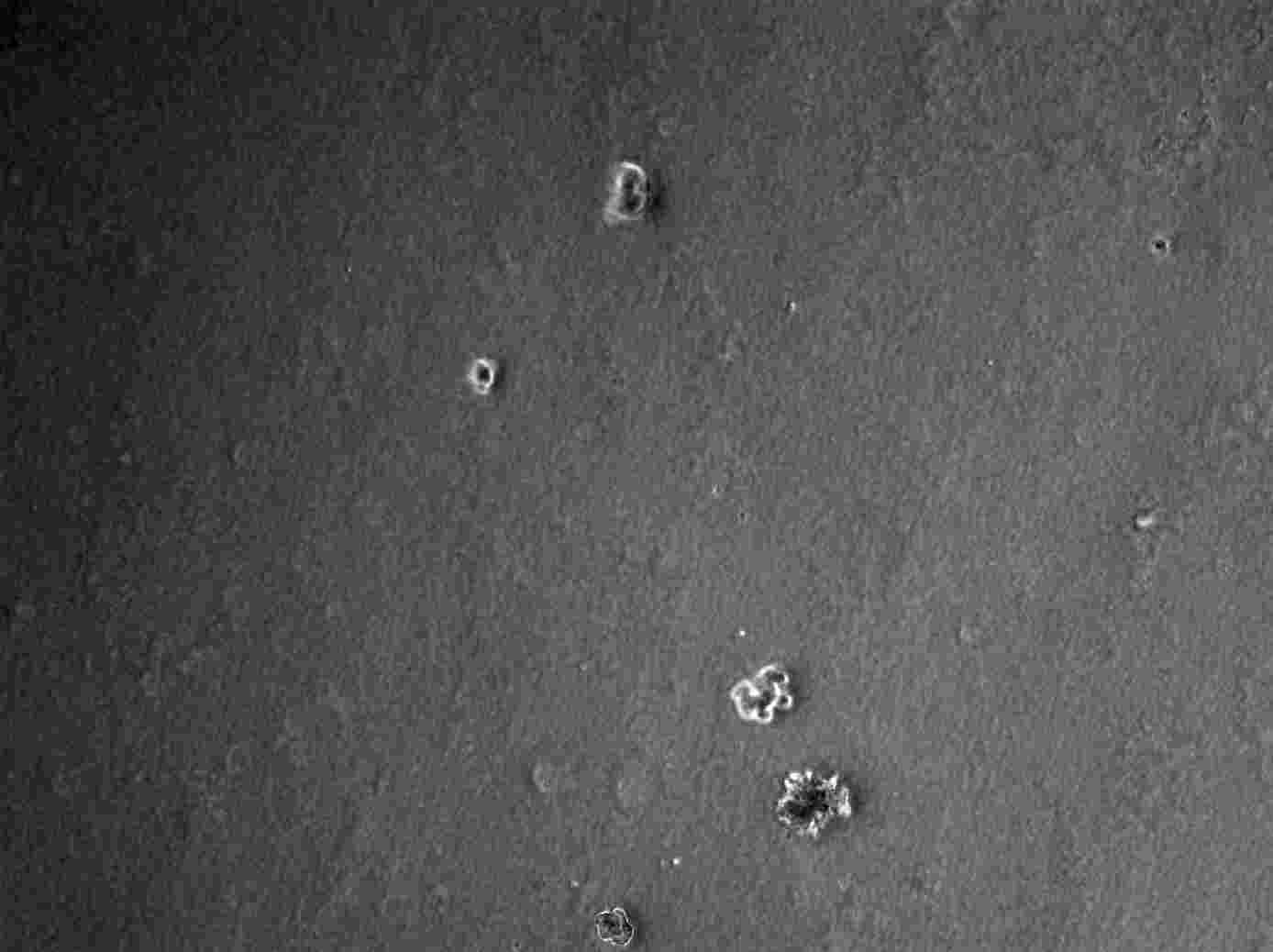

Supplement: S2 File — The raw data are presented in Raw data.zip. (ZIP) [file pone.0339611.s002.zip › Raw data/Figure 4/soft agar/day 14/3+shI-1-day14 (3).jpg]

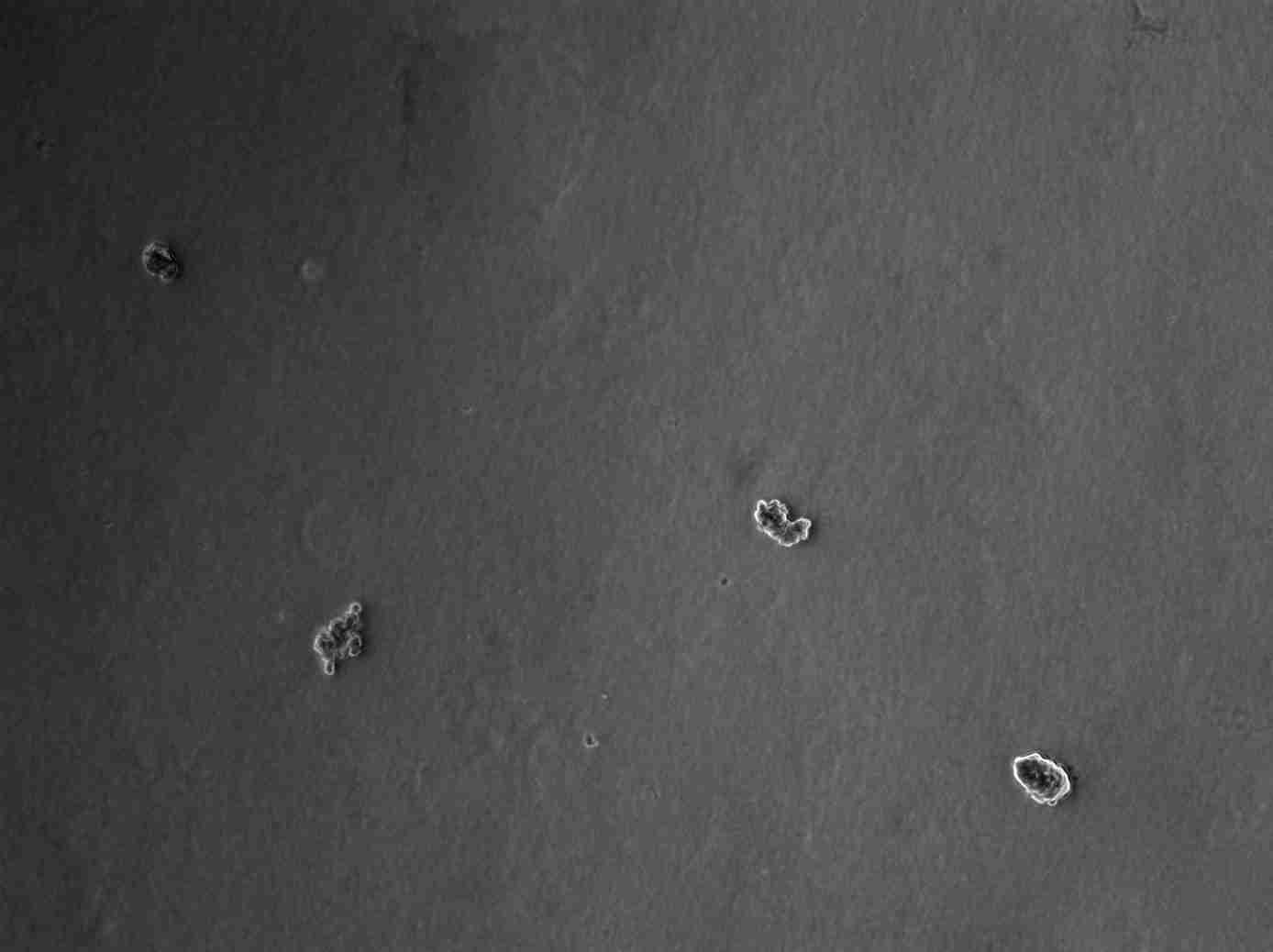

Supplement: S2 File — The raw data are presented in Raw data.zip. (ZIP) [file pone.0339611.s002.zip › Raw data/Figure 4/soft agar/day 14/3+shI-1-day14 (4).jpg]

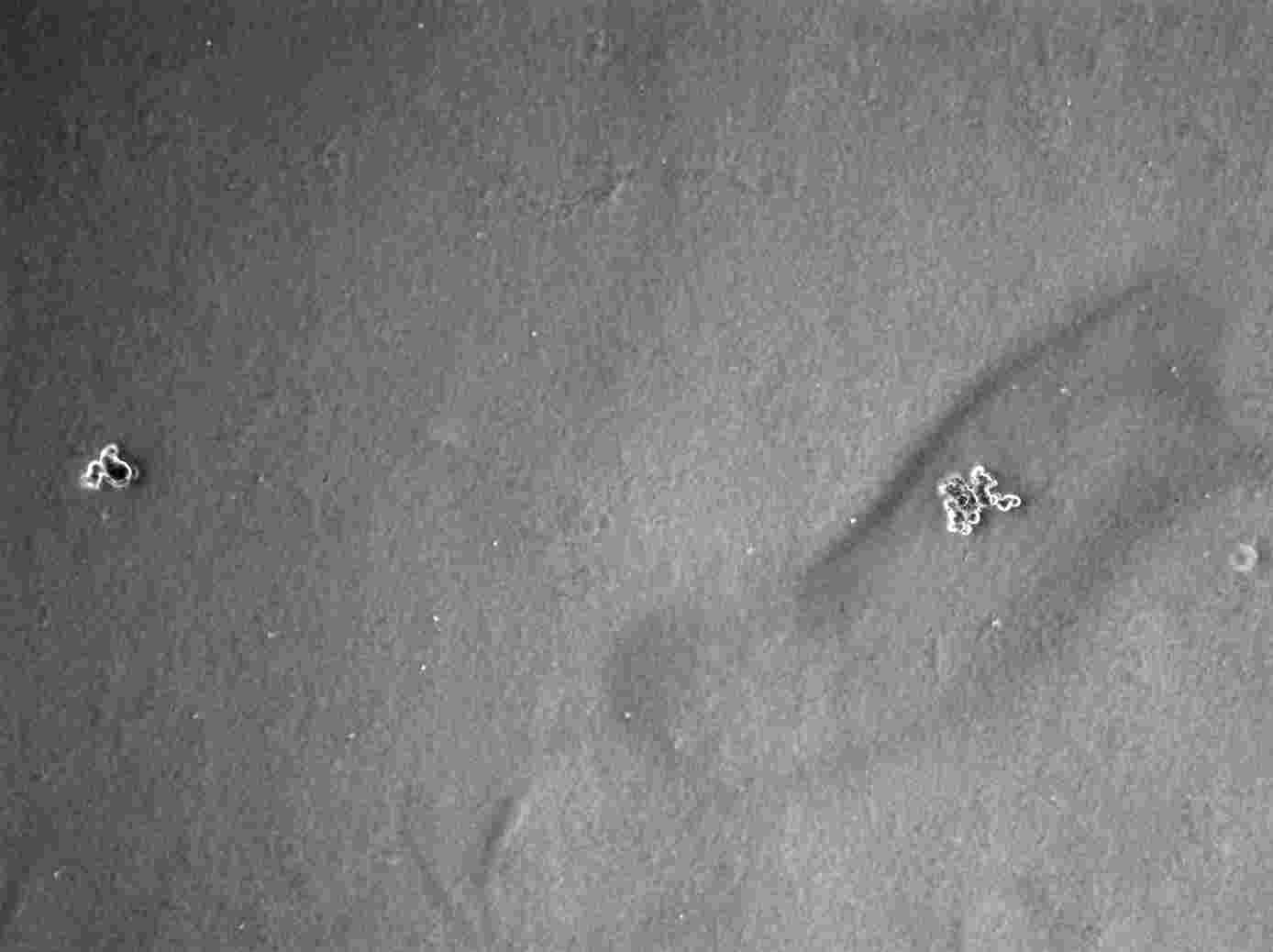

Supplement: S2 File — The raw data are presented in Raw data.zip. (ZIP) [file pone.0339611.s002.zip › Raw data/Figure 4/soft agar/day 14/3+shI-1-day14 (5).jpg]

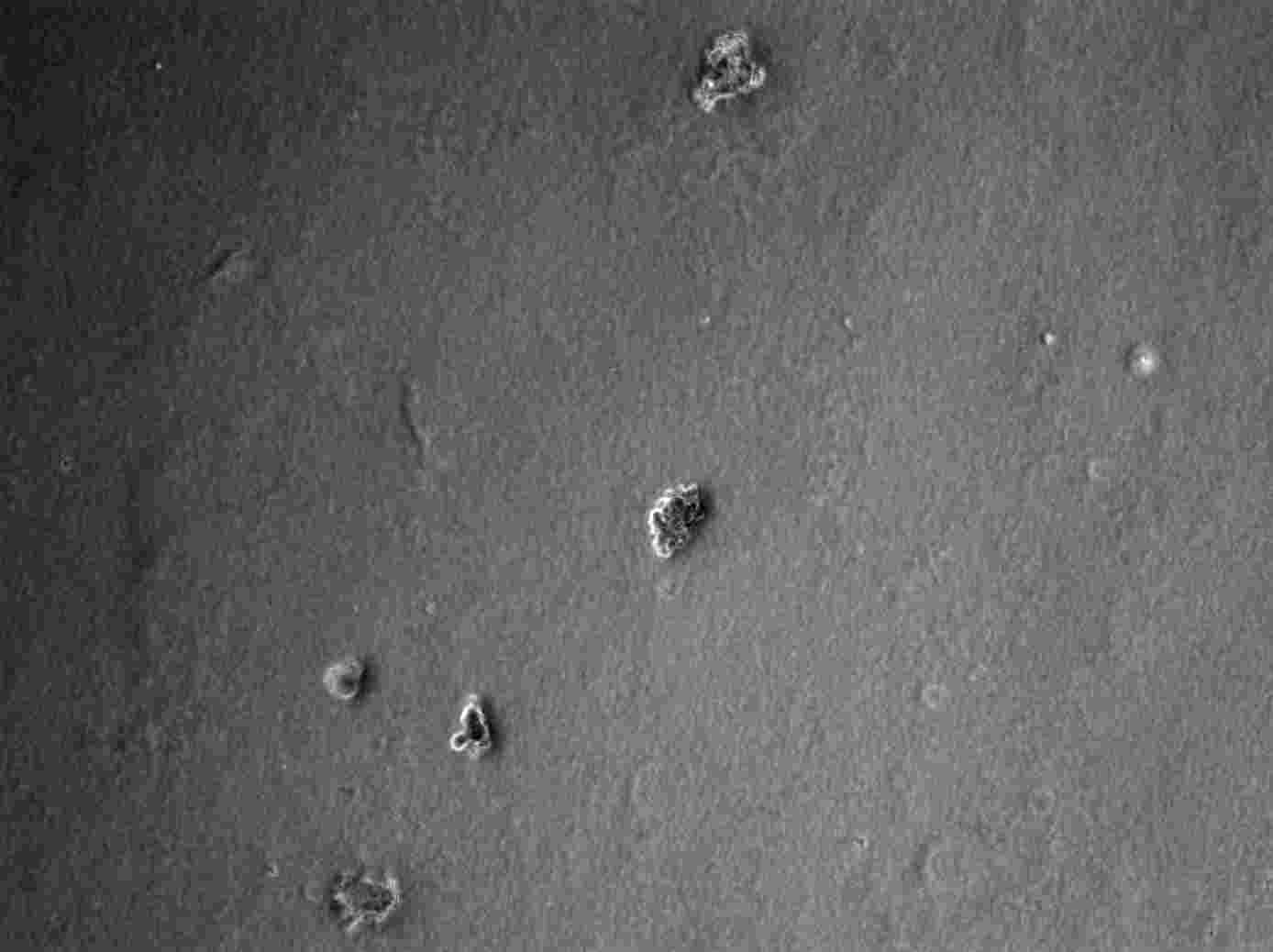

Supplement: S2 File — The raw data are presented in Raw data.zip. (ZIP) [file pone.0339611.s002.zip › Raw data/Figure 4/soft agar/day 14/3+shI-1-day14 (6).jpg]

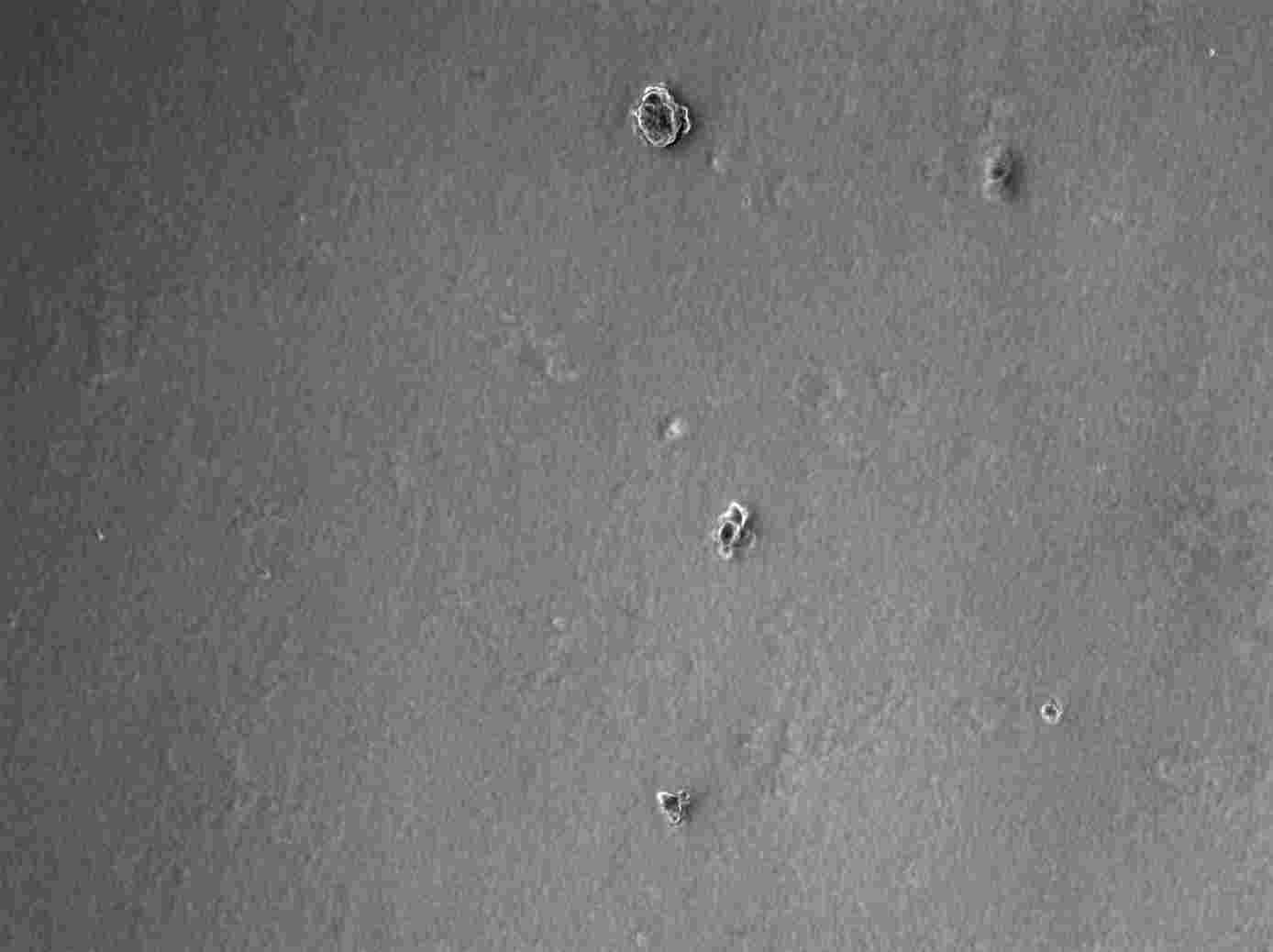

Supplement: S2 File — The raw data are presented in Raw data.zip. (ZIP) [file pone.0339611.s002.zip › Raw data/Figure 4/soft agar/day 14/3+shI-1-day14 (7).jpg]

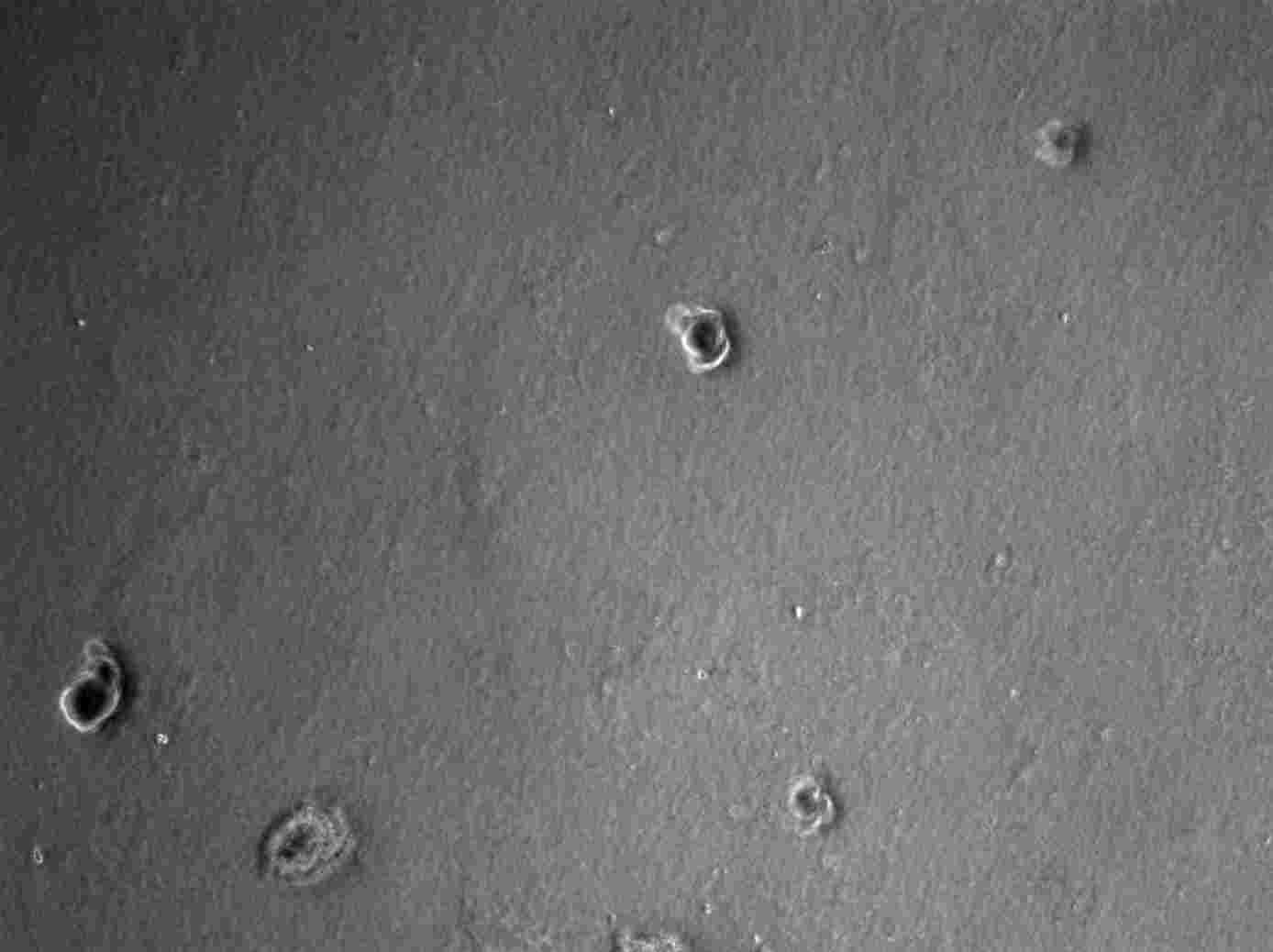

Supplement: S2 File — The raw data are presented in Raw data.zip. (ZIP) [file pone.0339611.s002.zip › Raw data/Figure 4/soft agar/day 14/3+shI-1-day14 (8).jpg]

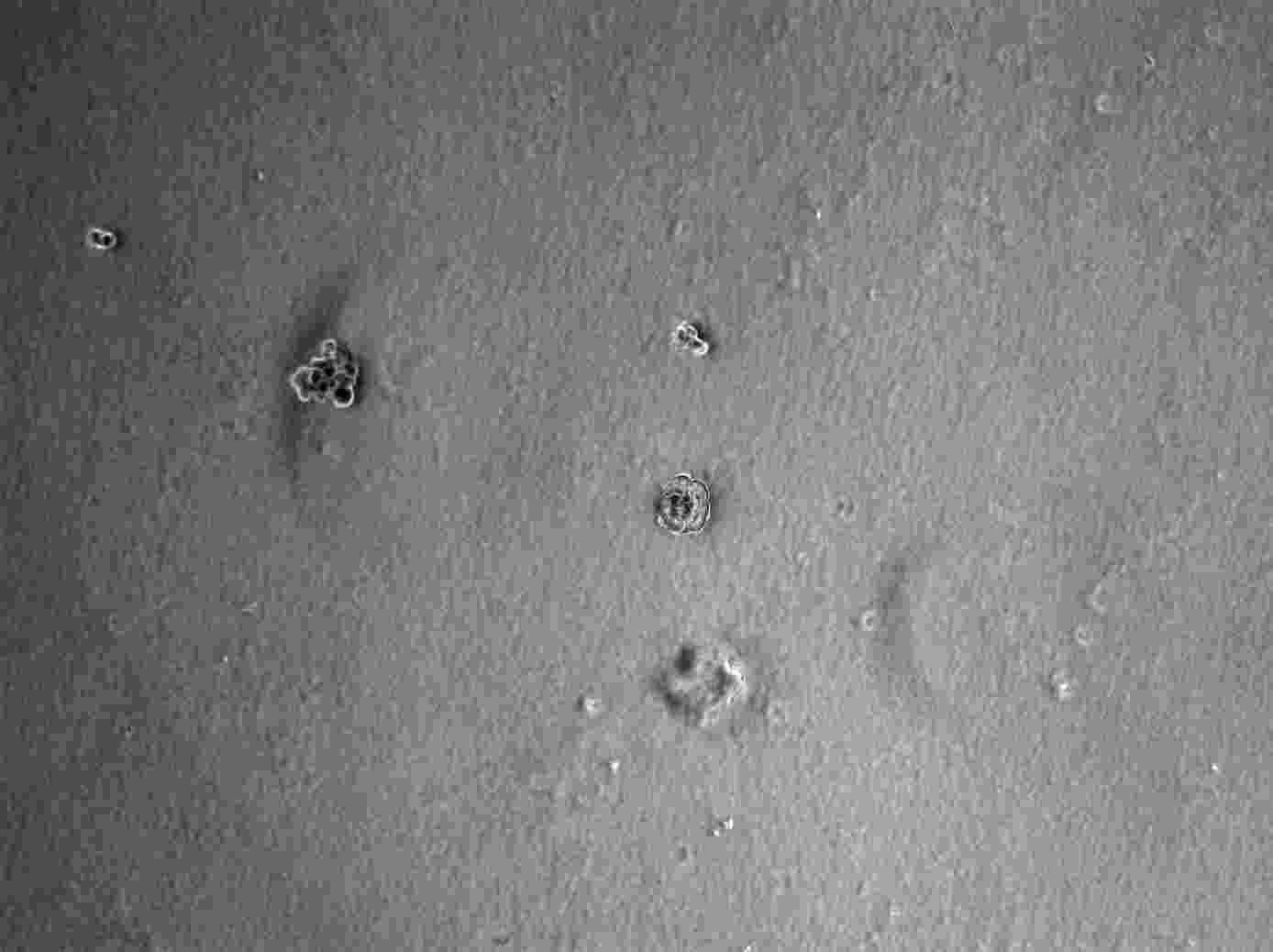

Supplement: S2 File — The raw data are presented in Raw data.zip. (ZIP) [file pone.0339611.s002.zip › Raw data/Figure 4/soft agar/day 14/3+shI-1-day14 (9).jpg]

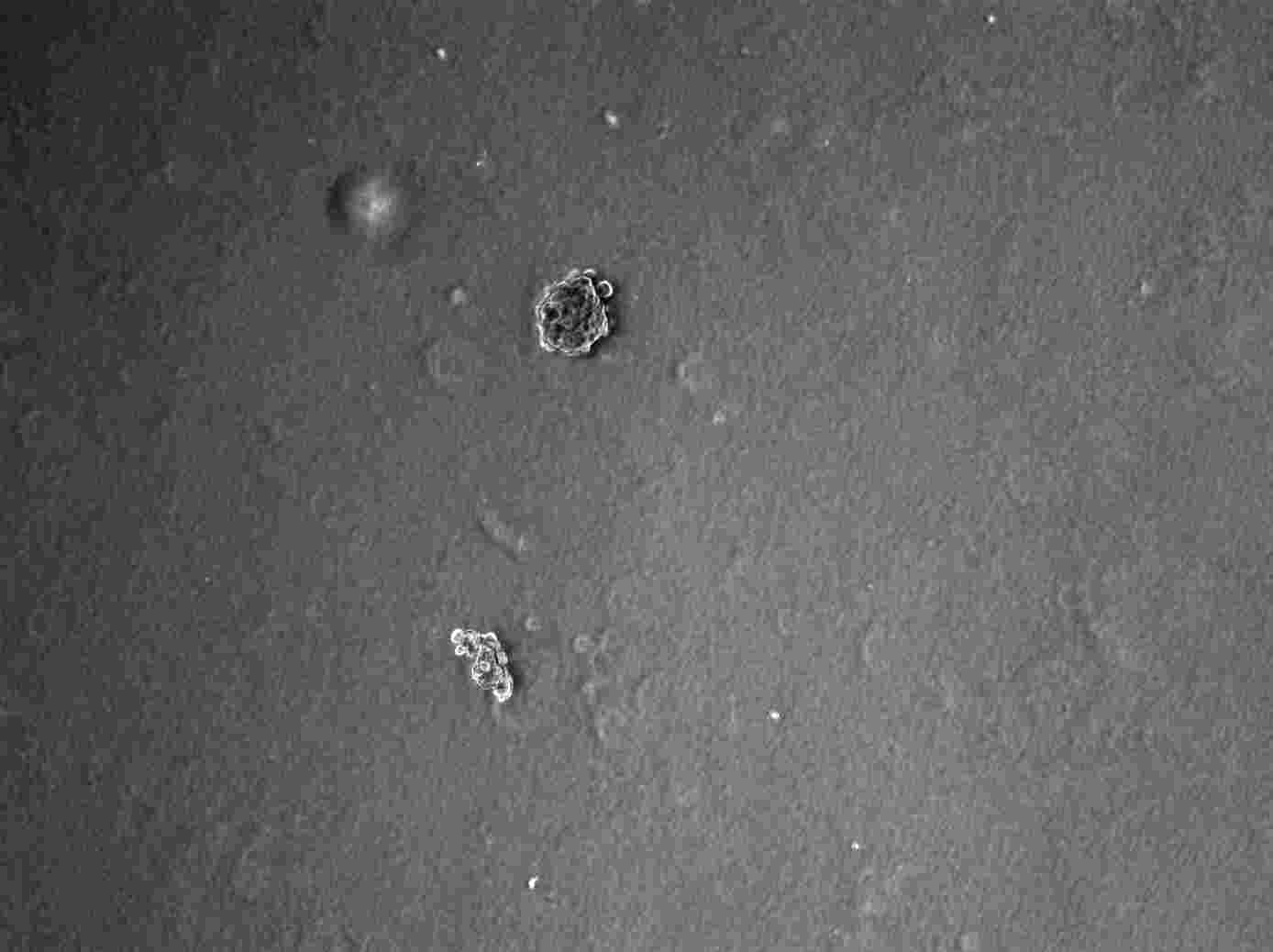

Supplement: S2 File — The raw data are presented in Raw data.zip. (ZIP) [file pone.0339611.s002.zip › Raw data/Figure 4/soft agar/day 14/3+shI-1-day14.jpg]

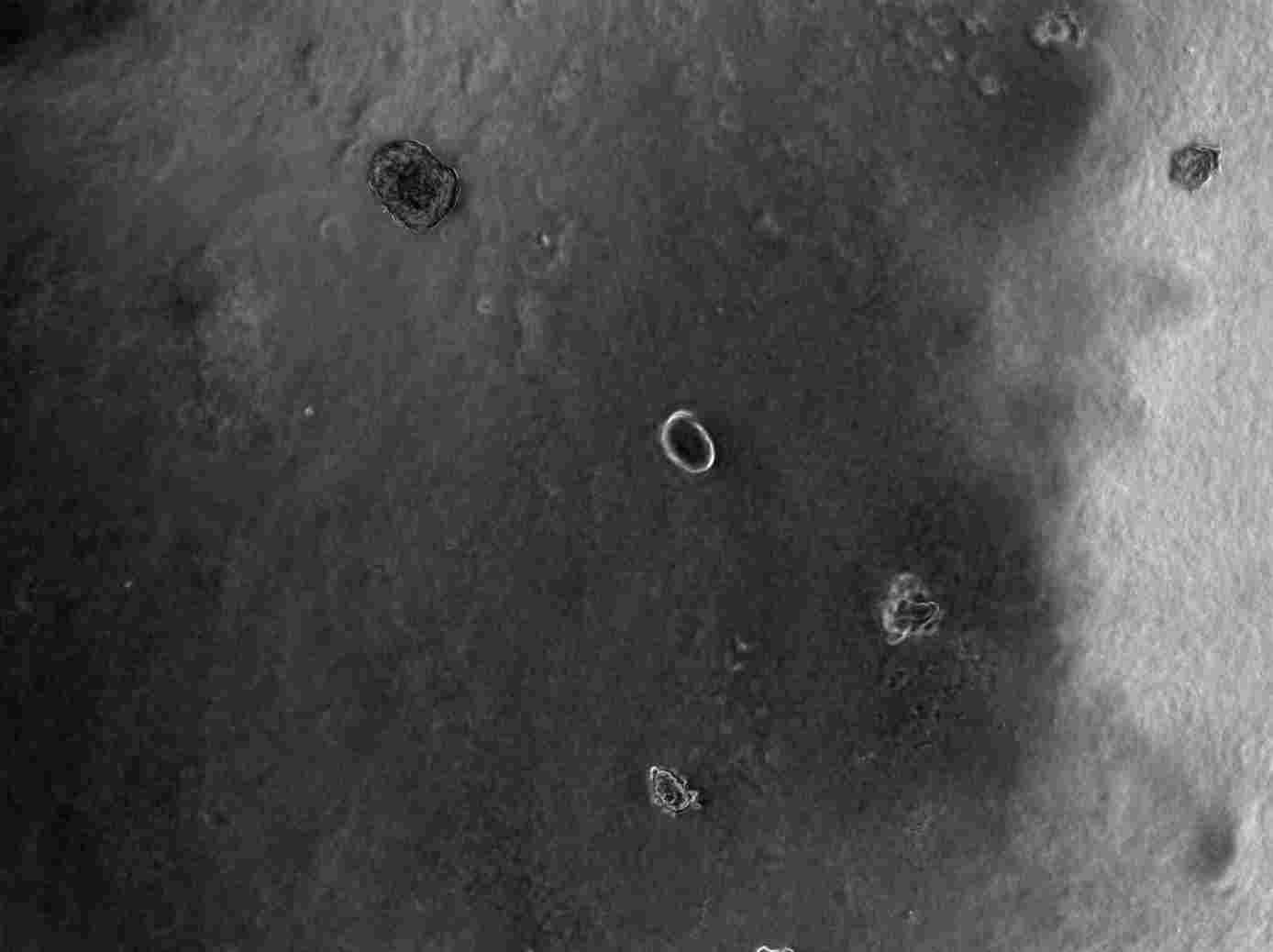

Supplement: S2 File — The raw data are presented in Raw data.zip. (ZIP) [file pone.0339611.s002.zip › Raw data/Figure 4/soft agar/day 14/3+shI-2-day14 (10).jpg]

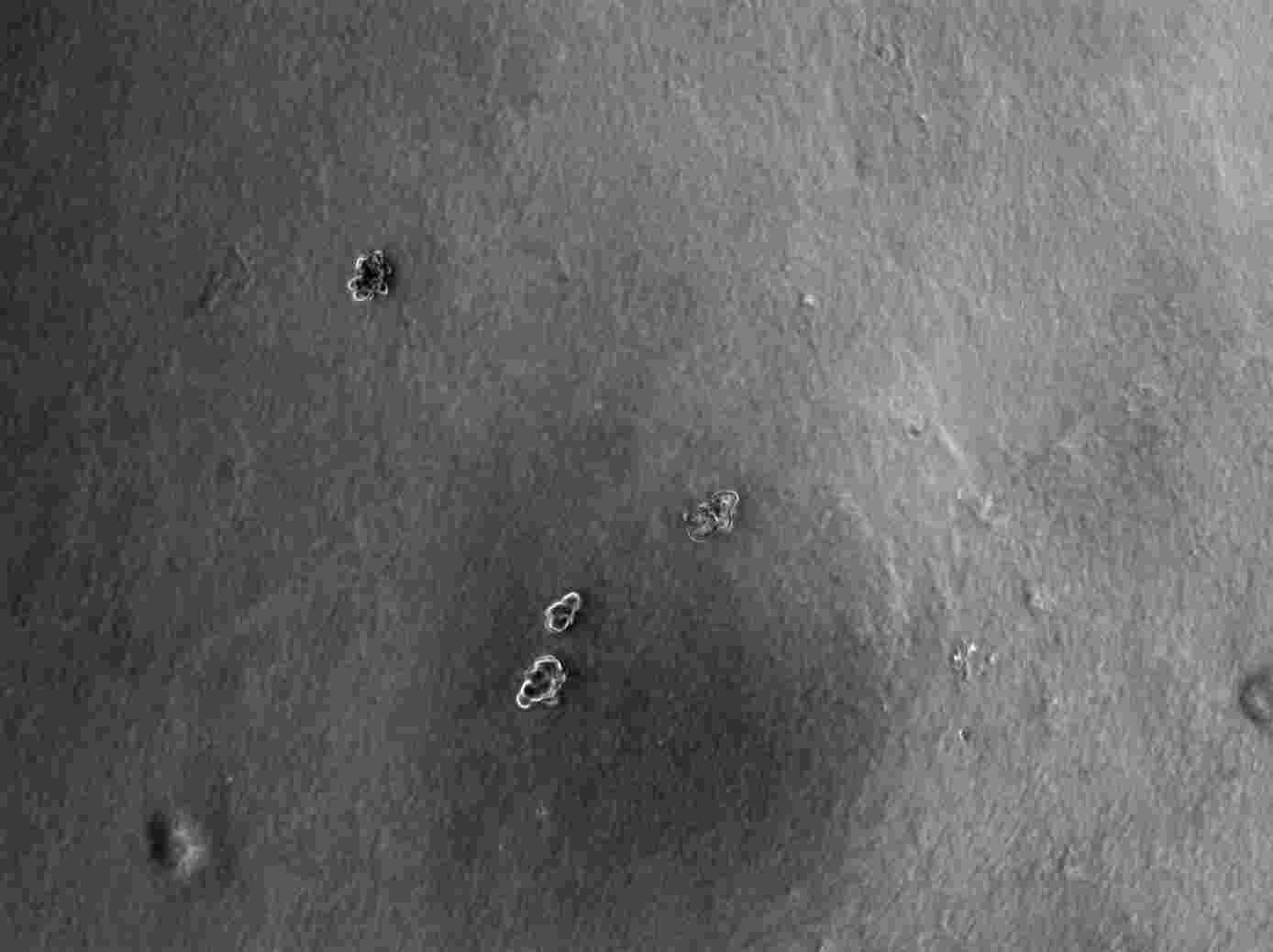

Supplement: S2 File — The raw data are presented in Raw data.zip. (ZIP) [file pone.0339611.s002.zip › Raw data/Figure 4/soft agar/day 14/3+shI-2-day14 (11).jpg]

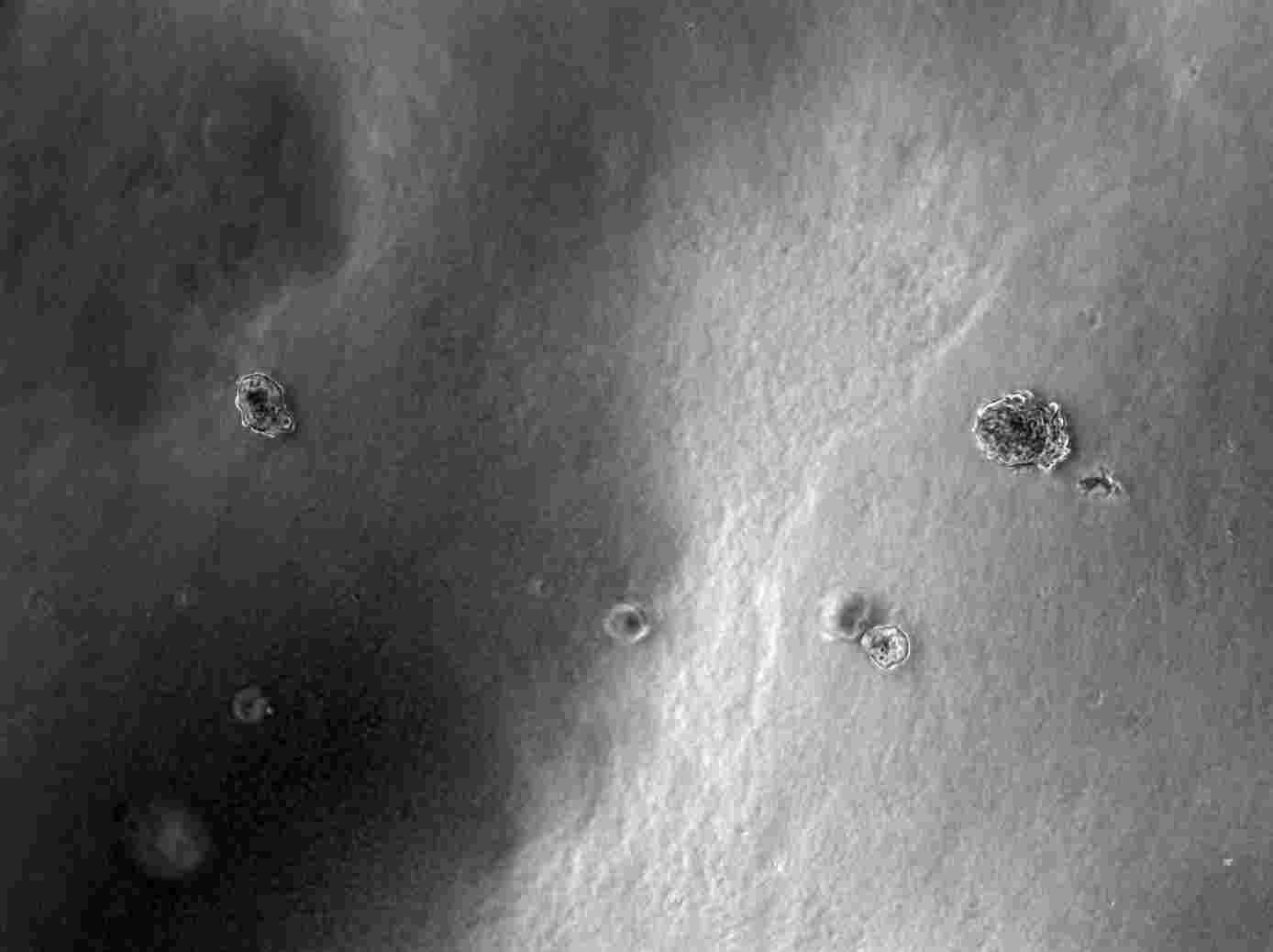

Supplement: S2 File — The raw data are presented in Raw data.zip. (ZIP) [file pone.0339611.s002.zip › Raw data/Figure 4/soft agar/day 14/3+shI-2-day14 (12).jpg]

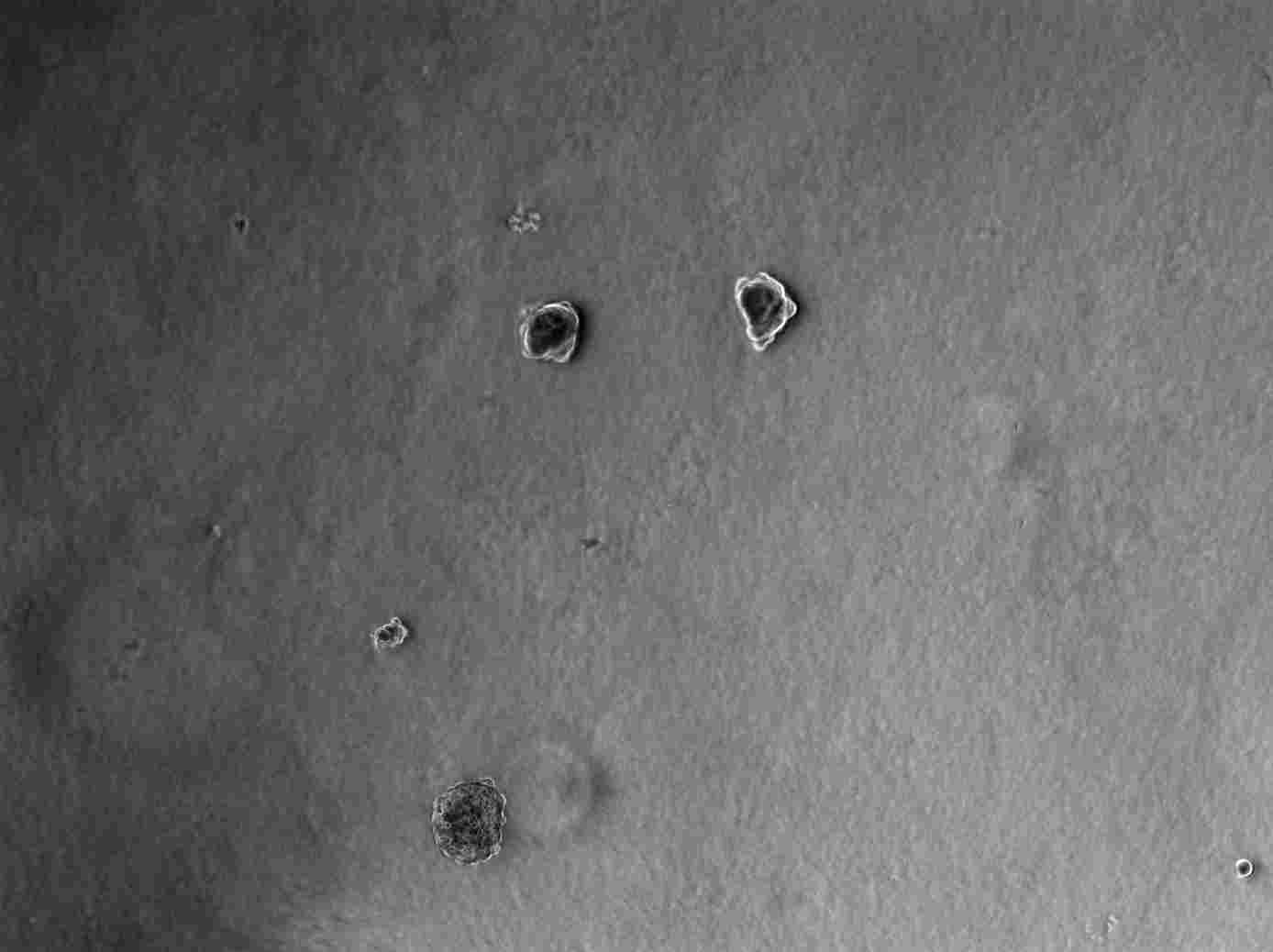

Supplement: S2 File — The raw data are presented in Raw data.zip. (ZIP) [file pone.0339611.s002.zip › Raw data/Figure 4/soft agar/day 14/3+shI-2-day14 (13).jpg]

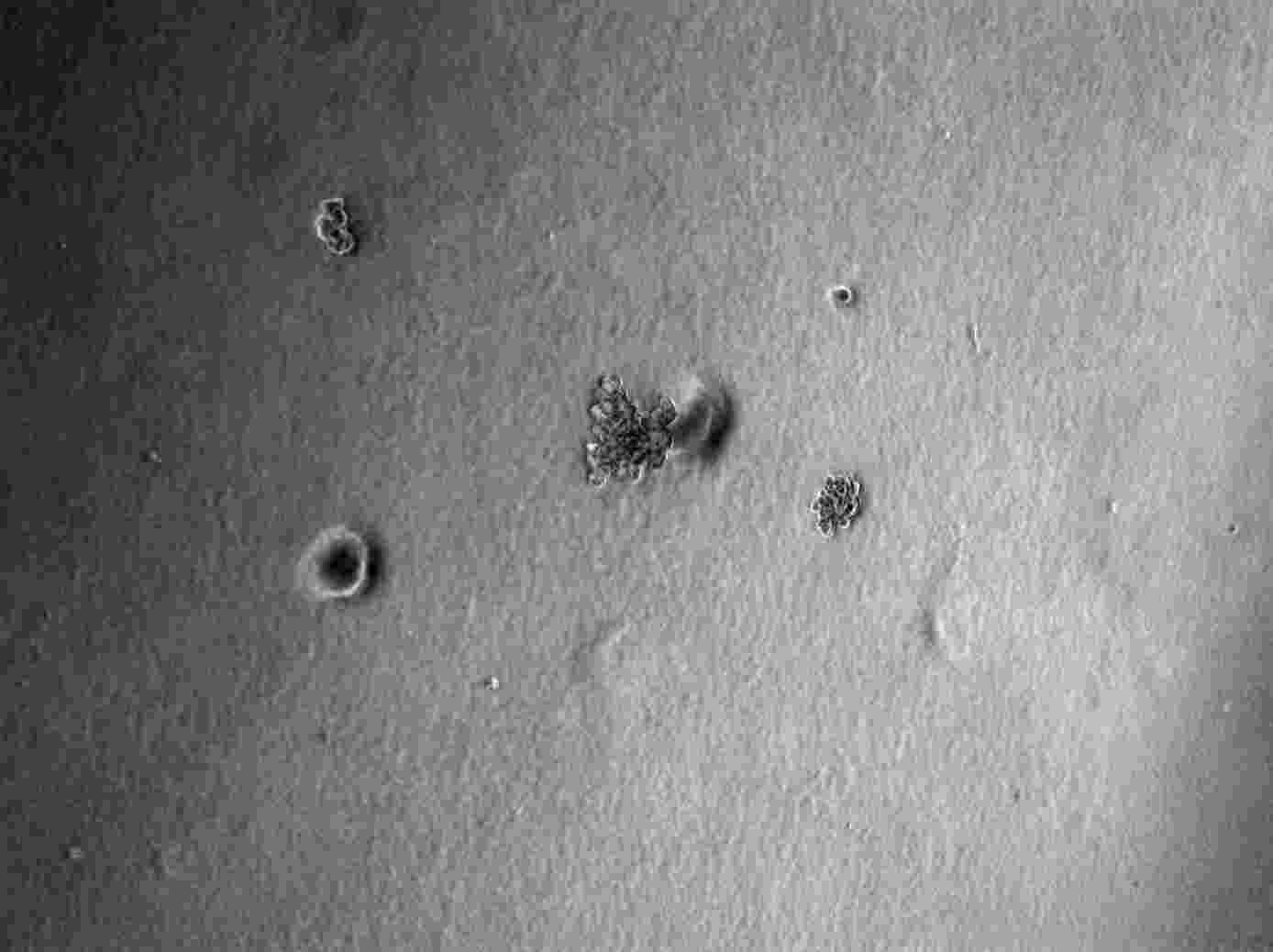

Supplement: S2 File — The raw data are presented in Raw data.zip. (ZIP) [file pone.0339611.s002.zip › Raw data/Figure 4/soft agar/day 14/3+shI-2-day14 (14).jpg]

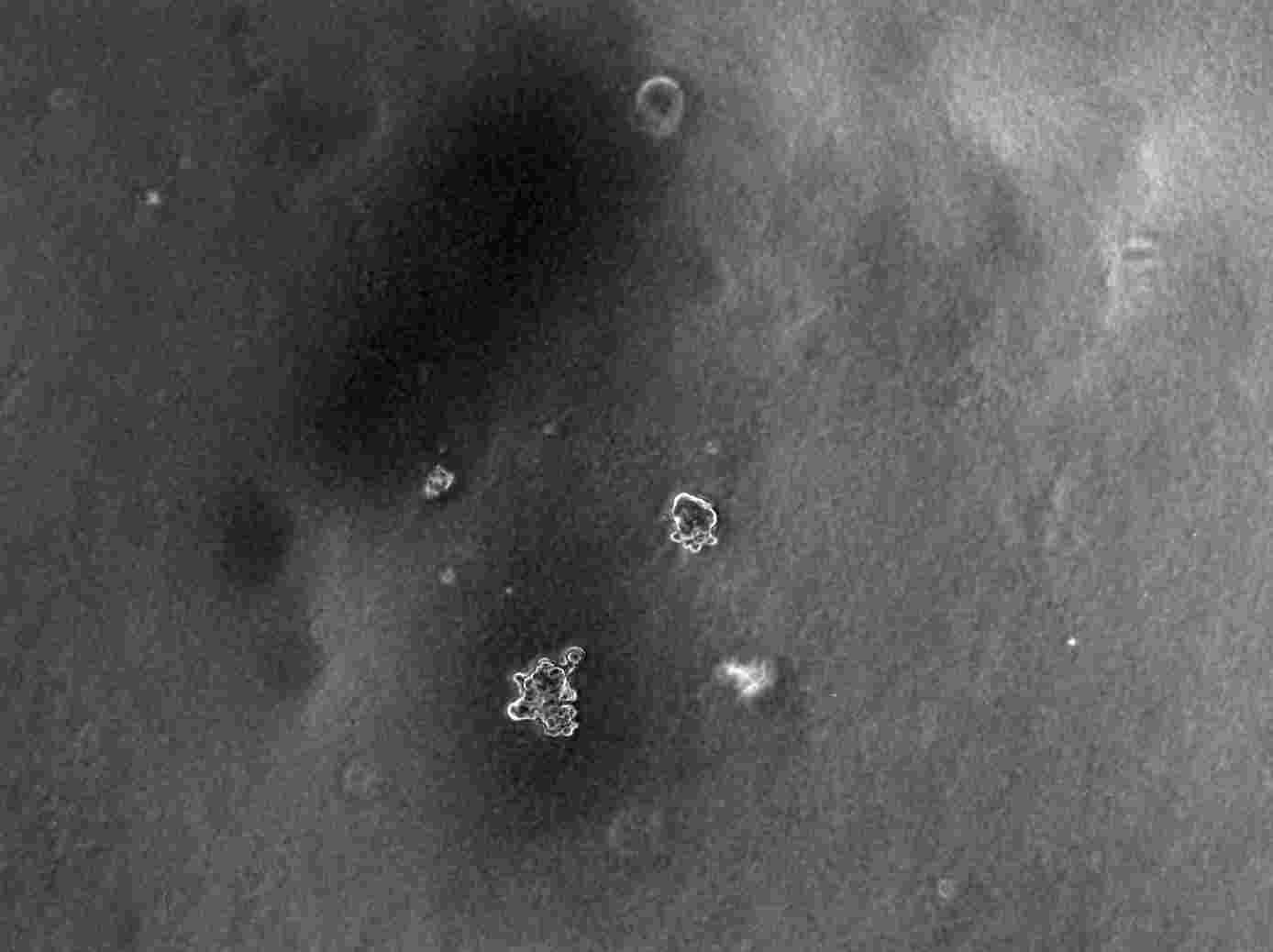

Supplement: S2 File — The raw data are presented in Raw data.zip. (ZIP) [file pone.0339611.s002.zip › Raw data/Figure 4/soft agar/day 14/3+shI-2-day14 (15).jpg]

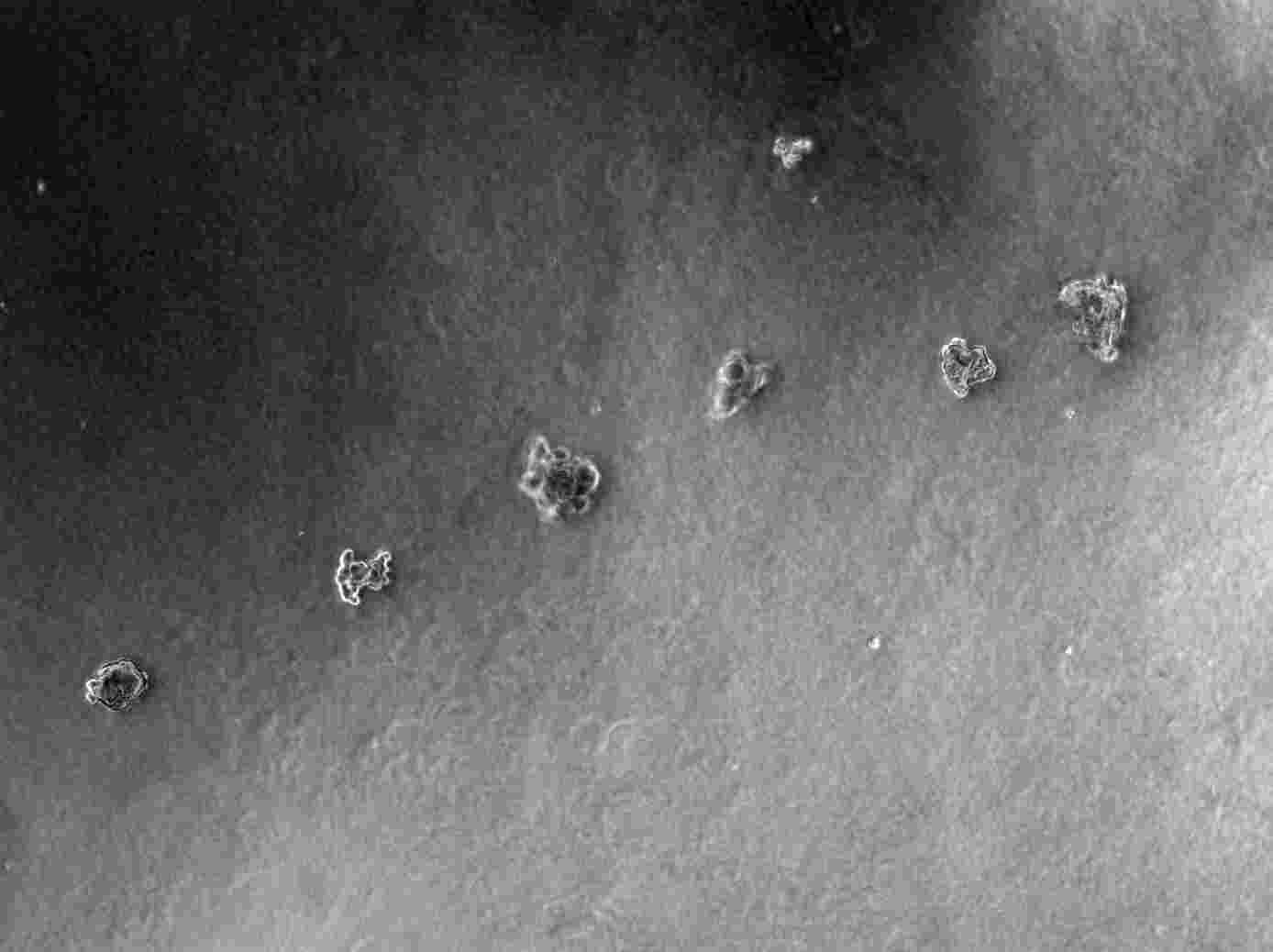

Supplement: S2 File — The raw data are presented in Raw data.zip. (ZIP) [file pone.0339611.s002.zip › Raw data/Figure 4/soft agar/day 14/3+shI-2-day14 (2).jpg]

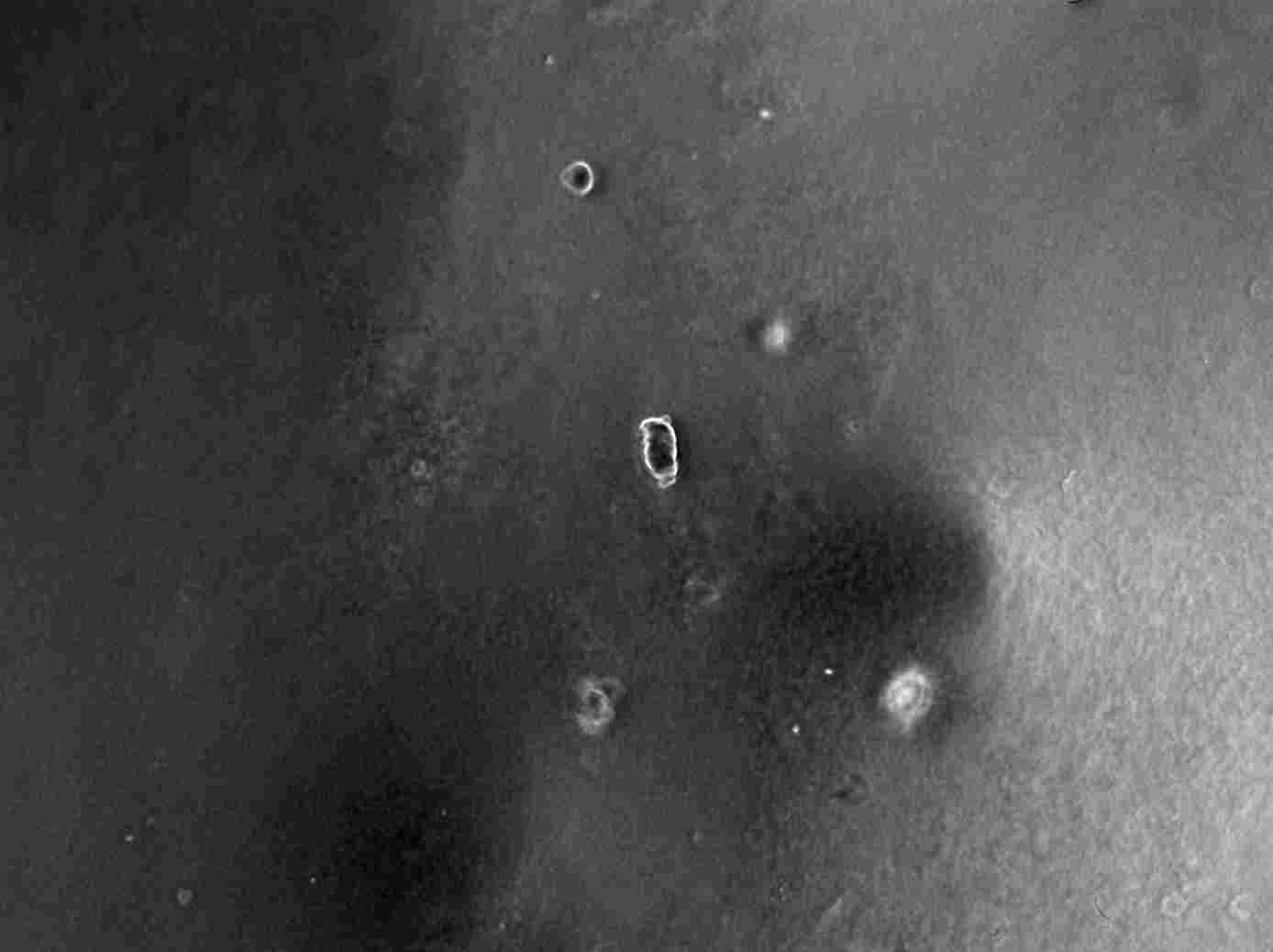

Supplement: S2 File — The raw data are presented in Raw data.zip. (ZIP) [file pone.0339611.s002.zip › Raw data/Figure 4/soft agar/day 14/3+shI-2-day14 (3).jpg]

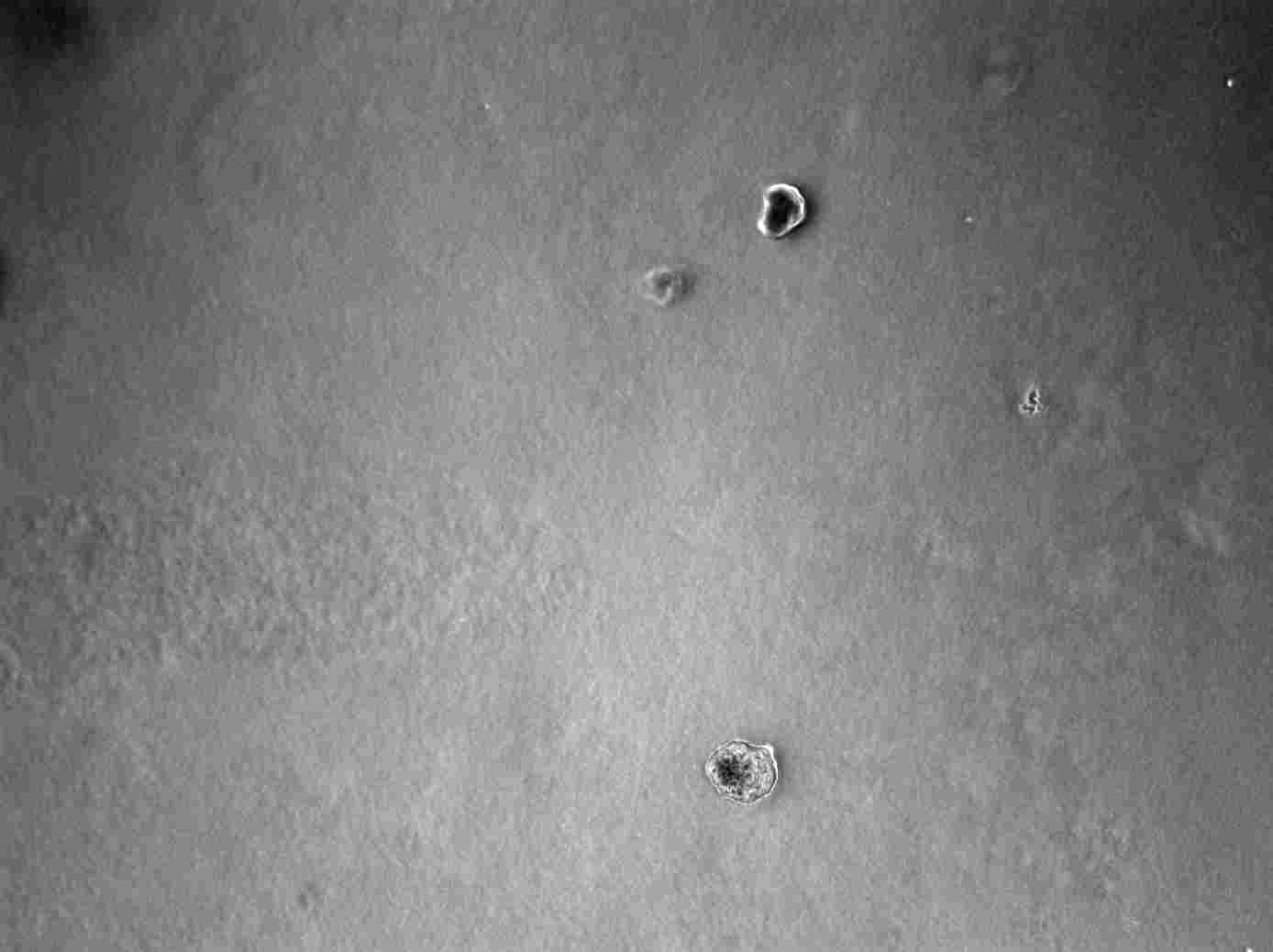

Supplement: S2 File — The raw data are presented in Raw data.zip. (ZIP) [file pone.0339611.s002.zip › Raw data/Figure 4/soft agar/day 14/3+shI-2-day14 (4).jpg]

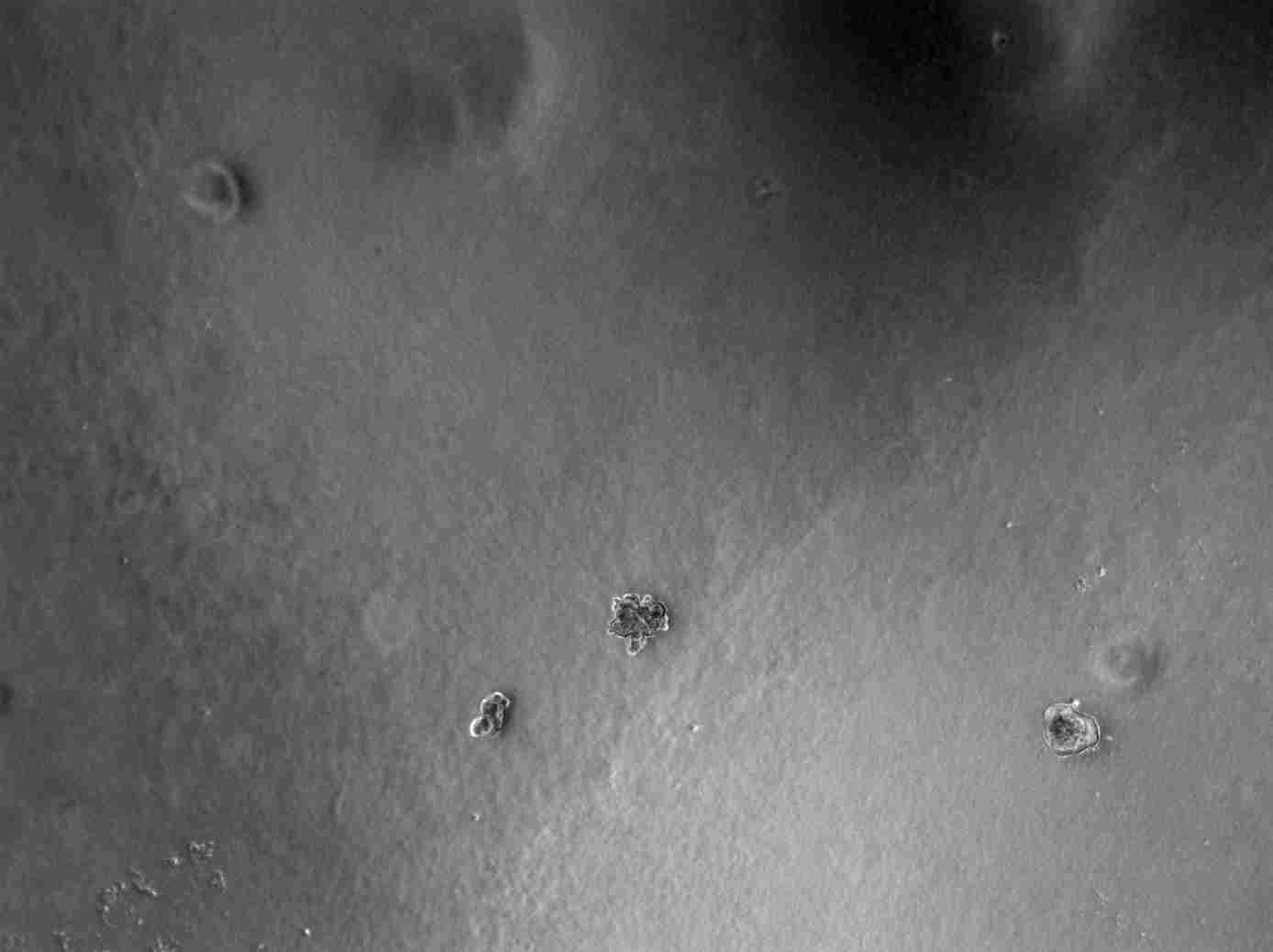

Supplement: S2 File — The raw data are presented in Raw data.zip. (ZIP) [file pone.0339611.s002.zip › Raw data/Figure 4/soft agar/day 14/3+shI-2-day14 (5).jpg]

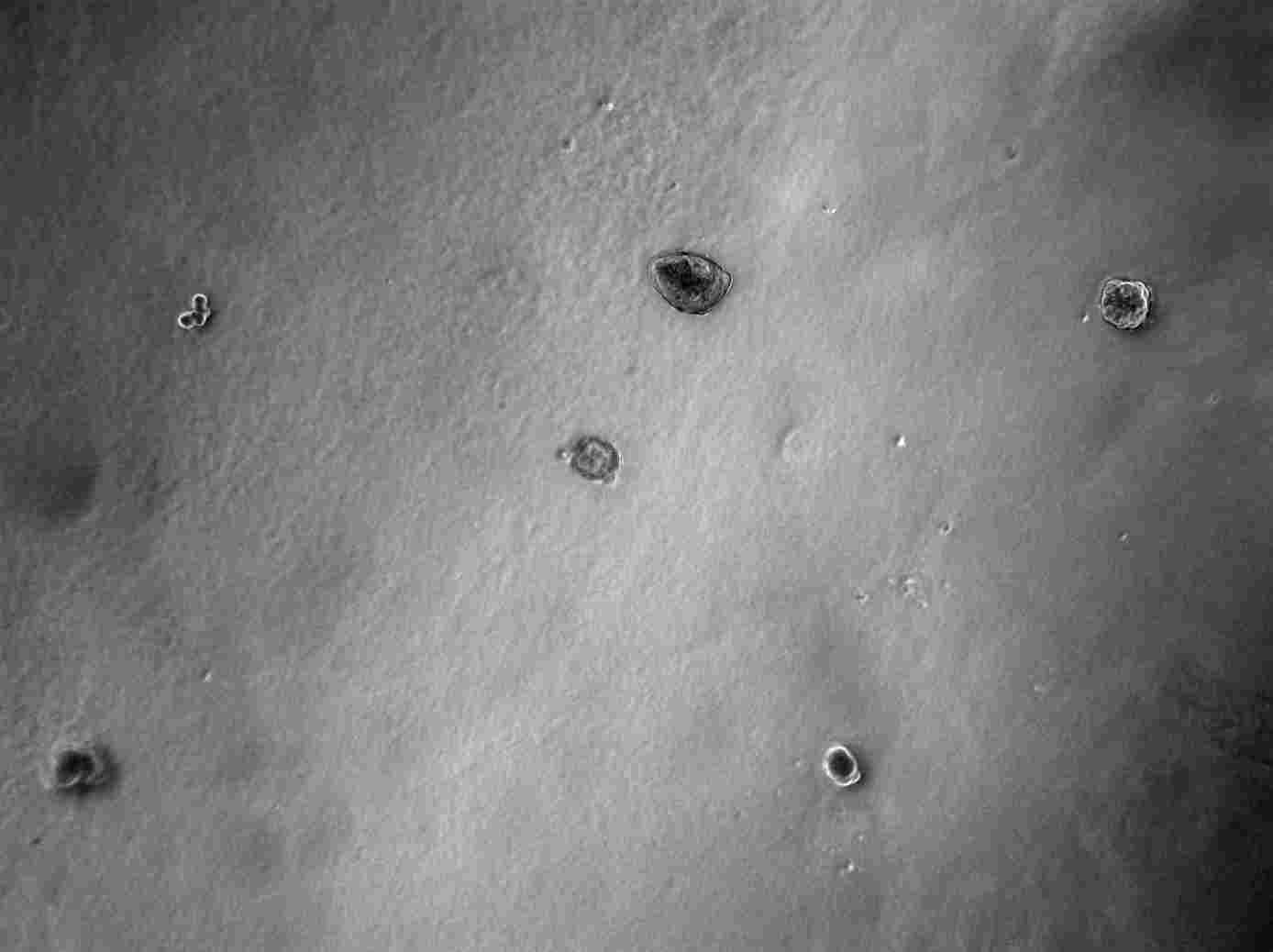

Supplement: S2 File — The raw data are presented in Raw data.zip. (ZIP) [file pone.0339611.s002.zip › Raw data/Figure 4/soft agar/day 14/3+shI-2-day14 (6).jpg]

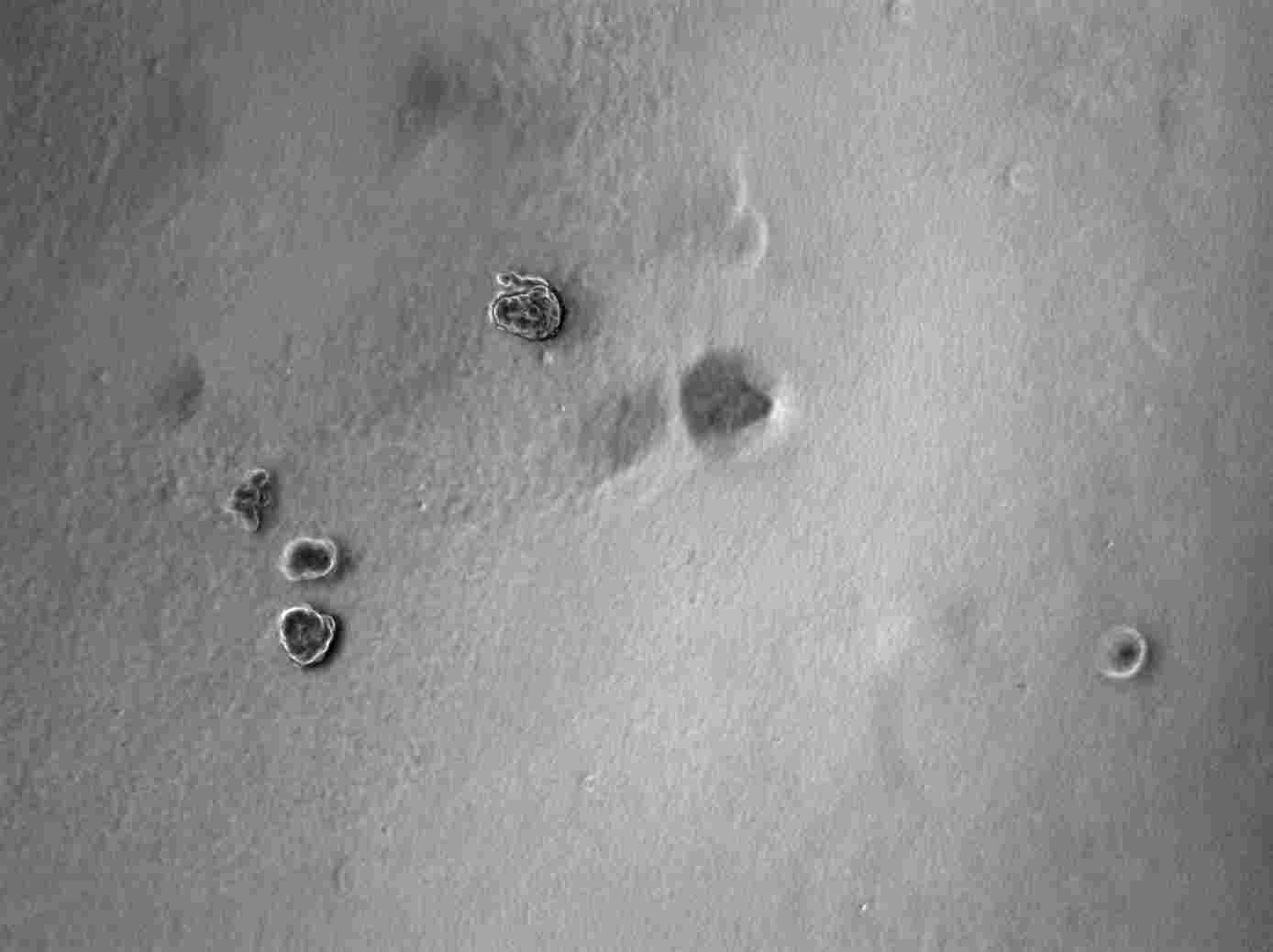

Supplement: S2 File — The raw data are presented in Raw data.zip. (ZIP) [file pone.0339611.s002.zip › Raw data/Figure 4/soft agar/day 14/3+shI-2-day14 (7).jpg]

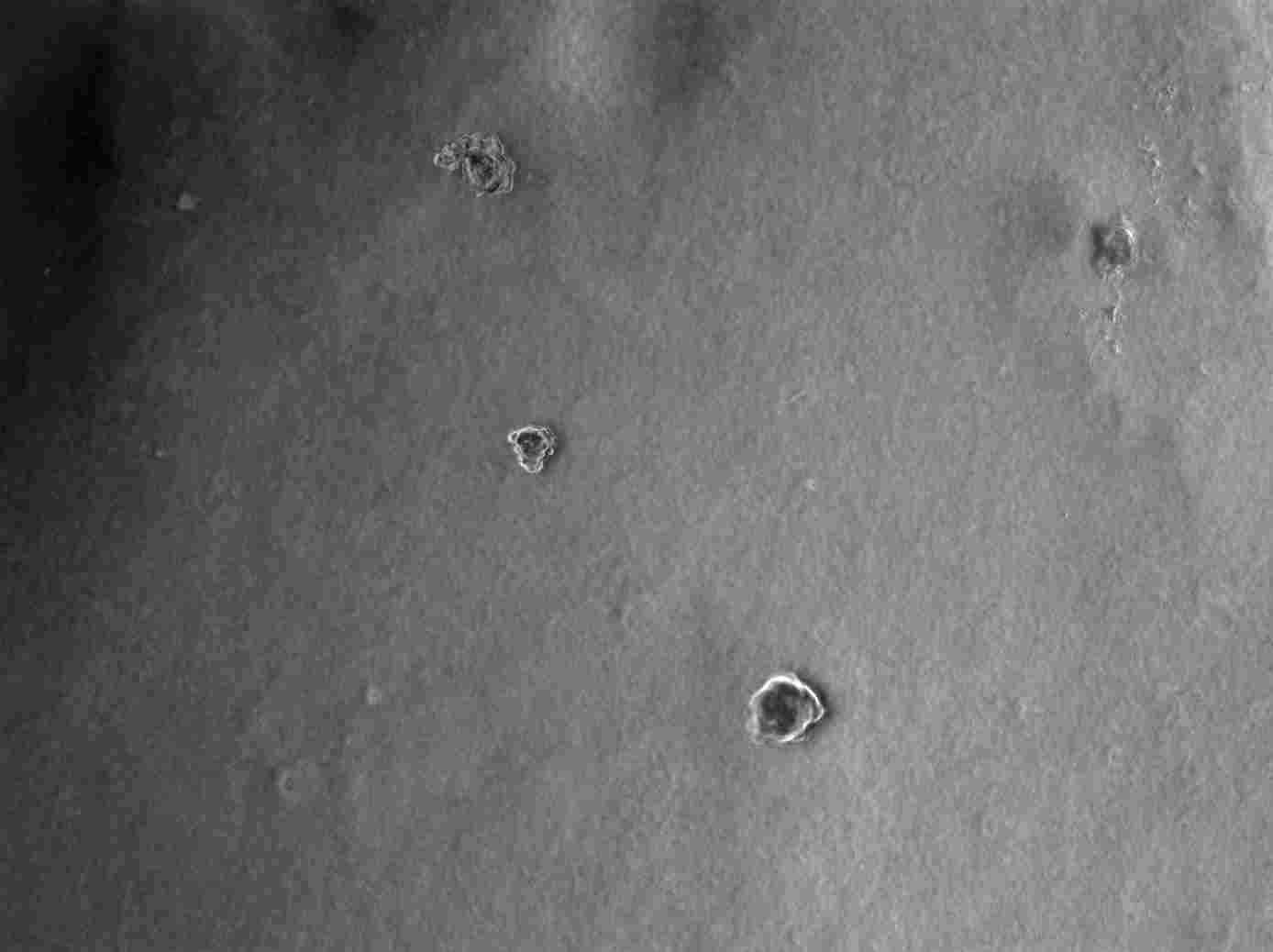

Supplement: S2 File — The raw data are presented in Raw data.zip. (ZIP) [file pone.0339611.s002.zip › Raw data/Figure 4/soft agar/day 14/3+shI-2-day14 (8).jpg]

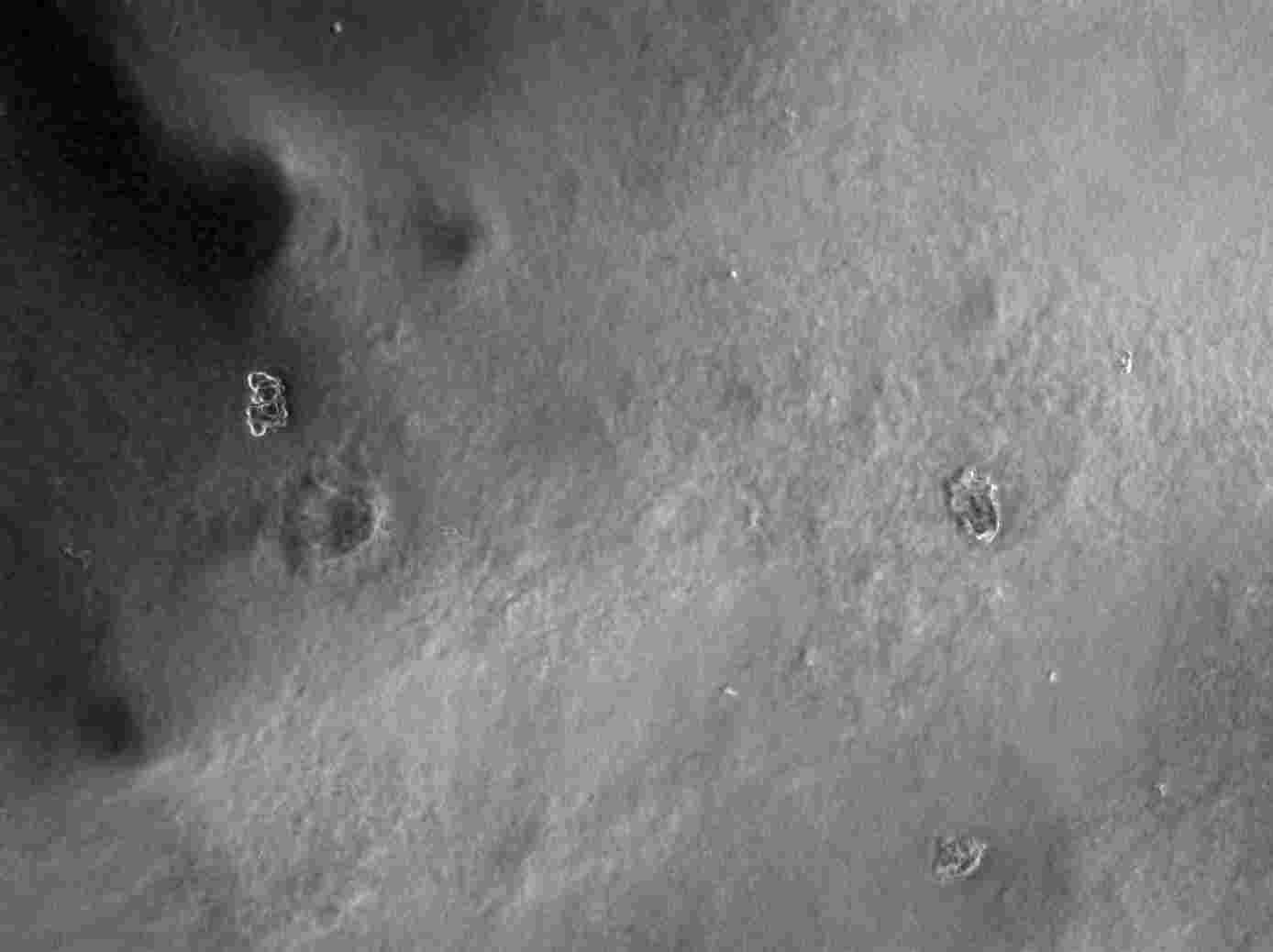

Supplement: S2 File — The raw data are presented in Raw data.zip. (ZIP) [file pone.0339611.s002.zip › Raw data/Figure 4/soft agar/day 14/3+shI-2-day14 (9).jpg]

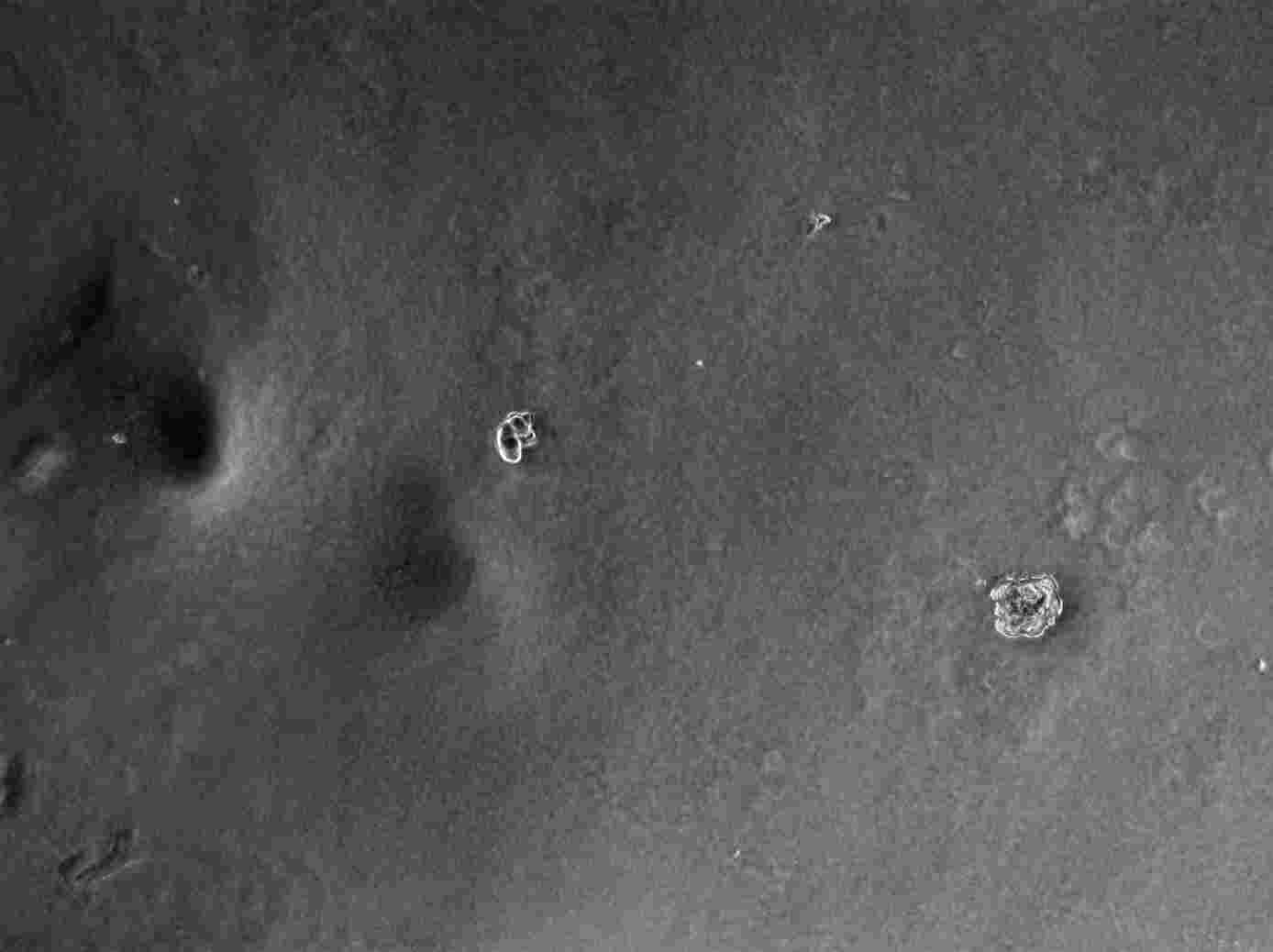

Supplement: S2 File — The raw data are presented in Raw data.zip. (ZIP) [file pone.0339611.s002.zip › Raw data/Figure 4/soft agar/day 14/3+shI-2-day14.jpg]

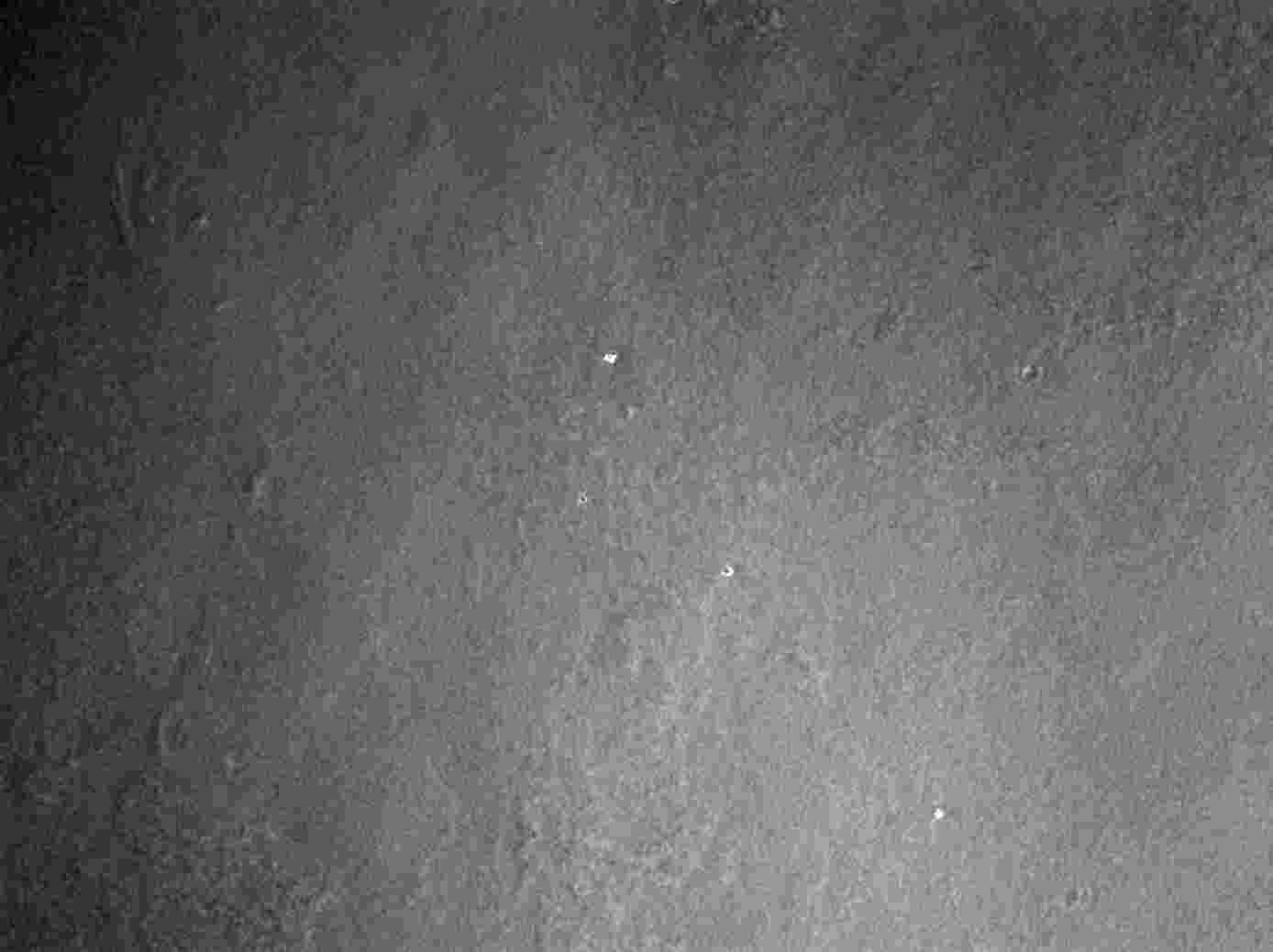

Supplement: S2 File — The raw data are presented in Raw data.zip. (ZIP) [file pone.0339611.s002.zip › Raw data/Figure 4/soft agar/day 14/3+shscr-day14 (10).jpg]

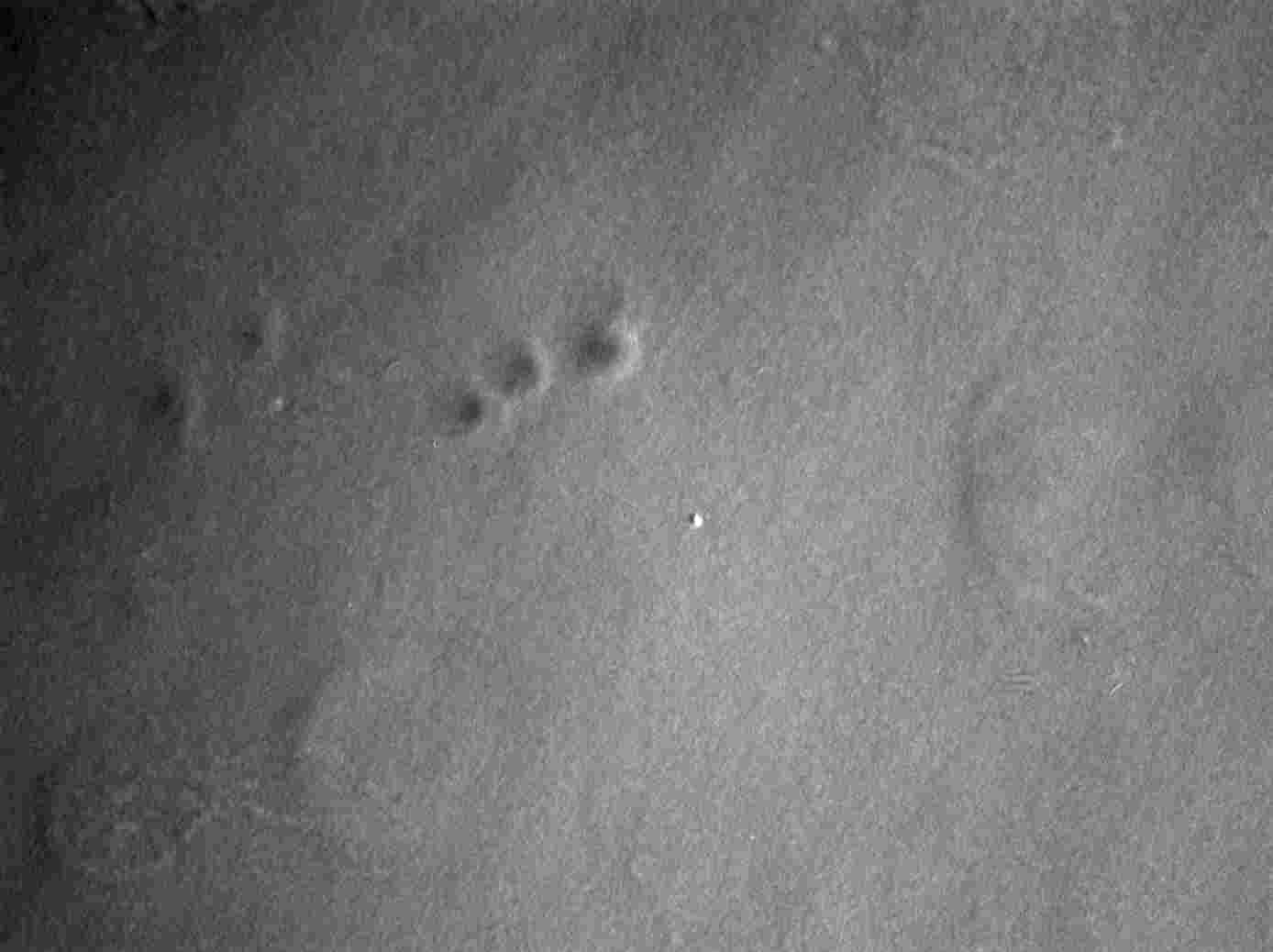

Supplement: S2 File — The raw data are presented in Raw data.zip. (ZIP) [file pone.0339611.s002.zip › Raw data/Figure 4/soft agar/day 14/3+shscr-day14 (11).jpg]

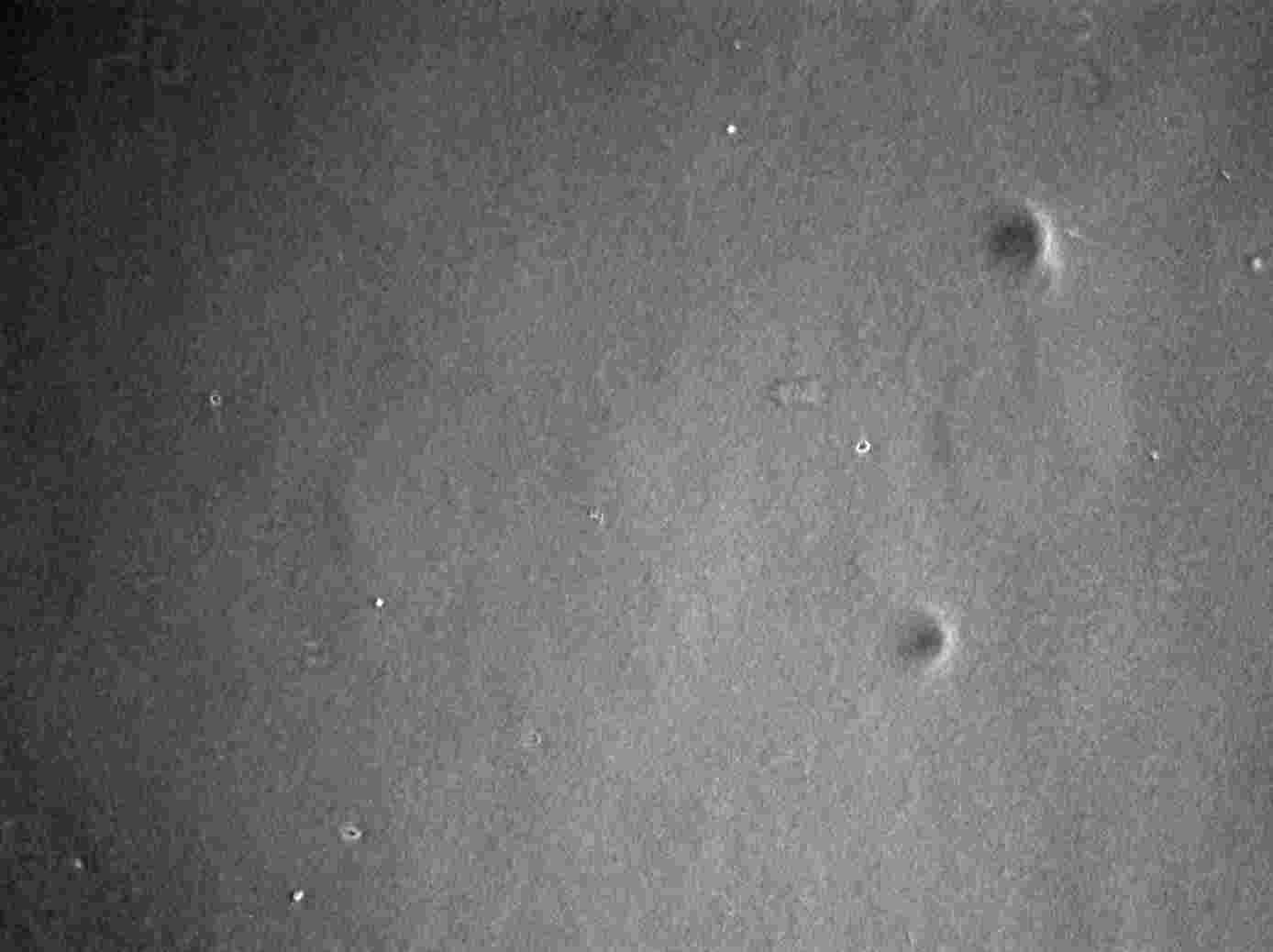

Supplement: S2 File — The raw data are presented in Raw data.zip. (ZIP) [file pone.0339611.s002.zip › Raw data/Figure 4/soft agar/day 14/3+shscr-day14 (12).jpg]

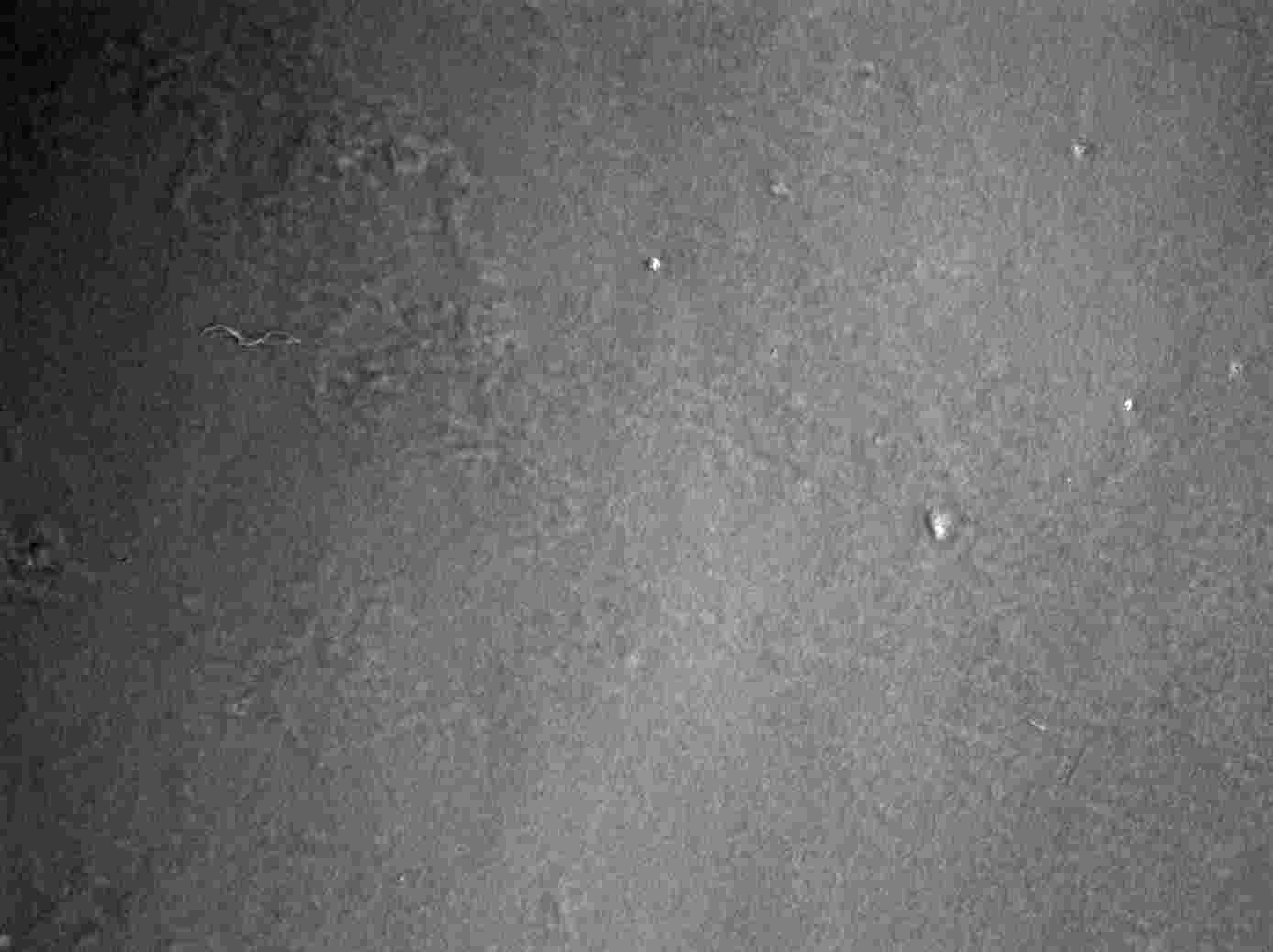

Supplement: S2 File — The raw data are presented in Raw data.zip. (ZIP) [file pone.0339611.s002.zip › Raw data/Figure 4/soft agar/day 14/3+shscr-day14 (13).jpg]

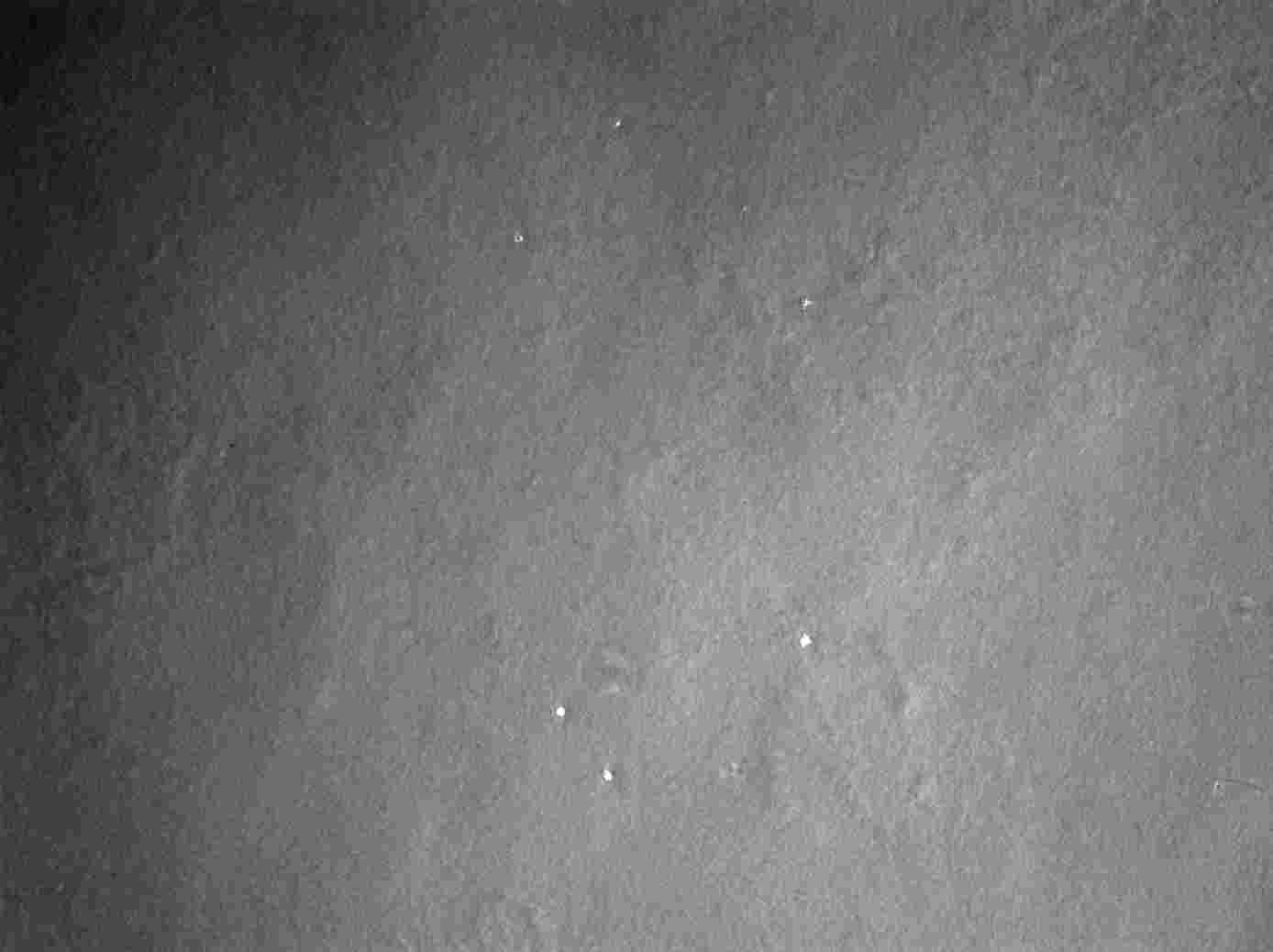

Supplement: S2 File — The raw data are presented in Raw data.zip. (ZIP) [file pone.0339611.s002.zip › Raw data/Figure 4/soft agar/day 14/3+shscr-day14 (14).jpg]

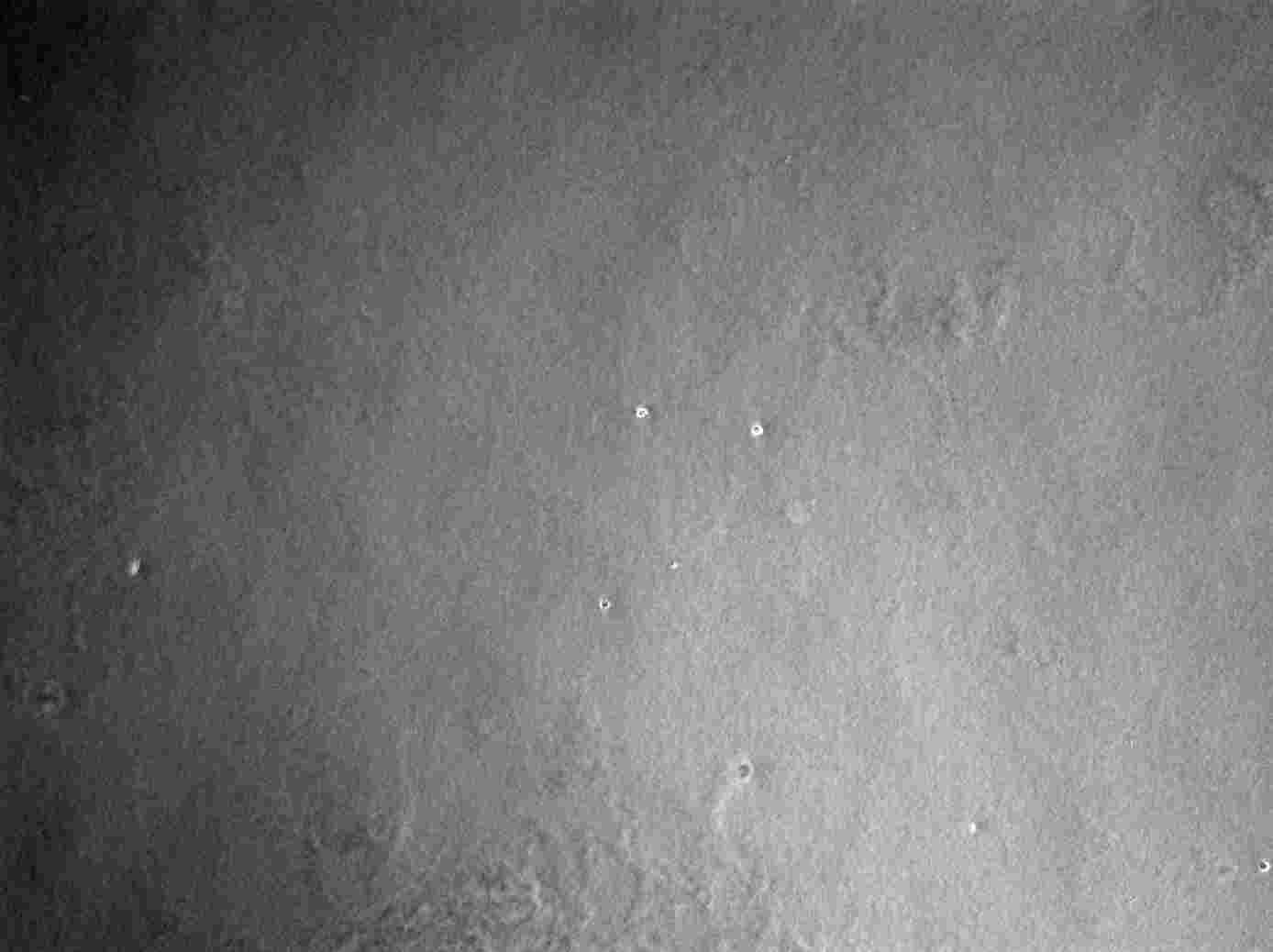

Supplement: S2 File — The raw data are presented in Raw data.zip. (ZIP) [file pone.0339611.s002.zip › Raw data/Figure 4/soft agar/day 14/3+shscr-day14 (15).jpg]

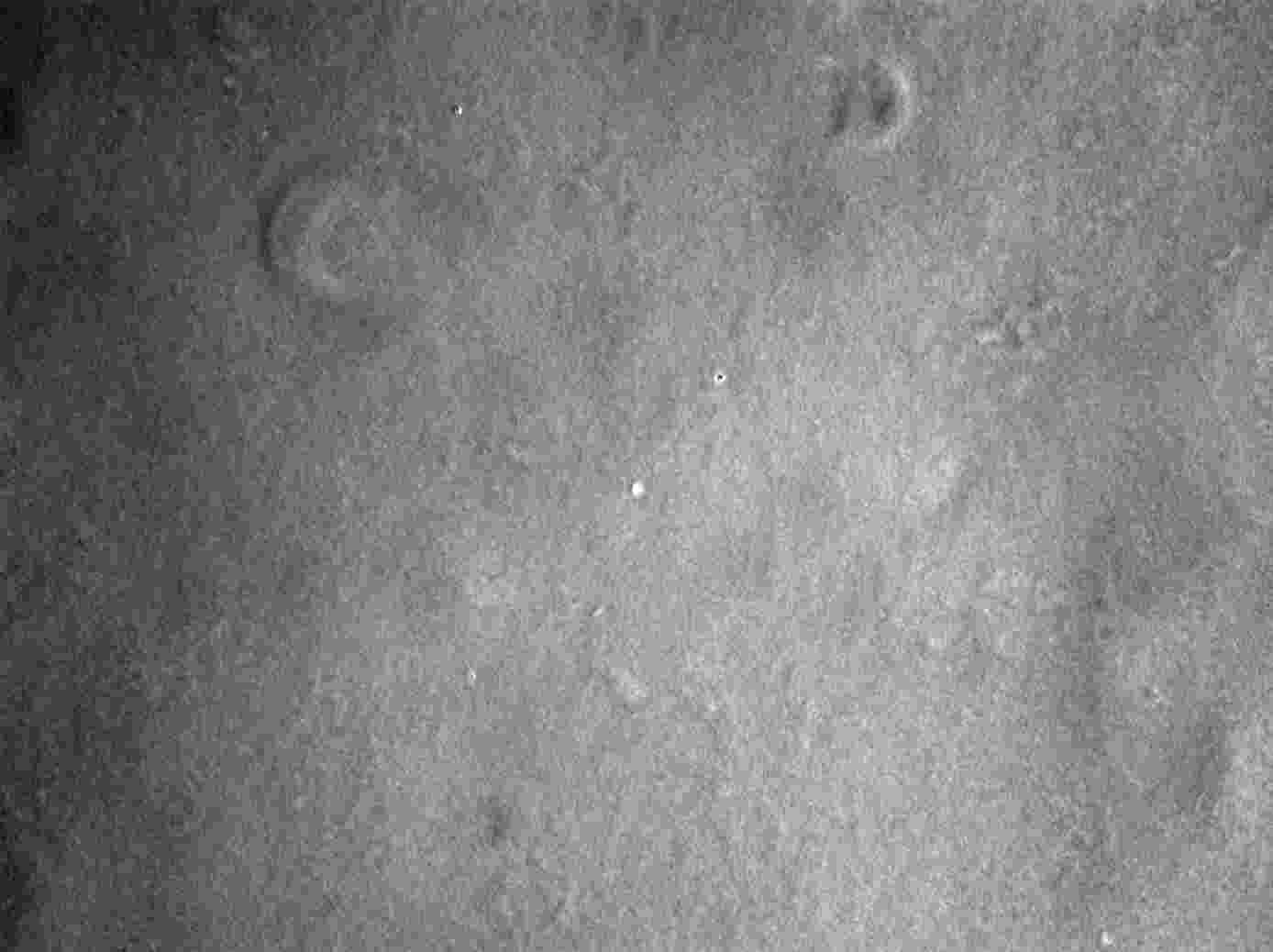

Supplement: S2 File — The raw data are presented in Raw data.zip. (ZIP) [file pone.0339611.s002.zip › Raw data/Figure 4/soft agar/day 14/3+shscr-day14 (2).jpg]

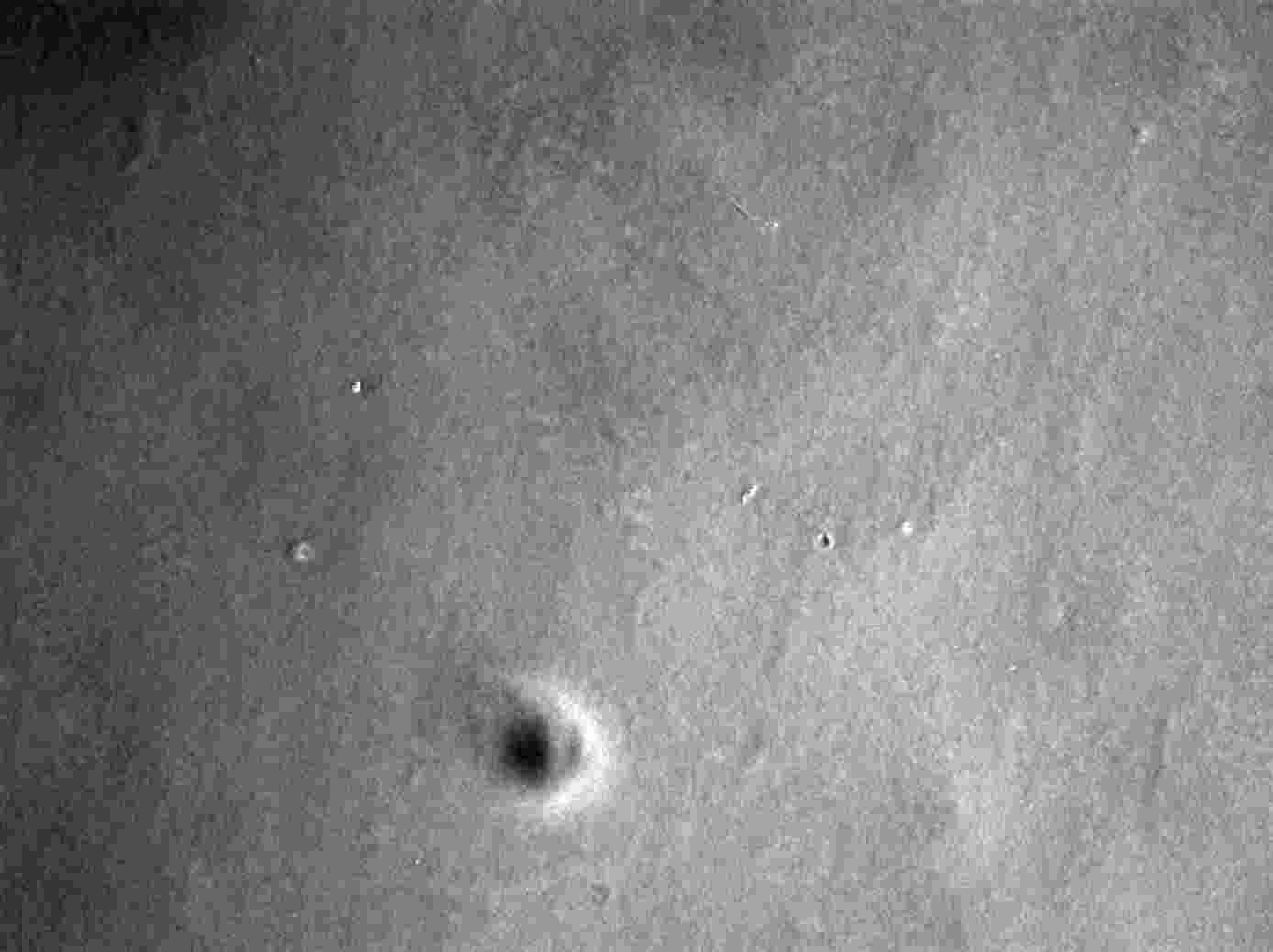

Supplement: S2 File — The raw data are presented in Raw data.zip. (ZIP) [file pone.0339611.s002.zip › Raw data/Figure 4/soft agar/day 14/3+shscr-day14 (3).jpg]

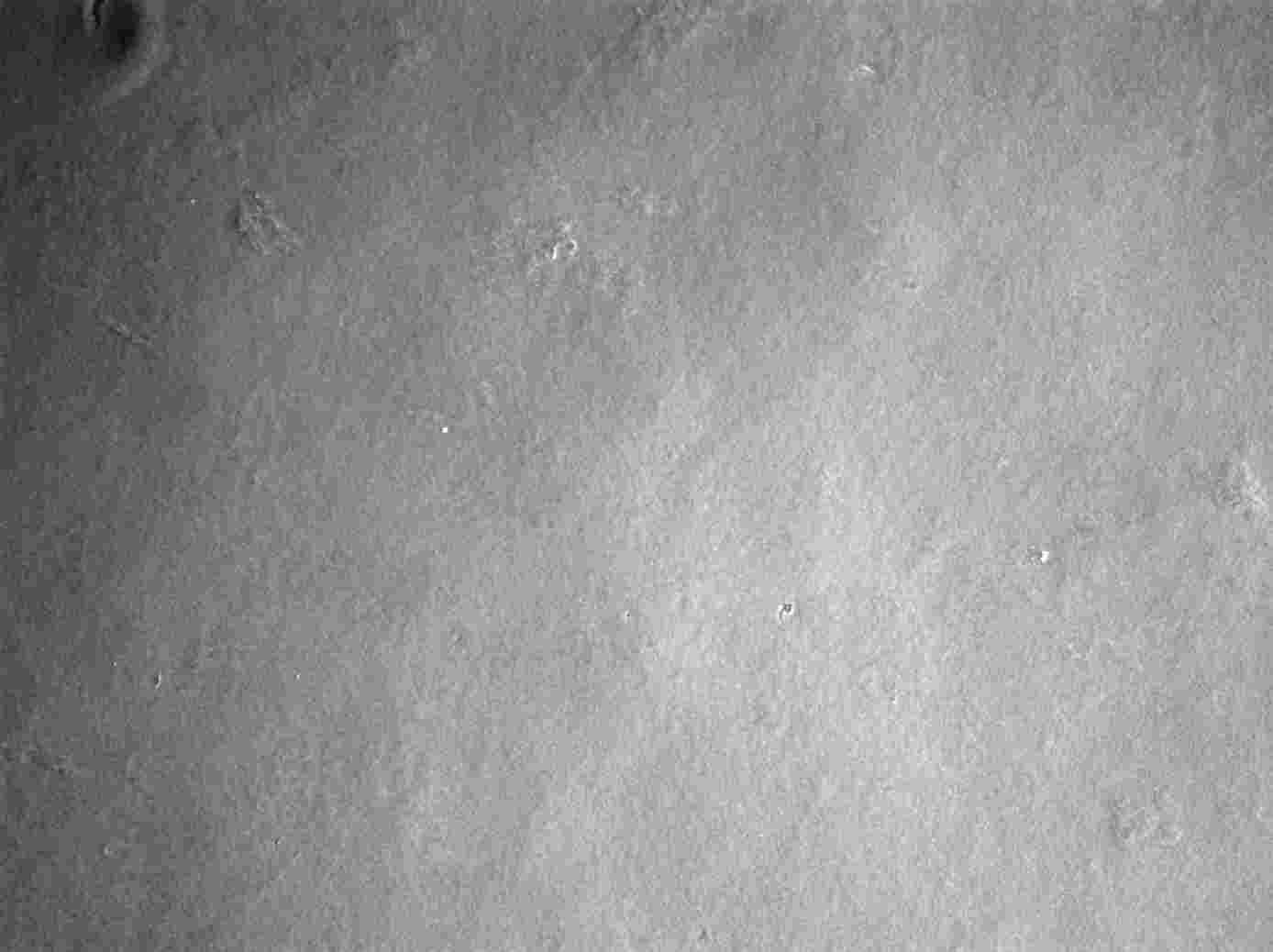

Supplement: S2 File — The raw data are presented in Raw data.zip. (ZIP) [file pone.0339611.s002.zip › Raw data/Figure 4/soft agar/day 14/3+shscr-day14 (4).jpg]

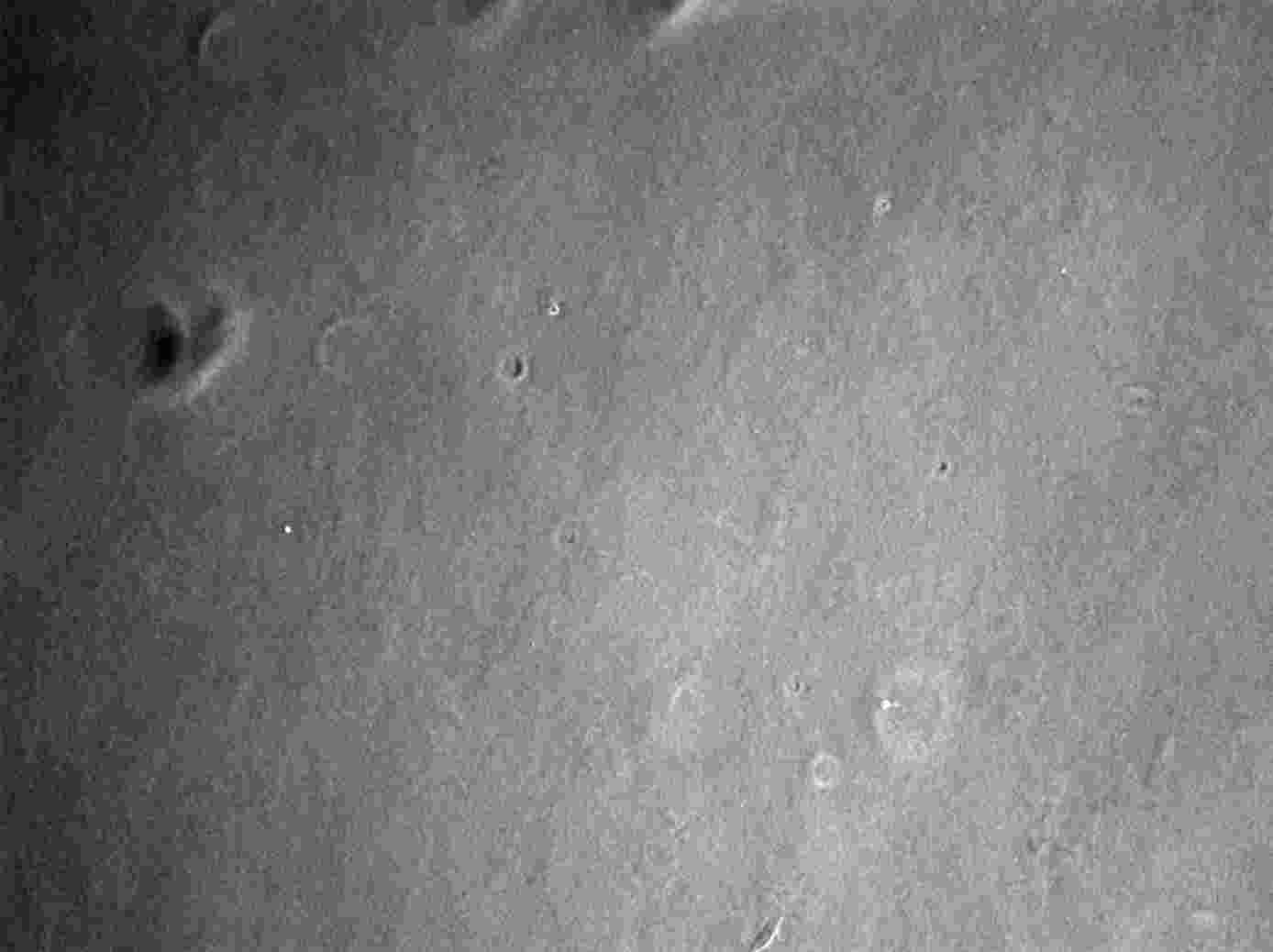

Supplement: S2 File — The raw data are presented in Raw data.zip. (ZIP) [file pone.0339611.s002.zip › Raw data/Figure 4/soft agar/day 14/3+shscr-day14 (5).jpg]

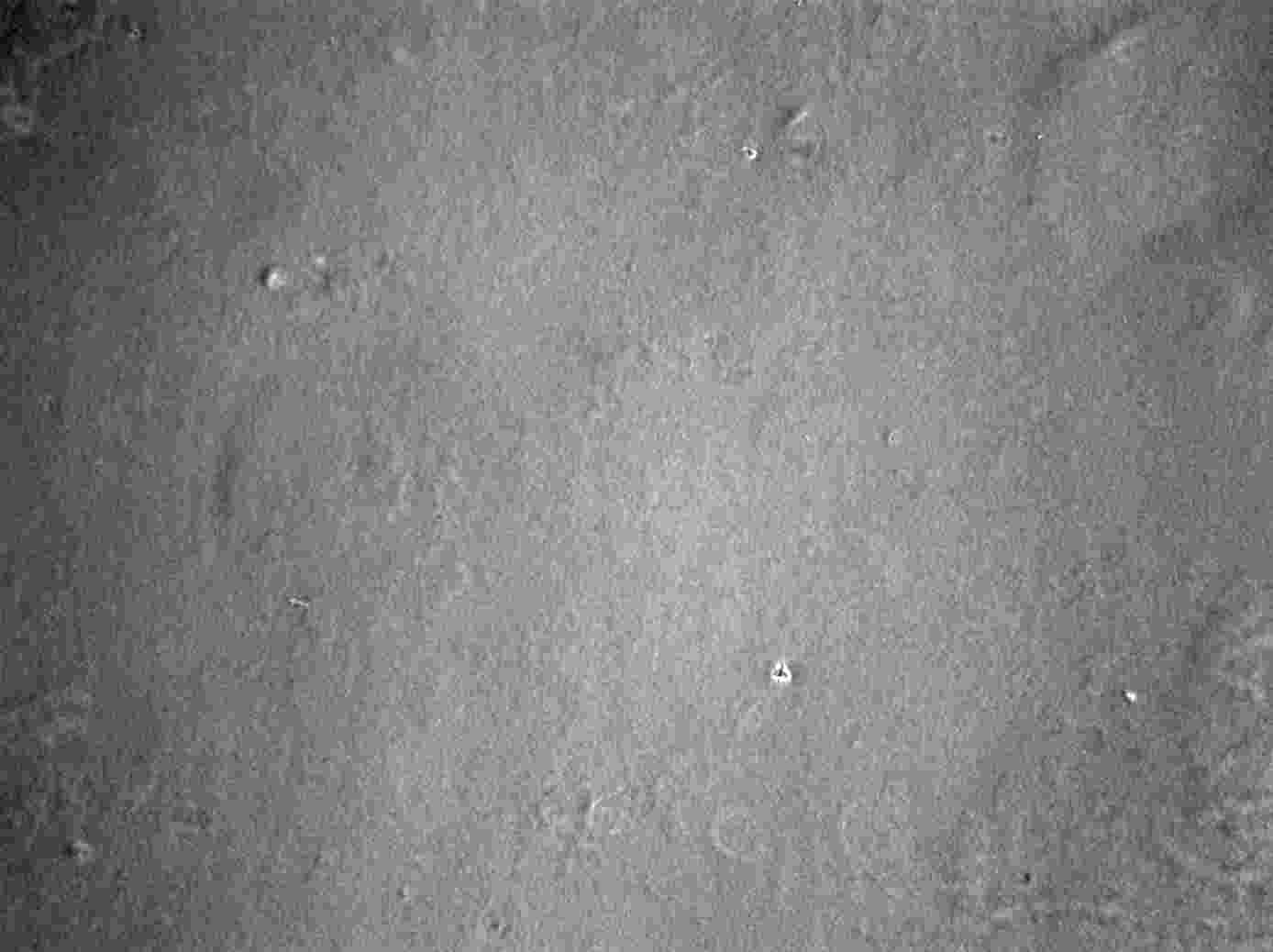

Supplement: S2 File — The raw data are presented in Raw data.zip. (ZIP) [file pone.0339611.s002.zip › Raw data/Figure 4/soft agar/day 14/3+shscr-day14 (6).jpg]

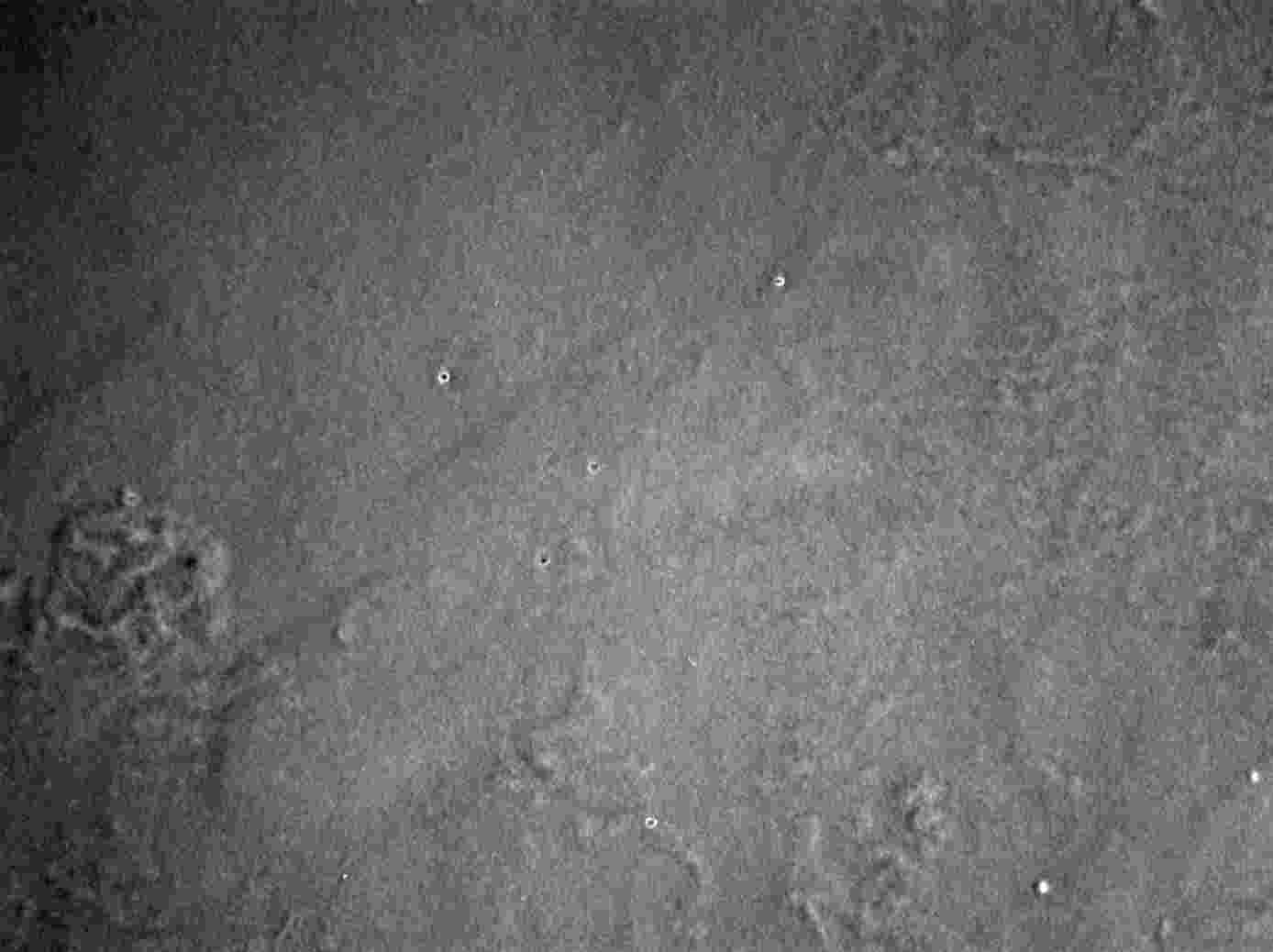

Supplement: S2 File — The raw data are presented in Raw data.zip. (ZIP) [file pone.0339611.s002.zip › Raw data/Figure 4/soft agar/day 14/3+shscr-day14 (7).jpg]

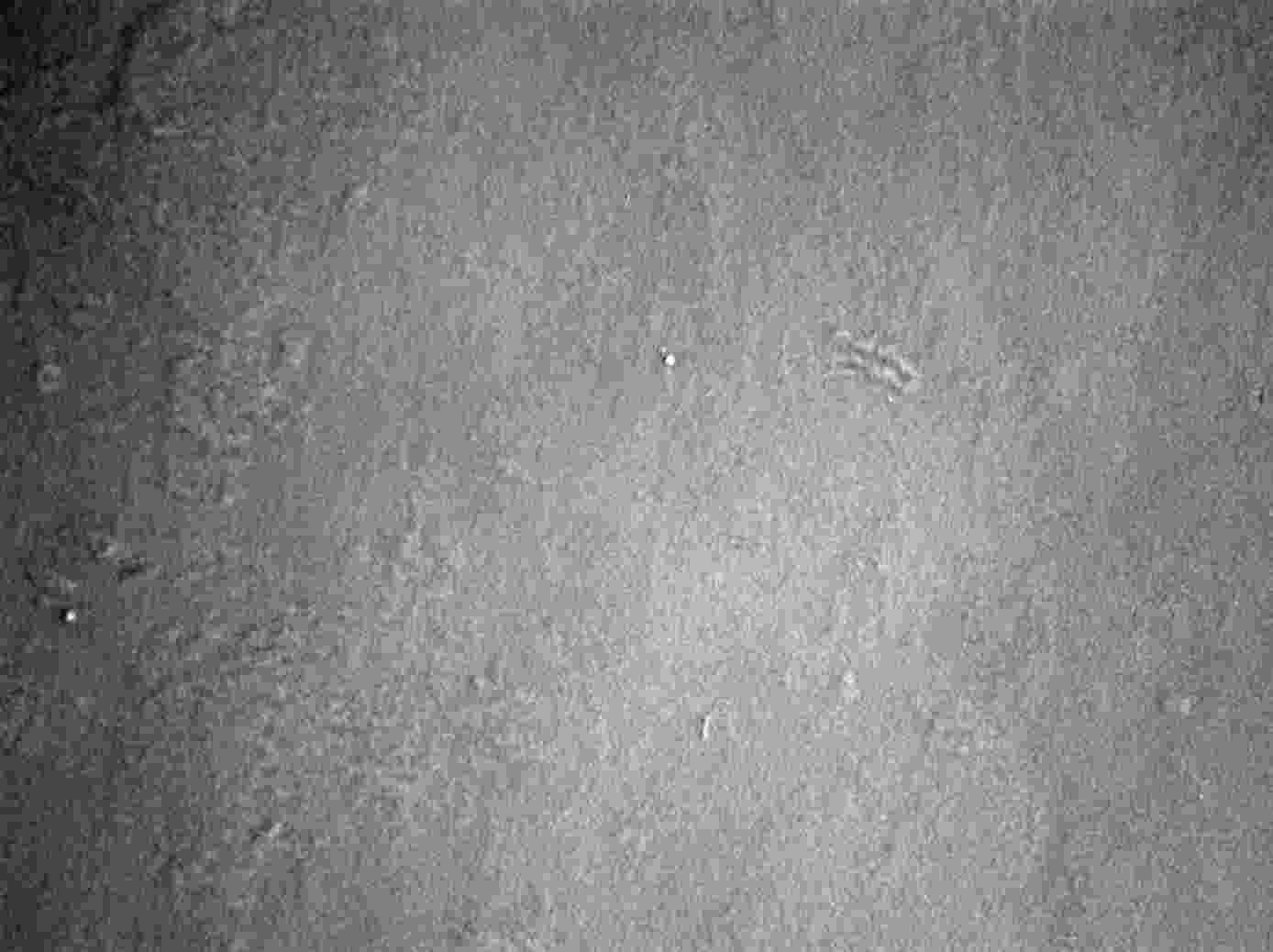

Supplement: S2 File — The raw data are presented in Raw data.zip. (ZIP) [file pone.0339611.s002.zip › Raw data/Figure 4/soft agar/day 14/3+shscr-day14 (8).jpg]

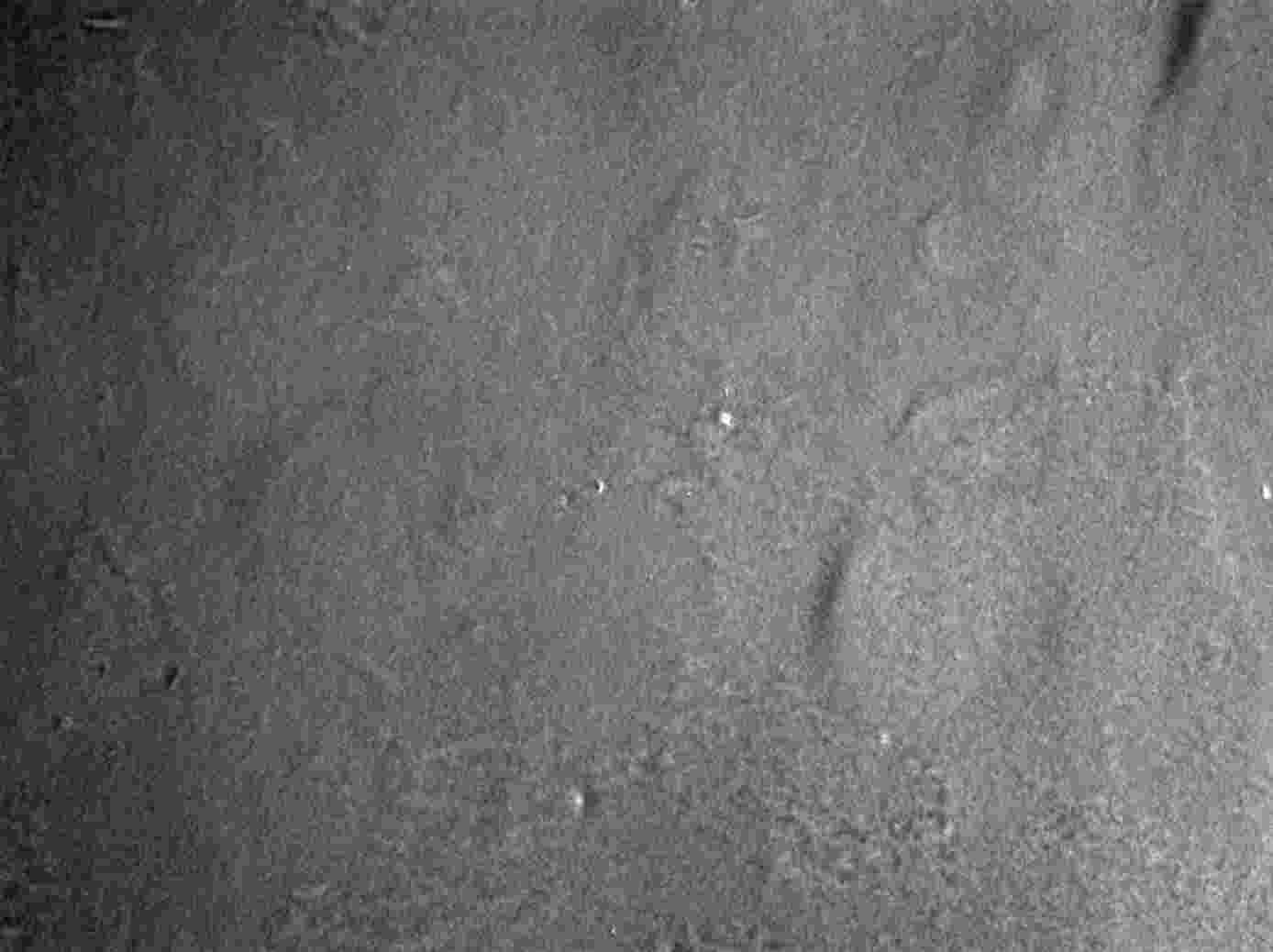

Supplement: S2 File — The raw data are presented in Raw data.zip. (ZIP) [file pone.0339611.s002.zip › Raw data/Figure 4/soft agar/day 14/3+shscr-day14 (9).jpg]

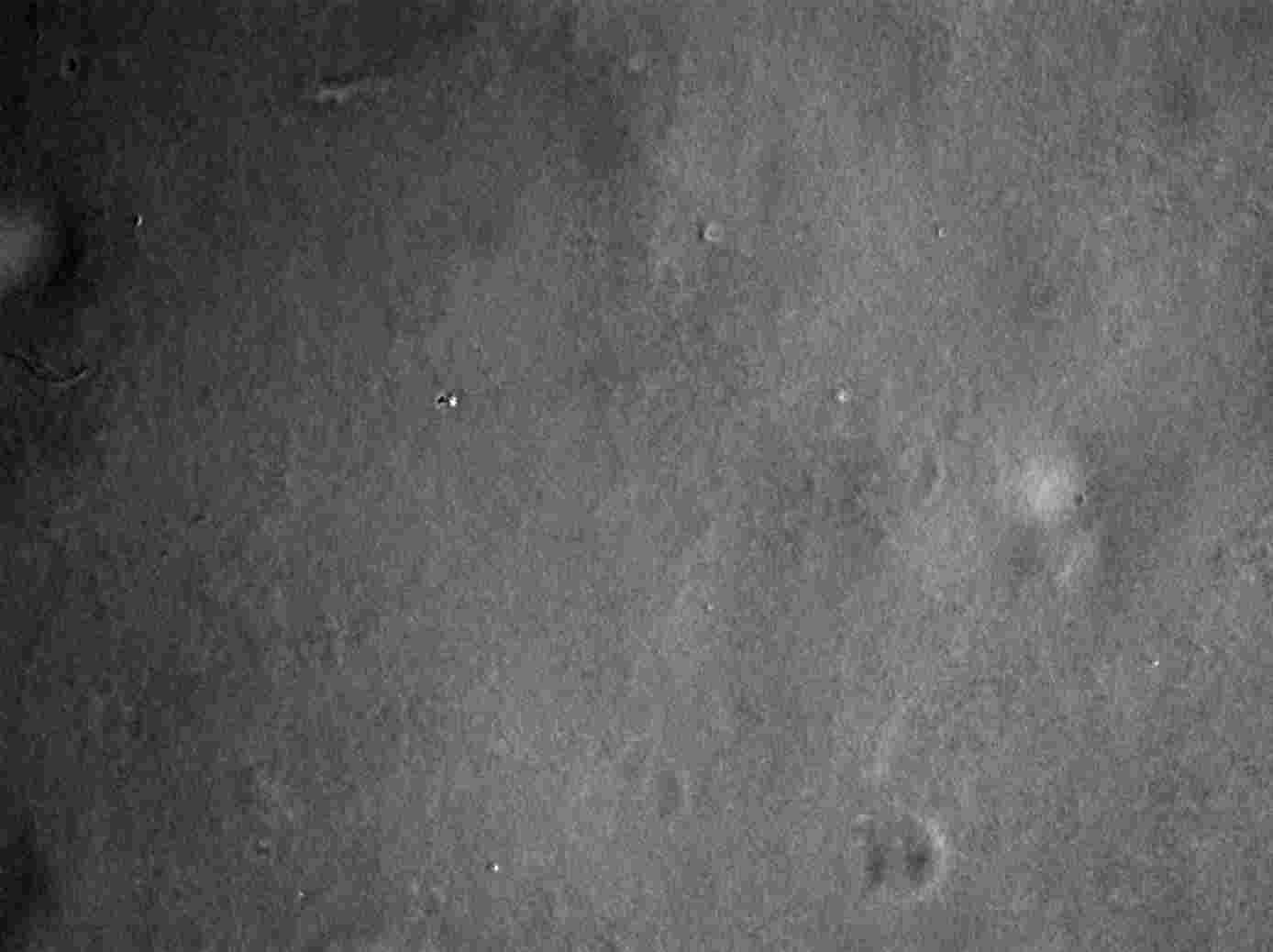

Supplement: S2 File — The raw data are presented in Raw data.zip. (ZIP) [file pone.0339611.s002.zip › Raw data/Figure 4/soft agar/day 14/3+shscr-day14.jpg]

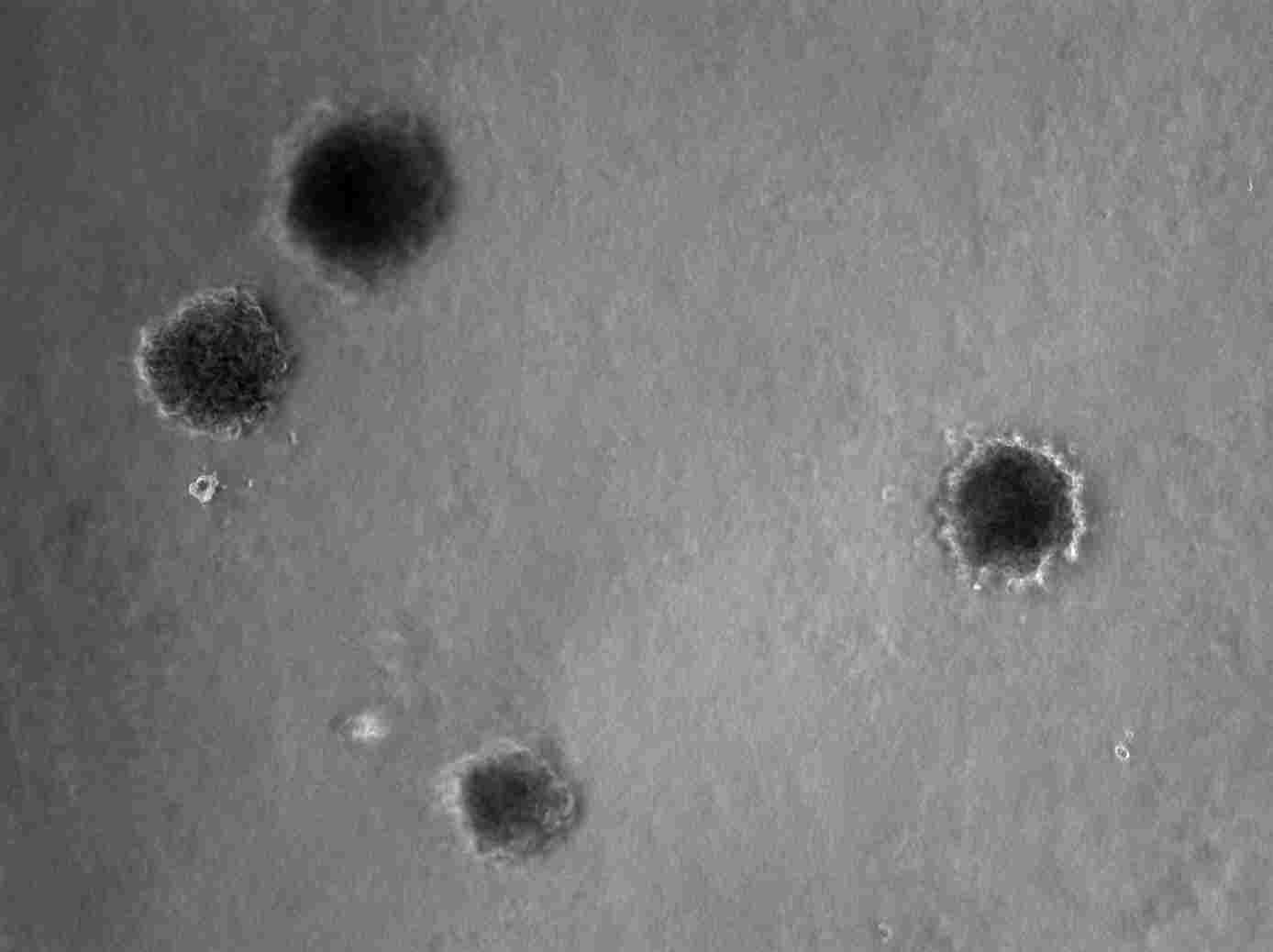

Supplement: S2 File — The raw data are presented in Raw data.zip. (ZIP) [file pone.0339611.s002.zip › Raw data/Figure 4/soft agar/day 14/4+EV-day14 (10).jpg]

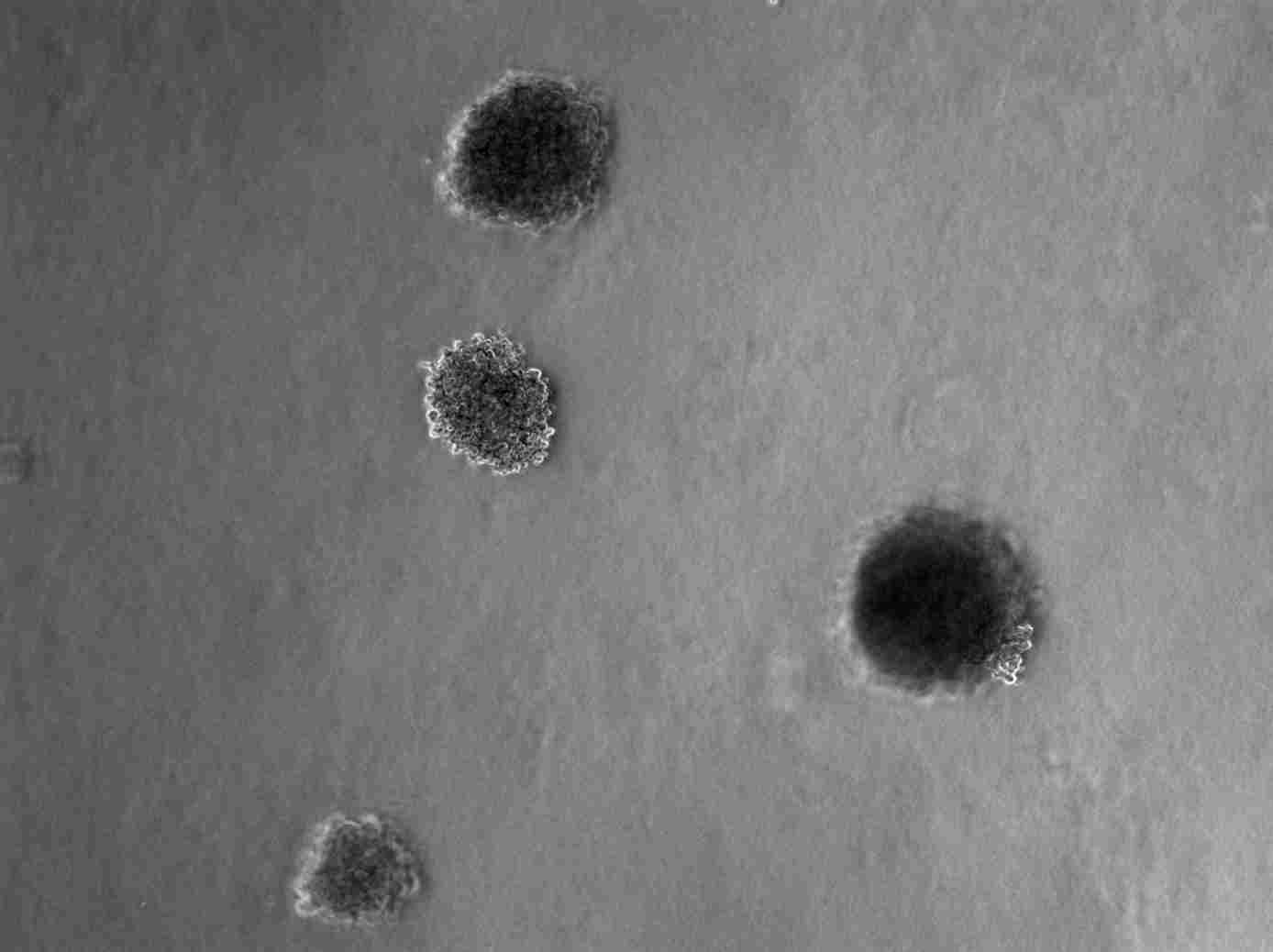

Supplement: S2 File — The raw data are presented in Raw data.zip. (ZIP) [file pone.0339611.s002.zip › Raw data/Figure 4/soft agar/day 14/4+EV-day14 (11).jpg]

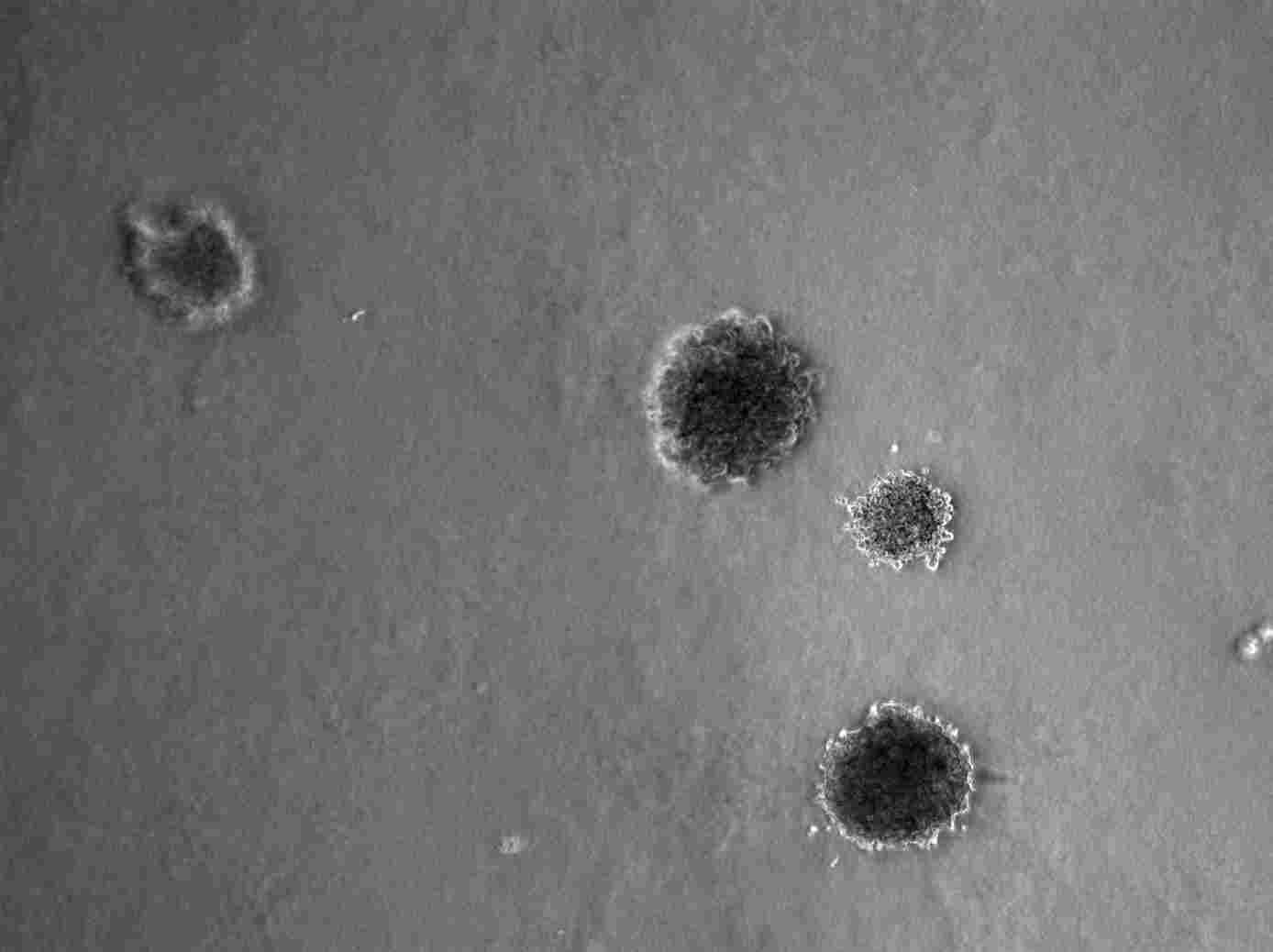

Supplement: S2 File — The raw data are presented in Raw data.zip. (ZIP) [file pone.0339611.s002.zip › Raw data/Figure 4/soft agar/day 14/4+EV-day14 (12).jpg]

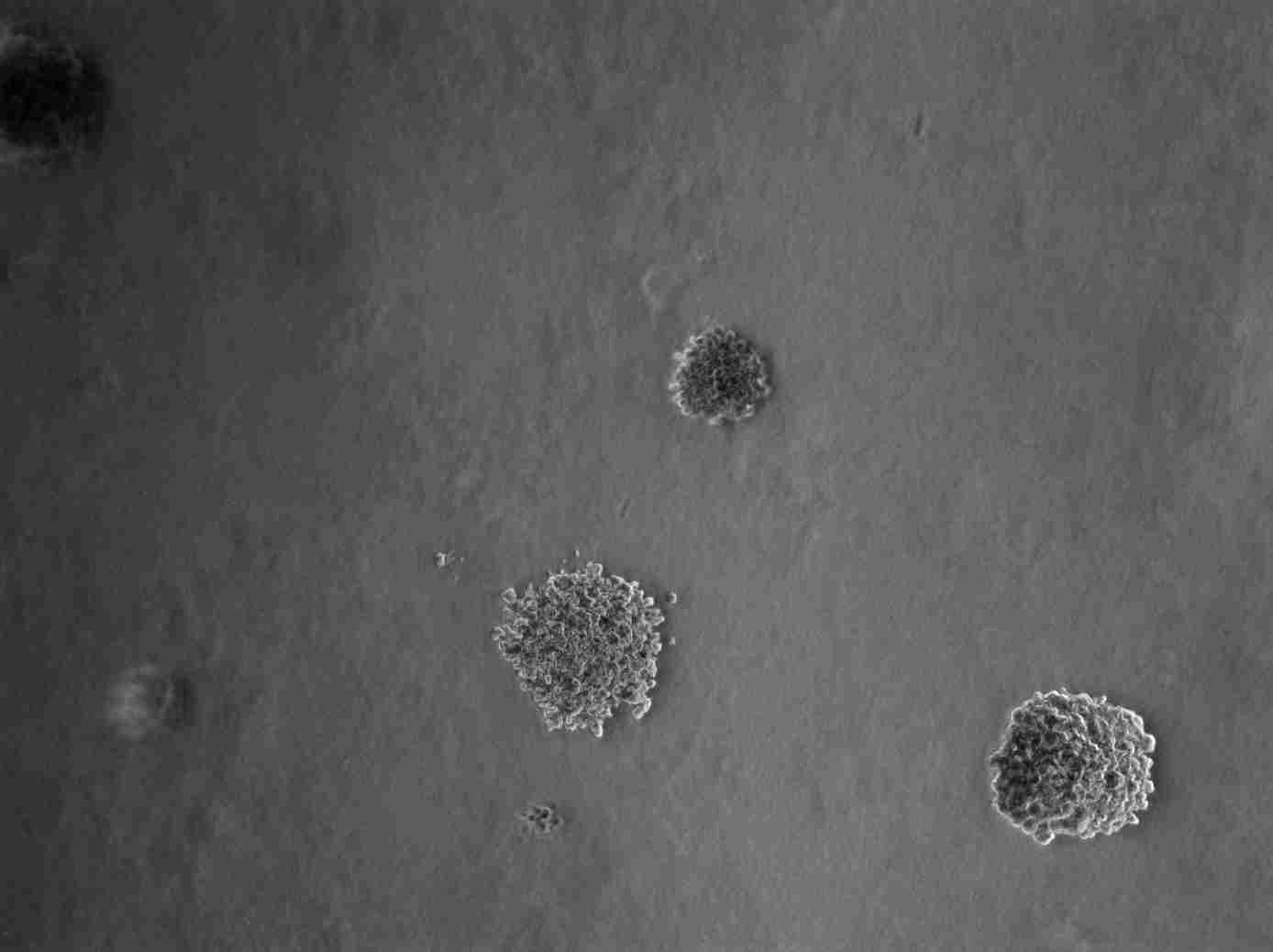

Supplement: S2 File — The raw data are presented in Raw data.zip. (ZIP) [file pone.0339611.s002.zip › Raw data/Figure 4/soft agar/day 14/4+EV-day14 (13).jpg]

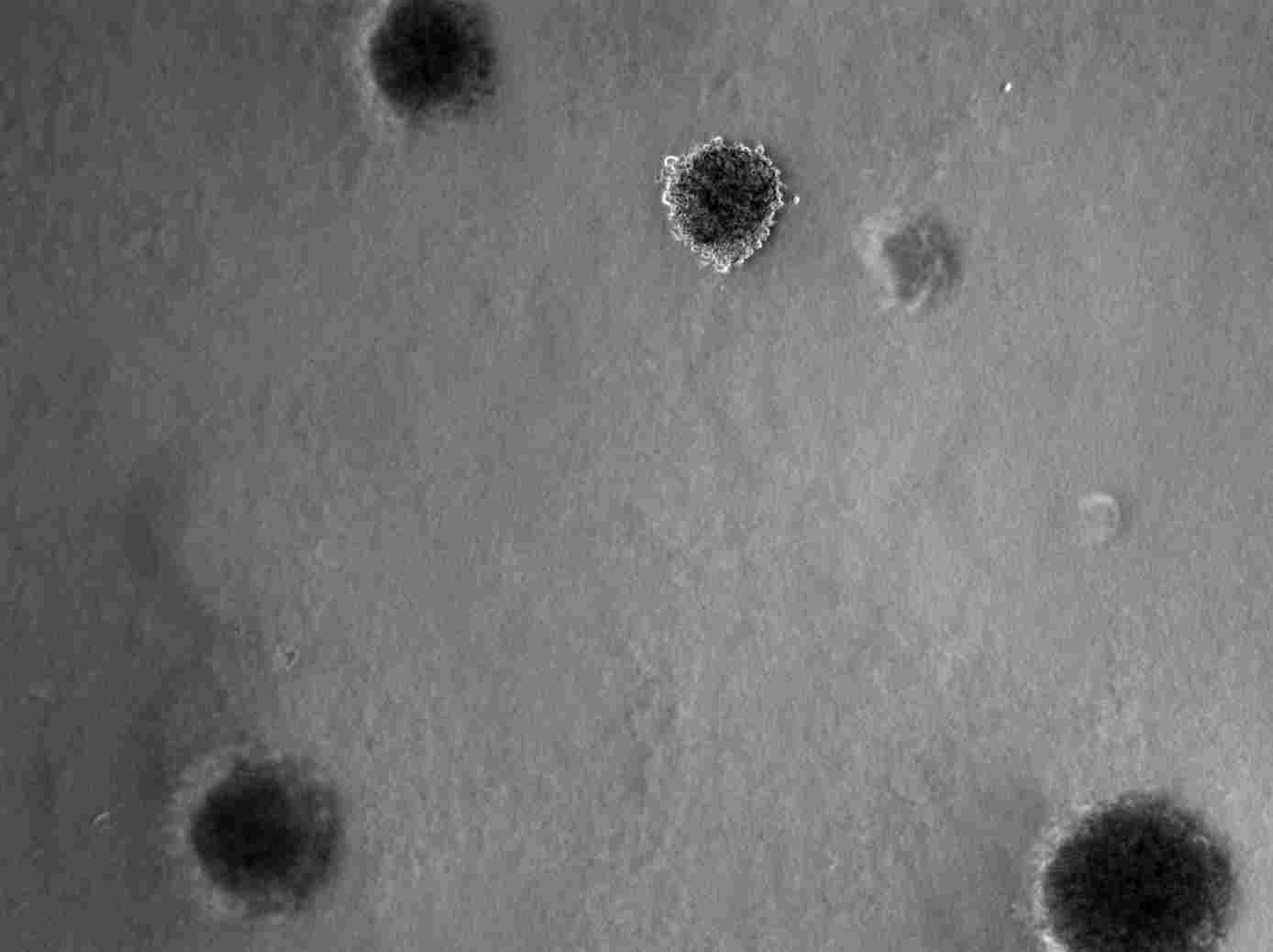

Supplement: S2 File — The raw data are presented in Raw data.zip. (ZIP) [file pone.0339611.s002.zip › Raw data/Figure 4/soft agar/day 14/4+EV-day14 (14).jpg]

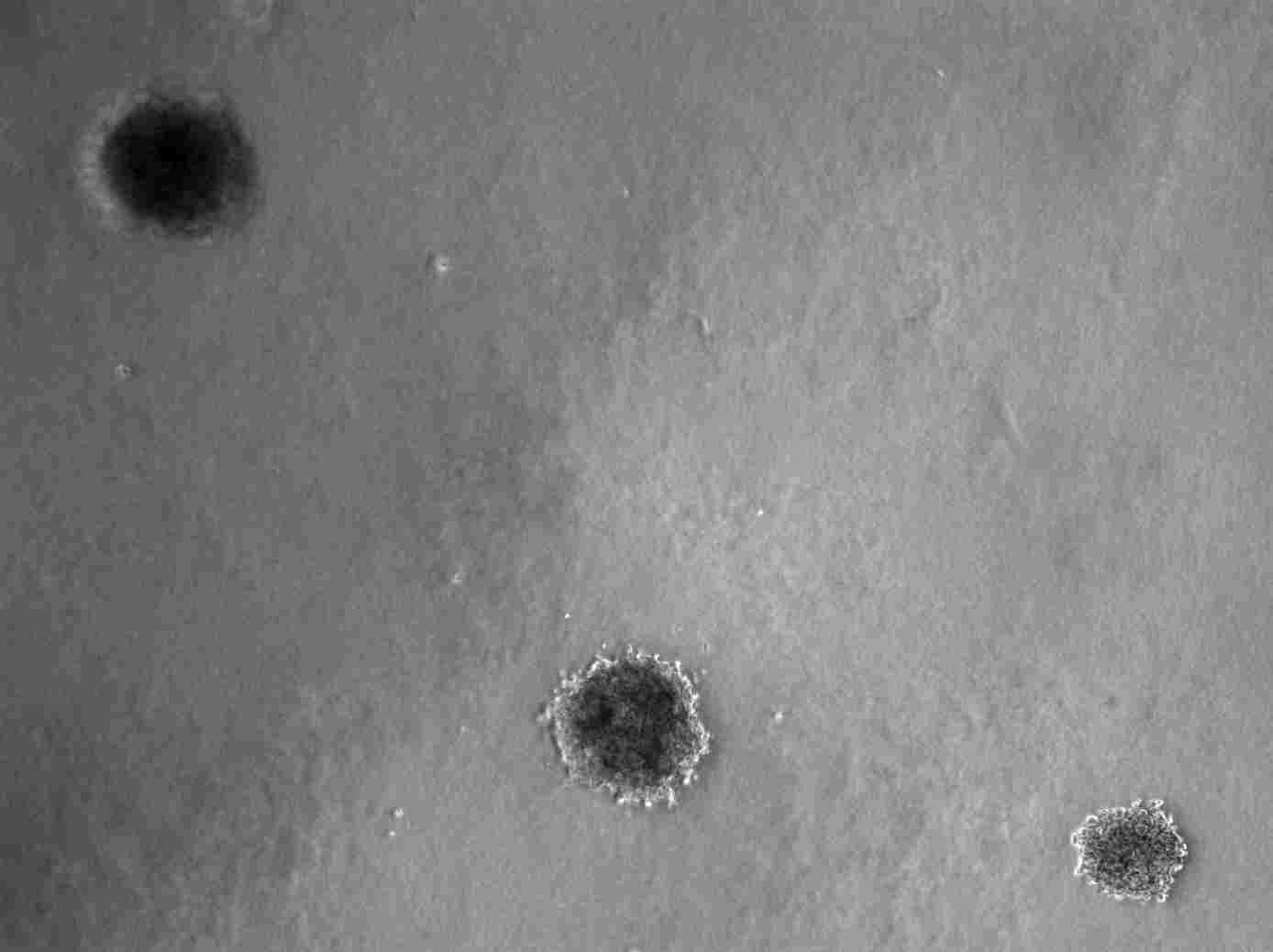

Supplement: S2 File — The raw data are presented in Raw data.zip. (ZIP) [file pone.0339611.s002.zip › Raw data/Figure 4/soft agar/day 14/4+EV-day14 (15).jpg]

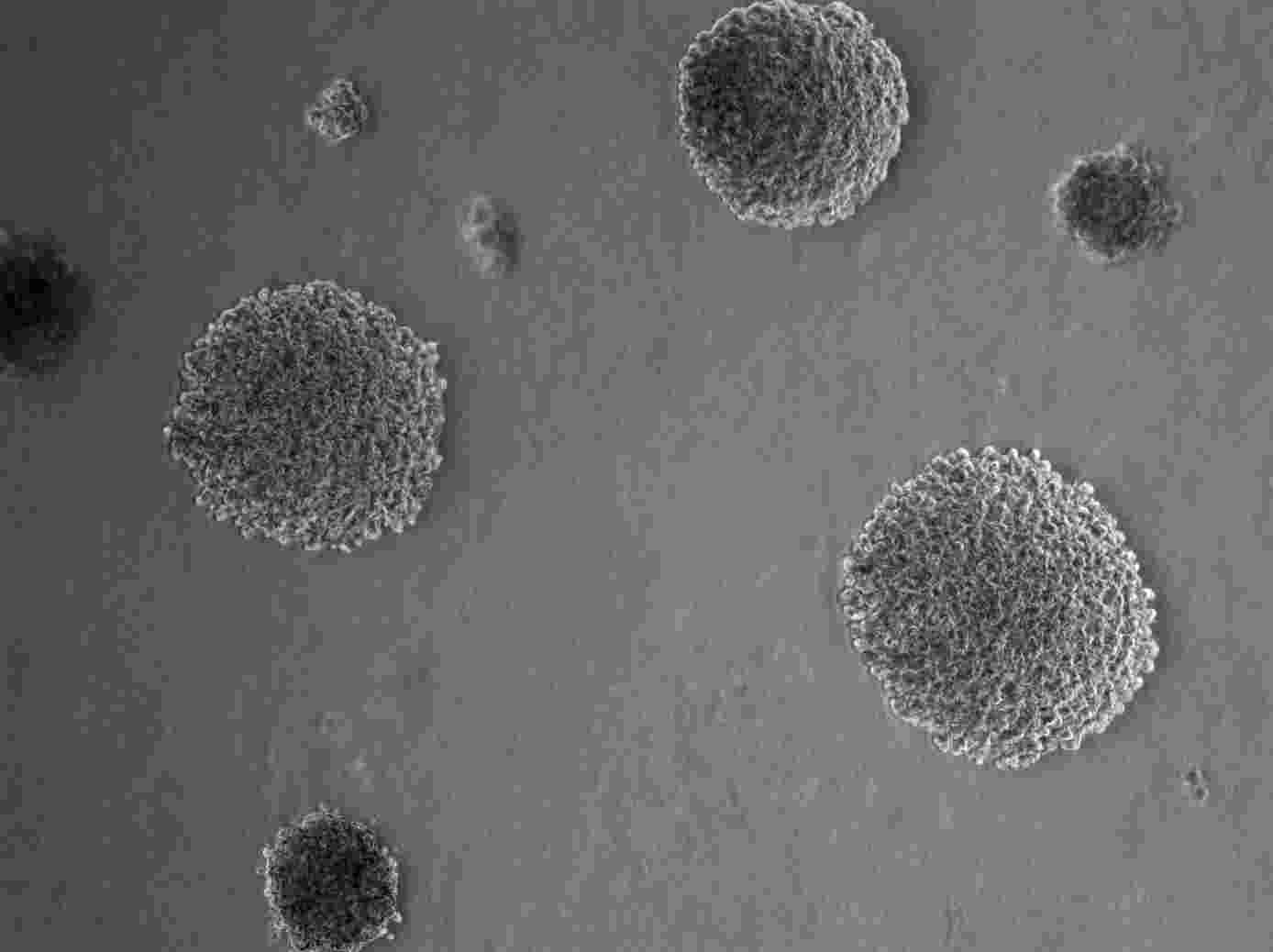

Supplement: S2 File — The raw data are presented in Raw data.zip. (ZIP) [file pone.0339611.s002.zip › Raw data/Figure 4/soft agar/day 14/4+EV-day14 (2).jpg]

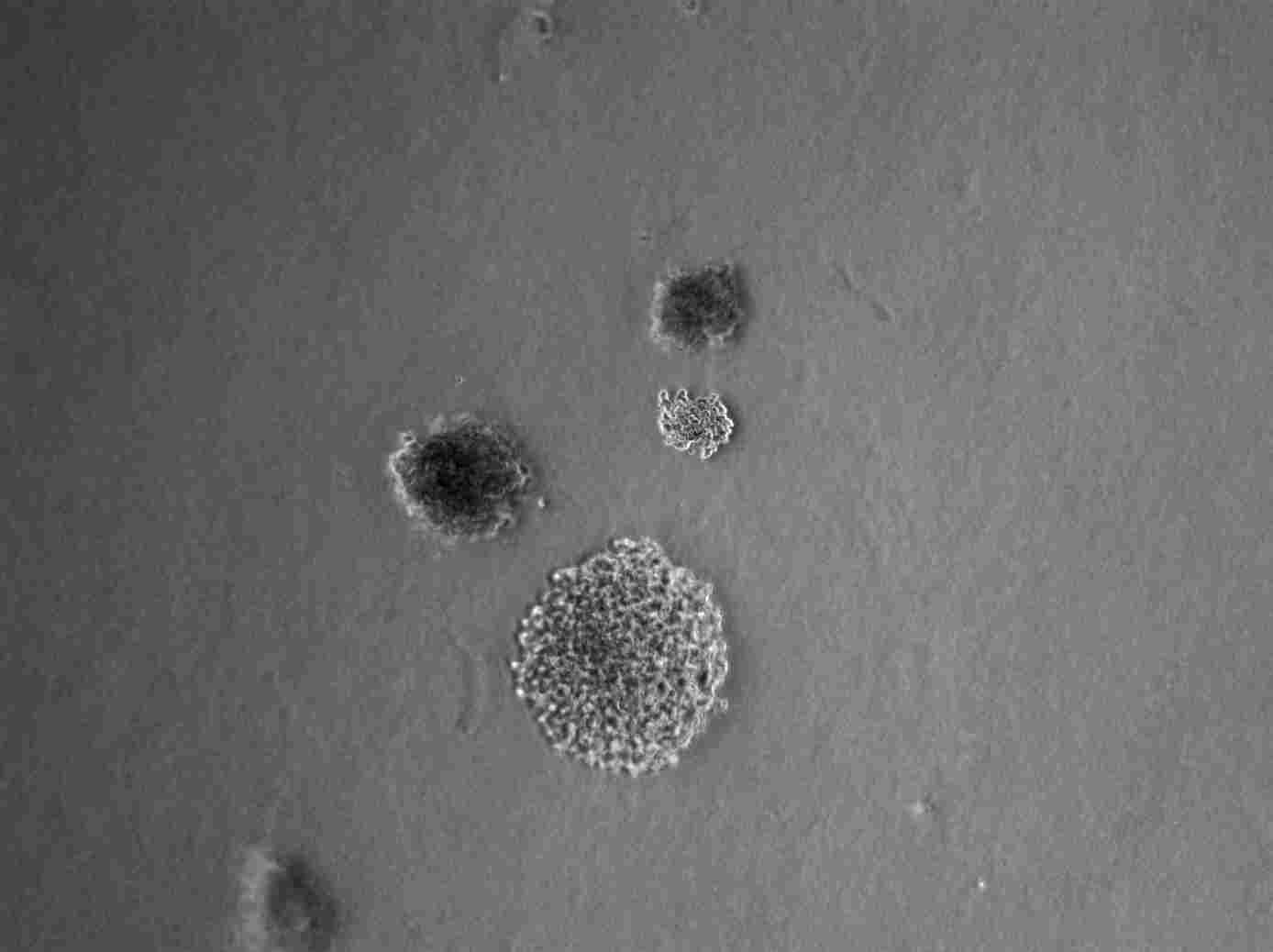

Supplement: S2 File — The raw data are presented in Raw data.zip. (ZIP) [file pone.0339611.s002.zip › Raw data/Figure 4/soft agar/day 14/4+EV-day14 (3).jpg]

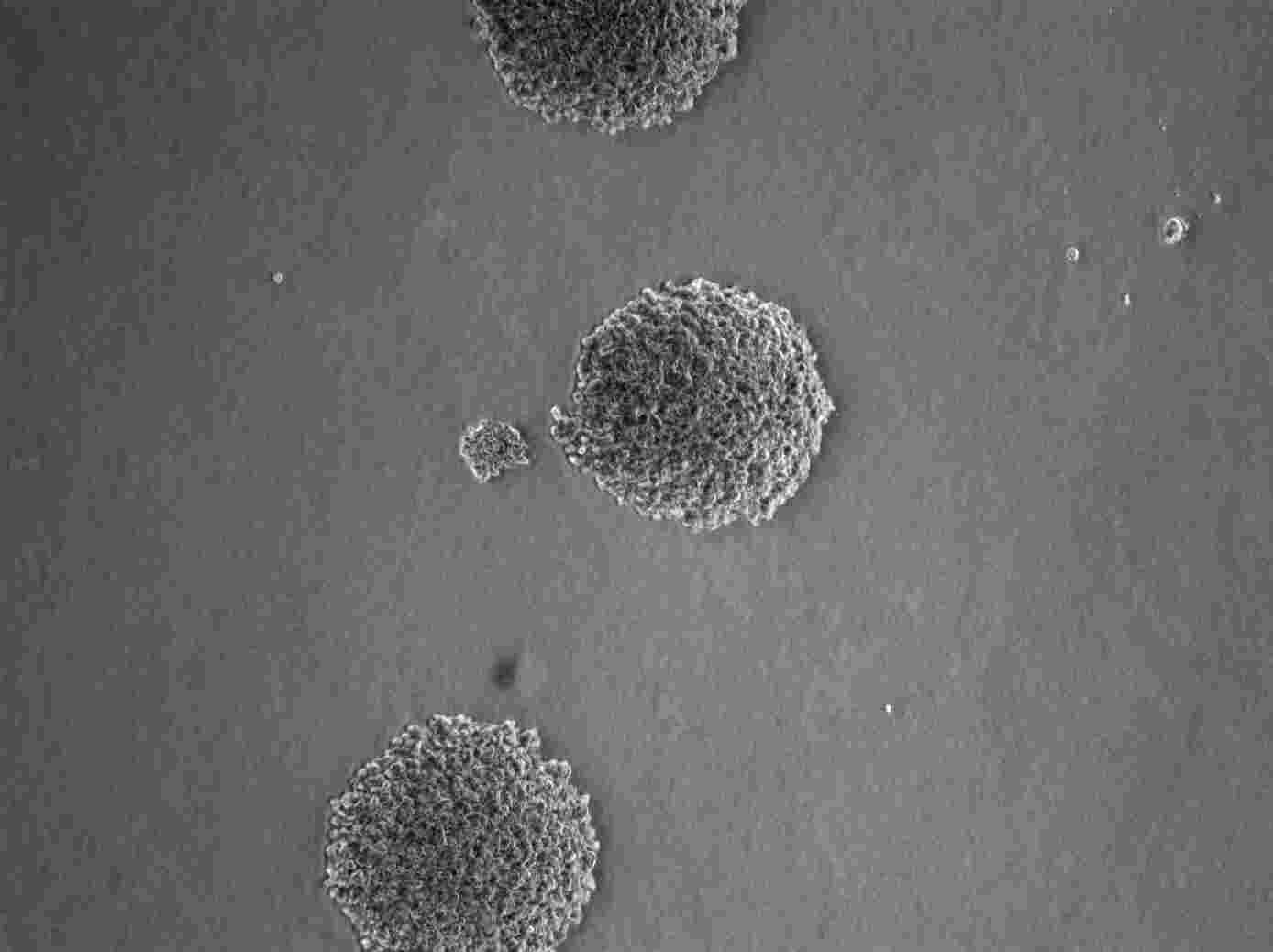

Supplement: S2 File — The raw data are presented in Raw data.zip. (ZIP) [file pone.0339611.s002.zip › Raw data/Figure 4/soft agar/day 14/4+EV-day14 (4).jpg]

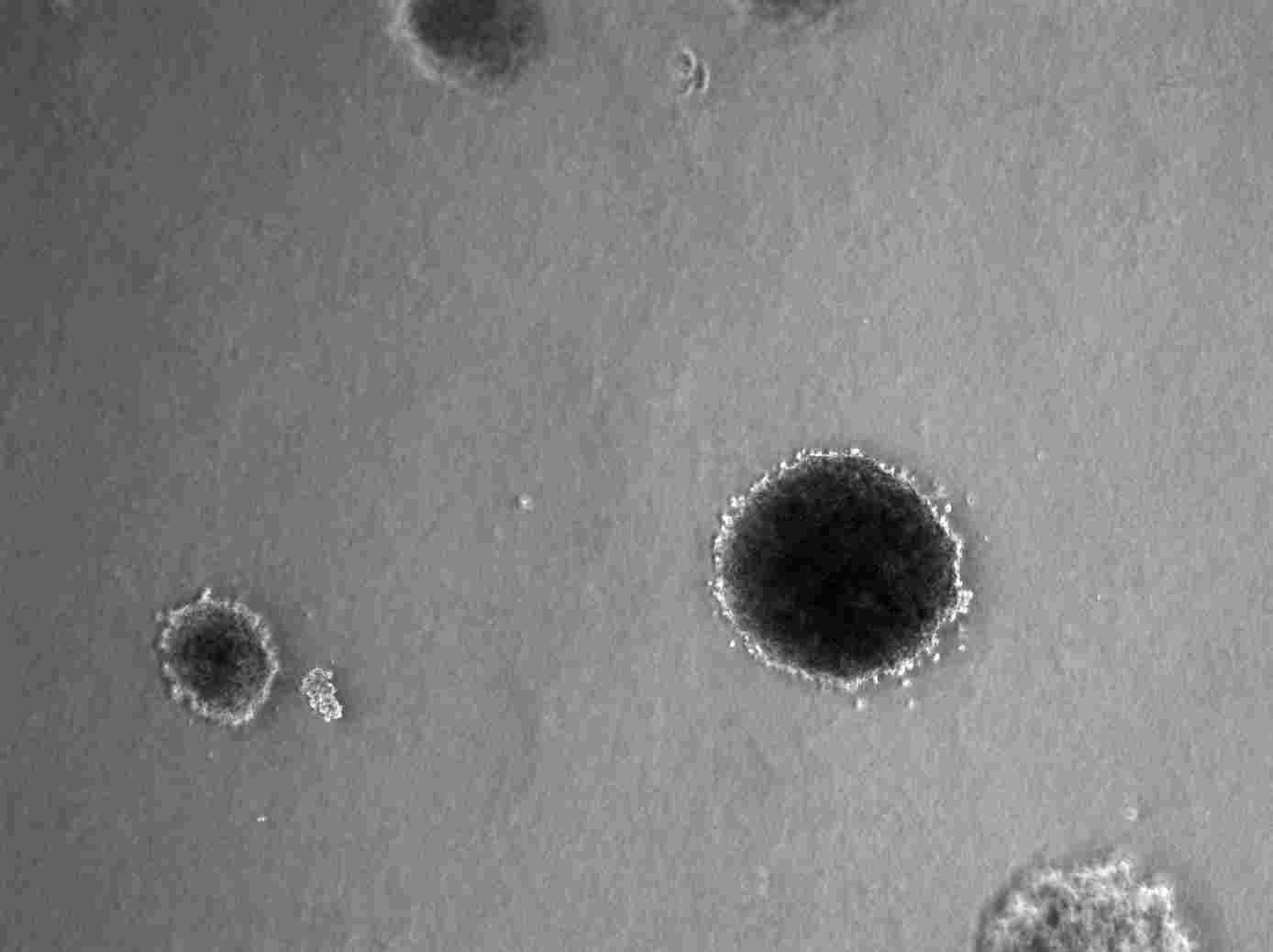

Supplement: S2 File — The raw data are presented in Raw data.zip. (ZIP) [file pone.0339611.s002.zip › Raw data/Figure 4/soft agar/day 14/4+EV-day14 (5).jpg]

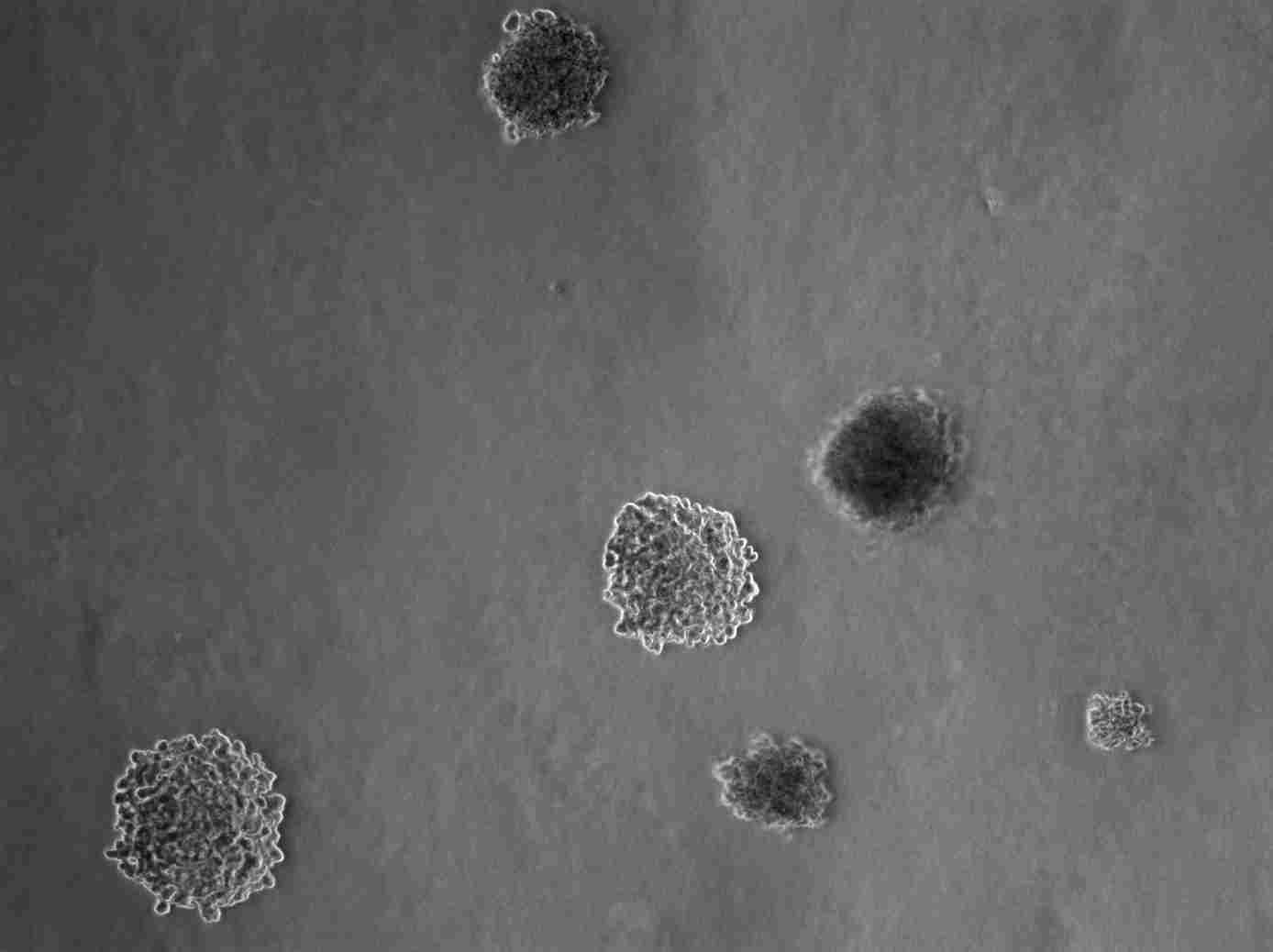

Supplement: S2 File — The raw data are presented in Raw data.zip. (ZIP) [file pone.0339611.s002.zip › Raw data/Figure 4/soft agar/day 14/4+EV-day14 (6).jpg]

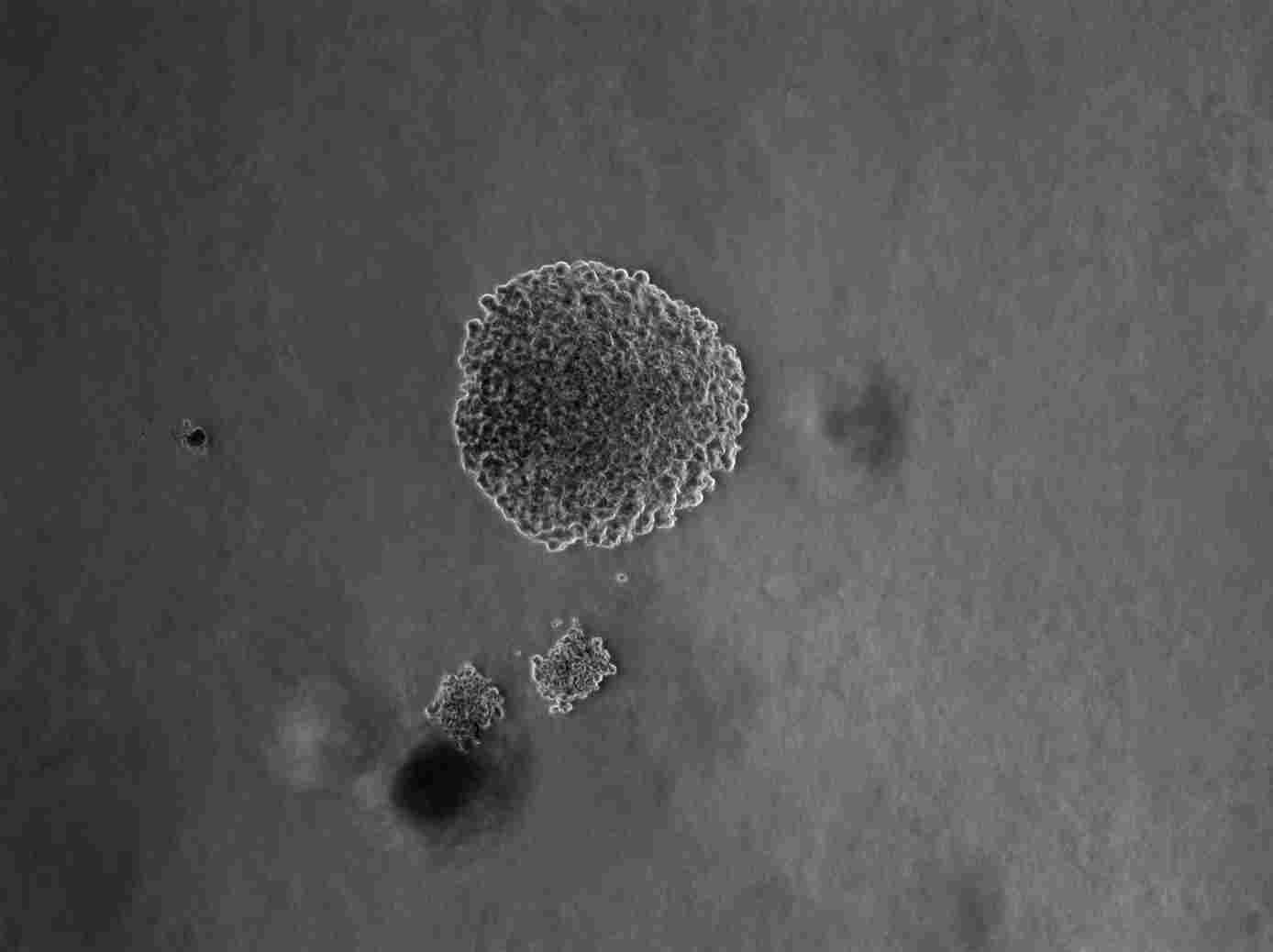

Supplement: S2 File — The raw data are presented in Raw data.zip. (ZIP) [file pone.0339611.s002.zip › Raw data/Figure 4/soft agar/day 14/4+EV-day14 (7).jpg]

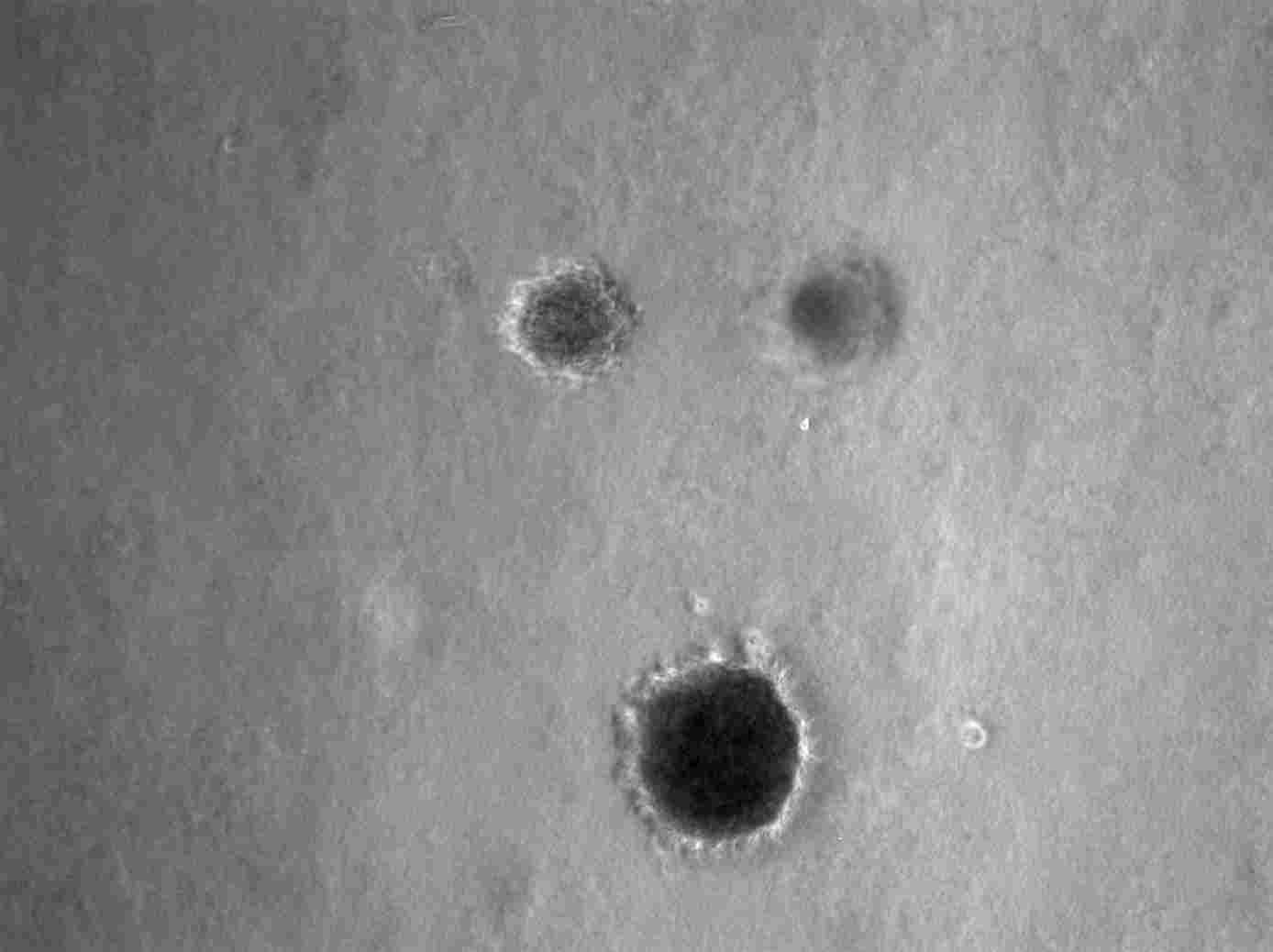

Supplement: S2 File — The raw data are presented in Raw data.zip. (ZIP) [file pone.0339611.s002.zip › Raw data/Figure 4/soft agar/day 14/4+EV-day14 (8).jpg]

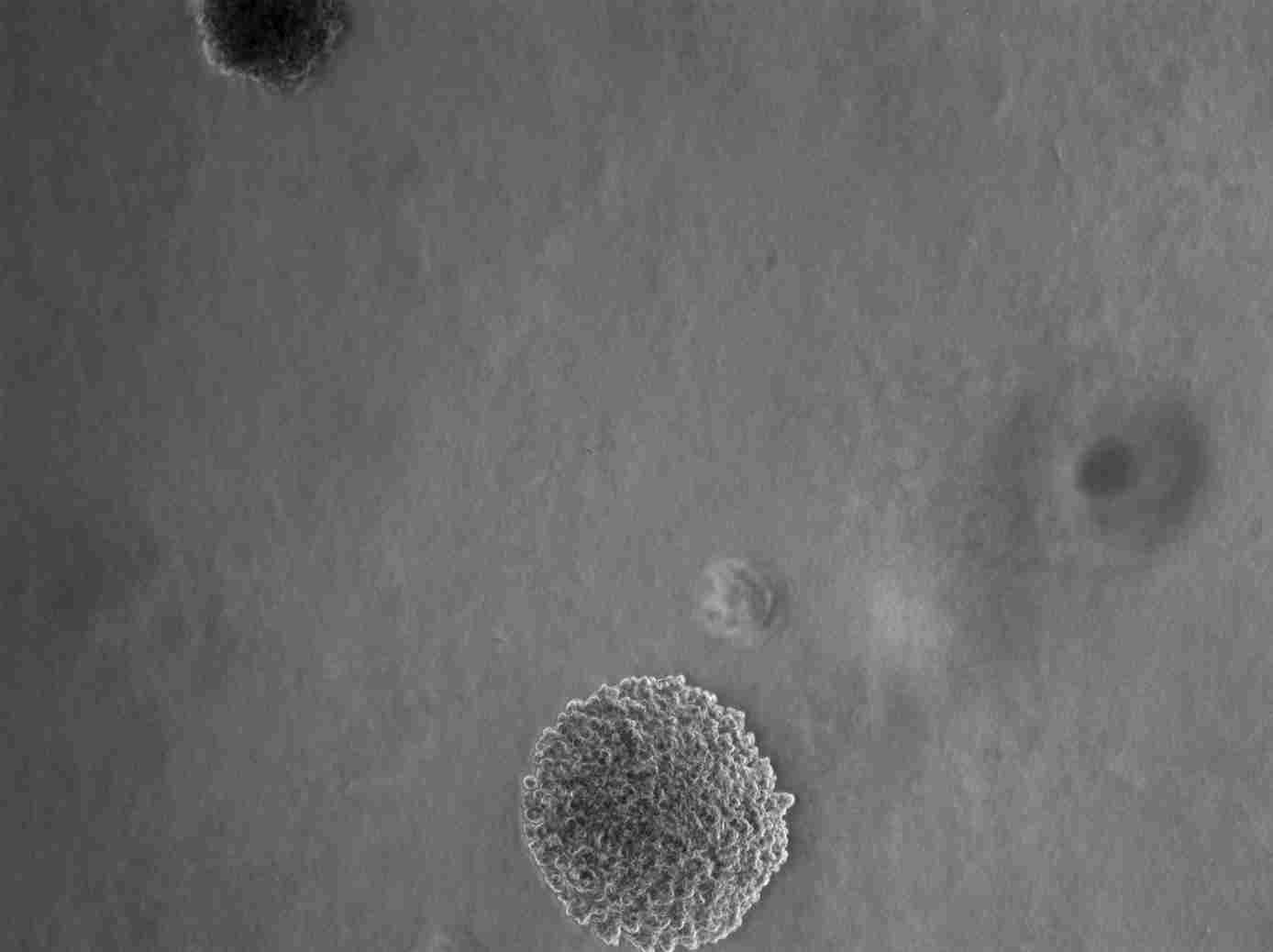

Supplement: S2 File — The raw data are presented in Raw data.zip. (ZIP) [file pone.0339611.s002.zip › Raw data/Figure 4/soft agar/day 14/4+EV-day14 (9).jpg]

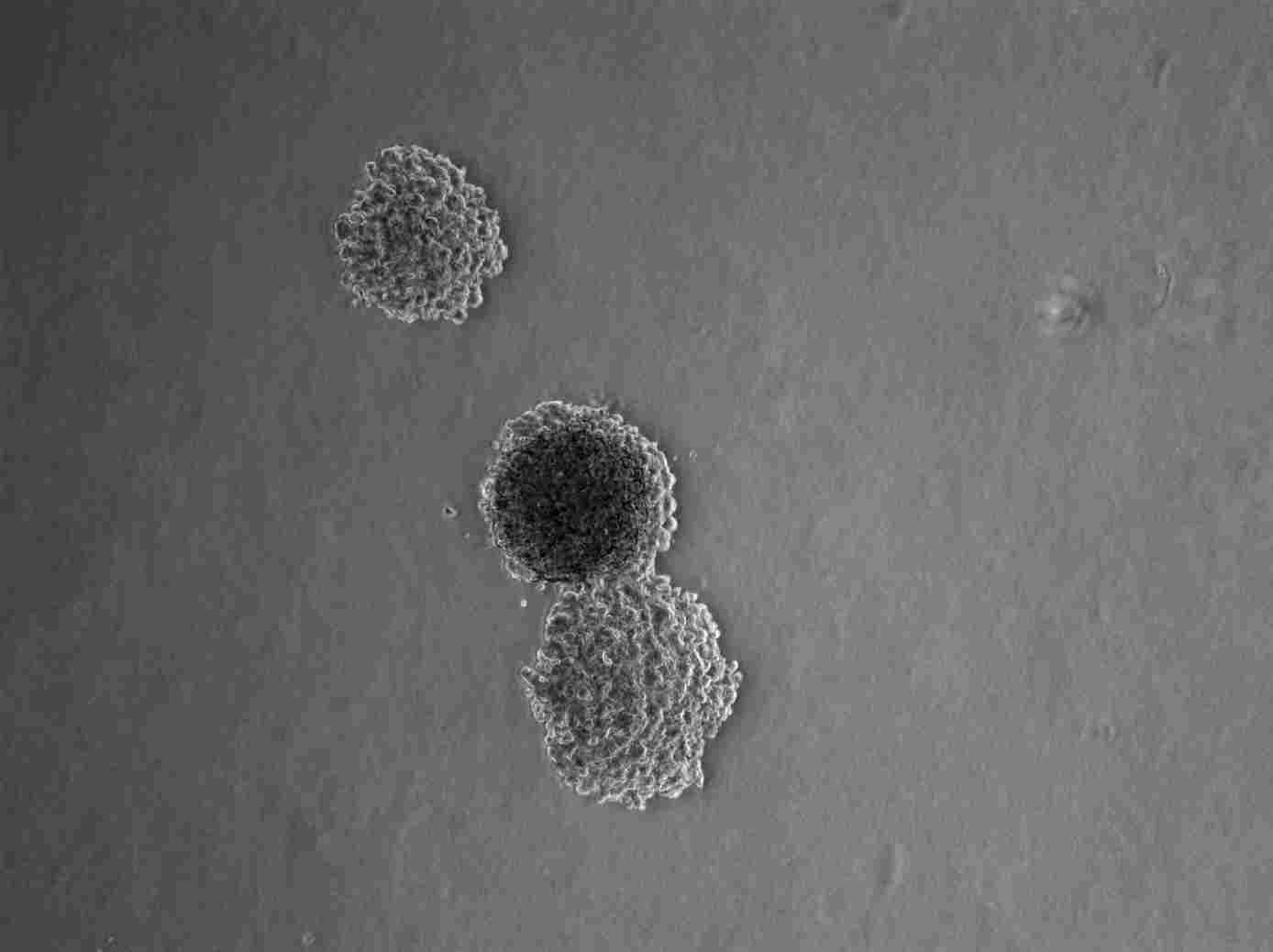

Supplement: S2 File — The raw data are presented in Raw data.zip. (ZIP) [file pone.0339611.s002.zip › Raw data/Figure 4/soft agar/day 14/4+EV-day14.jpg]

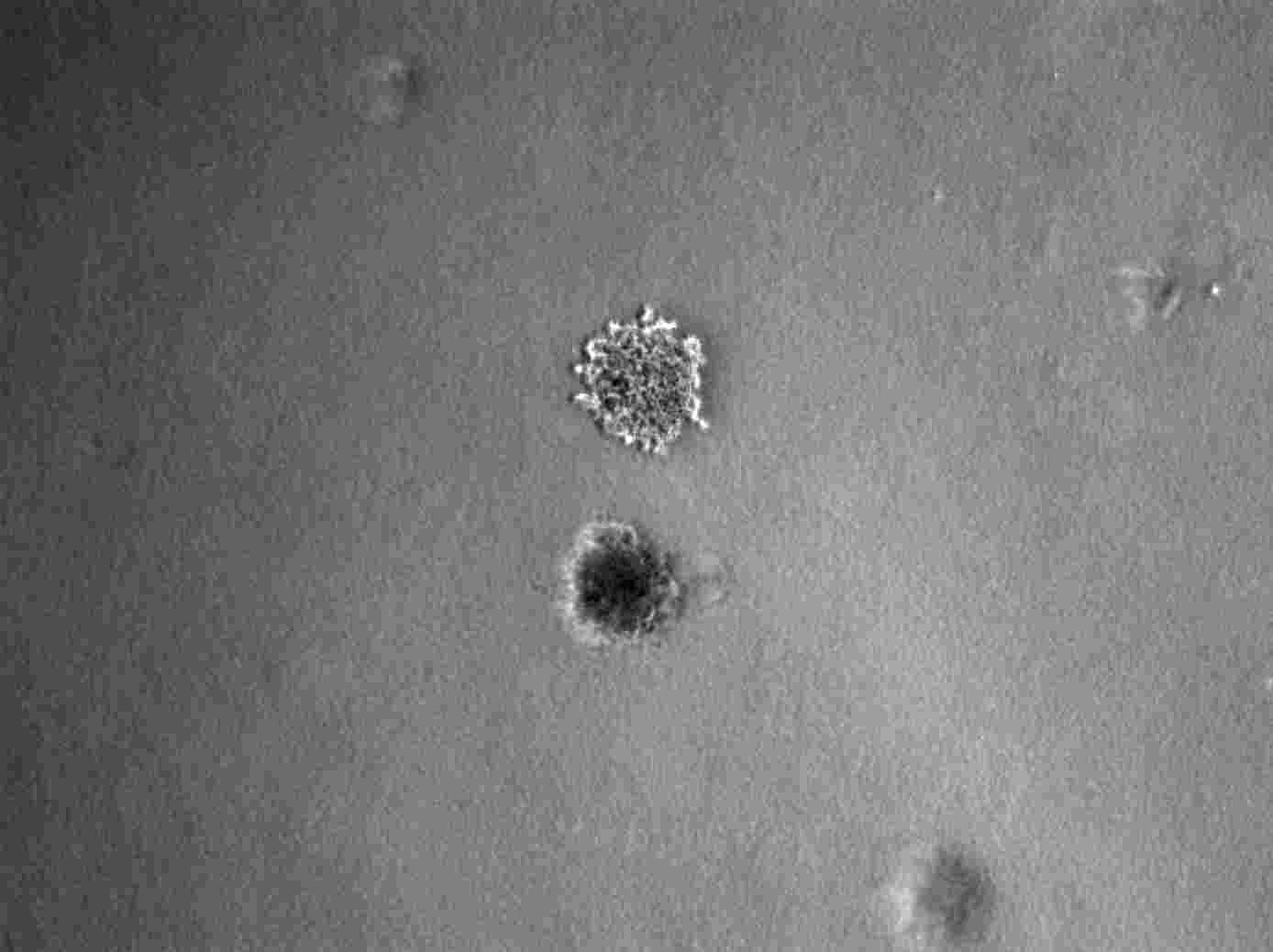

Supplement: S2 File — The raw data are presented in Raw data.zip. (ZIP) [file pone.0339611.s002.zip › Raw data/Figure 4/soft agar/day 14/4+OE-day14 (10).jpg]

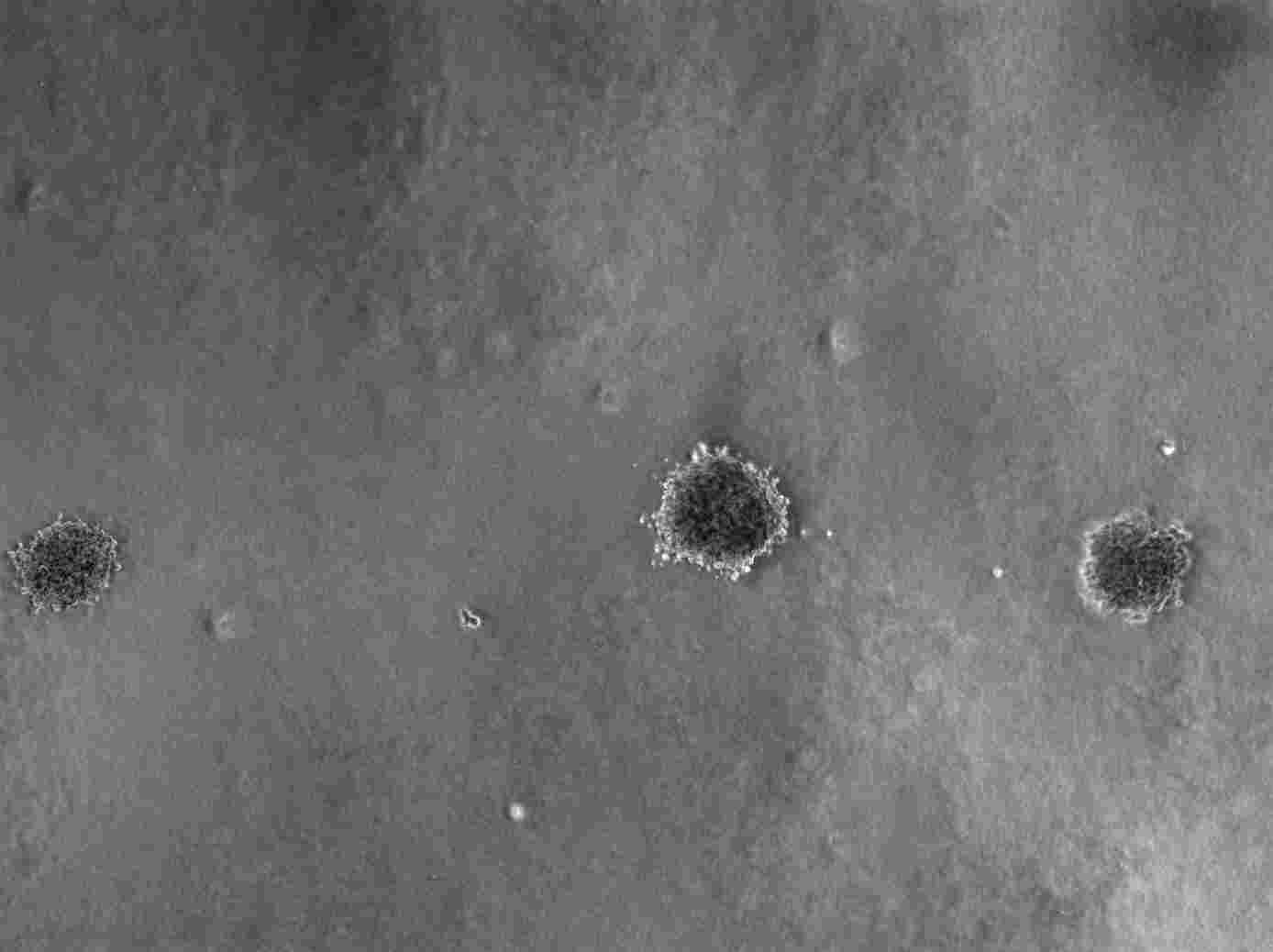

Supplement: S2 File — The raw data are presented in Raw data.zip. (ZIP) [file pone.0339611.s002.zip › Raw data/Figure 4/soft agar/day 14/4+OE-day14 (11).jpg]

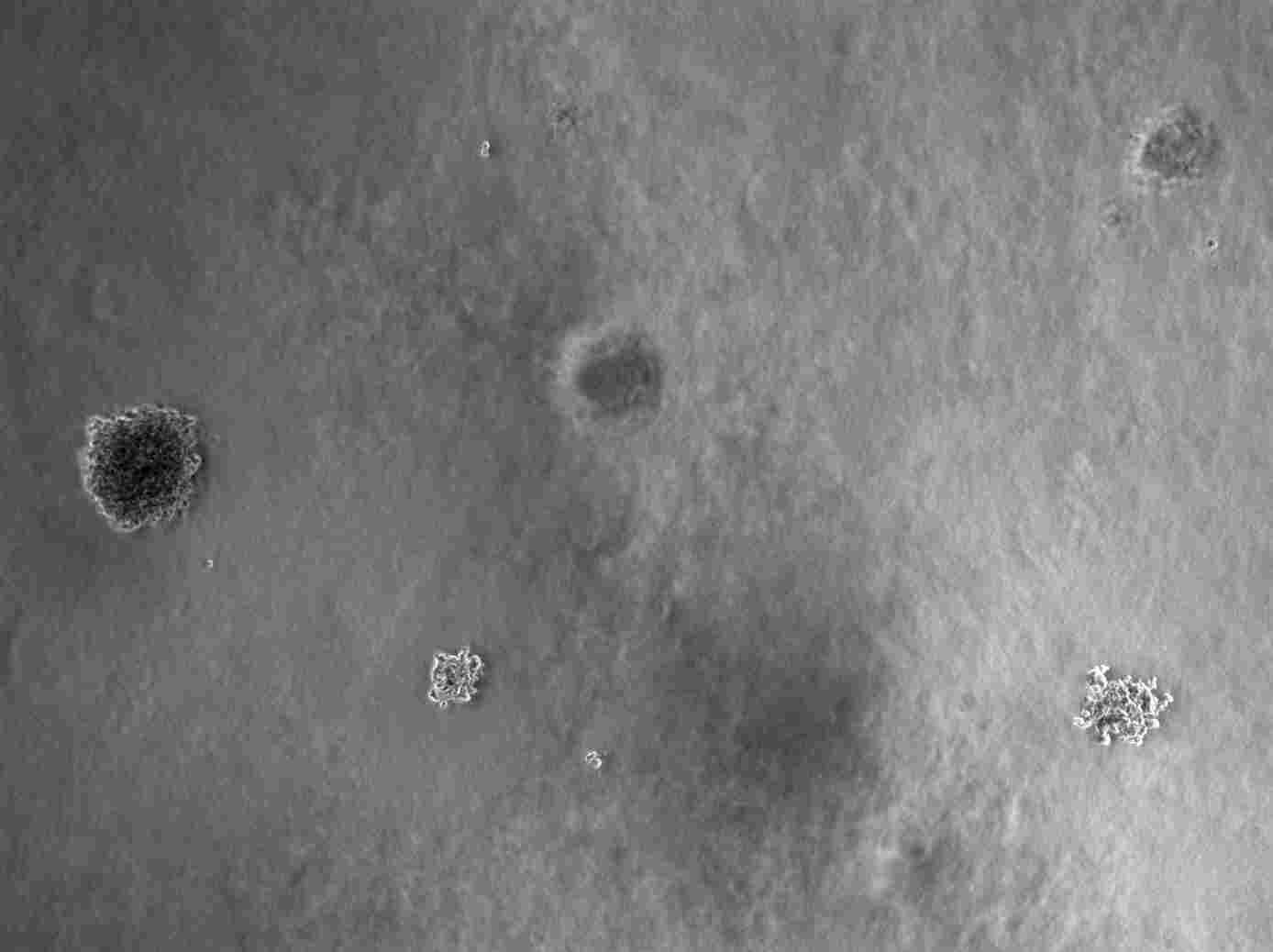

Supplement: S2 File — The raw data are presented in Raw data.zip. (ZIP) [file pone.0339611.s002.zip › Raw data/Figure 4/soft agar/day 14/4+OE-day14 (12).jpg]
